# Proteomic analysis of Buffalo milk somatic cells reveals metabolomic and immunological transitions during early lactation

**DOI:** 10.1038/s41598-025-08433-0

**Published:** 2025-07-02

**Authors:** Priyanka M. Kittur, Lija Satheesan, Narasimha Tanuj Gunturu, Yallappa M. Somagond, A. P. Madhusoodan, Ravi Kumar Gandham, Rani Alex, Ajay Kumar Dang

**Affiliations:** 1 Lactation and Immuno-Physiology Laboratory, Animal Physiology Division, National Dairy Research Institute, Karnal, Haryana India; 2 National Bureau of Animal Genetic Resources, Karnal, Haryana India; 3 National Research Centre on Mithun, Medziphema, Nagaland India; 4 National Dairy Research Institute, Karnal, Haryana India

**Keywords:** Colostrum, Composition, Hub-proteins, Milk somatic cells, LC-MS/MS, Proteomics, Metabolism, Innate immunity

## Abstract

Buffalo milk is renowned for its nutritional and functional properties. Milk somatic cells protect the mammary gland, contribute to the functionality of the udder, and also aid in the health and development of newborn calves, particularly during the critical early lactation period. However, proteomic changes in buffalo milk somatic cells during the transition from colostrum to mature milk remain poorly understood. This study was formulated to characterize the proteomic dynamics of buffalo milk somatic cells using Liquid chromatography-tandem mass spectrometry (LC-MS/MS) during colostrum-to-mature milk transition and to reveal shifts in metabolic and immune functions. A total of 4,429 high-confidence proteins were identified in the colostrum and milk of buffaloes. Up-regulated proteins [P_adj_<0.05, log_2_(Fold change, FC) ≥ 1.5] across different days of sampling were involved in metabolism of sugars, lipids, and amino acids, pentose-phosphate pathway, insulin-signaling, biosynthesis of amino acids and cofactors, and ubiquitin-proteasome system. Down-regulated proteins [P_adj_<0.05, log_2_(FC) ≤ 0.5] were associated with lipid transport, aldosterone synthesis and secretion, mineral balance, complement-coagulation system, antigen processing and presentation, and mRNA processing. A notable shift in hub proteins was detected, and selected ones were validated by real-time qPCR. These findings highlight significant changes in the proteome profile, biological functions, and specific pathways in milk somatic cells during early lactation in buffaloes. In conclusion, milk somatic cells contribute not only to mammary immunity but also to the nutritional support of the growing calf.

**Supplementary Information:**

The online version contains supplementary material available at 10.1038/s41598-025-08433-0.

## Introduction

Buffaloes significantly contribute to global milk production, are found on all continents, and are spread across 77 countries. They produce over 75 million metric tons of milk annually and maintain a steady growth rate of about 3%, making them the second-largest source of milk worldwide^1^. Buffalo milk contains higher fat, total solids, proteins, caseins, lactose, and ash levels than other farm animals. Colostrum and milk are vital sources that provide essential nutrients and bioactive factors to support life outside the womb, promote neonatal growth, and aid in adapting to external challenges^2^. Colostrum contains immunostimulatory components with variable cytological qualities that function as antimicrobial factors under lysis conditions^3^. During lactation, the cells of the mammary gland are responsible for synthesizing and transporting the diverse components to the milk, as well as responding to maternal and infant signals to maintain lactational viability. Somatic cells present in milk are mainly a mix of milk-producing mammary epithelial cells (MECs) and polymorphonuclear (PMN) leukocytes, serving as non-invasive indicators of mammary health and milk quality^4^. Short-term gene expression changes make somatic cells motile, allowing them to pass through weak interepithelial junctions of the intestinal mucosa and transfer to the infant before gut closure, boosting mucosal and systemic immunity^5^.

The MECs contribute to 2 to 15% of the somatic cell count (SCC), express pathogen recognition receptors (toll-like receptors, TLRs), and also synthesize antimicrobial proteins along with milk. Maternal colostral cells accelerate the development of adaptive immunity by enhancing the antigen-presenting capabilities of monocytes and lymphocytes, exerting long-term effects on the newborn calf’s immune system. Maternal vaccination enhances neonatal defenses against specific pathogens by transferring antigen-specific lymphocytes in colostrum^6^. Earlier studies have been undertaken by us, to estimate the immunological activity of the colostrum during its transition to milk in Murrah buffaloes^7,8^along with growth factors and stress biomarkers in dairy cows^9,10^. Macrophage activating factor has been synthesized from bovine colostrum, and its impacts on in-vitro phagocytic activity of different tissue macrophages were also studied^11^.

Research suggests milk proteins have various physiological activities, including immune regulation, growth, and metabolic balance. Proteomics has been used to identify and characterize proteins, evolving from a qualitative approach focusing on protein identification to a quantitative approach that compares protein expression levels^12^ and aims to discover how proteins function and interact. Advanced “omics” technologies and high-throughput techniques like LC-MS/MS have identified numerous proteins influencing lactation. Some studies in farm animals have characterized the proteome of colostrum, milk, milk fat globular membrane, milk-derived exosomes, and mammary epithelial cells^2,12–17^ and reported changes in protein abundances associated with important functions like immunity, complement-coagulation cascade, enzymes involved in digestion, growth, and maturation of infant organs, and mammary gland metabolism.

Proteomic studies in Sahiwal cows have explored immune responses and mastitis biomarkers in milk somatic cells^18^. Bulk transcriptomics in humans revealed upregulated insulin signaling, lactose, and fatty acid synthesis during early lactation but lacked cell-type specificity^19^. Studying key genes in bovine mammary cells can uncover lactation mechanisms and biomarkers influenced by physiology, nutrition, and management. Despite challenges, omics integration holds promise for improving lactation^20^. While cellular fractions contribute significantly (30–32%) to milk proteins^17^buffalo somatic cell proteomics dynamics from colostrum to mature milk remain understudied. This study examines protein interactions in the somatic cells at the initial lactation stages of buffaloes to understand functional shifts from colostrum to mature milk.

This study was designed to quantify and compare the buffalo colostrum and milk somatic cell proteome on different days postpartum with the changes occurring in composition, SCC, and differential leukocyte count **(**DLC) with the hypothesis that somatic cell proteome of buffalo milk exhibits dynamic and stage-specific changes during the transition from colostrum to mature milk, reflecting physiological adaptations in the mammary gland and the evolving immunological and nutritional needs of the neonate. We assessed the qualitative and quantitative variation in milk somatic cells proteome of 6 buffaloes from day 1 to day 15 of the post-partum, colostrum, transitional milk, and mature milk somatic cells using repeated sampling.

## Results

### Composition, somatic cell count (SCC), and differential leukocyte count (DLC) of colostrum and milk

Solids not fat (SNF, %), density (kg/dL), protein (%), and salts (%) in colostrum and milk samples were significantly (*P* < 0.05) higher on day 1 (D1), showing a decreasing trend in subsequent collection days. Fat (%) exhibited a significant (*P* < 0.05) decrease in the day 15 (D15) milk sample. Conductivity (mS/cm) was found to be elevated on D1 and day 4 (D4), while lactose (%), and pH were significantly (*P* < 0.05) lower on D1. SCC (10^3^ cells/mL) was significantly (*P* < 0.05) different and higher on D1 followed by D4, day 7 (D7), and D15. Cell viability (%) demonstrated a consistent increase from D1 to D15. Neutrophils and macrophages (%) were significantly (*P* < 0.05) higher on D1, whereas lymphocytes (%) were significantly (*P* < 0.05) higher at D15 (Table 1).

**Table 1:** Composition, somatic cell count (SCC, 10^3^ cells/mL), cell viability (%), and differential leukocyte count (DLC) of colostrum (D1), early transitional (D4), late transitional (D7), and mature milk (D15) in Murrah buffaloes.

	D1	D4	D7	D15
**Fat (%)**	8.61^b^ ± 0.71	7.96^ab^ ± 0.45	7.35^ab^ ± 0.37	6.77^a^ ± 0.32
**SNF (%)**	14.14^b^ ± 0.84	11.07^a^ ± 0.31	11.05^a^ ± 0.37	10.60^a^ ± 0.18
**Density (kg/dL)**	48.68^b^ ± 2.95	34.87^a^ ± 1.50	33.35^a^ ± 1.27	32.27^a^ ± 0.85
**Protein (%)**	12.98^d^ ± 0.46	9.05^c^ ± 0.11	7.70^b^ ± 0.18	5.96^a^ ± 0.17
**Lactose (%)**	3.17^a^ ± 0.51	4.70^b^ ± 0.15	5.32^bc^ ± 0.17	5.82^c^ ± 0.18
**Salts (%)**	1.27^b^ ± 0.07	0.91^a^ ± 0.02	0.90^a^ ± 0.04	0.87^a^ ± 0.01
**Conductivity (mS/cm)**	5.10^b^ ± 0.16	4.68^b^ ± 0.27	3.84^a^ ± 0.15	3.68^a^ ± 0.14
**pH**	6.45^a^ ± 0.06	6.55^ab^ ± 0.09	6.62^ab^ ± 0.03	6.72^b^ ± 0.10
**SCC (×10**^**3**^ **cells/mL)**	476.91^d^ ± 35.64	373.16^c^ ± 25.56	254.25^b^ ± 25.24	129.08^a^ ± 14.94
**Cell viability (%)**	47.83^a^ ± 2.14	69.08^b^ ± 2.48	82.25^c^ ± 1.56	90.08^d^ ± 1.66
**Neutrophils (%)**	20.50^b^ ± 0.86	22.08^b^ ± 0.89	17.16^a^ ± 0.82	15.58^a^ ± 0.95
**Lymphocytes (%)**	32.75^a^ ± 0.97	35.25^a^ ± 0.97	41.08^b^ ± 1.48	45.16^c^ ± 1.31
**Macrophages (%)**	46.75^b^ ± 1.52	42.66^ab^ ± 1.43	41.75^ab^ ± 1.54	39.25^a^ ± 1.40

Means with different superscripts (a, b, c, and d) differ significantly (*P* < 0.05) between the days.

## Proteomic analysis of colostrum and milk somatic cells

**Fig. 1:**
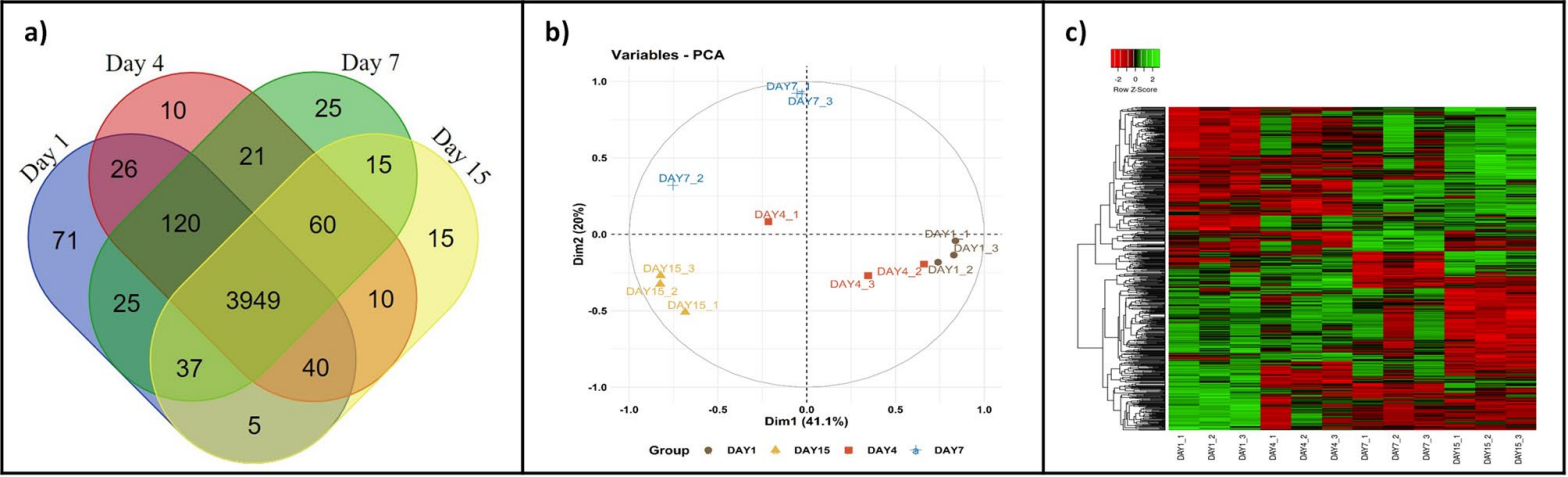
Represents the Venn diagram (**a**), Principle component analysis, PCA (**b**), and Heatmap and dendrogram hierarchical cluster analysis (**c**) of the entire set of somatic cell proteins isolated from colostrum (day 1), early transitional (day 4), late transitional (day 7), and mature milk (day 15) of buffaloes.

LC-MS/MS quantitative proteomic analysis of buffalo colostrum and milk somatic cells homogenate D1, D4, D7, and D15 identified 4429 proteins. Among the high-confidence proteins (≥ 1 high-quality peptide-spectrum match [PSM/s], ≥ peptide, P_adj_<0.05, and FDR < 0.01), 3949 were found to be common across all four days of the initial lactation. The Venn diagram (Fig. 1a**)** visually portrays the distinct proteins identified across the days, highlighting their overlap and uniqueness. Notably, 71 proteins were uniquely expressed on D1, while D4, D7, and D15 had 10, 25, and 15 unique proteins, respectively. The principal component analysis **(**PCA) plot (Fig. 1b) represents the distinction between the biological replicates demonstrating the variations in protein expression profiles over the sampling days.

## Differentially expressed proteins (DEPs) of colostrum and milk somatic cells

**Fig. 2:**
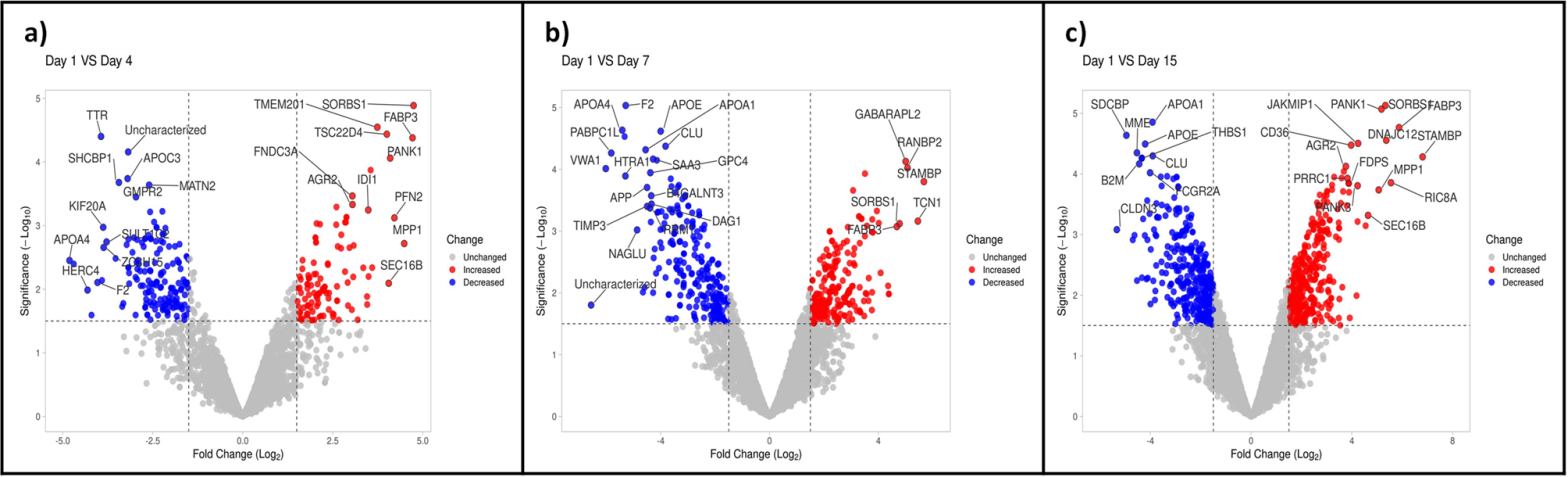
Volcano plot displaying differentially expressed proteins of day 1 vs. day 4 (**a**), day 1 vs. day 7 (**b**), and day 1 vs. day 15 (**c**). The y-axis corresponded to the mean abundance value log_10_ (P value/significance), and the x-axis displayed the log_2_ fold change value. The red and blue dots represent the significant differentially expressed proteins (*P* < 0.05) in buffalo milk somatic cells. The grey dots represent the proteins whose abundance level did not reach statistical significance during the respective days.

Detecting significant changes in protein abundance typically involves analyzing proteomic data to identify proteins whose levels have changed under different experimental conditions. These have been depicted in the heatmap (Fig. 1c), indicating the hierarchical clustering of milk somatic cell proteins across sampling days. The bar color in the heatmap represents a logarithmic scale of significant proteins, ranging from − 2 to 2. Out of the specific proteins identified, 100, 196, and 309 proteins had significant up-regulation (P_adj_<0.05, log_2_ (FC) ≥ 1.5) on days 4, 7, and 15, respectively, compared with day 1. Conversely, 179, 291, and 513 proteins showed significant down-regulation (P_adj_<0.05, log_2_(FC) ≤ 0.5) on days 4, 7, and 15, respectively, compared with day 1. The top significant DEPs identified on different days are represented in the volcano plot (Fig. 2).

## Protein-protein interaction (PPI) analysis of DEPs

**Fig. 3:**
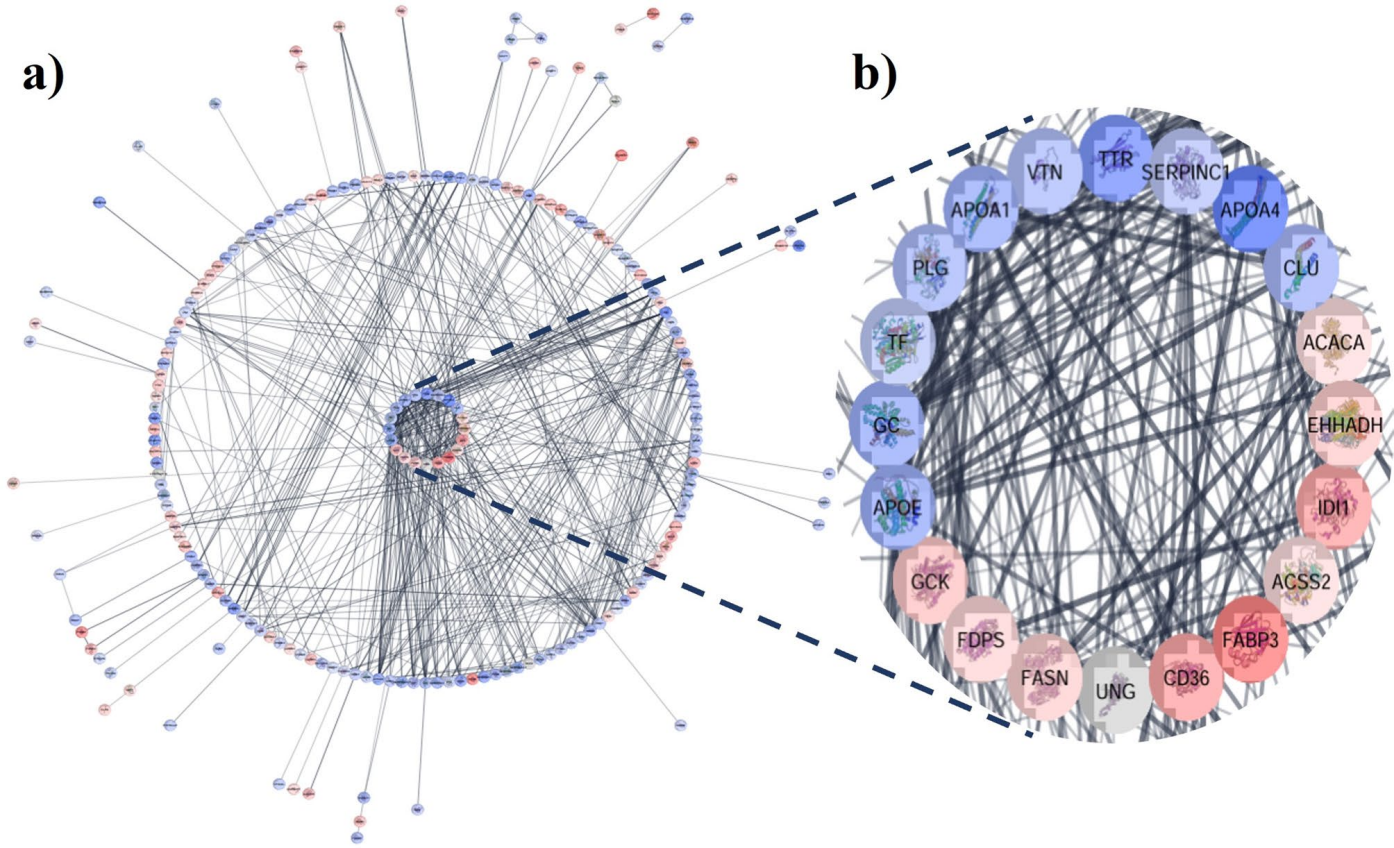
PPI network analysis of the differentially expressed proteins of day 1 vs. day 4 (D1 VS D4) in buffalo milk somatic cells. The red nodes represent the upregulated proteins; the blue nodes represent the downregulated proteins (*P* < 0.05). Proteins with ≤ 2 interactions are hidden (**a**). The top 10 hub proteins of both up and downregulated proteins (**b**). The darker the color of the protein, the higher the log_2_FC.

STRING analysis of D1 vs. D4 DEPs indicated significant (*P* < 0.05) hubs involved in crucial biological pathways such as metabolic pathways, complement and coagulation cascades, thyroid hormone synthesis, PPAR signaling pathway, cholesterol and carbon metabolism, mineral absorption, vitamins, carbohydrate and fat metabolism, endocrine and other factor-regulated calcium reabsorption **(Supplementary Fig. ****S1**** online)**. Regarding D1 vs. D7, other significant (*P* < 0.05) hubs were identified, which are involved in antigen processing and presentation, nucleotide metabolism, ECM-receptor interaction, and aldosterone synthesis and secretion (**Supplementary Fig. S2 online**). The PPIs of D1 vs. D15 highlighted significant (*P* < 0.05) pathways regulating amino sugar and nucleotide sugar metabolism, proteasome, biosynthesis of cofactors, glucagon signaling, cholesterol, propanoate, fructose, and mannose metabolism, and glycolysis/gluconeogenesis **(Supplementary Fig. S3 online)**. Cytoscape visualization identified the top 10 hub proteins from D1 vs. D4 **(**Fig. 3**)**, D1 vs. D7 **(Supplementary Fig. S4 online)**, and D1 vs. D15 **(Supplementary Fig. S5 online)**. The mapped proteins were able to interconnect to form the networks, revealing the strong functional relationship between the DEPs.

## Gene ontology and pathway analysis of DEPs

**Fig. 4:**
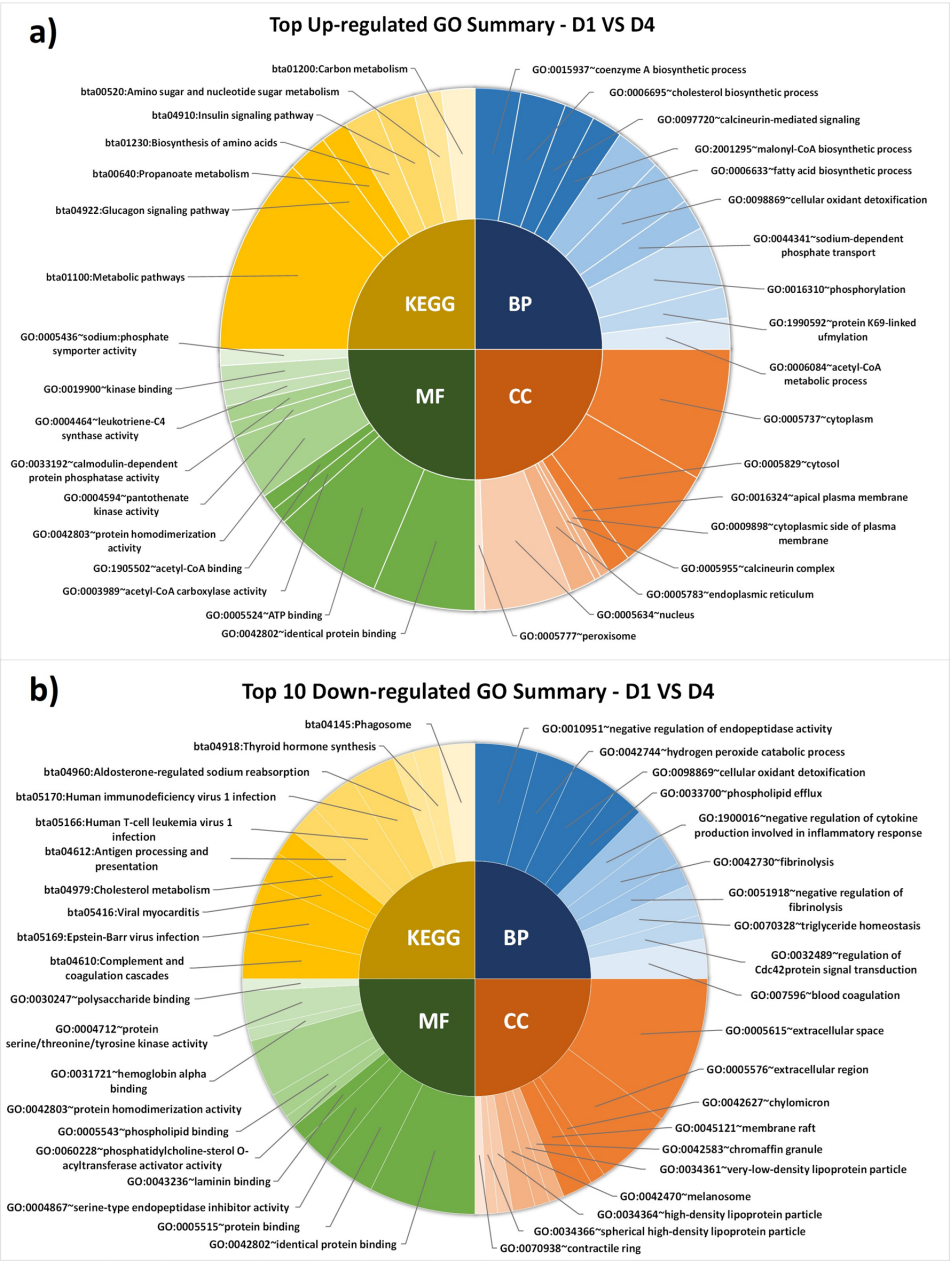
Top significant (*P* < 0.05) GO and pathway of up-regulated proteins (**a**) and down-regulated proteins (**b**) on D1 vs. D4 (day 1 vs. day 4).

The functional analysis of up-regulated somatic cell proteins on D1 vs. D4 revealed significant (*P* < 0.05) gene ontology terms like biological processes (BPs) such as biosynthetic processes of coenzyme A, cholesterol, malonyl-CoA, and FA biosynthetic process; molecular functions (MFs) such as identical protein binding, adenosine triphosphate (ATP) binding, acetyl-coA carboxylase activity, acetyl-coA binding, and protein homodimerization activity; significant (*P* < 0.05) KEGG pathways were in metabolic pathways, glucagon signaling pathway, propanoate metabolism, biosynthesis of amino acids, and insulin signaling pathway **(**Fig. 4a**).** In contrast, the functional annotations of down-regulated proteins revealed the significant (*P* < 0.05) BPs such as phospholipid efflux, negative regulation of cytokine production involved in the inflammatory process and fibrinolysis, triglyceride (TG) homeostasis, and blood coagulation; MFs associated with identical protein binding, and phosphatidylcholine-sterol O-acyltransferase activator activity; significant (*P* < 0.05) KEGG pathway involved in complement and coagulation cascades, viral infections, cholesterol metabolism, antigen processing and presentation, aldosterone-regulated sodium reabsorption, and thyroid hormone synthesis **(**Fig. 4b**).**

**Fig. 5:**
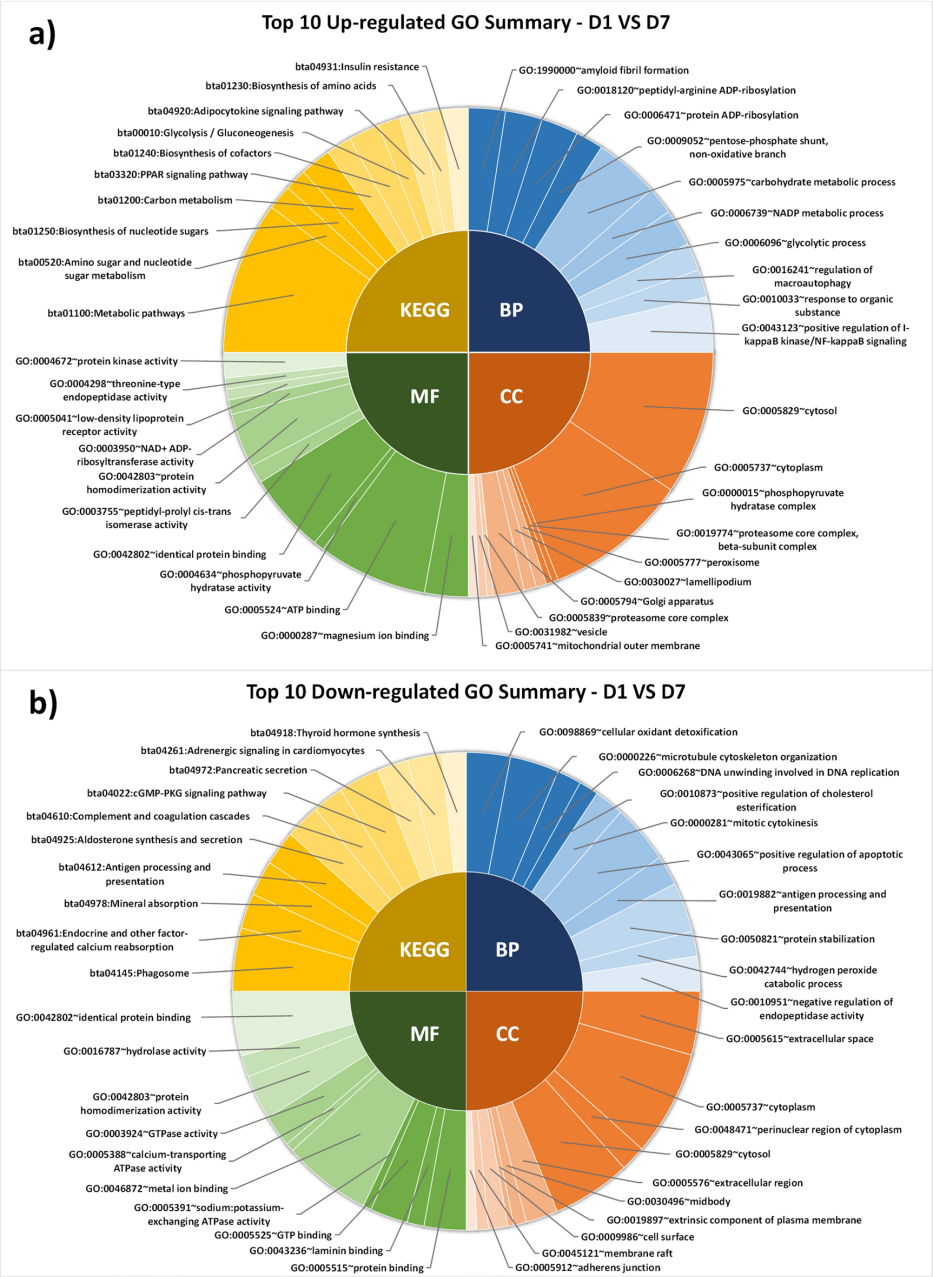
Top 10 significant (*P* < 0.05) GO and pathway of up-regulated proteins (**a**) and down-regulated proteins (**b**) on D1 vs. D7 (day 1 vs. day 7).

Up-regulated proteins on D1 vs. D7 revealed significant (*P* < 0.05) BPs of interest like pentose-phosphate shunt, carbohydrate metabolism, glycolytic process, and positive regulation of NF-κB signaling; MFs like magnesium ion binding, ATP binding, phosphopyruvate hydratase activity, identical protein binding, and low-density lipoprotein receptor activity; and KEGG pathways highlighted metabolic pathways, metabolism of amino and nucleotide sugars, biosynthesis of nucleotide sugars, cofactors and amino acids, PPAR signaling pathway, and glycolysis/gluconeogenesis (Fig. 5a**)**. Down-regulated proteins were associated with the significant (*P* < 0.05) BPs related to positive regulation of cholesterol esterification and apoptotic process, antigen presentation and processing, and protein stabilization. MFs associated with these proteins were protein binding, sodium: potassium-exchanging ATPase activity, calcium-transporting ATPase activity, metal ion binding, and protein homodimerization activity. KEGG pathways revealed significant (*P* < 0.05) pathways like endocrine and other factor-regulated calcium reabsorption, mineral absorption, antigen processing and presentation, aldosterone synthesis and secretion, complement and coagulation cascades, cyclic guanosine monophosphate-dependent protein kinase (cGMP-PKG) signaling pathway, adrenergic signaling in cardiomyocytes, and thyroid hormone synthesis (Fig. 5b**)**.

**Fig. 6:**
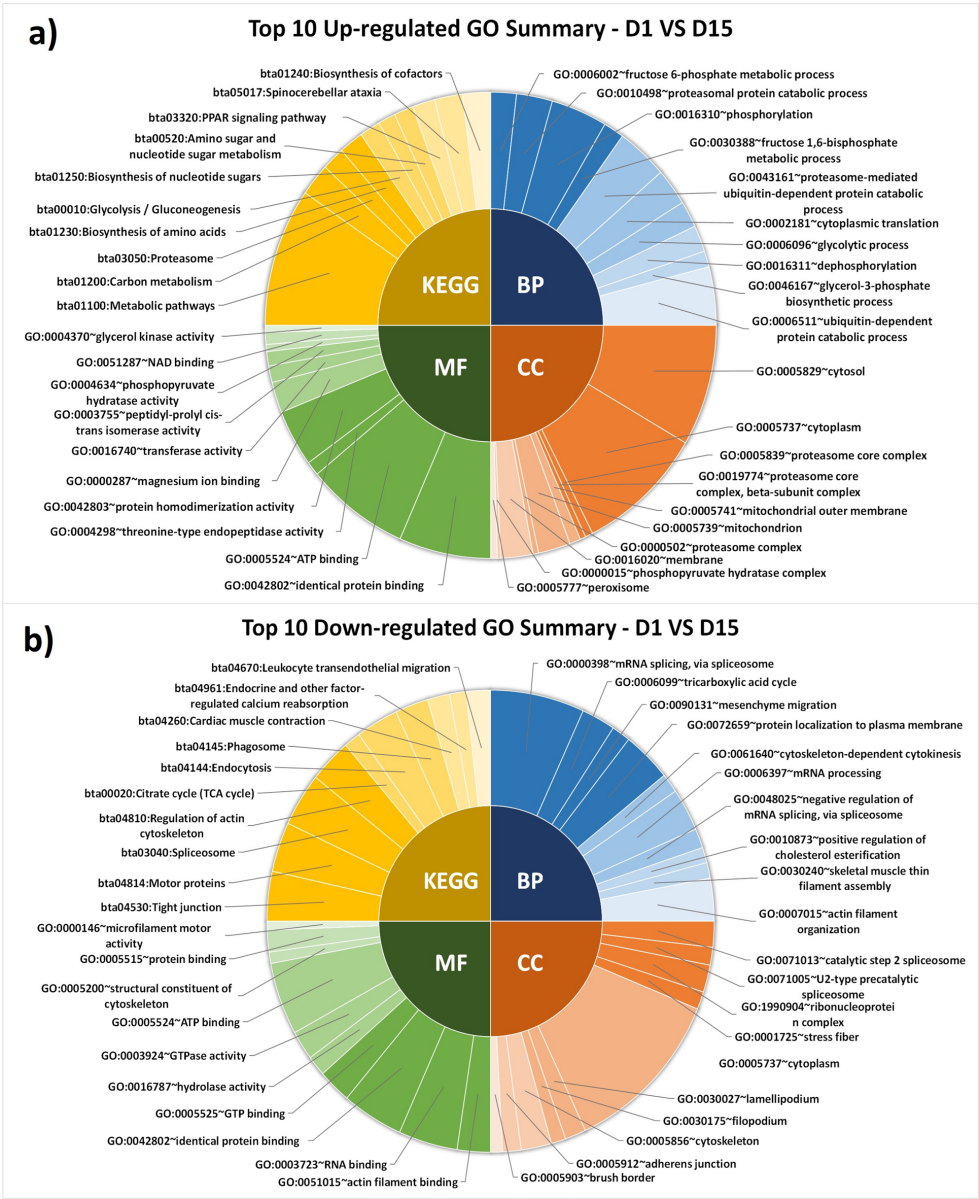
Top 10 significant (*P* < 0.05) GO and pathway of up-regulated proteins (**a**) and down-regulated proteins (**b**) on D1 vs. D15 (day 1 vs. day 15).

The analysis of up-regulated proteins on D1 vs. D15 identified significant (*P* < 0.05) BPs like fructose metabolic process, proteasomal protein catabolic process, phosphorylation, cytoplasmic translation, glycolytic process, glycerol-3-phosphate biosynthesis, and ubiquitin-dependent protein catabolic process. Top significant (*P* < 0.05) MFs associated were identical protein binding, ATP binding, threonine-type endopeptidase activity, protein homodimerization, magnesium ion binding, phosphopyruvate hydratase activity, and glycerol kinase activity. KEGG pathways highlighted their significant (*P* < 0.05) involvement in metabolic pathways, carbon metabolism, the proteasome, biosynthesis of amino acids, nucleotide sugars and cofactors, glycolysis, amino sugar and nucleotide sugar metabolism, and PPAR signaling (Fig. 6a**)**. The down-regulated proteins were involved in significant (*P* < 0.05) BPs like mRNA splicing, TCA cycle, negative regulation of mRNA splicing, mRNA processing, and positive regulation of cholesterol esterification; significant (*P* < 0.05) MFs were RNA, ATP, identical protein, guanosine triphosphate (GTP) binding, and GTPase activity; and KEGG pathways indicated involvement of pathways related to the spliceosome, cardiac muscle contraction, endocrine and other factor-regulated calcium reabsorption, and leukocyte trans-endothelial migration (Fig. 6b**)**.

**Fig. 7:**
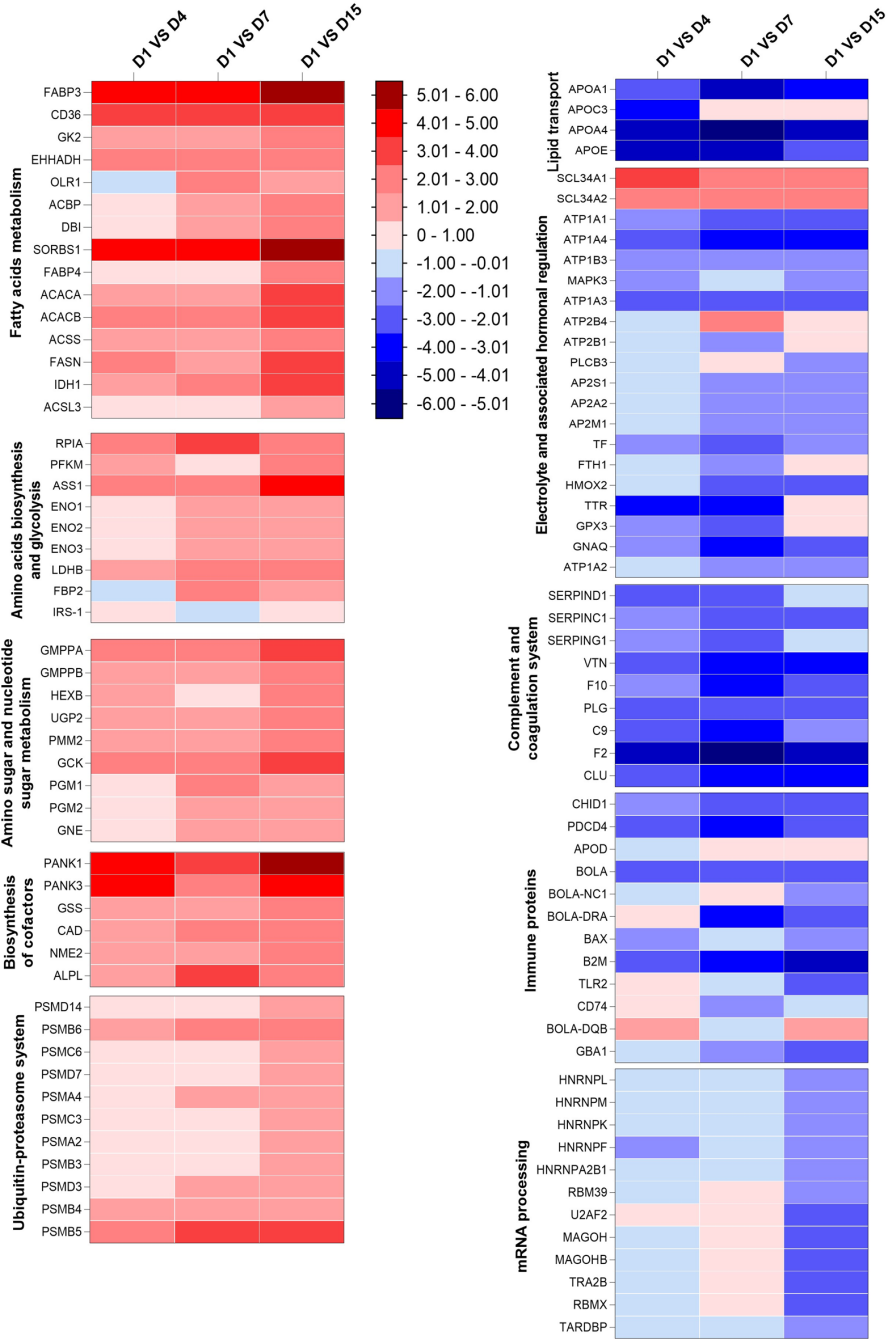
Heatmap representing major pathways and the log_2_FC values of DEPs between different days. Cells shaded in red and blue were the up-regulated proteins and down-regulated proteins, respectively.

The heatmap represents the log_2_FC values of DEPs between different days (Fig. 7**)**. Cells shaded in red and blue were the up-regulated and down-regulated proteins, respectively. The study’s major findings highlighted that the up-regulated proteins were mainly involved in the metabolism of the mammary gland such as fatty acid, amino acids biosynthesis, glycolysis, amino sugar and nucleotide sugar metabolism, biosynthesis of cofactors, and the ubiquitin-proteasome system. The down-regulated proteins were associated with lipids transport, electrolytes and associated hormonal regulation, complement and coagulation, immunity, and mRNA processing.

### Validation of hub proteins/genes through qPCR

**Fig. 8:**
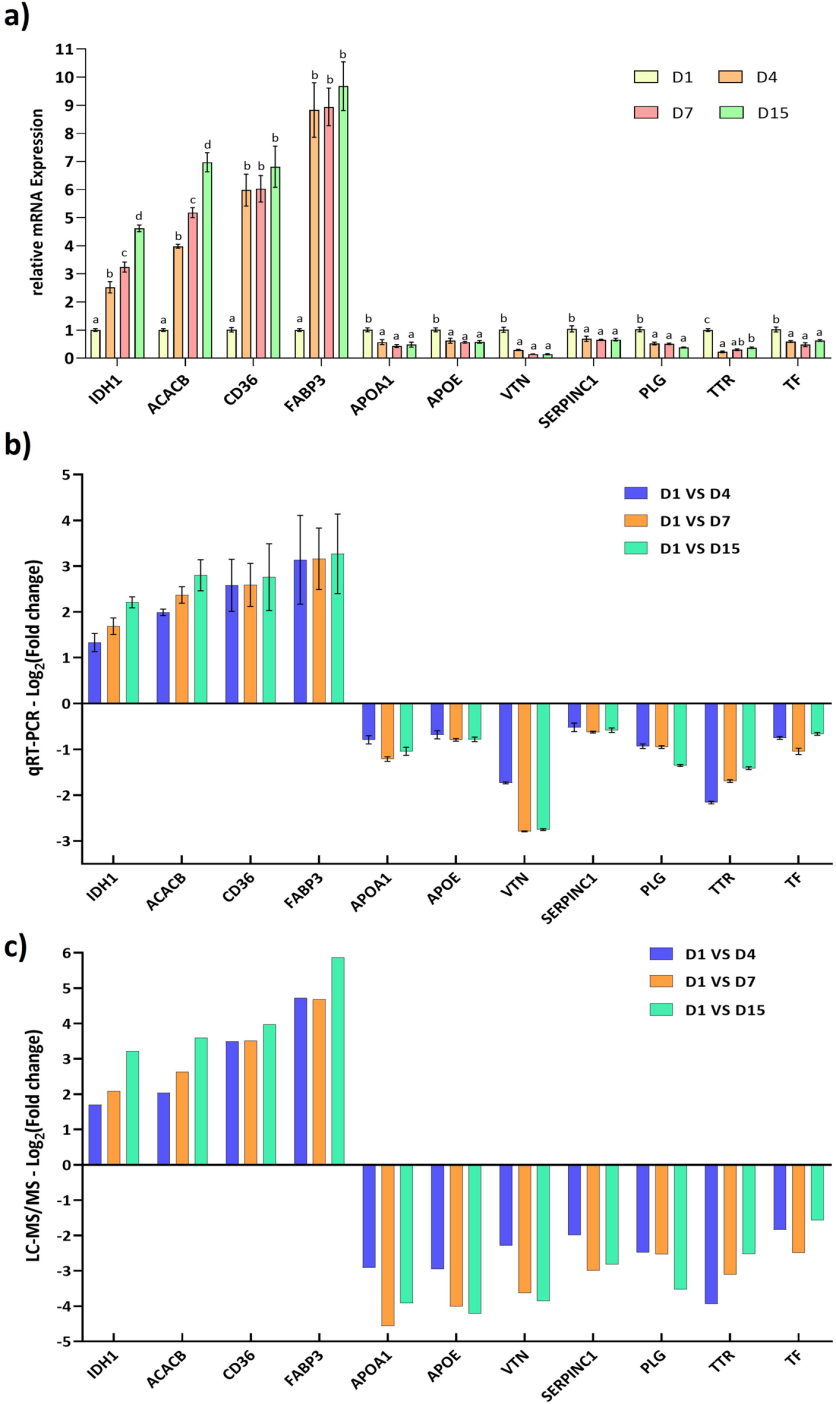
Validation of proteomic (LC-MS/MS) data by qRT-PCR for a few selected genes. Relative mRNA expression of selected from the DEPs (**a**). Fold values of the qRT-PCR analysis of candidate genes are normalized to the housekeeping genes RPS23 and RPS9. Log_2_(Fold change) of the genes by qRT-PCR (**b**), and proteomics or LC-MS/MS (**c**).

The selected hub proteins were validated by calculating their relative mRNA expression. The end products of the PCR were visualized by agarose gel electrophoresis for the target genes, confirming the product size (**Supplementary Fig. S6 online).** The up-regulated hub proteins exhibited significantly (*P* < 0.05) higher mRNA expression. IDH1 and acetyl-CoA carboxylase beta (ACACB) were differentially expressed across all four sampling days. Conversely, all down-regulated hub proteins showed significantly (*P* < 0.05) lower mRNA expression **(**Fig. 8a**).** When comparing the qPCR log_2_(FC) **(**Fig. 8b**)** with the LC-MS/MS proteomic log_2_(FC) **(**Fig. 8c**)**, it was observed that the up-regulated mRNAs exhibited higher values in the LC-MS/MS analysis. In contrast, the down-regulated mRNAs showed a decrease in LC-MS/MS values.

## Discussion

Colostrum and milk composition change significantly during lactation. Studies have shown a decline in SNF, fat, and protein (%) over time, while pH gradually increased from colostrum to mature milk^21^. Colostrum from uninfected glands has a mean SCC of 470,000/mL, decreasing nearly 12-fold within the first 250 h. It also shows increased T-lymphocytes, plasma cell clones, neutrophils, and monocytes, with macrophages as the predominant leukocytes in bovines^22^. Our findings reflect these dynamic changes in milk composition and immune cell profiles during early lactation.

Up-regulated proteins were linked to metabolism, PPAR signaling, and amino sugar, nucleotide sugar, and cofactor biosynthesis, aligning with earlier findings in Sahiwal cow MECs^13^. PPARs regulate transcription, differentiation, carbohydrate, lipid, and amino acid metabolism, inflammation, and tissue repair, while also controlling FA metabolism^23^. Their differential expression highlights the PPAR role in lactation and milk synthesis.

Milk fat is a critical determinant of the organoleptic quality and commercial value of milk. Buffalo milk, in particular, has a higher fat content than cow, goat, and sheep^24^. FA binding proteins (FABP3 and FABP4), cluster of differentiation 36 (CD36), Glycerol kinases (GK and GK2), Enoyl-CoA hydratase, and 3-hydroxyacyl CoA dehydrogenase (EHHADH), Oxidized low-density lipoprotein receptor 1 (OLR1), Acyl-CoA-binding protein (ACBP) or diazepam-binding inhibitor (DBI), and Sorbin and SH3 domain-containing protein 1 (SORBS1) were found to be up-regulated on days 7 and 15 milk somatic cells. These play significant roles in lactation, enhancing FA metabolism and glucose homeostasis^25–28^. We noted proteins involved in FA *de novo* synthesis acetyl-CoA carboxylase (ACACA), acetyl-CoA short-chain family member (ACSS), FA synthase (FASN), and isocitrate dehydrogenase 1 (IDH1), which have been previously reported in dairy cattle^13,30^. ACACA and FASN are essential enzymes in the biosynthesis of short- and medium-chain FAs. Within MECs, acetyl-CoA synthetase 2 (ACSS2) and ACACA biosynthesize the long-chain FAs (LCFAs). FASN facilitates FA chain extension^29^. ACSS2 catalyzes short-chain FA formation^30^. IDH1 catalyzes the oxidative decarboxylation of isocitrate to alpha-ketoglutarate, producing NADPH, essential for mammary FA synthesis^31^. This suggests that MECs shed in colostrum and milk possess the machinery necessary for FA synthesis, ultimately secreting milk fat globules^13^.

In our study, several proteins facilitating LCFAs uptake and transport, including ACBP, FABP3, FA transport proteins (FATP), CD36, and long-chain acyl-coenzyme-A synthetase (ACSL) were identified. FABP3 and CD36 work synergistically in MECs, facilitating FA uptake, and delivering stearoyl coenzyme A to SCD, which releases oleic acid after desaturation^29^. mRNA levels of ACSL3 and FABP3 rise during lactation, indicating their role in transporting endothelial LCFAs for TG synthesis and secretion^30^. The increased expression of these proteins in our study suggests enhanced mammary FA biosynthesis during lactation and its contribution to buffalo milk’s fat content and quality.

Ribose 5-Phosphate Isomerase A (RPIA), IDH1, Phosphofructokinase, Muscle (PFKM), Argininosuccinate Synthetase 1 (ASS1), and Enolases (ENO1-3) identified in this study are key proteins in amino acid biosynthesis which are enriched in M2 macrophages^32^highlighting the metabolic activity of milk macrophages and their significant role in amino acid biosynthesis pathways during lactation. In the pentose phosphate pathway, RPIA converts ribose-5-phosphate to ribulose-5-phosphate, crucial for producing NADPH and ribose-5-phosphate for nucleotide synthesis^33^. PFKM converts fructose-6-phosphate to fructose-1,6-bisphosphate^34^. ASS1 contributes to arginine synthesis^32^a precursor for nitric oxide and polyamines, important for cellular proliferation and differentiation during lactation.

Furthermore, several proteins involved in amino sugar and nucleotide sugar metabolism such as guanosine diphosphate (GDP)-Mannose Pyrophosphorylases (GMPPA and GMPPB), Hexosaminidase Subunit Beta (HEXB), Uridine diphosphate (UDP)-Glucose Pyrophosphorylase 2 (UGP2), Phosphomannomutase 2 (PMM2), Glucokinase (GCK), Phosphoglucomutases (PGM1 and PGM2), and Glucosamine (UDP-N-Acetyl)−2-Epimerase/N-Acetylmannosamine Kinase (GNE), were differentially up-regulated in this study. GMPPA and GMPPB are essential for glycan moieties of glycoproteins and glycolipids in GDP-mannose biosynthesis^35^. HEXB is a key regulator for promoting glycolysis^36^. UGP2 catalyzes the formation of UDP-glucose which is ultimately converted to lactose and determines milk volume^37^. During lactation initiation, increased PGMs and UGP2 transcription regulate lactose synthesis^38^. PMM2 converts mannose-6-phosphate to mannose-1-phosphate, GCK phosphorylates glucose to glucose-6-phosphate, and PGMs interconvert glucose-1-phosphate and glucose-6-phosphate, ensuring a continuous glucose-6-phosphate supply for lactose synthesis. GNE is responsible for the sialylation of glycoproteins and glycolipids, affecting stability, activity, and half-life of milk glycoproteins that influence immune properties and milk quality^39^.

On days 7 and 15, UGP2, Pantothenate Kinases (PANK1 and PANK3), Glutathione Synthetase (GSS), carbamoyl-phosphate synthetase 2 (CAD), PMM2, GMPPA, Nucleoside Diphosphate Kinase 2 (NME2), and Alkaline Phosphatase (ALPL) were up-regulated and involved in biosynthesis of cofactors. PANK enzymes catalyze the coenzyme A (CoA) biosynthesis^40^. GSS synthesizes glutathione, protecting cells from oxidative stress. CAD drives pyrimidine nucleotide synthesis, crucial for DNA, RNA, and cell proliferation^41^. NME2 maintains nucleoside triphosphate and diphosphate balance for energy transfer and signaling^42^. ALPL regulates phosphate metabolism, dephosphorylation, and mineralization^43^.

Glycolysis is essential in metabolically active MECs, supplying key precursors for lactose, triacylglycerol, and fatty acid synthesis while generating ATP for milk production. Upregulated glycolytic proteins on days 7 and 15 included lactate dehydrogenase-B (LDHB), ENO1-3, Fructose-bisphosphatase 2 (FBP2), GCK, and PGM1. Enolases catalyze the conversion of 2-phosphoglycerate into phosphoenolpyruvate and regulate gene expression, signaling, and immune responses^44^. FBP2 supports high milk production by converting fructose-1,6-bisphosphate to fructose-6-phosphate^45^while GCK activates glycogen and lipid synthesis enzymes like Insulin receptor substrate-1 (IRS1) and FASN^46^. The presence of these proteins confirms the metabolic activity of MECs, ensuring energy supply for milk synthesis and secretion^13^.

The metabolic roles of upregulated hub proteins on D1 vs. D4 (ACSS2, ACACA, FASN, EHHADH, FABP3, CD36, GCK) and D1 vs. D7 (ENO1-3, FBP2, RPIA, PGM1, IDH1) were discussed above. However, on D1 vs. D15, upregulated hub proteins were linked to the proteasome pathway, a key part of the ubiquitin-proteasome system that regulates protein synthesis and degradation by eliminating misfolded or damaged proteins. The 26 S proteasome consists of the 20 S core (α [PSMA1-7] and β subunits [PSMB1-7], including β1, β2, and β5) and the 19 S regulatory complex (Rpt [PSMC1-6] and Rpn [PSMD1-13] subunits)^47^. In cows, 20 S proteasome activity is higher in early lactation, reflecting protein mobilization from body reserves antepartum^48^. The 19 S complex processes ubiquitin-tagged substrates, facilitating their degradation in the 20 S core. Increased oxidative stress elevates 20 S proteasome levels, aiding in protein protection by degrading disordered proteins and inhibiting excessive translation. Thus, 26 S proteasome upregulation supports proper milk protein synthesis, MEC proliferation, and stress management, ensuring mammary gland health and functionality^49^.

Apolipoproteins (APOs) such as APOA1, APOC3, APOA4, and APOE play a critical role in lipid transport and cholesterol homeostasis by mediating phospholipid efflux and TG homeostasis were down-regulated on D4. Cholesterol is crucial for synthesizing vitamin D and steroid hormones vital for newborn development. APOE was significantly decreased over lactation, with high levels of D1 highlighting cholesterol’s importance in neonatal development^16^. APOA4 facilitates chylomicron formation and dietary FA transportation from the intestine to circulation. APOA1, a key high-density lipoprotein component, facilitates reverse cholesterol transport. APOC3 primarily contributes to very-low-density lipoprotein production, potentially reducing intracellular TG accumulation^50^. The decline in APO abundance from colostrum to mature milk likely reflects the transition from blood-borne lipid dependence to *de novo* FA synthesis by lactocytes as a source of milk fat. This is also reflected in the present study, as fat % decreased from D1 to D15.

On D4, solute carrier family 34 alpha proteins (SLC34A1 and SLC34A2) associated with sodium: phosphate transport, ATPase Na+/K + Transporting-alpha (ATP1A1 and ATP1A4), -beta (ATP1B3) proteins, and Mitogen-activated protein kinase-3 (MAPK3) associated with aldosterone-regulated sodium absorption were up-regulated. On D7, ATP1A1-ATP1A4, ATP2B3, Plasma Membrane Calcium-Transporting ATPases ATP2B4 and ATP2B1 were involved in significant pathways such as endocrine and other factor-regulated calcium reabsorption, mineral absorption, aldosterone synthesis and secretion, cGMP-PKG signaling pathway, pancreatic secretion, and adrenergic signaling in cardiomyocytes. Several other proteins distinguished these pathways. The SLC34 family proteins regulate phosphate concentration via intestinal absorption and renal excretion. They are expressed in the proximal tubules of kidneys (SLC34A1), small intestines, and various lumen fluids (milk, saliva, pancreatic fluid, and lung surfactant) (SLC34A2) and detected only in lactating mammary glands of goats, making it a potential marker for mammary secretory activity^51^. ATP1A1-4 and ATP1B3 regulate ion gradients for muscle, nerve function, and growth, with higher levels reported in Murrah colostrum^15^.

ATP2B4 and ATP2B1 transport calcium in MECs, eject calcium from the cytoplasm into milk, and regulate cellular Ca^2+^ homeostasis^52^. On D15, Phosphoinositide phospholipase C beta 3 (PLCB3), Adaptor protein complex 2 subunit sigma (AP2S1), Adaptor-related protein complex 2 subunit alpha (AP2A2), and Adaptor-related protein complex 2 subunit mu (AP2M1) were associated with endocrine and other factor-regulated calcium reabsorption were down-regulated. PLCB3 triggers a rapid release of inositol triphosphate, increasing intracellular calcium levels^53^. AP2S1 is crucial for cellular calcium-sensing pathways, with mutations causing hypothyroidism^54^. AP2A2 is involved in protein interactions, and AP2M1 is involved in receptor binding and signal recognition^55^. Proteins specific to the mineral absorption pathway, such as Serotransferrin (TF), Ferritin heavy chain (FTH1), and Heme oxygenase 2 (HMOX2), were down-regulated. TF was higher in colostrum, while ferritin, essential for iron storage, was lower than in mature milk indicating distinct roles in hemoglobin formation and iron transfer in each milk type^15^. HMOX2, a key enzyme in heme catabolism, provides free ferrous iron (Fe^2+^), biliverdin, and carbon monoxide, and protects cells against oxidative stress^56^.

In thyroid hormone synthesis pathway, on D4, Transthyretin (TTR), Glutathione Peroxidase 3 (GPX3), ATP1A4, ATP1B3, and ATP1A1, and on D1 vs. D7, G Protein Subunit Alpha Q (GNAQ), ATP1A3, and ATP1A2 were down-regulated. Thyroid hormones regulate cellular differentiation and metabolism, and are galactopoietic in mammary tissue. They stimulate the synthesis of casein induced by prolactin and the development of MECs in the transition from pregnancy to lactation^57^. TTR transports thyroid hormones from the bloodstream to the different organs and is higher in colostral cells than in transitional and mature milk cells^15^. GPX3 interferes with thyroid hormone synthesis through its antioxidant function^58^. GNAQ is essential for thyroid gland growth and showed greater down-regulation as lactation proceeds^59^. In the present study, we obtained an increased expression of thyroid hormone-related proteins in colostrum, highlighting their importance in mammary gland growth and functionality.

Colostrum contains a complex array of bioactive proteins that play a crucial role in protecting neonates against pathogens and environmental challenges encountered during the early postpartum period. The complement system, part of innate immunity, regulates coagulation-fibrinolysis and supports innate immunity in newborn calves^17^. Milk complement components modify gut microbiota and lyse bacteria, with low abundance in colostrum and transition milk, participating in the plasminogen-activating cascade^2^. As part of the membrane attack complex (MAC), they eliminate microorganisms and apoptotic cells, with fluctuating abundance from colostrum to mature milk and throughout lactation^60^. In our study, SERPINs (D1, C1, G1), vitronectin (VTN), coagulation factors (F2, F10), plasminogen (PLG), complement component 9 (C9), and clusterin (CLU) were downregulated on D4 and D7. The expression of SERPINs during lactation suggests that proteolytic machinery is needed for producing nutritionally functional proteins with stabilized structures and ensuring passive immunity transfer by inhibiting proteolysis^13^. In the calves’ GI tracts, they ensure passive immunity transfer by inhibiting proteolysis of immunoglobulin and other proteins^17^. SERPIND1 inhibits thrombin activity via heparin interaction, promotes leukocyte chemotactic factors release, and induces angiogenesis^61^. SERPINC1 inhibits proteases by interacting with endothelial heparin-like substances, and procoagulant factors like thrombin and factors IXa, Xa, XIa, XIIa, and VIIa, and accounts for 60–70% of the body’s antithrombin activity. It also has strong anti-inflammatory effects. In dairy goats, it’s overexpression increased milk fat and decreased lactose, urea, nitrogen, and somatic cells^62^. VTN prevents MAC-mediated host cell lysis and aids wound healing^17^. F2 converts fibrinogen to fibrin, forms a clot crucial for host defense and intravascular immunity, activates complement components C3 and C5, and promotes the complement cascade. C9 forms the multi-protein complex, creating pores in target pathogen membranes^63^. PLG, in its active form plasmin, participates in fibrinolysis and provides proteolytic activity against bacterial cells^64^. CLU decreased in 9 day’s post-partum bovine milk^16^and is involved in MECs differentiation and mammary gland development. It interacts with lipids, complement components, amyloid-forming proteins, and immunoglobulins, regulating complement activity, cell interactions, and cell survival^65^.

Immune-related proteins showed large changes over postpartum. The high concentration of immune-related proteins aligns with findings in bovine colostrum studies^16^maybe reflecting their role in the maturation of calves’ immune systems. Postnatal development of the mammalian mucosal immune system is crucial for responding to rapid colonization by commensal bacteria and potential pathogens^66^. Some maternal cells enter neonatal circulation, peaking 24 h after birth. Colostrum containing maternal leukocytes aids faster development of antigen-presenting cells (APCs), essential for acquired immune response^67^. On comparison between D1 and D4, proteins involved in the negative regulation of cytokine production in the inflammatory response, such as chitinase domain-containing protein 1 precursor (CHID1), programmed cell death protein 4 (PDCD4), APOD, and F2, and proteins involved in other immune systems functions like Bovine Lymphocyte Antigen (BOLA), non-classical Major Histocompatibility Complex (MHC) class I antigen (BOLA-NC1), alpha chain of the MHC class II DR (BOLA-DRA), Bcl-2-associated X protein (BAX), Beta-2 microglobulin (B2M), mitogen-activated protein kinase 3 (MAPK3), and Toll-like receptor (TLR)−2 were down-regulated. On D7, proteins involved in antigen processing and presentation, such as Cluster of Differentiation 74 (CD74), BOLA-NC1, Bovine lymphocyte antigen DQ Beta (BOLA-DQB), Glucosylceramidase Beta 1 (GBA1), BOLA-DRA, and BOLA were down-regulated.

The down-regulation of immune-related proteins and up-regulation of proteins associated with metabolism in mature milk may be essential for maintaining lactational consistency and supporting the metabolic demands of sustained milk production. This transition also reflects the functional adaptation of the mammary gland from an immune-protective to a nutritive role. CHID1 breaks down chitin, helping newborns combat pathogenic fungi colonization during initial^68^. PDCD4 promotes apoptosis in MECs via the ERK/Bcl-2/Bax pathway^69^modulates oxidative stress by releasing cytochrome c and inflammation by binding to arachidonic acid (ARA), and inhibits the synthesis of pro-inflammatory prostaglandins. APOD regulates the ARA metabolism and has antioxidant and anti-inflammatory potential^70^. MHC system proteins (BOLA and BOLA-DRA) present foreign antigens to T-cells, initiating the immune response. BOLA-NC1 modulates immunity by interacting with inhibitory receptors on natural killer cells, T lymphocytes, and APCs^21^. High B2M levels reflect active IgG transport into the colostrum^71^. TLRs and CD74, expressed in professional APCs, activate pro-inflammatory cytokine production, triggering the NF-κB/MAPK pathways and innate immune response. Down-regulation of this pathway leads to reduced oxidative stress and apoptosis in MECs^72,73^. As the mammary gland repeatedly undergoes apoptosis and growth cycles during pregnancy, parturition, lactation, and involution, the apoptosis and immune proteins play important roles during all these phases to maintain the functionality of the mammary gland^74^.

Similar to the up-regulated hub proteins, the down-regulated hub proteins on D4 and D7 (PLG, VTN, SERPINC1, APOA1, TTR, APOE, TF, and APOE) were involved in different functions, and are discussed above. However, the down-regulated hub proteins on D15 were only associated mRNA processing and transport. Heterogeneous Nuclear Ribonucleoproteins (HNRNPs) such as HNRNPL, HNRNPK, HNRNPF, HNRNPA2B1, HNRNPH1, and HNRNPM^75^splicing factors, including RNA Binding Motif Protein 39 (RBM39), U2 Small Nuclear RNA Auxiliary Factor 2 (U2AF2), and Transformer 2 Beta Homolog (TRA2B) proteins bind to RNA and influence pre-mRNA processing, ensuring proper splicing and maturation of mRNAs, facilitate spliceosome assembly function and remove intron from pre-mRNA^76^. RNA Binding Proteins like RNA Binding Motif Protein X-linked (RBMX) and TAR DNA Binding Protein (TARDBP) play significant roles in post-transcriptional regulation^77^which is crucial for synthesizing necessary proteins for lactation and colostrum’s immune functions. Hence, in our study, by visualizing the hub proteins we noted the proteins that regulate gene expression, mRNA stability, and translation, which are crucial for producing high-quality colostrum.

To summarize our results, the up-regulated somatic cell proteins identified from colostrum to the mature milk stage were involved in metabolic processes due to the mammary gland’s increased milk synthesis and secretion activity. Following proteins such as FABP3, CD36, ACACA, and ACACB associated with lipid biosynthesis, RPIA, PFKM, ASS1, ENO1-3, and LDHB involved in amino acid biosynthesis and glycolysis, GMPPA-B, PMM2, PGM1-2, and GNE associated with amino- and nucleotide-sugar metabolism, PANK1-2, and ALPL in cofactors’ biosynthesis and PSMBs and PSMDs involved in the protein turnover were found upregulated in the mature milk. The down-regulated and highly connected proteins were involved in other important functions during the transition of colostrum to milk. Among them proteins associated with lipid transport (APOA1, APOA4, and APOE), regulation of electrolytes, minerals, and associated hormones (SLC34A1-2, ATP1A1-4, AP2S1, AP2A2, AP2M1, TF, TTR, GPX3, and GNAQ), complement and coagulation systems (SERPINC1, SERPIND1, SERPING1, F2, F10, VTN, and CLU), immune system (CHID1, PDCD4, BOLA, BOLA-NC1, BOLA-DRA, BAX, and B2M) and mRNA processing (HNRNPs, RBMs, TARDBP, and U2AF2). This alteration is essential for the initiation and maintenance of lactation needed for the growth and development of the suckling newborn calves. In the current study, most of these DEPs were also identified among the top 10 hub proteins across various combinations, highlighting their potential significance in lactation mechanisms. The data from this study provides a reference map of somatic cell proteomic changes in buffalo colostrum and milk during early lactation.

We underscore the novelty of this study by noting that it is the first to provide a comprehensive characterization of changes occurring in the colostrum and milk somatic cell proteome during the initial lactation stages in Murrah buffaloes, revealing coordinated shifts in immune protection and metabolic adaptation. Additionally, we validated several key hub proteins by assessing their relative mRNA expression levels. We also acknowledge research gaps, including the need for functional validation of candidate proteins and exploration of post-translational modifications influencing their activity throughout lactation.

In conclusion, buffalo somatic cells are enriched with proteins involved not only in immune function but also in metabolism, biosynthesis, regulation of minerals, and mRNA processing required for milk component synthesis. This study provides new insights into the proteomic remodeling of milk somatic cells during the colostrum-to-mature milk transition, deepening our understanding of buffalo lactation. The identified proteins may serve as biomarkers for early monitoring lactation performance (mammary gland health and milk quality), improving breeding strategies, and enhancing neonatal health through better colostrum quality.

## Materials and methods

### Colostrum and milk sampling

 Six late pregnant Murrah buffaloes (*Bubalus bubalis*) were selected from the dairy herd of ICAR-National Dairy Research Institute, Karnal, India. These buffaloes had been dried off 5 to 7 weeks before calving. Selected buffaloes were multiparous (parity 3 to 5), high yielders (> 10 kg/day) during peak lactation of previous lactation. Composite colostrum, early transitional, late transitional, and mature milk samples were collected from buffaloes (*n* = 6 each) on postpartum D1, D4, D7, and D15, respectively. All foremilk strippings were screened using the California mastitis test (CMT) for the udder inflammation scoring. The samples were collected aseptically in a sterile centrifuge tube of 50 mL, and transported immediately (collected within one hour) to the laboratory on ice for the composition and milk somatic cell counts (SCCs) using a Lactoscan milk analyzer (Milkotronic Ltd., Stara Zagora Bulgaria). Milk DLC was performed microscopically using the methylene blue staining dye.

### Isolation of somatic cells for proteomic analysis

 The collected colostrum and milk samples were filtered through a nylon filter (40 μm) into 50 mL siliconized tubes, then centrifuged at 3000 rpm for 15 min at 4 °C. The fat layer was removed and the supernatant was discarded. The cell pellet was washed twice by dissolving in 1X Dulbecco’s Phosphate Buffered Saline (DPBS, pH 7.2) and centrifuged at 3000 rpm for 8 min at 4 °C. The supernatant was discarded and the cell pellet obtained was layered over the 30% percoll for gradient centrifugation at 3000 rpm for 30 min to get a purified population of milk somatic cells. The upper layer was collected, dissolved, and washed twice in DPBS. After the last wash, somatic cell viability (%) was evaluated using the trypan blue method. The cells obtained were finally suspended in 1 mL 1X DPBS in 2 mL autoclaved Eppendorf tubes and then centrifuged at 4 °C, 10,000 rpm for 5 min. The obtained cell pellet was lysed with 5% SDS + 0.1 M Tris buffer in a 1:1 ratio to minimize proteolysis and kept at −80 °C until further analysis. Six biological samples and three technical replicates were examined throughout the study similar to Janjanam et al.^13^.

### In-solution digestion and peptide fractionation

 The total protein concentration quantification was done using Thermo Nanodrop ONE. 50 µg of the total protein was taken from individual samples, reduced by using 5 mM tris 2-carboxyethyl phosphine, and alkylated with 50 mM iodoacetamide for 20 min at 37 °C. Then, samples were digested with trypsin (1:50 trypsin/lysate ratio) for 16 h at 37 °C, and a C18 silica cartridge column was used for desalting. The obtained peptides were dried using the vacuum evaporator and dissolved in loading buffer A (2% acetonitrile, 0.1% formic acid, v/v) for LC-MS/MS analysis.

### LC-MS/MS

The experiments were performed on an EASY-nLC-1000 system (Thermo Fisher Scientific) coupled with a Thermo Scientific-Orbitrap Exploris mass spectrometer. 1.0 µg of peptide mixture were loaded to the analytical column (PepMap™ RSLC C18, 75 µm × 15 cm Acclaim PepMap (Thermo Fisher Scientific)) and separated with a 0–40% gradient of buffer B (80% acetonitrile, 0.1% formic acid) at a flow rate of 300 nL/min and injected for Mass spectra (MS) analysis. Eluted the gradients for 60 minutes. MS were acquired in the Orbitrap (Max IT = 25 ms, AGQ target = 300%; RF Lens = 70%; R-60K, mass range (m/z)−375-1500). Dynamic exclusion was employed for 30 s excluding all charge states for a given precursor. MS2 spectra were collected for the top 12 peptides (Max IT = 22 ms, R = 15 K, AGC target 200%). Raw data files were analyzed using Proteome Discoverer software (v. 2.5, Thermo Scientific) with implemented SEQUEST and Amanda algorithm. To identify bovine proteins, raw data was searched against the bovine species from UniProt; the precursor and fragment mass tolerances were set at 10 ppm and 0.02 Da, respectively. The protease used to generate peptides, i.e., enzyme specificity was set for trypsin/P (cleavage at the C terminus of “K/R: unless followed by “P”). Carbamidomethyl on cysteine as fixed modification and oxidation of methionine and N-terminal acetylation were considered as variable modifications for database search. Both peptide spectrum match and protein false discovery rate (FDR) were set to 0.01 to increase the confidence and to avoid false-positive detection. The LC-MS/MS data have been submitted to the ProteomeXchange Consortium via the PRIDE partner repository with the dataset identifier **PXD054309**. Proteins showing one or more than one unique peptide were considered for identification. The 5 corresponding searches against the database were obtained for all proteins from each group with a 5 list of proteins along with its peak area-based quantification values, peptide spectrum match, score, coverage, number of unique peptides, number of peptides, peptide sequence matches, molecular weight, and the calculated isoelectric point (pl).

### **Identification of DEPs**,** heat map**,** and PCA**

 The protein’s abundances were further subjected to statistical analysis. Abundance matrices were filtered based on valid quantification values. The filtered protein abundances were log_2_ transformed followed by missing values imputation using normal distribution. Student’s t-test was applied and significance was calculated with P-values using R-studio environment. All the proteins with P_adj_-values < 0.05 were filtered and regarded as significant. The statistically substantial proteins were used for data visualization. The log_2_ abundances were z-score transformed and visualized using a heatmap and the PCA.

### Bioinformatics analysis

 A one-way Analysis of Variance (ANOVA) was applied to compare means across four days of lactation (D1, D4, D7, and D15), and the Student’s t-test was applied to compare means between two groups (D1 vs. D4, D1 vs. D7 and D1 vs. D15), significance was calculated with P_adj_-values < 0.05. Significantly (P_adj_<0.05) changed proteins were the colostrum to mature milk were analyzed for GO annotation [BP, cellular component (CC), MF], and KEGG pathway enrichment using the Database for Annotation, Visualization, and Integrated Discovery (DAVID) (https://david.ncifcrf.gov/tools.jsp) **(Supplementary file S1 online)**. In GO/KEGG enrichment analysis, we used the Benjamini-Hochberg (BH) correction method to control the FDR and reduce false positives, it balances sensitivity and specificity without being overly conservative. The PPI network maps were constructed using STRING (https://string-db.org/) and visualized using Cytoscape open-source software (version 3.10.1). In the STRING database, PPIs are scored by confidence levels, with medium confidence (≥ 0.4) to capture reliable interactions while maintaining network completeness.

### Validation by Real Time qPCR

 qPCR was performed to validate protein FC levels obtained from LC-MS/MS, using a subset of samples for the LC-MS/MS. Two technical replicates were done on each biological replicate of buffalo colostrum and milk somatic cells. Total RNA was extracted from the isolated cells using the Trizol method. The purity of the isolated RNA samples had the OD_260_/OD_280_ ranging from 1.8 to 2.0. cDNA was synthesized using RevertAidTM First-strand cDNA synthesis kit (Thermo Fisher Scientific, USA) and stored @ stored at −20 °C for downstream processes. 11 different genes (IDH1, ACACB, CD36, FABP3, APOA1, APOE, VTN, SERPINC1, PLG, TTR, and TF) were selected for validation based on their biological relevance, significance to cover the full range of differential expression and involvement in hub genes/proteins network. The primers for the selected genes were designed using the NCBI primer blast primer designing tool shown in **Supplementary Table ****S1**** online.** The fragment size of PCR amplified products was confirmed by agarose gel electrophoresis on 1.5% Agarose gel against 100 bp (bp) DNA ladder (Thermo Fisher Scientific, USA). The PCR assay was performed in 10 µL reaction volume containing Maxima SYBR Green/ROX qPCR Master Mix (2X) (Thermo Fisher Scientific, USA), 0.2 µM of each primer, and 1 µL of cDNA. Mean cycle threshold (Ct) values of the genes were normalized to the geometric mean of either of the two housekeeping genes (RPS23 and RPS9), and mRNA abundance of D1 was taken as a calibrator for all the genes to calculate the relative expression of different time points. The relative expression level of all genes was calculated by the **2**^**−∆∆CT**^ method^78^.

### Statistical analysis

 The SCC, milk composition, DLC, cell viability (%), and relative mRNA FCs were subjected to one-way ANOVA statistical analysis between the intervals at a 95% confidence level (*p* < 0.05) to compare means across four days of lactation (D1, D4, D7, and D15) using SPSS 20.0 software (SPSS, Chicago, IL, USA). The values have been expressed as mean ± standard error mean (SEM) for the tabular representation. Log_2_ transformation was used to normalize the relative mRNA expression data for comparison with the proteome data. To ensure valid use of these tests, normality of data is assessed by the Shapiro-Wilk test, which evaluates whether a dataset follows a normal distribution.

## Electronic supplementary material

Below is the link to the electronic supplementary material.

Supplementary Material 1

## Data Availability

The datasets analyzed during the current study are available in the PRIDE repository [Website: http://www.ebi.ac.uk/pride and Project accession: PXD054309].
