# Supplementary material for: Proteomic analysis of Buffalo milk somatic cells reveals metabolomic and immunological transitions during early lactation
Source: Sci Rep. 2025 Jul 2;15:22704. doi: 10.1038/s41598-025-08433-0 (PMC12216581; doi:10.1038/s41598-025-08433-0)
Supplement: Supplementary file 1 — Supplementary Material 1 [file 41598_2025_8433_MOESM1_ESM.pdf]

Supplementary Fig. S1 online

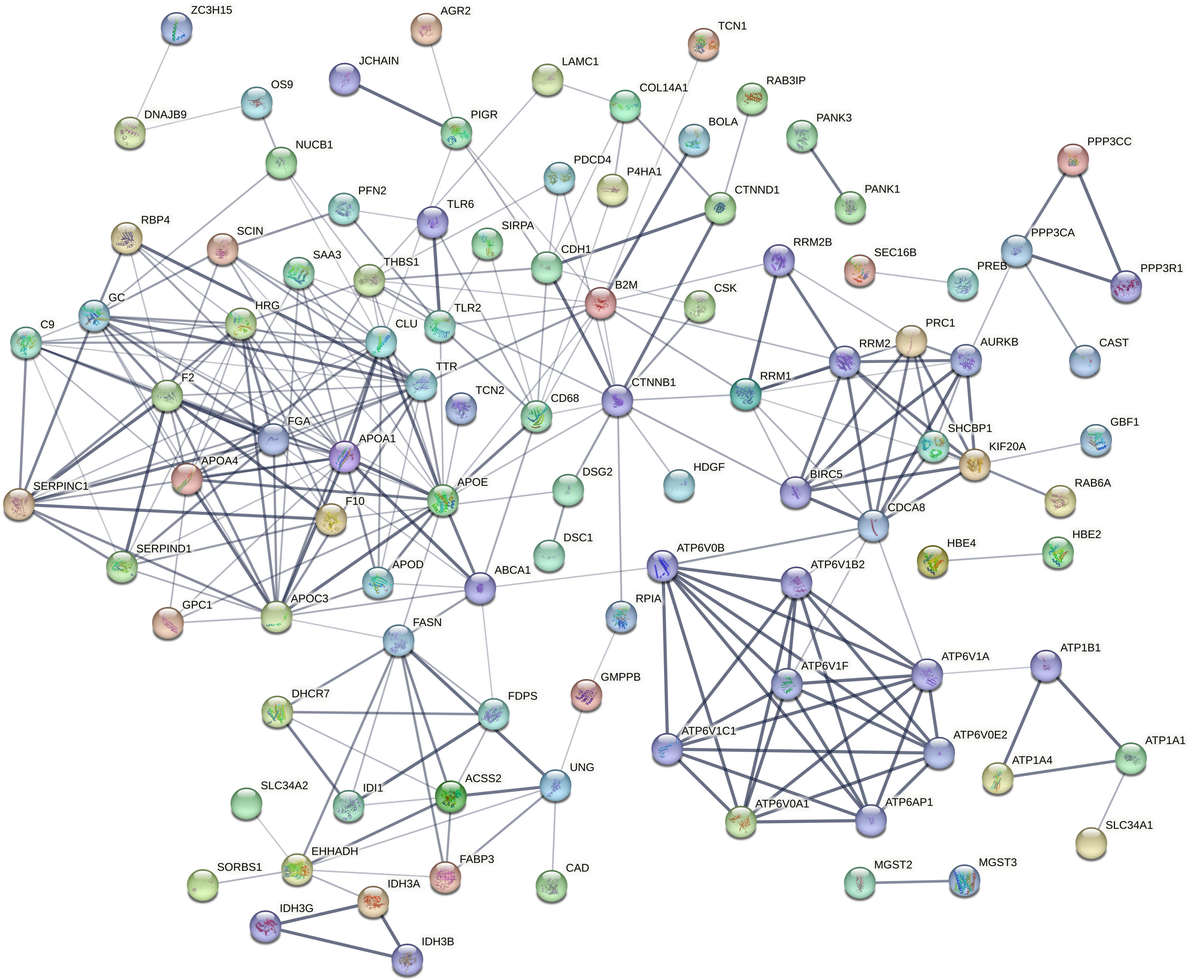

Supplementary Fig. S2 online

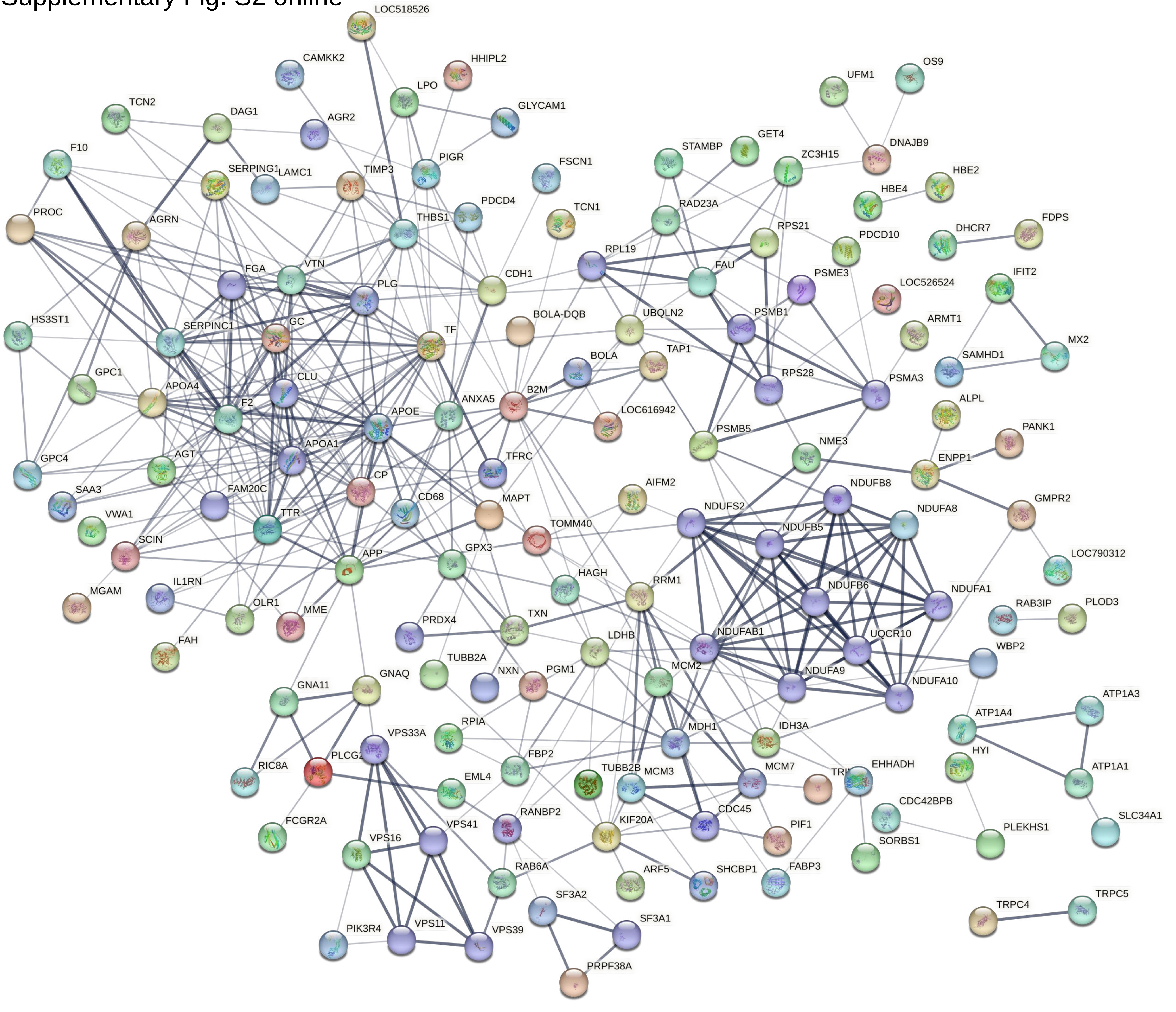

Supplementary Fig. S3 online

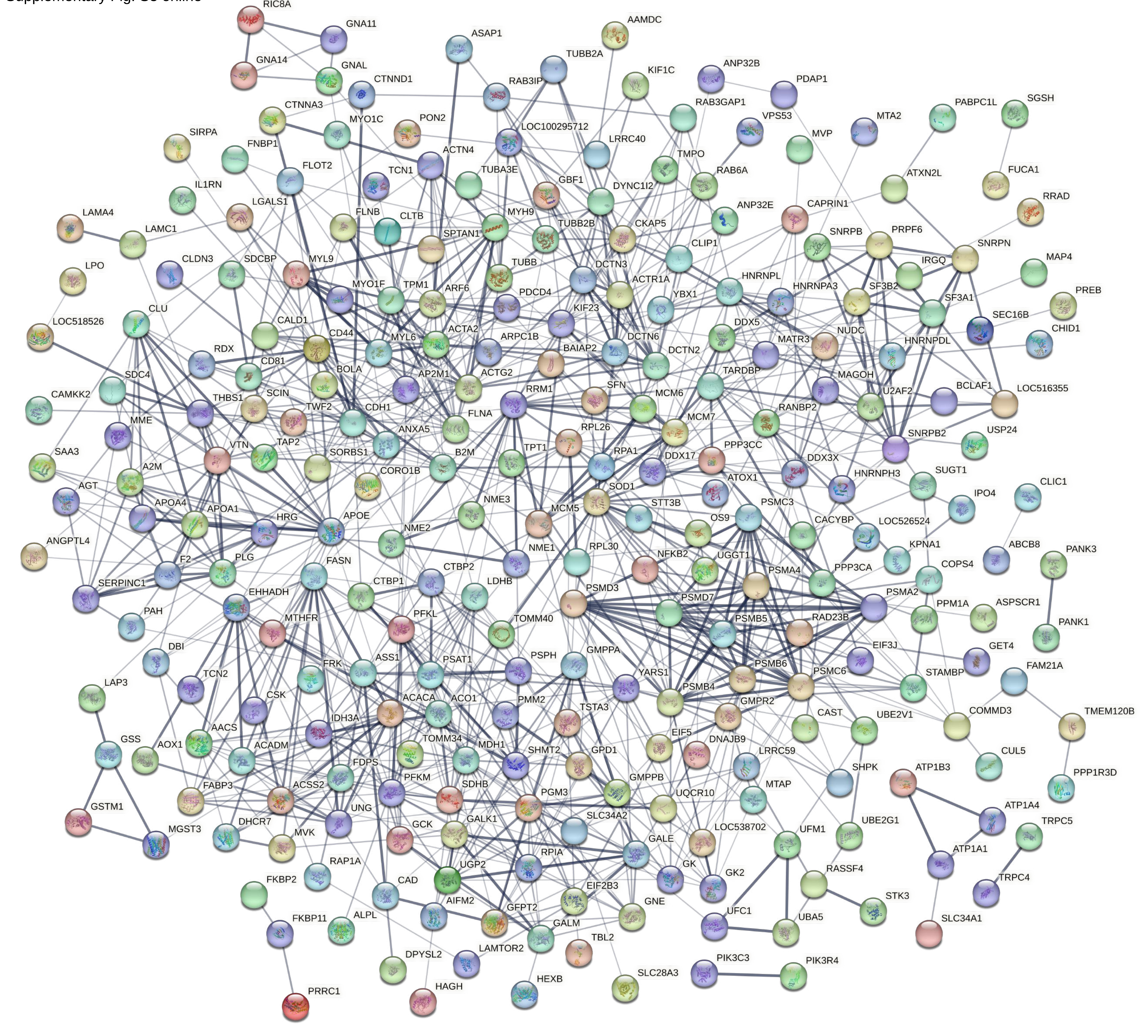

Supplementary Fig. S4 online

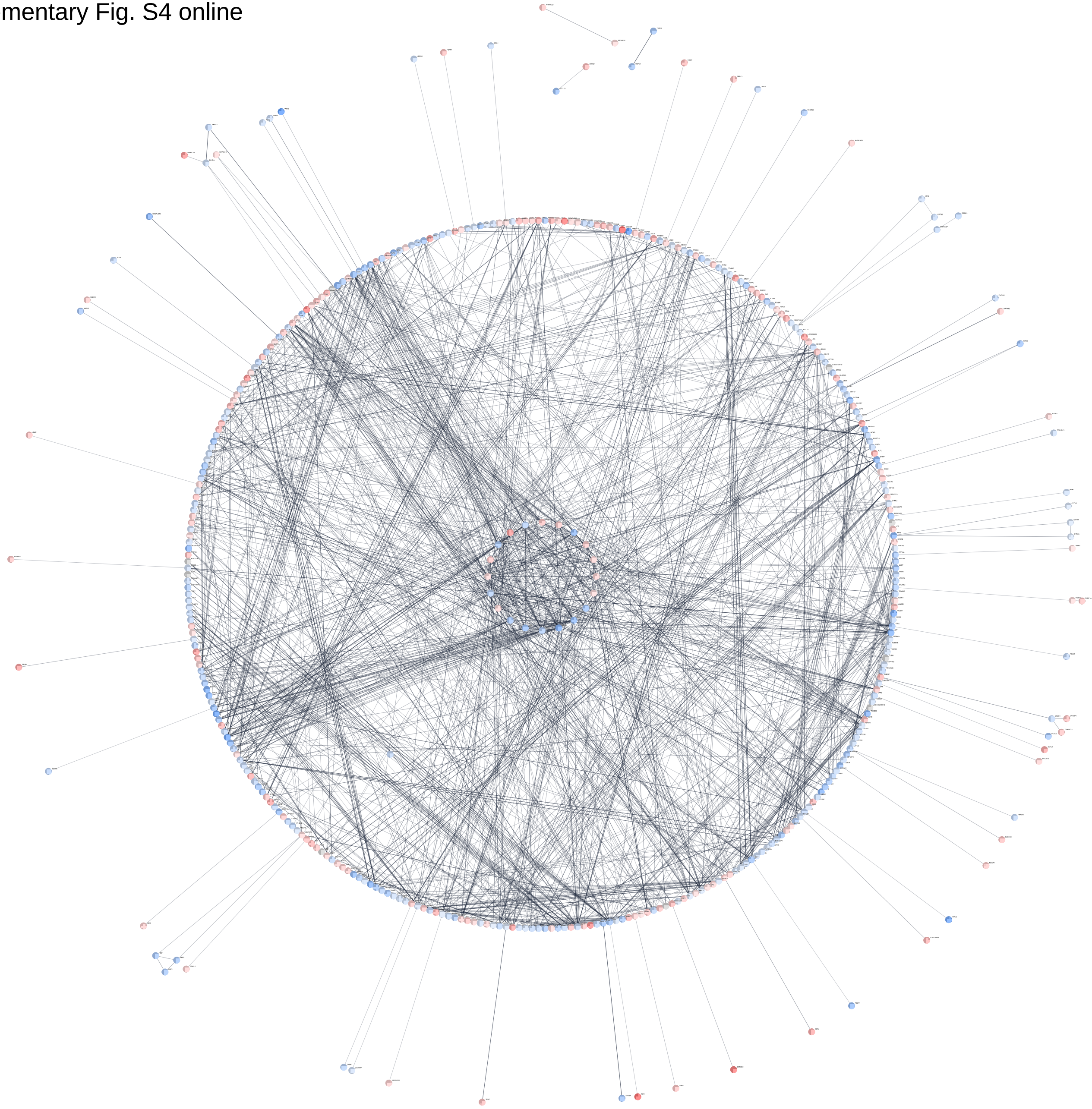

Supplementary Fig. S5 online

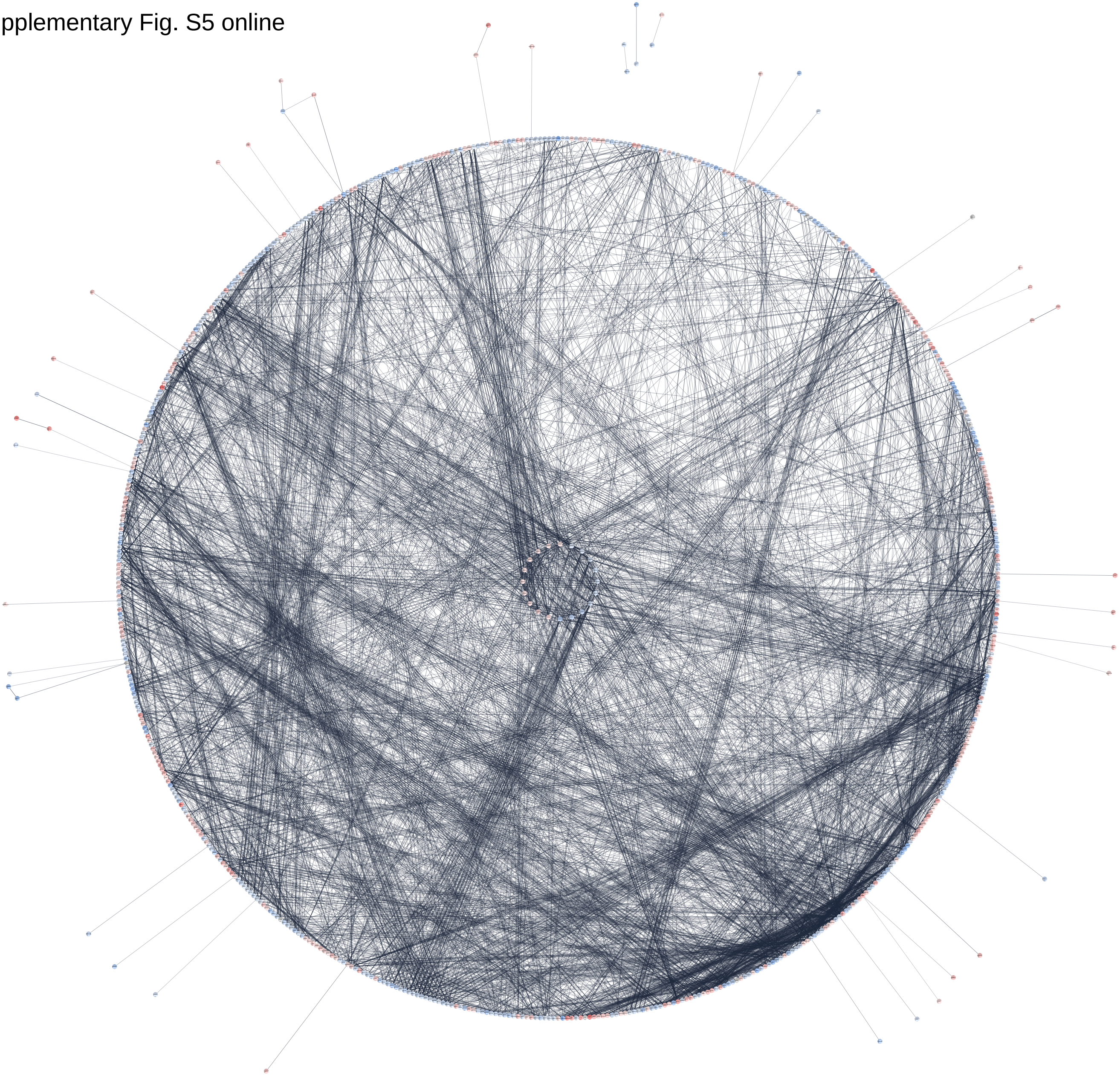

Supplementary Fig. S6 online

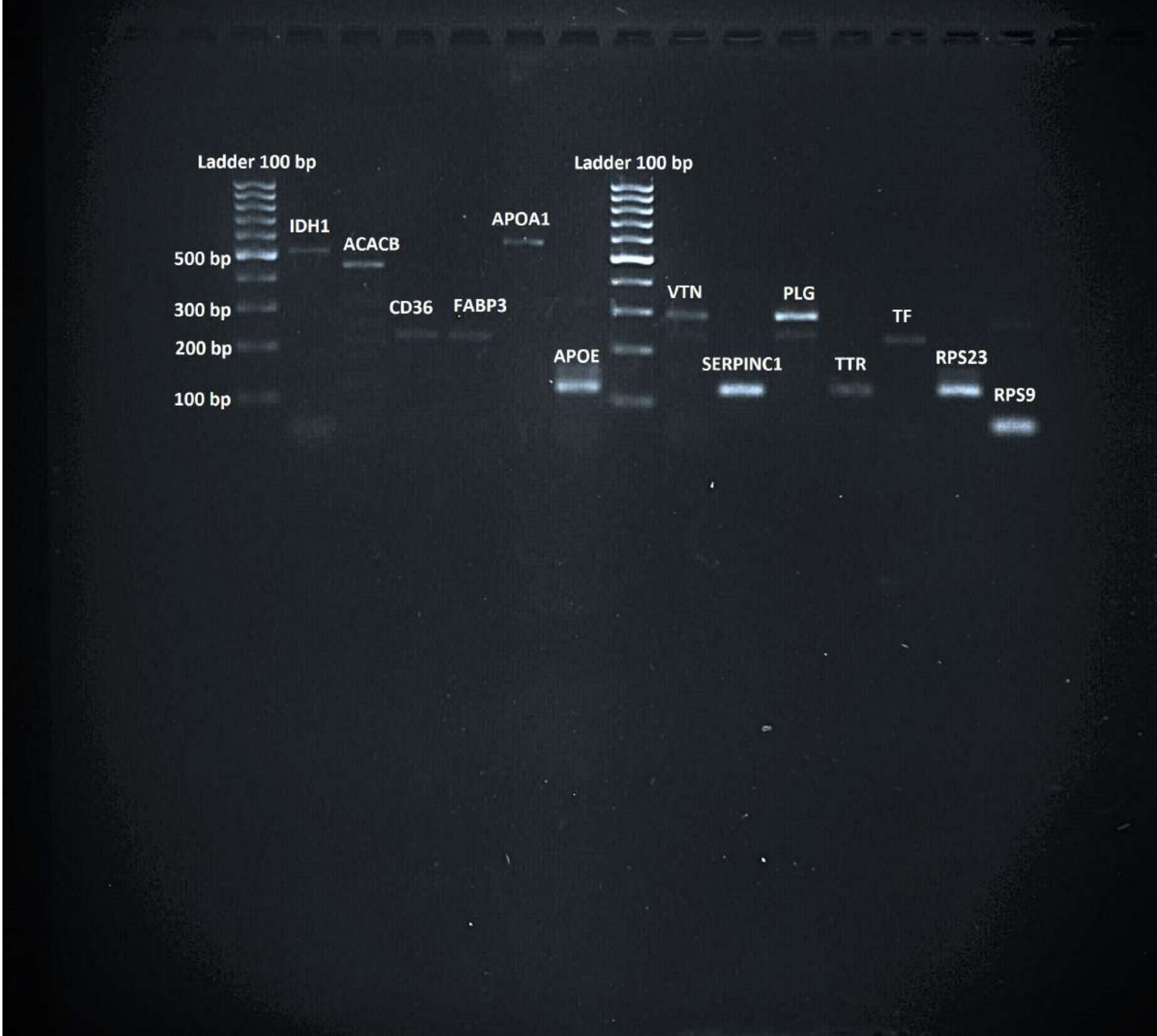

Supplementary Figure legends

Supplementary Fig. S1 online Map showing the relationship of differentially expressed proteins between day 1 and day 4 using STRING in confidence view.

Supplementary Fig. S2 online Map showing the relationship of differentially expressed proteins between day 1 and day 7 using STRING in confidence view.

Supplementary Fig. S3 online Map showing the relationship of differentially expressed proteins between day 1 and day 15 using STRING in confidence view.

Supplementary Fig. S4 online PPI network analysis of the differentially expressed proteins of day 1 vs day 7 (D1 VS D7) in buffalo milk somatic cells. The red nodes represent the upregulated proteins; the blue nodes represent the downregulated proteins ($P<0.05$). Proteins with ≤2 interactions are hidden. The top 10 hub proteins of both up and downregulated proteins are represented at the centre. The darker the color of the protein, the higher the log_2FC.

Supplementary Fig. S5 online PPI network analysis of the differentially expressed proteins of day 1 vs day 15 (D1 VS D15) in buffalo milk somatic cells. The red nodes represent the upregulated proteins; the blue nodes represent the downregulated proteins ($P<0.05$). Proteins with ≤2 interactions are hidden. The top 10 hub proteins of both up and downregulated proteins are represented at the centre. The darker the color of the protein, the higher the log_2FC.

Supplementary Fig. S6 online Agarose gel electrophoresis (1.5 %) of qRT-PCR amplified products of target genes. It shows Ladder (100 bp), IDH1 (528 bp), ACACB (424 bp), CD36 (225 bp), FABP3 (227 bp), APOA1 (564 bp), APOE (111 bp), Ladder (100 bp), VTN (286 bp), SERPINC1 (126 bp), PLG (263 bp), TTR (141 bp), TF (217 bp), RPS23 (101 bp), and RPS9 (64 bp).

Supplementary file S1 online

D1vsD4 Up-regulated

Category	Term	Count	%	PValue	Genes				Fold Enricl	Bonferroni	Benjamini	FDR
	GO:0015937~coenzyme A biosynthetic process				ACOT7, PANK3, PANK1	91	10	16801	55.38791	0.424755	0.552615	0.552615
	GO:0006695~cholesterol biosynthetic process				FDPS, IDI1, DHCR7	91	25	16801	22.15516	0.970123	0.912423	0.912423
GOTERM_BP_DIRECT	GO:0097720~calcineurin-mediated signaling				PPP3CA, PPP3CC	91	3	16801	123.0842	0.999231	0.912423	0.912423
	GO:2001295~malonyl-CoA biosynthetic process				ACACB, ACACA	91	3	16801	123.0842	0.999231	0.912423	0.912423
	GO:0006633~fatty acid biosynthetic process				FASN, ACACB, ACACA	91	38	16801	14.57577	0.999631	0.912423	0.912423
GOTERM_BP_DIRECT	GO:0098869~cellular oxidant detoxification				MGST3, MGST2, LPO	91	47	16801	11.78466	0.999993	0.912423	0.912423
	GO:0044341~sodium-dependent phosphate transport				SLC34A2, SLC34A1	91	5	16801	73.85055	0.999994	0.912423	0.912423
	GO:0016310~phosphorylation				PANK3, PFKM, XYLB, MAPK13	91	120	16801	6.154212	0.999994	0.912423	0.912423
	GO:1990592~protein K69-linked ufmylation				UFM1, UBA5	91	6	16801	61.54212	0.999999	0.912423	0.912423
	GO:0006084~acetyl-CoA metabolic process				ACACB, ACACA	91	6	16801	61.54212	0.999999	0.912423	0.912423
	GO:0071569~protein ufmylation				UFM1, UBA5	91	6	16801	61.54212	0.999999	0.912423	0.912423
	GO:0034976~response to endoplasmic reticulum stress				UFM1, UBA5, ALOX15	91	56	16801	9.890699	1	0.912423	0.912423
	GO:0042308~negative regulation of protein import into nucleus				UFM1, CHP1	91	7	16801	52.75039	1	0.912423	0.912423
	GO:0045048~protein insertion into ER membrane				TRAM1, GET4	91	7	16801	52.75039	1	0.912423	0.912423
	GO:0006970~response to osmotic stress				TSC22D4, MAPK13	91	7	16801	52.75039	1	0.912423	0.912423
	GO:0019370~leukotriene biosynthetic process				MGST3, MGST2	91	7	16801	52.75039	1	0.912423	0.912423
	GO:0006817~phosphate ion transport				SLC34A2, SLC34A1	91	7	16801	52.75039	1	0.912423	0.912423
	GO:0006739~NADP metabolic process				IDH1, GCK	91	7	16801	52.75039	1	0.912423	0.912423
	GO:0033146~regulation of intracellular estrogen receptor signaling pathway				UFM1, UBA5	91	8	16801	46.15659	1	0.91326	0.91326
	GO:0006629~lipid metabolic process				BDH1, MGST3, MGST2, AOX1	91	145	16801	5.093141	1	0.91326	0.91326
	GO:0050821~protein stabilization				CTNND1, CHP1, GOLGA7, PFN2	91	149	16801	4.956413	1	0.91326	0.91326
	GO:0098761~cellular response to interleukin-7				HDGF, YBX1	91	9	16801	41.02808	1	0.91326	0.91326
	GO:0030643~cellular phosphate ion homeostasis				SLC34A2, SLC34A1	91	9	16801	41.02808	1	0.91326	0.91326
	GO:0006979~response to oxidative stress				PSMB5, IDH1, LPO	91	68	16801	8.145281	1	0.954392	0.954392
	GO:0061709~reticulophagy				UFM1, UBA5	91	13	16801	28.40406	1	1	1
GOTERM_BP_DIRECT	GO:0033173~calcineurin-NFAT signaling cascade				PPP3CA, PPP3CC	91	13	16801	28.40406	1	1	1
	GO:0045047~protein targeting to ER				SEC61G, SRP14	91	15	16801	24.61685	1	1	1
	GO:0006888~ER to Golgi vesicle-mediated transport				SEC16B, YIPF6, TRAPPC11	91	98	16801	5.651828	1	1	1
	GO:0008544~epidermis development				PPP3CA, YBX1	91	19	16801	19.43436	1	1	1
	GO:0019369~arachidonic acid metabolic process				MGST3, ALOX15	91	19	16801	19.43436	1	1	1

Category	Term	Count	%	PValue	Genes				Fold Enricl	Bonferroni	Benjamini	FDR
	GO:0005737~cytoplasm				CLIC5, IDI1, TXNDC9, TPMT, UBA5, CTNND1, ARHGAP18, LPO, JAKMIP1, STYXL2, NUBP2, GMPPB, PPP3CA, MUC1, TMEM201, PPP3CC, PSMB5, GMPPA, CHP1, AOX1, EIF2D, CD36, CRYM, AAMDC, DTD2, TGM3, KPNA1, IDH1, CAD, ADRM1, DNAJC12, SORBS1, ASS1, MAPK13, FABP3, UFM1, FASN, HDGF, PFKM, PFN2	95	3925	20642	2.214361	5.68E-05	5.68E-05	5.68E-05
	GO:0005829~cytosol				TXNDC9, PANK3, ACSS2, PANK1, UBA5, ALOX15, CTNND1, CLTB, ARHGAP18, YBX1, ACACA, LIMD2, NUBP2, PPP3CA, AOX1, CRYM, XYLB, GET4, KPNA1, FDPS, ACOT7, GLRX3, IDH1, SLC34A1, CAD, STARD10, ADRM1, ASPSCR1, GCK, ASS1, FABP3, FASN	95	2961	20642	2.348225	5.47E-04	2.73E-04	2.73E-04
	GO:0016324~apical plasma membrane				SLC34A2, CLIC5, MUC1, SLC34A1, TCIRG1, CD36, PFKM	95	247	20642	6.157852	0.12354	0.043934	0.043934
	GO:0009898~cytoplasmic side of plasma membrane				PPP3CA, ALOX15, ASPSCR1	95	49	20642	13.30311	0.949856	0.636329	0.636329
	GO:0005955~calcineurin complex				PPP3CA, PPP3CC	95	5	20642	86.91368	0.959969	0.636329	0.636329
	GO:0005783~endoplasmic reticulum				UFM1, ATP8A1, MGST3, YIPF6, AGR2, CHP1, MGST2, YBX1	95	685	20642	2.537626	0.994945	0.775035	0.775035
GOTERM_CC_DIRECT	GO:0005634~nucleus				TXNDC9, PANK3, KHDRBS3, PANK1, UBA5, CTNND1, TCIRG1, YBX1, SRP14, ACACB, NUBP2, MUC1, PSMB5, CHP1, CRYM, KPNA1, GLRX3, ADRM1, SORBS1, H1-0, GCK, MAPK13, FABP3, UFM1, PREB, HDGF, PFKM	95	4064	20642	1.443571	0.996043	0.775035	0.775035
	GO:0005777~peroxisome				IDI1, EHHADH, IDH1	95	75	20642	8.691368	0.998682	0.809724	0.809724
GOTERM_CC_DIRECT	GO:0005815~microtubule organizing center				CLIC5, TXNDC9, NUBP2	95	100	20642	6.518526	0.999986	1	1
	GO:0030176~integral component of endoplasmic reticulum membrane				TRAM1, PREB, DHCR7	95	106	20642	6.149553	0.999996	1	1

Category	Term	Count	%	PValue	Genes				Fold Enricl	Bonferroni	Benjamini	FDR
	GO:0042802~identical protein binding				KHDRBS3, GLRX3, MGST3, CAD, MGST2, ACACB, ASS1, ACACA, RPIA, MPC2, AGR2, YIPF6, PFKM	82	1044	18277	2.775453	0.345196	0.422978	0.422978
GOTERM_MF_DIRECT	GO:0005524~ATP binding				PANK3, ATP8A1, PANK1, UBA5, CAD, ACACB, GCK, ASS1, ACACA, NUBP2, MAPK13, PFKM, MAP3K11, XYLB	82	1415	18277	2.205274	0.842164	0.446883	0.446883
	GO:0003989~acetyl-CoA carboxylase activity				ACACB, ACACA	82	3	18277	148.5935	0.933137	0.446883	0.446883
	GO:1905502~acetyl-CoA binding				PANK3, PANK1	82	3	18277	148.5935	0.933137	0.446883	0.446883
	GO:0042803~protein homodimerization activity				ACOT7, PANK3, PANK1, IDH1, UBA5, AOX1, CRYM, MAP3K11	82	587	18277	3.037686	0.955913	0.446883	0.446883
	GO:0004594~pantothenate kinase activity				PANK3, PANK1	82	4	18277	111.4451	0.972865	0.446883	0.446883
	GO:0033192~calmodulin-dependent protein phosphatase activity				PPP3CA, PPP3CC	82	4	18277	111.4451	0.972865	0.446883	0.446883
	GO:0004464~leukotriene-C4 synthase activity				MGST3, MGST2	82	4	18277	111.4451	0.972865	0.446883	0.446883
GOTERM_MF_DIRECT	GO:0019900~kinase binding				CHP1, JAKMIP1, PFKM	82	59	18277	11.3334	0.996993	0.621548	0.621548
	GO:0005436~sodium:phosphate symporter activity				SLC34A2, SLC34A1	82	7	18277	63.68293	0.998187	0.621548	0.621548
GOTERM_MF_DIRECT	GO:0016597~amino acid binding				CAD, ASS1	82	10	18277	44.57805	0.999879	0.801942	0.801942
	GO:0005085~guanyl-nucleotide exchange factor activity				PLEKHG3, RAB3IP, GBF1, PREB	82	206	18277	4.327966	0.999998	1	1
GOTERM_MF_DIRECT	GO:0051536~iron-sulfur cluster binding				GLRX3, NUBP2	82	15	18277	29.7187	0.999999	1	1
	GO:0004602~glutathione peroxidase activity				MGST3, MGST2	82	17	18277	26.22238	1	1	1

Category	Term	Count	%	PValue	Genes				Fold Enricl	Bonferroni	Benjamini	FDR
	bta01100:Metabolic pathways				IDI1, PANK3, ACSS2, PANK1, HEXB, MGST3, ALOX15, MGST2, TCIRG1, ACACB, ACACA, SGSH, GMPPB, GMPPA, AOX1, XYLB, FDPS, NDUFA4, IDH1, CAD, GCK, ASS1, RPIA, BDH1, EHHADH, FASN, PAH, DHCR7, PFKM	57	1602	9391	2.982445	2.49E-06	2.49E-06	2.37E-06
	bta04922:Glucagon signaling pathway				PPP3CA, PPP3CC, PFKM, ACACB, ACACA, GCK	57	103	9391	9.597343	0.061219	0.031581	0.029993
	bta00640:Propanoate metabolism				ACSS2, EHHADH, ACACB, ACACA	57	31	9391	21.25863	0.133793	0.040702	0.038655
	bta01230:Biosynthesis of amino acids				RPIA, PAH, IDH1, PFKM, ASS1	57	73	9391	11.28455	0.150308	0.040702	0.038655
	bta04910:Insulin signaling pathway				FASN, PPP1R3D, SORBS1, ACACB, ACACA, GCK	57	138	9391	7.163234	0.212555	0.04776	0.045359
KEGG_PATHWAY	bta00520:Amino sugar and nucleotide sugar metabolism				GMPPB, HEXB, GMPPA, GCK	57	52	9391	12.67341	0.476559	0.107694	0.102279
KEGG_PATHWAY	bta01200:Carbon metabolism				RPIA, ACSS2, IDH1, PFKM, GCK	57	112	9391	7.355107	0.543543	0.111792	0.106171
	bta00061:Fatty acid biosynthesis				FASN, ACACB, ACACA	57	18	9391	27.45906	0.594236	0.112464	0.10681
	bta04152:AMPK signaling pathway				FASN, CD36, PFKM, ACACB, ACACA	57	124	9391	6.643322	0.675329	0.124602	0.118337
	bta03060:Protein export				SEC61G, SRP14, SEC11C	57	23	9391	21.4897	0.769071	0.145966	0.138627
	bta01240:Biosynthesis of cofactors				GMPPB, PANK3, PANK1, CAD, GMPPA	57	151	9391	5.455443	0.891869	0.19031	0.180742
	bta03320:PPAR signaling pathway				FABP3, EHHADH, SORBS1, CD36	57	82	9391	8.036799	0.899582	0.19031	0.180742
KEGG_PATHWAY	bta00051:Fructose and mannose metabolism				GMPPB, GMPPA, PFKM	57	34	9391	14.53715	0.956341	0.238778	0.226772
	bta01250:Biosynthesis of nucleotide sugars				GMPPB, GMPPA, GCK	57	39	9391	12.67341	0.982907	0.27873	0.264716
KEGG_PATHWAY	bta05020:Prion disease				PPP3CA, PSMB5, PPP3CC, NDUFA4, ADRM1, MAPK13	57	279	9391	3.543105	0.98894	0.27873	0.264716
	bta05014:Amyotrophic lateral sclerosis				PPP3CA, PSMB5, PPP3CC, NDUFA4, ADRM1, PFN2, MAPK13	57	380	9391	3.034949	0.989069	0.27873	0.264716
	bta00620:Pyruvate metabolism				ACSS2, ACACB, ACACA	57	44	9391	11.23325	0.993978	0.296472	0.281565
	bta04370:VEGF signaling pathway				PPP3CA, PPP3CC, MAPK13	57	58	9391	8.521779	0.999808	0.398509	0.378472
	bta01212:Fatty acid metabolism				EHHADH, FASN, ACACA	57	58	9391	8.521779	0.999808	0.398509	0.378472
	bta04142:Lysosome				HEXB, CLTB, TCIRG1, SGSH	57	139	9391	4.741133	0.999894	0.398509	0.378472
KEGG_PATHWAY	bta00480:Glutathione metabolism				MGST3, IDH1, MGST2	57	61	9391	8.102675	0.999916	0.398509	0.378472
	bta00982:Drug metabolism - cytochrome P450				MGST3, MGST2, AOX1	57	62	9391	7.971986	0.999937	0.398509	0.378472
	bta05208:Chemical carcinogenesis - reactive oxygen species				NDUFA4, MGST3, MGST2, LPO, MAPK13	57	239	9391	3.446744	0.999949	0.398509	0.378472
KEGG_PATHWAY	bta05418:Fluid shear stress and atherosclerosis				MGST3, MGST2, ASS1, MAPK13	57	145	9391	4.544949	0.999962	0.398509	0.378472
	bta00010:Glycolysis / Gluconeogenesis				ACSS2, PFKM, GCK	57	64	9391	7.722862	0.999965	0.398509	0.378472
KEGG_PATHWAY	bta05230:Central carbon metabolism in cancer				IDH1, PFKM, GCK	57	68	9391	7.268576	0.999989	0.423281	0.401999
KEGG_PATHWAY	bta04936:Alcoholic liver disease				FASN, ACACB, ACACA, MAPK13	57	154	9391	4.279335	0.999993	0.423281	0.401999
	bta00983:Drug metabolism - other enzymes				TPMT, MGST3, MGST2	57	76	9391	6.503463	0.999999	0.480974	0.456791

D1vsD4 Down-regulated

Category	Term	Count	%	PValue	Genes	List Total	Pop Hits	Pop Total	Fold Enricl	Bonferron	Benjamini	FDR
GOTERM_BP_DIRECT	GO:0010951~negative regulation of endopeptidase activity	8	4.571429	2.54E-06	SERPINA3-2, SERPINA3-1, SERPIND1, SERPINC1, SERPINF1, SERPING1, HRG, SERPINA3-7	158	66	16929	12.98734	0.002512	0.002515	0.002479
GOTERM_BP_DIRECT	GO:0042744~hydrogen peroxide catabolic process	5	2.857143	7.74E-05	GPX3, HBE2, HBE1, HBE4, APOA4	158	25	16929	21.42911	0.073739	0.026362	0.025989
GOTERM_BP_DIRECT	GO:0098869~cellular oxidant detoxification	6	3.428571	7.99E-05	PRDX4, TXNRD2, GPX3, HBE2, HBE1, HBE4	158	48	16929	13.3932	0.076041	0.026362	0.025989
GOTERM_BP_DIRECT	GO:0033700~phospholipid efflux	4	2.285714	2.09E-04	APOA1, APOC3, APOA4, APOE	158	13	16929	32.96787	0.186955	0.043599	0.042983
GOTERM_BP_DIRECT	GO:1900016~negative regulation of cytokine production involved in inflammatory response	4	2.285714	2.64E-04	CHID1, PDCD4, APOD, F2	158	14	16929	30.61302	0.230205	0.043599	0.042983
GOTERM_BP_DIRECT	GO:0042730~fibrinolysis	4	2.285714	2.64E-04	SERPING1, PLG, HRG, F2	158	14	16929	30.61302	0.230205	0.043599	0.042983
GOTERM_BP_DIRECT	GO:0051918~negative regulation of fibrinolysis	3	1.714286	8.39E-04	PLG, HRG, THBS1	158	5	16929	64.28734	0.564413	0.101262	0.09983
GOTERM_BP_DIRECT	GO:0070328~triglyceride homeostasis	4	2.285714	9.21E-04	APOA1, APOC3, APOA4, APOE	158	21	16929	20.40868	0.59819	0.101262	0.09983
GOTERM_BP_DIRECT	GO:0032489~regulation of Cdc42 protein signal transduction	3	1.714286	0.001251	APOA1, APOC3, APOE	158	6	16929	53.57278	0.710399	0.104585	0.103106
GOTERM_BP_DIRECT	GO:0007596~blood coagulation	5	2.857143	0.001268	SERPIND1, F10, SERPINC1, SERPING1, PLG	158	51	16929	10.50447	0.715158	0.104585	0.103106
GOTERM_BP_DIRECT	GO:0034380~high-density lipoprotein particle assembly	3	1.714286	0.001741	APOA1, APOA4, APOE	158	7	16929	45.91953	0.821794	0.114888	0.113263
GOTERM_BP_DIRECT	GO:0034372~very-low-density lipoprotein particle remodeling	3	1.714286	0.001741	APOA1, APOA4, APOE	158	7	16929	45.91953	0.821794	0.114888	0.113263
GOTERM_BP_DIRECT	GO:0030193~regulation of blood coagulation	3	1.714286	0.001741	SERPINC1, SERPING1, HRG	158	7	16929	45.91953	0.821794	0.114888	0.113263
GOTERM_BP_DIRECT	GO:0010873~positive regulation of cholesterol esterification	3	1.714286	0.002307	APOA1, APOA4, APOE	158	8	16929	40.17959	0.89837	0.142736	0.140718
GOTERM_BP_DIRECT	GO:0043065~positive regulation of apoptotic process	8	4.571429	0.002805	SCIN, STK17B, HTRA1, BAX, IRF5, SCRIB, HRG, CLU	158	202	16929	4.243389	0.938019	0.163355	0.161045
GOTERM_BP_DIRECT	GO:0006869~lipid transport	5	2.857143	0.003284	APOA1, TSPO, APOD, APOA4, APOE	158	66	16929	8.117089	0.961484	0.180629	0.178074
GOTERM_BP_DIRECT	GO:0043691~reverse cholesterol transport	3	1.714286	0.003663	APOA1, APOA4, APOE	158	10	16929	32.14367	0.973551	0.190836	0.188138
GOTERM_BP_DIRECT	GO:1902600~hydrogen ion transmembrane transport	5	2.857143	0.004718	ATP6AP2, ATP1A4, ATP1A1, ATP6V1E2, ATP6V0A1	158	73	16929	7.338738	0.990741	0.228419	0.225189
GOTERM_BP_DIRECT	GO:0050870~positive regulation of T cell activation	4	2.285714	0.005204	LCK, SIRPA, BOLA-DRA, B2M	158	38	16929	11.27848	0.99429	0.228419	0.225189
GOTERM_BP_DIRECT	GO:0030007~cellular potassium ion homeostasis	3	1.714286	0.005307	ATP1A4, ATP1B3, ATP1A1	158	12	16929	26.78639	0.994844	0.228419	0.225189
GOTERM_BP_DIRECT	GO:0034375~high-density lipoprotein particle remodeling	3	1.714286	0.005307	APOA1, APOC3, APOE	158	12	16929	26.78639	0.994844	0.228419	0.225189
GOTERM_BP_DIRECT	GO:0036376~sodium ion export from cell	3	1.714286	0.006234	ATP1A4, ATP1B3, ATP1A1	158	13	16929	24.7259	0.997951	0.246849	0.243358
GOTERM_BP_DIRECT	GO:0046889~positive regulation of lipid biosynthetic process	3	1.714286	0.006234	APOA1, APOA4, APOE	158	13	16929	24.7259	0.997951	0.246849	0.243358
GOTERM_BP_DIRECT	GO:0019882~antigen processing and presentation	4	2.285714	0.006893	RAB4A, BOLA-NC1, BOLA-DRA, BOLA	158	42	16929	10.20434	0.998939	0.25276	0.249186
GOTERM_BP_DIRECT	GO:0032465~regulation of cytokinesis	4	2.285714	0.006893	PRC1, PIK3R4, KIF20A, AURKB	158	42	16929	10.20434	0.998939	0.25276	0.249186
GOTERM_BP_DIRECT	GO:2000379~positive regulation of reactive oxygen species metabolic process	3	1.714286	0.009417	TSPO, F2, THBS1	158	16	16929	20.08979	0.999915	0.33296	0.328251
GOTERM_BP_DIRECT	GO:0006955~immune response	8	4.571429	0.011458	VTN, IL1RN, BOLA-NC1, ENPP1, BOLA-DRA, BOLA, B2M, THBS1	158	263	16929	3.259181	0.999989	0.391136	0.385605
GOTERM_BP_DIRECT	GO:0006883~cellular sodium ion homeostasis	3	1.714286	0.011862	ATP1A4, ATP1B3, ATP1A1	158	18	16929	17.85759	0.999993	0.39145	0.385915
GOTERM_BP_DIRECT	GO:0050821~protein stabilization	6	3.428571	0.012657	PDCD10, AAK1, APOA1, ATP1B3, PPIB, CLU	158	149	16929	4.314587	0.999997	0.404211	0.398495
GOTERM_BP_DIRECT	GO:0043113~receptor clustering	3	1.714286	0.013178	PIGR, SCRIB, ITGAL	158	19	16929	16.91772	0.999998	0.407685	0.401919
GOTERM_BP_DIRECT	GO:0034332~adherens junction organization	3	1.714286	0.020637	CDH1, ADAM10, CSK	158	24	16929	13.3932	1	0.619102	0.610347
GOTERM_BP_DIRECT	GO:0006508~proteolysis	6	3.428571	0.023636	LACTB, F10, MME, HTRA1, PLG, F2	158	175	16929	3.673562	1	0.688235	0.678502
GOTERM_BP_DIRECT	GO:0042632~cholesterol homeostasis	4	2.285714	0.027254	APOA1, APOC3, APOA4, APOE	158	70	16929	6.122604	1	0.718179	0.708023
GOTERM_BP_DIRECT	GO:2001027~negative regulation of endothelial cell chemotaxis	2	1.142857	0.027566	HRG, THBS1	158	3	16929	71.43038	1	0.718179	0.708023
GOTERM_BP_DIRECT	GO:1902995~positive regulation of phospholipid efflux	2	1.142857	0.027566	APOA1, APOE	158	3	16929	71.43038	1	0.718179	0.708023
GOTERM_BP_DIRECT	GO:0034382~chylomicron remnant clearance	2	1.142857	0.027566	APOC3, APOE	158	3	16929	71.43038	1	0.718179	0.708023
GOTERM_BP_DIRECT	GO:2001244~positive regulation of intrinsic apoptotic signaling pathway	3	1.714286	0.029458	LCK, BAX, CLU	158	29	16929	11.08402	1	0.747769	0.737195
GOTERM_BP_DIRECT	GO:0071346~cellular response to interferon-gamma	4	2.285714	0.034767	LOC513659, VIM, GBP4, TLR2	158	77	16929	5.566004	1	0.842339	0.830427
GOTERM_BP_DIRECT	GO:0010903~negative regulation of very-low-density lipoprotein particle remodeling	2	1.142857	0.036586	APOA1, APOC3	158	4	16929	53.57278	1	0.842339	0.830427
GOTERM_BP_DIRECT	GO:0010248~establishment or maintenance of transmembrane electrochemical gradient	2	1.142857	0.036586	BAX, ATP1A1	158	4	16929	53.57278	1	0.842339	0.830427
GOTERM_BP_DIRECT	GO:0051838~cytolysis by host of symbiont cells	2	1.142857	0.036586	HRG, F2	158	4	16929	53.57278	1	0.842339	0.830427
GOTERM_BP_DIRECT	GO:0045087~innate immune response	8	4.571429	0.044289	OAS1X, CHID1, LCK, IRF5, FYN, SAMHD1, JCHAIN, TLR2	158	349	16929	2.456059	1	0.958895	0.945334
GOTERM_BP_DIRECT	GO:0042158~lipoprotein biosynthetic process	2	1.142857	0.045523	APOA1, APOE	158	5	16929	42.85823	1	0.958895	0.945334
GOTERM_BP_DIRECT	GO:0030300~regulation of intestinal cholesterol absorption	2	1.142857	0.045523	APOA1, APOA4	158	5	16929	42.85823	1	0.958895	0.945334
GOTERM_BP_DIRECT	GO:0030641~regulation of cellular pH	2	1.142857	0.045523	ATP1A4, MAPK3	158	5	16929	42.85823	1	0.958895	0.945334
GOTERM_BP_DIRECT	GO:0051256~mitotic spindle midzone assembly	2	1.142857	0.054378	PRC1, AURKB	158	6	16929	35.71519	1	1	0.986855
GOTERM_BP_DIRECT	GO:0086009~membrane repolarization	2	1.142857	0.054378	ATP1B3, ATP1A1	158	6	16929	35.71519	1	1	0.986855
GOTERM_BP_DIRECT	GO:0034115~negative regulation of heterotypic cell-cell adhesion	2	1.142857	0.054378	IL1RN, APOA1	158	6	16929	35.71519	1	1	0.986855
GOTERM_BP_DIRECT	GO:1990573~potassium ion import across plasma membrane	3	1.714286	0.055379	ATP1A4, ATP1B3, ATP1A1	158	41	16929	7.83992	1	1	0.986855
GOTERM_BP_DIRECT	GO:0015671~oxygen transport	2	1.142857	0.063151	HBE2, HBE4	158	7	16929	30.61302	1	1	0.986855
GOTERM_BP_DIRECT	GO:1903588~negative regulation of blood vessel endothelial cell proliferation involved in sprouting angiogenesis	2	1.142857	0.063151	PDCD10, THBS1	158	7	16929	30.61302	1	1	0.986855
GOTERM_BP_DIRECT	GO:0008217~regulation of blood pressure	3	1.714286	0.070452	GNA11, STK39, ATP1A1	158	47	16929	6.839079	1	1	0.986855
GOTERM_BP_DIRECT	GO:0045324~late endosome to vacuole transport	2	1.142857	0.071843	PIK3R4, CHMP3	158	8	16929	26.78639	1	1	0.986855
GOTERM_BP_DIRECT	GO:0051006~positive regulation of lipoprotein lipase activity	2	1.142857	0.080454	APOA1, APOA4	158	9	16929	23.81013	1	1	0.986855
GOTERM_BP_DIRECT	GO:0060425~lung morphogenesis	2	1.142857	0.080454	PLOD3, MAPK3	158	9	16929	23.81013	1	1	0.986855
GOTERM_BP_DIRECT	GO:0043687~post-translational protein modification	2	1.142857	0.080454	STT3B, AURKB	158	9	16929	23.81013	1	1	0.986855
GOTERM_BP_DIRECT	GO:0018401~peptidyl-proline hydroxylation to 4-hydroxy-L-proline	2	1.142857	0.088987	PRDX4, P4HA1	158	10	16929	21.42911	1	1	0.986855
GOTERM_BP_DIRECT	GO:0060020~Bergmann glial cell differentiation	2	1.142857	0.088987	VIM, MAPK3	158	10	16929	21.42911	1	1	0.986855
GOTERM_BP_DIRECT	GO:1905907~negative regulation of amyloid fibril formation	2	1.142857	0.088987	APOE, CLU	158	10	16929	21.42911	1	1	0.986855
GOTERM_BP_DIRECT	GO:0001886~endothelial cell morphogenesis	2	1.142857	0.088987	PLOD3, STC1	158	10	16929	21.42911	1	1	0.986855
GOTERM_BP_DIRECT	GO:2000353~positive regulation of endothelial cell apoptotic process	2	1.142857	0.088987	PDCD4, THBS1	158	10	16929	21.42911	1	1	0.986855
GOTERM_BP_DIRECT	GO:0010898~positive regulation of triglyceride catabolic process	2	1.142857	0.088987	APOA1, APOA4	158	10	16929	21.42911	1	1	0.986855
GOTERM_BP_DIRECT	GO:0044319~wound healing, spreading of cells	2	1.142857	0.088987	PDCD10, CD44	158	10	16929	21.42911	1	1	0.986855
GOTERM_BP_DIRECT	GO:0006144~purine nucleobase metabolic process	2	1.142857	0.088987	TTR, GMPR2	158	10	16929	21.42911	1	1	0.986855
GOTERM_BP_DIRECT	GO:0010976~positive regulation of neuron projection development	3	1.714286	0.09232	TMEM30A, FYN, APOE	158	55	16929	5.844304	1	1	0.986855
GOTERM_BP_DIRECT	GO:0048266~behavioral response to pain	2	1.142857	0.097441	VWA1, THBS1	158	11	16929	19.48101	1	1	0.986855
GOTERM_BP_DIRECT	GO:0016241~regulation of macroautophagy	2	1.142857	0.097441	PIK3R4, ATP6V0A1	158	11	16929	19.48101	1	1	0.986855

Category	Term	Count	%	PValue	Genes	List Total	Pop Hits	Pop Total	Fold Enricl	Bonferron	Benjamini	FDR
GOTERM_CC_DIRECT	GO:0005615~extracellular space	39	22.28571	1.15E-11	IL1RN, TINAGL1, MSTN, SERPINC1, COL14A1, STC1, PLOD3, PLG, CLU, THBS1, VTN, TTR, GPC1, C9, DAG1, ENPP1, APOD, FAM3C, APOE, GC, BOLA, CTSB, CHID1, FGFBP1, BOLA-NC1, SERPINA3-2, SERPINA3-1, F10, SERPIND1, ZG16B, GPX3, SERPINF1, F2, CP, TF, RBP4, TCN2, SERPING1, SERPINA3-7	165	1399	20801	3.514367	2.94E-09	2.94E-09	2.73E-09
GOTERM_CC_DIRECT	GO:0005576~extracellular region	21	12	9.49E-06	CHID1, MSTN, F10, COL14A1, SERPINF1, HTRA1, PLG, LAMC1, SBSN, CP, THBS1, TCN2, C9, DAG1, NUCB1, VWA1, S100A4, MAPT, HRG, B2M, CD44	165	833	20801	3.178151	0.002417	0.00121	0.001125
GOTERM_CC_DIRECT	GO:0042627~chylomicron	4	2.285714	5.54E-05	APOA1, APOC3, APOA4, APOE	165	10	20801	50.42667	0.014039	0.004713	0.00438
GOTERM_CC_DIRECT	GO:0045121~membrane raft	8	4.571429	8.20E-05	MME, LCK, GPC1, ATP1A4, DAG1, FYN, ATP1A1, TLR2	165	131	20801	7.698728	0.020685	0.005225	0.004856
GOTERM_CC_DIRECT	GO:0042583~chromaffin granule	4	2.285714	1.30E-04	SERPINA3-2, SERPINA3-1, CLU, SERPINA3-7	165	13	20801	38.78974	0.032574	0.006623	0.006155
GOTERM_CC_DIRECT	GO:0034361~very-low-density lipoprotein particle	4	2.285714	2.50E-04	APOA1, APOC3, APOA4, APOE	165	16	20801	31.51667	0.06174	0.01062	0.00987
GOTERM_CC_DIRECT	GO:0042470~melanosome	6	3.428571	3.58E-04	SERPINF1, ATP1B3, ATP1A1, PPIB, ATP6V0A1, CTSB	165	77	20801	9.823377	0.087325	0.013051	0.01213
GOTERM_CC_DIRECT	GO:0034364~high-density lipoprotein particle	4	2.285714	4.25E-04	SAA3, APOA1, APOA4, APOE	165	19	20801	26.54035	0.102716	0.013545	0.012589
GOTERM_CC_DIRECT	GO:0034366~spherical high-density lipoprotein particle	3	1.714286	0.001264	APOA1, APOC3, CLU	165	7	20801	54.02857	0.275716	0.030947	0.028763
GOTERM_CC_DIRECT	GO:0070938~contractile ring	3	1.714286	0.001264	PRC1, DAG1, AURKB	165	7	20801	54.02857	0.275716	0.030947	0.028763
GOTERM_CC_DIRECT	GO:0005788~endoplasmic reticulum lumen	5	2.857143	0.00137	TOR4A, OS9, P4HA1, PPIB, SIL1	165	61	20801	10.33333	0.295095	0.030947	0.028763
GOTERM_CC_DIRECT	GO:0005829~cytosol	39	22.28571	0.001456	SHMT2, PIK3R4, HTRA1, STK39, THUMPD3, CLU, HERC4, LACTB, PRDX4, SNX27, PDCD10, GPC1, TSPO, FYN, ZC3H15, ATP6V0A1, MAPK3, CAST, DUSP3, TXNRD2, UCHL5, OAS1X, GGCT, COPS3, MTAP, SNRNP40, LCK, PSME3, PDCD4, CHMP3, BAX, S100A4, MAPT, TBC1D23, PABPC1L, VIM, LSM14A, BLVRA, CD44	165	2967	20801	1.657095	0.310394	0.030947	0.028763
GOTERM_CC_DIRECT	GO:0031410~cytoplasmic vesicle	8	4.571429	0.001892	SERPINA3-2, SERPINA3-1, ATP6AP2, LOC513659, FAM3C, TBC1D23, SERPINA3-7, GBP4	165	221	20801	4.563499	0.383006	0.037111	0.034491
GOTERM_CC_DIRECT	GO:0005890~sodium:potassium-exchanging ATPase complex	3	1.714286	0.002145	ATP1A4, ATP1B3, ATP1A1	165	9	20801	42.02222	0.421614	0.039066	0.036309
GOTERM_CC_DIRECT	GO:0001772~immunological synapse	4	2.285714	0.002838	SNX27, LCK, SCRIB, BOLA-DRA	165	36	20801	14.00741	0.515596	0.048254	0.044848
GOTERM_CC_DIRECT	GO:0009986~cell surface	10	5.714286	0.003498	GPC1, ADAM10, ENPP1, APOA4, BOLA-DRA, HRG, ITGAL, THBS1, CD44, TLR2	165	384	20801	3.282986	0.590794	0.055748	0.051813
GOTERM_CC_DIRECT	GO:0005768~endosome	7	4	0.005154	CYTH2, RAB4A, STEAP4, CDH1, GPC1, FYN, VPS35L	165	200	20801	4.412333	0.73224	0.074843	0.06956
GOTERM_CC_DIRECT	GO:0031838~haptoglobin-hemoglobin complex	3	1.714286	0.005283	HBE2, HBE1, HBE4	165	14	20801	27.01429	0.740955	0.074843	0.06956
GOTERM_CC_DIRECT	GO:0005833~hemoglobin complex	3	1.714286	0.006064	HBE2, HBE1, HBE4	165	15	20801	25.21333	0.787993	0.081391	0.075646
GOTERM_CC_DIRECT	GO:0005794~Golgi apparatus	14	8	0.006855	TMEM30A, ADAM10, HS3ST1, CDH1, PDCD10, NUCB1, FAM3C, TBC1D23, KIF20A, APOE, CD44, ATP6V0A1, MAPK3, TLR2	165	754	20801	2.34076	0.826949	0.087408	0.081238
GOTERM_CC_DIRECT	GO:0005783~endoplasmic reticulum	13	7.428571	0.008399	CAST, TMEM30A, GIMAP1, PLOD3, THBS1, PRDX4, OS9, P4HA1, TSPO, DNAJB9, BAX, APOD, APOE	165	687	20801	2.385541	0.883609	0.101988	0.094789
GOTERM_CC_DIRECT	GO:0048471~perinuclear region of cytoplasm	10	5.714286	0.009049	COPS3, RAB4A, TF, CDH1, S100A4, APOD, PPIB, CLU, ATP6V0A1, CTSB	165	446	20801	2.826607	0.901526	0.104885	0.097481
GOTERM_CC_DIRECT	GO:0016324~apical plasma membrane	7	4	0.013986	TF, TMEM30A, STK39, ATP1B3, ATP1A1, CD44, CTSB	165	248	20801	3.558333	0.972446	0.155063	0.144117
GOTERM_CC_DIRECT	GO:0005911~cell-cell junction	5	2.857143	0.020161	CDH1, DAG1, DSG2, CSK, DSC1	165	131	20801	4.811705	0.994447	0.214205	0.199085
GOTERM_CC_DIRECT	GO:0016020~membrane	16	9.142857	0.02619	CAST, NAPB, MBOAT7, GLYCAM1, PIK3R4, SCRIB, LAMC1, OAS1X, ABHD12, GNG2, GNA11, DAG1, NUCB1, HHIPL1, BAX, DSC1	165	1093	20801	1.845441	0.998849	0.257465	0.239291
GOTERM_CC_DIRECT	GO:0031252~cell leading edge	3	1.714286	0.026251	AAK1, SCRIB, VIM	165	32	20801	11.81875	0.998868	0.257465	0.239291
GOTERM_CC_DIRECT	GO:0034363~intermediate-density lipoprotein particle	2	1.142857	0.031168	APOC3, APOE	165	4	20801	63.03333	0.999689	0.289245	0.268828
GOTERM_CC_DIRECT	GO:0030670~phagocytic vesicle membrane	3	1.714286	0.03267	BOLA-NC1, BOLA, TLR2	165	36	20801	10.50556	0.99979	0.289245	0.268828
GOTERM_CC_DIRECT	GO:0009897~external side of plasma membrane	8	4.571429	0.032895	BOLA-NC1, TCN2, ATP6AP2, DAG1, F2, BOLA, THBS1, CTSB	165	384	20801	2.626389	0.999802	0.289245	0.268828
GOTERM_CC_DIRECT	GO:0031902~late endosome membrane	4	2.285714	0.037296	TMEM30A, CHMP3, BOLA-DRA, CD68	165	93	20801	5.422222	0.999938	0.317016	0.294639
GOTERM_CC_DIRECT	GO:0005802~trans-Golgi network	5	2.857143	0.038804	CHID1, MME, CDH1, PLOD3, TBC1D23	165	161	20801	3.915114	0.999959	0.319193	0.296662
GOTERM_CC_DIRECT	GO:0016323~basolateral plasma membrane	5	2.857143	0.042185	STK39, ATP1B3, SCRIB, ATP1A1, CD44	165	165	20801	3.820202	0.999983	0.336166	0.312436
GOTERM_CC_DIRECT	GO:0005770~late endosome	4	2.285714	0.045792	CHID1, TF, PIK3R4, MAPK3	165	101	20801	4.992739	0.999994	0.347912	0.323353
GOTERM_CC_DIRECT	GO:0031232~extrinsic component of external side of plasma membrane	2	1.142857	0.046388	TF, PLG	165	6	20801	42.02222	0.999995	0.347912	0.323353
GOTERM_CC_DIRECT	GO:0005737~cytoplasm	41	23.42857	0.049429	SHMT2, PIK3R4, THUMPD3, STC1, CLU, AURKB, SDCBP, CYTH2, SULT1C2, SCIN, CDH1, GNA11, DAG1, APOD, CSK, GC, MAPK3, CAST, DUSP3, CLUH, SERPINA3-1, MME, TXNRD2, ADAM10, UCHL5, OAS1X, COPS3, SEPSECS, PRC1, PSME3, AAK1, PDCD4, BAX, IRF5, MAPT, ADSS2, VIM, PPIB, SAE1, LSM14A, BLVRA	165	3943	20801	1.310863	0.999998	0.360127	0.334706
GOTERM_CC_DIRECT	GO:0032133~chromosome passenger complex	2	1.142857	0.053909	CDCA8, AURKB	165	7	20801	36.01905	0.999999	0.381855	0.3549
GOTERM_CC_DIRECT	GO:0097197~tetraspanin-enriched microdomain	2	1.142857	0.061371	ADAM10, SAMHD1	165	8	20801	31.51667	1	0.42296	0.393104
GOTERM_CC_DIRECT	GO:0000220~vacuolar proton-transporting V-type ATPase, V0 domain	2	1.142857	0.076119	ATP6AP2, ATP6V0A1	165	10	20801	25.21333	1	0.486869	0.452502
GOTERM_CC_DIRECT	GO:0071203~WASH complex	2	1.142857	0.076119	SNX27, TBC1D23	165	10	20801	25.21333	1	0.486869	0.452502
GOTERM_CC_DIRECT	GO:0014069~postsynaptic density	4	2.285714	0.076372	ADAM10, FYN, SCRIB, ATP1A1	165	125	20801	4.034133	1	0.486869	0.452502
GOTERM_CC_DIRECT	GO:1990023~mitotic spindle midzone	2	1.142857	0.083407	PRC1, AURKB	165	11	20801	22.92121	1	0.503559	0.468013
GOTERM_CC_DIRECT	GO:0010369~chromocenter	2	1.142857	0.083407	CDCA8, AURKB	165	11	20801	22.92121	1	0.503559	0.468013
GOTERM_CC_DIRECT	GO:0030424~axon	5	2.857143	0.084914	MME, ATP6AP2, ADAM10, ATP1A1, MAPT	165	210	20801	3.001587	1	0.503559	0.468013
GOTERM_CC_DIRECT	GO:0046930~pore complex	2	1.142857	0.097811	ADAM10, BAX	165	13	20801	19.39487	1	0.566858	0.526845

Category	Term	Count	%	PValue	Genes	List Total	Pop Hits	Pop Total	Fold Enricl	Bonferron	Benjamini	FDR
GOTERM_MF_DIRECT	GO:0042802~identical protein binding	26	14.85714	1.34E-06	MSTN, SERPINC1, HTRA1, STC1, SAMHD1, PRDX4, TTR, CDH1, FYN, CSK, APOE, SIL1, MAPK3, RRM1, GPX3, GLYCAM1, APOA1, P4HA1, LCK, PSME3, VWA1, S100A4, IRF5, VIM, SLC25A13, TLR2	152	1047	18413	3.008206	4.30E-04	2.76E-04	2.68E-04
GOTERM_MF_DIRECT	GO:0005515~protein binding	12	6.857143	1.71E-06	CYTH2, GNG2, OS9, DAG1, ADAM10, PLG, ATP1A1, MAPT, VIM, ITGAL, F2, PPIB	152	216	18413	6.729898	5.52E-04	2.76E-04	2.68E-04
GOTERM_MF_DIRECT	GO:0004867~serine-type endopeptidase inhibitor activity	8	4.571429	1.25E-04	SERPINA3-2, SERPINA3-1, SERPIND1, SERPINC1, SERPINF1, SERPING1, HRG, SERPINA3-7	152	135	18413	7.178558	0.039477	0.013425	0.013008
GOTERM_MF_DIRECT	GO:0043236~laminin binding	4	2.285714	3.38E-04	TINAGL1, GPC1, DAG1, THBS1	152	17	18413	28.5031	0.103108	0.0272	0.026356
GOTERM_MF_DIRECT	GO:0060228~phosphatidylcholine-sterol O-acyltransferase activator activity	3	1.714286	6.57E-04	APOA1, APOA4, APOE	152	5	18413	72.68289	0.190823	0.040092	0.038847
GOTERM_MF_DIRECT	GO:0005543~phospholipid binding	6	3.428571	7.47E-04	F10, MYOF, APOA1, APOC3, APOA4, APOE	152	87	18413	8.354356	0.213879	0.040092	0.038847

GOTERM MF DIRECT	GO:0042803~protein homodimerization activity	14	8	0.001128	MSTN, MME, TXNRD2, SHMT2, ADAM10, APOA1, APOA4, JCHAIN, GGCT, STK10, PDCD10, ENPP1, BAX, APOE	152	588	18413	2.884242	0.30463	0.051872	0.050261
GOTERM MF DIRECT	GO:0031721~hemoglobin alpha binding	3	1.714286	0.001366	HBE2, HBE1, HBE4	152	7	18413	51.91635	0.355983	0.054966	0.053259
GOTERM MF DIRECT	GO:0004712~protein serine/threonine/tyrosine kinase activity	9	5.142857	0.001643	STK10, LCK, STK39, PIK3R4, AAK1, FYN, CSK, AURKB, MAPK3	152	267	18413	4.083309	0.411042	0.058774	0.056949
GOTERM MF DIRECT	GO:0030247~polysaccharide binding	3	1.714286	0.002316	VTN, ENPP1, BOLA-DRA	152	9	18413	40.37939	0.526026	0.074574	0.072258
GOTERM MF DIRECT	GO:0008201~heparin binding	6	3.428571	0.004814	MSTN, SERPINC1, APOE, HRG, F2, THBS1	152	133	18413	5.464879	0.788589	0.140824	0.136451
GOTERM MF DIRECT	GO:0015485~cholesterol binding	4	2.285714	0.005248	APOA1, TSPO, APOD, APOA4	152	43	18413	11.26867	0.81628	0.140824	0.136451
GOTERM MF DIRECT	GO:0043177~organic acid binding	3	1.714286	0.005699	HBE2, HBE1, HBE4	152	14	18413	25.95818	0.841228	0.141157	0.136773
GOTERM MF DIRECT	GO:0005507~copper ion binding	4	2.285714	0.008908	CUTA, GPC1, APOA4, CP	152	52	18413	9.31832	0.943941	0.188738	0.182877
GOTERM MF DIRECT	GO:0016209~antioxidant activity	3	1.714286	0.009378	PRDX4, APOA4, APOE	152	18	18413	20.18969	0.95188	0.188738	0.182877
GOTERM MF DIRECT	GO:0005344~oxygen transporter activity	3	1.714286	0.009378	HBE2, HBE1, HBE4	152	18	18413	20.18969	0.95188	0.188738	0.182877
GOTERM MF DIRECT	GO:0019825~oxygen binding	3	1.714286	0.012667	HBE2, HBE1, HBE4	152	21	18413	17.30545	0.983508	0.239931	0.23248
GOTERM MF DIRECT	GO:0005509~calcium ion binding	12	6.857143	0.015646	SCIN, F10, CDH1, DAG1, NUCB1, DSG2, S100A4, F2, DSC1, THBS1, SLC25A13, MATN2	152	635	18413	2.289225	0.993766	0.279883	0.271191
GOTERM MF DIRECT	GO:0008289~lipid binding	5	2.857143	0.017153	CYTH2, APOA1, BAX, APOA4, APOE	152	120	18413	5.047423	0.996194	0.290702	0.281674
GOTERM MF DIRECT	GO:0070653~high-density lipoprotein particle receptor binding	2	1.142857	0.024402	APOA1, APOC3	152	3	18413	80.75877	0.999649	0.383492	0.371583
GOTERM MF DIRECT	GO:0031210~phosphatidylcholine binding	3	1.714286	0.02501	APOA1, APOA4, JCHAIN	152	30	18413	12.11382	0.999713	0.383492	0.371583
GOTERM MF DIRECT	GO:0004175~endopeptidase activity	3	1.714286	0.031577	ADAM10, PLG, CTSB	152	34	18413	10.68866	0.999967	0.453659	0.43957
GOTERM MF DIRECT	GO:0070051~fibrinogen binding	2	1.142857	0.032404	F2, THBS1	152	4	18413	60.56908	0.999975	0.453659	0.43957
GOTERM MF DIRECT	GO:0020037~heme binding	5	2.857143	0.038887	STEAP4, HBE2, HBE1, HBE4, HRG	152	155	18413	3.907683	0.999997	0.517486	0.501415
GOTERM MF DIRECT	GO:0004715~non-membrane spanning protein tyrosine kinase activity	3	1.714286	0.04062	LCK, FYN, CSK	152	39	18413	9.31832	0.999998	0.517486	0.501415
GOTERM MF DIRECT	GO:0005525~GTP binding	8	4.571429	0.042015	RAB4A, GNA11, GIMAP1, LOC513659, ADSS2, SAMHD1, GNL1, GBP4	152	390	18413	2.484885	0.999999	0.517486	0.501415
GOTERM MF DIRECT	GO:0004674~protein serine/threonine kinase activity	7	4	0.043879	STK10, STK17B, STK39, PIK3R4, AAK1, AURKB, MAPK3	152	312	18413	2.717843	0.999999	0.517486	0.501415
GOTERM MF DIRECT	GO:0050839~cell adhesion molecule binding	3	1.714286	0.046458	CDH1, DSG2, ITGAL	152	42	18413	8.652726	1	0.517486	0.501415
GOTERM MF DIRECT	GO:0005391~sodium:potassium-exchanging ATPase activity	2	1.142857	0.048213	ATP1A4, ATP1A1	152	6	18413	40.37939	1	0.517486	0.501415
GOTERM MF DIRECT	GO:0042608~T cell receptor binding	2	1.142857	0.048213	LCK, BOLA-DRA	152	6	18413	40.37939	1	0.517486	0.501415
GOTERM MF DIRECT	GO:0001540~beta-amyloid binding	3	1.714286	0.058974	APOA1, APOE, TLR2	152	48	18413	7.571135	1	0.612566	0.593543
GOTERM MF DIRECT	GO:0043395~heparan sulfate proteoglycan binding	2	1.142857	0.063765	APOE, HRG	152	8	18413	30.28454	1	0.641636	0.62171
GOTERM MF DIRECT	GO:0004713~protein tyrosine kinase activity	3	1.714286	0.074845	LCK, FYN, CSK	152	55	18413	6.607536	1	0.69277	0.671255
GOTERM MF DIRECT	GO:0004252~serine-type endopeptidase activity	5	2.857143	0.075365	F10, HTRA1, PLG, PRSS8, F2	152	194	18413	3.122117	1	0.69277	0.671255
GOTERM MF DIRECT	GO:0042834~peptidoglycan binding	2	1.142857	0.079065	JCHAIN, TLR2	152	10	18413	24.22763	1	0.69277	0.671255
GOTERM MF DIRECT	GO:0016004~phospholipase activator activity	2	1.142857	0.079065	LCK, FYN	152	10	18413	24.22763	1	0.69277	0.671255
GOTERM MF DIRECT	GO:0001786~phosphatidylserine binding	3	1.714286	0.079604	SCIN, MME, THBS1	152	57	18413	6.375693	1	0.69277	0.671255
GOTERM MF DIRECT	GO:0019901~protein kinase binding	7	4	0.082851	CTBP2, PRC1, PDCD10, LCK, ADAM10, IRF5, KIF20A	152	368	18413	2.304258	1	0.697295	0.67564
GOTERM MF DIRECT	GO:0019900~kinase binding	3	1.714286	0.084455	ATP1A4, PLG, AURKB	152	59	18413	6.159567	1	0.697295	0.67564
GOTERM MF DIRECT	GO:0005102~receptor binding	5	2.857143	0.093659	LCK, PLG, FYN, HRG, F2	152	209	18413	2.898042	1	0.739156	0.7162
GOTERM MF DIRECT	GO:0048156~tau protein binding	2	1.142857	0.094116	FYN, APOE	152	12	18413	20.18969	1	0.739156	0.7162

Category	Term	Count	%	PValue	Genes	List Total	Pop Hits	Pop Total	Fold Enricl	Bonferron	Benjamini	FDR
KEGG PATHWAY	bta04610:Complement and coagulation cascades	9	5.142857	1.01E-05	VTN, SERPIND1, F10, SERPINC1, C9, SERPING1, PLG, F2, CLU	107	93	9288	8.400362	0.002454	0.002457	0.002397
KEGG PATHWAY	bta05169:Epstein-Barr virus infection	10	5.714286	0.001186	OAS1X, BOLA-NC1, BAX, BOLA-DRA, VIM, ITGAL, BOLA, B2M, CD44, TLR2	107	229	9288	3.790556	0.251441	0.129346	0.126165
KEGG PATHWAY	bta05416:Viral myocarditis	6	3.428571	0.002032	BOLA-NC1, DAG1, FYN, BOLA-DRA, ITGAL, BOLA	107	79	9288	6.592689	0.391273	0.129346	0.126165
KEGG PATHWAY	bta04979:Cholesterol metabolism	5	2.857143	0.00212	APOA1, TSPO, APOC3, APOA4, APOE	107	48	9288	9.042056	0.404249	0.129346	0.126165
KEGG PATHWAY	bta04612:Antigen processing and presentation	6	3.428571	0.002662	BOLA-NC1, PSME3, BOLA-DRA, BOLA, B2M, CTSB	107	84	9288	6.200267	0.478121	0.129891	0.126697
KEGG PATHWAY	bta05166:Human T-cell leukemia virus 1 infection	9	5.142857	0.004953	BOLA-NC1, LCK, TSPO, BAX, BOLA-DRA, ITGAL, BOLA, B2M, MAPK3	107	232	9288	3.367386	0.702278	0.191112	0.186413
KEGG PATHWAY	bta05170:Human immunodeficiency virus 1 infection	9	5.142857	0.005483	BOLA-NC1, GNG2, GNA11, BAX, SAMHD1, BOLA, B2M, MAPK3, TLR2	107	236	9288	3.310312	0.738538	0.191112	0.186413
KEGG PATHWAY	bta04960:Aldosterone-regulated sodium reabsorption	4	2.285714	0.008471	ATP1A4, ATP1B3, ATP1A1, MAPK3	107	37	9288	9.384188	0.874546	0.258377	0.252023
KEGG PATHWAY	bta04918:Thyroid hormone synthesis	5	2.857143	0.01048	TTR, GPX3, ATP1A4, ATP1B3, ATP1A1	107	75	9288	5.786916	0.923506	0.269776	0.263142
KEGG PATHWAY	bta04145:Phagosome	7	4	0.011056	BOLA-NC1, BOLA-DRA, BOLA, ATP6V1E2, THBS1, ATP6V0A1, TLR2	107	164	9288	3.705038	0.933648	0.269776	0.263142
KEGG PATHWAY	bta01232:Nucleotide metabolism	5	2.857143	0.016016	RRM1, GMPR2, ENPP1, NME3, ADSS2	107	85	9288	5.106102	0.980544	0.350247	0.341634
KEGG PATHWAY	bta05165:Human papillomavirus infection	10	5.714286	0.017442	VTN, BOLA-NC1, BAX, SCRIB, LAMC1, BOLA, ATP6V1E2, THBS1, ATP6V0A1, MAPK3	107	347	9288	2.501549	0.986343	0.350247	0.341634
KEGG PATHWAY	bta04512:ECM-receptor interaction	5	2.857143	0.018661	VTN, DAG1, LAMC1, THBS1, CD44	107	89	9288	4.876615	0.98991	0.350247	0.341634
KEGG PATHWAY	bta04964:Proximal tubule bicarbonate reclamation	3	1.714286	0.025689	ATP1A4, ATP1B3, ATP1A1	107	22	9288	11.83687	0.998253	0.442381	0.431503
KEGG PATHWAY	bta05205:Proteoglycans in cancer	7	4	0.029053	VTN, GPC1, PDCD4, THBS1, CD44, MAPK3, TLR2	107	204	9288	2.97856	0.999249	0.442381	0.431503
KEGG PATHWAY	bta05323:Rheumatoid arthritis	5	2.857143	0.029054	BOLA-DRA, ITGAL, ATP6V1E2, ATP6V0A1, TLR2	107	102	9288	4.255085	0.999249	0.442381	0.431503
KEGG PATHWAY	bta04978:Mineral absorption	4	2.285714	0.030921	TF, ATP1A4, ATP1B3, ATP1A1	107	60	9288	5.786916	0.99953	0.442381	0.431503
KEGG PATHWAY	bta04977:Vitamin digestion and absorption	3	1.714286	0.032635	TCN2, APOA1, APOA4	107	25	9288	10.41645	0.999695	0.442381	0.431503
KEGG PATHWAY	bta04514:Cell adhesion molecules	6	3.428571	0.039382	BOLA-NC1, CDH1, GLYCAM1, BOLA-DRA, ITGAL, BOLA	107	164	9288	3.175747	0.999945	0.505745	0.493308
KEGG PATHWAY	bta04974:Protein digestion and absorption	5	2.857143	0.059829	MME, COL14A1, ATP1A4, ATP1B3, ATP1A1	107	129	9288	3.364486	1	0.710027	0.692567
KEGG PATHWAY	bta05163:Human cytomegalovirus infection	7	4	0.062089	BOLA-NC1, GNG2, GNA11, BAX, BOLA, B2M, MAPK3	107	246	9288	2.470025	1	0.710027	0.692567
KEGG PATHWAY	bta00230:Purine metabolism	5	2.857143	0.064019	RRM1, GMPR2, ENPP1, NME3, ADSS2	107	132	9288	3.28802	1	0.710027	0.692567
KEGG PATHWAY	bta04911:Insulin secretion	4	2.285714	0.072969	GNA11, ATP1A4, ATP1B3, ATP1A1	107	85	9288	4.084882	1	0.773233	0.754219
KEGG PATHWAY	bta05216:Thyroid cancer	3	1.714286	0.076056	CDH1, BAX, MAPK3	107	40	9288	6.51028	1	0.773233	0.754219
KEGG PATHWAY	bta05219:Bladder cancer	3	1.714286	0.082748	CDH1, THBS1, MAPK3	107	42	9288	6.200267	1	0.807616	0.787757
KEGG PATHWAY	bta04520:Adherens junction	4	2.285714	0.087736	CDH1, PDCD10, FYN, MAPK3	107	92	9288	3.774076	1	0.809834	0.78992
KEGG PATHWAY	bta04973:Carbohydrate digestion and absorption	3	1.714286	0.089613	ATP1A4, ATP1B3, ATP1A1	107	44	9288	5.918437	1	0.809834	0.78992
KEGG PATHWAY	bta04925:Aldosterone synthesis and secretion	4	2.285714	0.096698	GNA11, ATP1A4, ATP1B3, ATP1A1	107	96	9288	3.616822	1	0.842652	0.821931

D1vsD7 Up-regulated

Category	Term	Count	%	PValue	Genes	List Total	Pop Hits	Pop Total	Fold Enricl	Bonferron	Benjamini	FDR
GOTERM_BP_DIRECT	GO:1990000~amyloid fibril formation	4	2.072539	2.31E-05	FKBP1A, GSN, RIPK1, CD36	181	6	16929	62.35359	0.020118	0.020323	0.0203
GOTERM_BP_DIRECT	GO:0018120~peptidyl-arginine ADP-ribosylation	4	2.072539	2.42E-04	LOC507756, LOC407148, LOC618664, LOC112441637	181	12	16929	31.1768	0.192078	0.106632	0.106511
GOTERM_BP_DIRECT	GO:0006471~protein ADP-ribosylation	4	2.072539	0.001011	LOC507756, LOC407148, LOC618664, LOC112441637	181	19	16929	19.69061	0.589219	0.242188	0.241913
GOTERM_BP_DIRECT	GO:0009052~pentose-phosphate shunt, non-oxidative branch	3	1.554404	0.001101	RPIA, TALDO1, TKT	181	5	16929	56.11823	0.620646	0.242188	0.241913
GOTERM_BP_DIRECT	GO:0005975~carbohydrate metabolic process	7	3.626943	0.001862	LDHB, MGAM, MDH1, GNPDA2, TALDO1, FBP2, PGM1	181	121	16929	5.410849	0.806051	0.327727	0.327354
GOTERM_BP_DIRECT	GO:0006739~NADP metabolic process	3	1.554404	0.00228	MDH1, IDH1, GCK	181	7	16929	40.08445	0.865787	0.33434	0.33396
GOTERM_BP_DIRECT	GO:0006096~glycolytic process	4	2.072539	0.004674	ENO1, ENO2, ENO3, GCK	181	32	16929	11.6913	0.983806	0.587637	0.586969
GOTERM_BP_DIRECT	GO:0016241~regulation of macroautophagy	3	1.554404	0.005806	PRKAA2, PIK3C3, UBQLN2	181	11	16929	25.50829	0.994047	0.638615	0.637889
GOTERM_BP_DIRECT	GO:0010033~response to organic substance	3	1.554404	0.006918	FKBP1A, ANXA5, SOD1	181	12	16929	23.3826	0.997778	0.67645	0.675681
GOTERM_BP_DIRECT	GO:0043123~positive regulation of I-kappaB kinase/NF-kappaB signaling	6	3.108808	0.013453	FKBP1A, IKBKB, AKAP13, RIPK1, UBE2V1, RELA	181	132	16929	4.251381	0.999993	1	1
GOTERM_BP_DIRECT	GO:0007030~Golgi organization	5	2.590674	0.014427	GAK, SEC16B, LMAN1, ATP8B4, TRAPPC11	181	88	16929	5.314227	0.999997	1	1
GOTERM_BP_DIRECT	GO:0071801~regulation of podosome assembly	2	1.036269	0.021153	HCK, GSN	181	2	16929	93.53039	1	1	1
GOTERM_BP_DIRECT	GO:0006695~cholesterol biosynthetic process	3	1.554404	0.028734	FDPS, IDI1, DHCR7	181	25	16929	11.22365	1	1	1
GOTERM_BP_DIRECT	GO:2001237~negative regulation of extrinsic apoptotic signaling pathway	3	1.554404	0.033158	LGALS3, PSME3, RELA	181	27	16929	10.39227	1	1	1
GOTERM_BP_DIRECT	GO:0019915~lipid storage	3	1.554404	0.035464	LOC518526, PLIN3, CD36	181	28	16929	10.02111	1	1	1
GOTERM_BP_DIRECT	GO:0030041~actin filament polymerization	3	1.554404	0.035464	PSTPIP1, GSN, AIF1	181	28	16929	10.02111	1	1	1
GOTERM_BP_DIRECT	GO:0016310~phosphorylation	5	2.590674	0.039252	IKBKB, PANK3, NME2, MAPK13, GK2	181	120	16929	3.897099	1	1	1
GOTERM_BP_DIRECT	GO:0050819~negative regulation of coagulation	2	1.036269	0.041861	PROC, ANXA5	181	4	16929	46.76519	1	1	1
GOTERM_BP_DIRECT	GO:0046686~response to cadmium ion	2	1.036269	0.041861	SLC34A1, GSS	181	4	16929	46.76519	1	1	1
GOTERM_BP_DIRECT	GO:0010498~proteasomal protein catabolic process	3	1.554404	0.045274	PSMB6, PSMB4, PSMB5	181	32	16929	8.768474	1	1	1
GOTERM_BP_DIRECT	GO:0051092~positive regulation of NF-kappaB transcription factor activity	5	2.590674	0.050101	IKBKB, PLCG2, RIPK1, UBE2V1, RELA	181	130	16929	3.597323	1	1	1
GOTERM_BP_DIRECT	GO:0061077~chaperone-mediated protein folding	3	1.554404	0.05051	FKBP1A, FKBP2, LOC526524	181	34	16929	8.252681	1	1	1
GOTERM_BP_DIRECT	GO:0044341~sodium-dependent phosphate transport	2	1.036269	0.052051	SLC34A2, SLC34A1	181	5	16929	37.41215	1	1	1
GOTERM_BP_DIRECT	GO:0007204~positive regulation of cytosolic calcium ion concentration	4	2.072539	0.062049	FKBP1A, CXCR1, CXCR2, CD36	181	85	16929	4.40143	1	1	1
GOTERM_BP_DIRECT	GO:0071569~protein ufmylation	2	1.036269	0.062133	UFM1, UBA5	181	6	16929	31.1768	1	1	1
GOTERM_BP_DIRECT	GO:1990592~protein K69-linked ufmylation	2	1.036269	0.062133	UFM1, UBA5	181	6	16929	31.1768	1	1	1
GOTERM_BP_DIRECT	GO:0070301~cellular response to hydrogen peroxide	3	1.554404	0.06741	RIPK1, RELA, MAPK13	181	40	16929	7.014779	1	1	1
GOTERM_BP_DIRECT	GO:0042308~negative regulation of protein import into nucleus	2	1.036269	0.072108	UFM1, CHP1	181	7	16929	26.72297	1	1	1
GOTERM_BP_DIRECT	GO:0061635~regulation of protein complex stability	2	1.036269	0.072108	TRAPPC11, SNF8	181	7	16929	26.72297	1	1	1
GOTERM_BP_DIRECT	GO:0006817~phosphate ion transport	2	1.036269	0.072108	SLC34A2, SLC34A1	181	7	16929	26.72297	1	1	1
GOTERM_BP_DIRECT	GO:0019370~leukotriene biosynthetic process	2	1.036269	0.072108	MGST3, MGST2	181	7	16929	26.72297	1	1	1
GOTERM_BP_DIRECT	GO:0045048~protein insertion into ER membrane	2	1.036269	0.072108	TRAM1, GET4	181	7	16929	26.72297	1	1	1
GOTERM_BP_DIRECT	GO:0055062~phosphate ion homeostasis	2	1.036269	0.072108	SLC34A1, ALPL	181	7	16929	26.72297	1	1	1
GOTERM_BP_DIRECT	GO:0035457~cellular response to interferon-alpha	2	1.036269	0.072108	IFIT3, IFIT2	181	7	16929	26.72297	1	1	1
GOTERM_BP_DIRECT	GO:0006970~response to osmotic stress	2	1.036269	0.072108	TSC22D4, MAPK13	181	7	16929	26.72297	1	1	1
GOTERM_BP_DIRECT	GO:0006750~glutathione biosynthetic process	2	1.036269	0.081978	GSS, MGST2	181	8	16929	23.3826	1	1	1
GOTERM_BP_DIRECT	GO:0033146~regulation of intracellular estrogen receptor signaling pathway	2	1.036269	0.081978	UFM1, UBA5	181	8	16929	23.3826	1	1	1
GOTERM_BP_DIRECT	GO:0048678~response to axon injury	2	1.036269	0.091744	PLCG2, SOD1	181	9	16929	20.78453	1	1	1
GOTERM_BP_DIRECT	GO:0006000~fructose metabolic process	2	1.036269	0.091744	PFKFB4, FBP2	181	9	16929	20.78453	1	1	1
GOTERM_BP_DIRECT	GO:0006228~UTP biosynthetic process	2	1.036269	0.091744	CAD, NME2	181	9	16929	20.78453	1	1	1
GOTERM_BP_DIRECT	GO:0030643~cellular phosphate ion homeostasis	2	1.036269	0.091744	SLC34A2, SLC34A1	181	9	16929	20.78453	1	1	1
GOTERM_BP_DIRECT	GO:0098869~cellular oxidant detoxification	3	1.554404	0.092337	MGST3, MGST2, LPO	181	48	16929	5.845649	1	1	1
GOTERM_BP_DIRECT	GO:0035556~intracellular signal transduction	8	4.145078	0.096258	AKAP13, PRKAA2, RPS6KA1, PLCG2, CDC42BPB, GMIP, MAPK13, STK3	181	366	16929	2.04438	1	1	1
GOTERM_BP_DIRECT	GO:0006749~glutathione metabolic process	3	1.554404	0.098933	IDH1, HAGH, SOD1	181	50	16929	5.611823	1	1	1
GOTERM_BP_DIRECT	GO:0006935~chemotaxis	3	1.554404	0.098933	CXCR1, CXCR2, DOCK2	181	50	16929	5.611823	1	1	1

Category	Term	Count	%	PValue	Genes	List Total	Pop Hits	Pop Total	Fold Enricl	Bonferron	Benjamini	FDR
GOTERM_CC_DIRECT	GO:0005829~cytosol	70	36.26943	2.83E-15	PANK3, NUMA1, PANK1, CLTB, IFIT3, LIMD2, IFIT2, IKBKB, PCMT1, AKAP13, RPS6KA1, KIF21B, PGM1, HTATIP2, SCYL1, GET4, CAST, GABARAPL2, GLRX3, SLC34A1, ANXA5, CAD, AMPD2, RAD23A, EDF1, GMIP, TOM1L1, GAK, SKIC3, PSME3, PLIN3, PIK3C3, UBE2V1, STAMBP, FBP2, PFKFB4, FAM114A1, UBA5, TXN, AIF1, GIG	184	2967	20801	2.667147	6.32E-13	6.20E-13	6.12E-13
GOTERM_CC_DIRECT	GO:0005737~cytoplasm	70	36.26943	2.13E-09	TPMT, ENO1, IKBKB, LGALS3, PCMT1, AKAP13, PSTPIP1, PNP, IMPA1, RPS6KA1, GMPPA, CHP1, CD36, HMGN2, HTATIP2, CAST, LOC526524, ANXA5, CAD, NME2, TALDO1, MIF, PTGR1, GAK, DUSP23, DOK3, PSME3, PIK3C3, DOCK2, TARS1, FBP2, PFN2, DCTN6, SASH3, IDI1, LOC790312, UBA5, LPO, GLRX, JAKMIP1, AIF1, REI	184	3943	20801	2.006955	4.65E-07	2.33E-07	2.30E-07
GOTERM_CC_DIRECT	GO:0000015~phosphopyruvate hydratase complex	3	1.554404	4.57E-04	ENO1, ENO2, ENO3	184	4	20801	84.78668	0.095166	0.033327	0.032871
GOTERM_CC_DIRECT	GO:0019774~proteasome core complex, beta-subunit complex	3	1.554404	0.004018	PSMB6, PSMB4, PSMB5	184	11	20801	30.83152	0.585969	0.189832	0.187231
GOTERM_CC_DIRECT	GO:0005777~peroxisome	5	2.590674	0.004334	IDI1, EHHADH, IDH1, PIK3C3, SOD1	184	75	20801	7.536594	0.613731	0.189832	0.187231
GOTERM_CC_DIRECT	GO:0030027~lamellipodium	6	3.108808	0.006687	GSN, CAPRIN1, NME2, CDC42BPB, WASF2, AIF1	184	134	20801	5.061892	0.769943	0.218204	0.215215
GOTERM_CC_DIRECT	GO:0005794~Golgi apparatus	15	7.772021	0.006975	PRKAA2, FAM114A1, UBA5, DBI, RAD23A, FNDC3A, GIGYF2, GAK, LOC518526, PRRC1, PROC, TRAPPC11, SCLY, CD36, SCYL1	184	754	20801	2.248984	0.784065	0.218204	0.215215
GOTERM_CC_DIRECT	GO:0005839~proteasome core complex	3	1.554404	0.014484	PSMB6, PSMB4, PSMB5	184	21	20801	16.14984	0.95904	0.396497	0.391065
GOTERM_CC_DIRECT	GO:0031982~vesicle	4	2.072539	0.02211	GAK, SLC34A2, SLC34A1, GIGYF2	184	68	20801	6.649936	0.992527	0.500696	0.493837
GOTERM_CC_DIRECT	GO:0005741~mitochondrial outer membrane	5	2.590674	0.025325	MSTO1, MGST3, LPIN1, GCK, GK2	184	126	20801	4.486068	0.996366	0.500696	0.493837
GOTERM_CC_DIRECT	GO:0005739~mitochondrion	16	8.290155	0.025984	RANBP2, PIF1, IDH1, HARS2, ACACB, GCK, IFIT3, RELA, GK2, SOD1, PSMB6, MSRA, PSMB4, MTHFD1L, RIPK1, HAGH	184	977	20801	1.851364	0.996867	0.500696	0.493837
GOTERM_CC_DIRECT	GO:0005654~nucleoplasm	29	15.02591	0.027435	KHDRBS3, NUMA1, FAM114A1, TXN, LIMD2, PSMB6, PSMB4, PSMB5, RPS6KA1, OLR1, GET4, RANBP2, FDPS, PRPF38A, CAD, ASPSCR1, RAD23A, EDF1, SNF8, GMIP, MSRA, DUSP23, SKIC3, PSME3, HDGF, MAN2B1, STAMBP, UBE2V1, SNRPA	184	2188	20801	1.498363	0.99774	0.500696	0.493837
GOTERM_CC_DIRECT	GO:0016020~membrane	17	8.80829	0.031997	LGALS3BP, CAST, SLC34A2, MGAM, STARD10, ANXA5, JAKMIP1, GIGYF2, RELA, FKBP1A, TOM1L1, LOC407148, LOC618664, LOC112441637, PIK3C3, STAMBP, CD36	184	1093	20801	1.758309	0.999193	0.502897	0.496008
GOTERM_CC_DIRECT	GO:0005768~endosome	6	3.108808	0.032149	TOM1L1, PIK3C3, STAMBP, FOLR1, GIGYF2, VPS16	184	200	20801	3.391467	0.99922	0.502897	0.496008
GOTERM_CC_DIRECT	GO:0005789~endoplasmic reticulum membrane	11	5.699482	0.034925	SEC16B, LMAN1, GABARAPL2, FKBP2, TRAM1, GPAT4, MGST3, UBA5, MGST2, DHCR7, TKT	184	586	20801	2.122079	0.999584	0.509912	0.502927
GOTERM_CC_DIRECT	GO:0005635~nuclear envelope	4	2.072539	0.050206	RANBP2, MGST3, MGST2, HTATIP2	184	94	20801	4.810592	0.999987	0.687191	0.677778
GOTERM_CC_DIRECT	GO:0005640~nuclear outer membrane	3	1.554404	0.057237	RANBP2, ENO1, DHCR7	184	44	20801	7.70788	0.999998	0.737346	0.727245
GOTERM_CC_DIRECT	GO:0005783~endoplasmic reticulum	11	5.699482	0.082579	CAST, UFM1, PROC, GPAT4, MGST3, AGR2, CHP1, MGST2, DBI, GIGYF2, IFIT2	184	687	20801	1.810099	1	1	0.990826
GOTERM_CC_DIRECT	GO:0005793~endoplasmic reticulum-Golgi intermediate compartment	3	1.554404	0.092451	LMAN1, CHP1, SCYL1	184	58	20801	5.847358	1	1	0.990826
GOTERM_CC_DIRECT	GO:0005776~autophagosome	3	1.554404	0.095162	GABARAPL2, UBQLN2, VPS16	184	59	20801	5.74825	1	1	0.990826

Category	Term	Count	%	PValue	Genes	List Total	Pop Hits	Pop Total	Fold Enricl	Bonferron	Benjamini	FDR
GOTERM_MF_DIRECT	GO:0000287~magnesium ion binding	12	6.217617	2.93E-06	PIF1, ATP8B4, IMPA1, IDH1, GSS, RPS6KA1, ENO1, TKT, ENO2, ENO3, PGM1, STK3	169	205	18413	6.377717	9.63E-04	9.64E-04	9.49E-04
GOTERM_MF_DIRECT	GO:0005524~ATP binding	30	15.54404	3.27E-05	PIF1, PFKFB4, PRKAA2, PANK3, PANK1, UBA5, ACACB, CAMKK2, GK2, STK3, IKBKB, MTHFD1L, RPS6KA1, RIPK1, KIF21B, SCYL1, ATP8B4, GSS, CAD, NME2, CDC42BPB, HARS2, GCK, MAPK13, GAK, HCK, CAPRIN1, PIK3C3, TARS1, GALK2	169	1419	18413	2.303439	0.010705	0.005381	0.005299
GOTERM_MF_DIRECT	GO:0004634~phosphopyruvate hydratase activity	3	1.554404	4.91E-04	ENO1, ENO2, ENO3	169	4	18413	81.7145	0.149085	0.047087	0.046372
GOTERM_MF_DIRECT	GO:0042802~identical protein binding	22	11.39896	5.72E-04	KHDRBS3, GLRX3, MGST3, CAD, MGST2, DBI, HARS2, ACACB, IFIT3, STK3, RPIA, PSTPIP1, UGP2, GNPDA2, MPC2, PSME3, AGR2, OLR1, TARS1, SNRPA, FBP2, UBQLN2	169	1047	18413	2.289359	0.171719	0.047087	0.046372
GOTERM_MF_DIRECT	GO:0003755~peptidyl-prolyl cis-trans isomerase activity	5	2.590674	8.78E-04	FKBP1A, RANBP2, FKBP2, LOC526524, PTPRJ	169	47	18413	11.59071	0.2511	0.055705	0.054859
GOTERM_MF_DIRECT	GO:0042803~protein homodimerization activity	15	7.772021	0.001016	PANK3, PANK1, GSS, UBA5, IDH1, ENO1, TXN, SNF8, RELA, SOD1, FKBP1A, IKBKB, MTHFD1L, RIPK1, TKT	169	588	18413	2.779405	0.284233	0.055705	0.054859
GOTERM_MF_DIRECT	GO:0003950~NAD+ ADP-ribosyltransferase activity	4	2.072539	0.00278	LOC507756, LOC407148, LOC618664, LOC112441637	169	31	18413	14.05841	0.599808	0.130648	0.128663
GOTERM_MF_DIRECT	GO:0005041~low-density lipoprotein receptor activity	3	1.554404	0.003549	LOC518526, OLR1, CD36	169	10	18413	32.6858	0.68955	0.145957	0.143739
GOTERM_MF_DIRECT	GO:0004298~threonine-type endopeptidase activity	3	1.554404	0.007007	PSMB6, PSMB4, PSMB5	169	14	18413	23.347	0.901085	0.233146	0.229603
GOTERM_MF_DIRECT	GO:0004672~protein kinase activity	7	3.626943	0.007086	GAK, IKBKB, PRKAA2, RIPK1, SCYL1, CAMKK2, STK3	169	185	18413	4.122533	0.903649	0.233146	0.229603
GOTERM_MF_DIRECT	GO:0046872~metal ion binding	25	12.95337	0.013118	IDI1, PRKAA2, DYSF, ACACB, AIF1, PPM1F, LIMD2, STK3, UGP2, IMPA1, DTD1, RANBP2, FDPS, ARMT1, GSN, GLRX3, PMM2, NME2, AMPD2, FAH, SOD1, MAN2B1, HAGH, TKT, FBP2	169	1630	18413	1.671053	0.987018	0.392333	0.386371
GOTERM_MF_DIRECT	GO:0004731~purine-nucleoside phosphorylase activity	2	1.036269	0.018165	PNP, LOC790312	169	2	18413	108.9527	0.997598	0.417794	0.411445
GOTERM_MF_DIRECT	GO:0019959~interleukin-8 binding	2	1.036269	0.018165	CXCR1, CXCR2	169	2	18413	108.9527	0.997598	0.417794	0.411445
GOTERM_MF_DIRECT	GO:0047975~guanosine phosphorylase activity	2	1.036269	0.018165	PNP, LOC790312	169	2	18413	108.9527	0.997598	0.417794	0.411445
GOTERM_MF_DIRECT	GO:0016740~transferase activity	4	2.072539	0.019048	RANBP2, FDPS, SCLY, TKT	169	62	18413	7.029204	0.998213	0.417794	0.411445
GOTERM_MF_DIRECT	GO:1905502~acetyl-CoA binding	2	1.036269	0.027124	PANK3, PANK1	169	3	18413	72.63511	0.999882	0.557746	0.54927
GOTERM_MF_DIRECT	GO:0004712~protein serine/threonine/tyrosine kinase activity	7	3.626943	0.035996	IKBKB, HCK, PRKAA2, RPS6KA1, CDC42BPB, MAPK13, STK3	169	267	18413	2.856437	0.999994	0.62341	0.613936
GOTERM_MF_DIRECT	GO:0004594~pantothenate kinase activity	2	1.036269	0.036002	PANK3, PANK1	169	4	18413	54.47633	0.999994	0.62341	0.613936
GOTERM_MF_DIRECT	GO:0004464~leukotriene-C4 synthase activity	2	1.036269	0.036002	MGST3, MGST2	169	4	18413	54.47633	0.999994	0.62341	0.613936
GOTERM_MF_DIRECT	GO:0005085~guanyl-nucleotide exchange factor activity	6	3.108808	0.040739	RIC8A, AKAP13, RAB3IP, GBF1, PREB, DOCK2	169	206	18413	3.173379	0.999999	0.670152	0.659968
GOTERM_MF_DIRECT	GO:0042301~phosphate ion binding	2	1.036269	0.053517	SLC34A2, RELA	169	6	18413	36.31755	1	0.838439	0.825697
GOTERM_MF_DIRECT	GO:0043295~glutathione binding	2	1.036269	0.062156	GSS, MGST2	169	7	18413	31.12933	1	0.841631	0.82884
GOTERM_MF_DIRECT	GO:0016494~C-X-C chemokine receptor activity	2	1.036269	0.062156	CXCR1, CXCR2	169	7	18413	31.12933	1	0.841631	0.82884
GOTERM_MF_DIRECT	GO:0005436~sodium:phosphate symporter activity	2	1.036269	0.062156	SLC34A2, SLC34A1	169	7	18413	31.12933	1	0.841631	0.82884
GOTERM_MF_DIRECT	GO:0005509~calcium ion binding	11	5.699482	0.066096	PROC, GSN, ADGRE5, ANXA5, DYSF, CHP1, ALPL, LPO, TKT, AIF1, CAMKK2	169	635	18413	1.887369	1	0.841631	0.82884
GOTERM_MF_DIRECT	GO:0004674~protein serine/threonine kinase activity	7	3.626943	0.066512	IKBKB, PRKAA2, RPS6KA1, CDC42BPB, HTATIP2, MAPK13, STK3	169	312	18413	2.444451	1	0.841631	0.82884
GOTERM_MF_DIRECT	GO:0051880~G-quadruplex DNA binding	2	1.036269	0.079199	PIF1, NME2	169	9	18413	24.2117	1	0.965049	0.950382

Category	Term	Count	%	PValue	Genes	List Total	Pop Hits	Pop Total	Fold Enricl	Bonferron	Benjamini	FDR
KEGG_PATHWAY	bta01100:Metabolic pathways	49	25.3886	2.79E-10	PANK3, PANK1, ENO1, ENO2, ENO3, PNP, IMPA1, GMPPA, PGM1, CAD, NME2, TALDO1, AMPD2, MIF, BDH1, EHHADH, PIK3C3, HAGH, TKT, FBP2, GNE, IDI1, PFKFB4, LOC790312, MGST3, MGST2, ACACB, GK2, LDHB, UGP2, MTHFD1L, GPAT4, PLCG2, HYI, FDPS, MGAM, MDH1, GSS, IDH1, PMM2, FAH, GCK, RPIA, GNPDA2,	115	1594	9288	2.482745	6.24E-08	6.24E-08	5.77E-08
KEGG_PATHWAY	bta00520:Amino sugar and nucleotide sugar metabolism	8	4.145078	2.96E-06	UGP2, GNPDA2, PMM2, GMPPA, GCK, PGM1, GALK2, GNE	115	52	9288	12.42542	6.62E-04	3.31E-04	3.06E-04
KEGG_PATHWAY	bta01250:Biosynthesis of nucleotide sugars	7	3.626943	7.03E-06	UGP2, PMM2, GMPPA, GCK, PGM1, GALK2, GNE	115	39	9288	14.49632	0.001573	4.58E-04	4.23E-04
KEGG_PATHWAY	bta01200:Carbon metabolism	10	5.181347	8.17E-06	RPIA, MDH1, IDH1, TALDO1, ENO1, TKT, ENO2, ENO3, FBP2, GCK	115	111	9288	7.276146	0.001828	4.58E-04	4.23E-04
KEGG_PATHWAY	bta03320:PPAR signaling pathway	8	4.145078	5.74E-05	LOC518526, FABP3, EHHADH, OLR1, DBI, SORBS1, CD36, GK2	115	81	9288	7.976812	0.012766	0.00257	0.002375
KEGG_PATHWAY	bta01240:Biosynthesis of cofactors	10	5.181347	9.01E-05	UGP2, PANK3, MTHFD1L, PANK1, GSS, CAD, PMM2, GMPPA, NME2, ALPL	115	150	9288	5.384348	0.019971	0.003362	0.003107
KEGG_PATHWAY	bta00010:Glycolysis / Gluconeogenesis	7	3.626943	1.26E-04	LDHB, ENO1, ENO2, ENO3, FBP2, GCK, PGM1	115	64	9288	8.833696	0.027798	0.004027	0.003722
KEGG_PATHWAY	bta04920:Adipocytokine signaling pathway	7	3.626943	2.24E-04	IKBKB, LOC518526, PRKAA2, CD36, ACACB, RELA, CAMKK2	115	71	9288	7.962768	0.048994	0.006279	0.005802
KEGG_PATHWAY	bta01230:Biosynthesis of amino acids	7	3.626943	2.61E-04	RPIA, IDH1, TALDO1, ENO1, TKT, ENO2, ENO3	115	73	9288	7.74461	0.056856	0.006503	0.00601
KEGG_PATHWAY	bta04931:Insulin resistance	8	4.145078	3.71E-04	IKBKB, LOC518526, PRKAA2, PPP1R3D, RPS6KA1, CD36, ACACB, RELA	115	109	9288	5.927722	0.079712	0.008216	0.007592
KEGG_PATHWAY	bta00030:Pentose phosphate pathway	5	2.590674	4.03E-04	RPIA, TALDO1, TKT, FBP2, PGM1	115	29	9288	13.92504	0.086426	0.008216	0.007592
KEGG_PATHWAY	bta04936:Alcoholic liver disease	8	4.145078	0.002712	IKBKB, PRKAA2, RIPK1, LPIN1, ACACB, RELA, MAPK13, CAMKK2	115	153	9288	4.223018	0.455735	0.050625	0.046782
KEGG_PATHWAY	bta04152:AMPK signaling pathway	7	3.626943	0.003977	LOC518526, PFKFB4, PRKAA2, CD36, ACACB, FBP2, CAMKK2	115	123	9288	4.596394	0.590416	0.068526	0.063325
KEGG_PATHWAY	bta00052:Galactose metabolism	4	2.072539	0.006303	MGAM, UGP2, GCK, PGM1	115	31	9288	10.42132	0.757418	0.09713	0.089759
KEGG_PATHWAY	bta00500:Starch and sucrose metabolism	4	2.072539	0.006894	MGAM, UGP2, GCK, PGM1	115	32	9288	10.09565	0.787667	0.09713	0.089759
KEGG_PATHWAY	bta04910:Insulin signaling pathway	7	3.626943	0.006938	IKBKB, PRKAA2, PPP1R3D, SORBS1, ACACB, FBP2, GCK	115	138	9288	4.096786	0.78976	0.09713	0.089759

KEGG_PATHWAY	bta00051:Fructose and mannose metabolism	4	2.072539	0.008171	PFKFB4, PMM2, GMPPA, FBP2	115	34	9288	9.50179	0.840841	0.107666	0.099495
KEGG_PATHWAY	bta05418:Fluid shear stress and atherosclerosis	7	3.626943	0.008763	IKBKB, PRKAA2, MGST3, MGST2, TXN, RELA, MAPK13	115	145	9288	3.89901	0.860762	0.109051	0.100775
KEGG_PATHWAY	bta04066:HIF-1 signaling pathway	6	3.108808	0.01125	LDHB, PLCG2, ENO1, ENO2, ENO3, RELA	115	110	9288	4.405375	0.920683	0.132633	0.122567
KEGG_PATHWAY	bta05132:Salmonella infection	9	4.663212	0.014222	DCTN6, IKBKB, RIPK1, PIK3C3, TXN, RELA, PFN2, VPS16, MAPK13	115	259	9288	2.806513	0.95959	0.15929	0.147201
KEGG_PATHWAY	bta00620:Pyruvate metabolism	4	2.072539	0.016556	LDHB, MDH1, HAGH, ACACB	115	44	9288	7.342292	0.976235	0.176596	0.163193
KEGG_PATHWAY	bta05014:Amyotrophic lateral sclerosis	11	5.699482	0.017856	DCTN6, PSMB6, RANBP2, PSMB4, PSMB5, ALYREF, PIK3C3, UBQLN2, PFN2, SOD1, MAPK13	115	379	9288	2.344109	0.982331	0.18181	0.168012
KEGG_PATHWAY	bta03050:Proteasome	4	2.072539	0.019742	PSMB6, PSMB4, PSMB5, PSME3	115	47	9288	6.873636	0.988511	0.192267	0.177676
KEGG_PATHWAY	bta05208:Chemical carcinogenesis - reactive oxygen species	8	4.145078	0.027544	IKBKB, MGST3, MGST2, PTPRJ, LPO, RELA, SOD1, MAPK13	115	239	9288	2.703438	0.998082	0.257078	0.237568
KEGG_PATHWAY	bta04062:Chemokine signaling pathway	7	3.626943	0.02931	IKBKB, HCK, CXCR1, CXCR2, PLCG2, DOCK2, RELA	115	190	9288	2.975561	0.998723	0.258184	0.23859
KEGG_PATHWAY	bta04621:NOD-like receptor signaling pathway	7	3.626943	0.029968	IKBKB, GABARAPL2, PSTPIP1, RIPK1, TXN, RELA, MAPK13	115	191	9288	2.959982	0.998903	0.258184	0.23859
KEGG_PATHWAY	bta04922:Glucagon signaling pathway	5	2.590674	0.037677	LDHB, PRKAA2, ACACB, FBP2, GCK	115	103	9288	3.920642	0.999816	0.310111	0.286576
KEGG_PATHWAY	bta00480:Glutathione metabolism	4	2.072539	0.038764	MGST3, IDH1, GSS, MGST2	115	61	9288	5.29608	0.999857	0.310111	0.286576
KEGG_PATHWAY	bta00640:Propanoate metabolism	3	1.554404	0.055121	LDHB, EHHADH, ACACB	115	31	9288	7.815989	0.999997	0.425763	0.393451
KEGG_PATHWAY	bta04141:Protein processing in endoplasmic reticulum	6	3.108808	0.059101	LMAN1, TRAM1, SEC61G, PREB, RAD23A, UBQLN2	115	171	9288	2.833867	0.999999	0.441285	0.407794
KEGG_PATHWAY	bta04722:Neurotrophin signaling pathway	5	2.590674	0.06291	IKBKB, RPS6KA1, PLCG2, RELA, MAPK13	115	122	9288	3.31005	1	0.454576	0.420077
KEGG_PATHWAY	bta00983:Drug metabolism - other enzymes	4	2.072539	0.06648	TPMT, MGST3, NME2, MGST2	115	76	9288	4.250801	1	0.46536	0.430043
KEGG_PATHWAY	bta03018:RNA degradation	4	2.072539	0.070711	SKIC3, ENO1, ENO2, ENO3	115	78	9288	4.141806	1	0.479981	0.443554
KEGG_PATHWAY	bta00230:Purine metabolism	5	2.590674	0.079024	PNP, LOC790312, NME2, AMPD2, PGM1	115	132	9288	3.059289	1	0.520631	0.481119
KEGG_PATHWAY	bta01232:Nucleotide metabolism	4	2.072539	0.086441	PNP, LOC790312, NME2, AMPD2	115	85	9288	3.800716	1	0.55322	0.511235

D1vsD7 Down-regulated

Category	Term	Count	%	PValue	Genes	List Total	Pop Hits	Pop Total	Fold Enricl	Bonferron	Benjamini	FDR
GOTERM_BP_DIRECT	GO:0098869~cellular oxidant detoxification	9	3.10345	7.12E-07	PRDX4, TXNRD2, GPX3, NXN, HBE2, HBE1, GSTA1, HBE4, CSN2	266	48	16929	11.933	0.00107	0.00107	0.00106
GOTERM_BP_DIRECT	GO:0000226~microtubule cytoskeleton organization	10	3.44828	9.97E-05	EML4, TUBA1C, TUBA3E, TUBB2B, TUBB2A, MAP2, MAPT, MARK4, TUBB4B, TUBA4A	266	118	16929	5.39346	0.13966	0.07521	0.07441
GOTERM_BP_DIRECT	GO:0006268~DNA unwinding involved in DNA replication	5	1.72414	1.89E-04	MCM7, MCM3, RPA1, MCM5, MCM2	266	19	16929	16.7481	0.24819	0.07559	0.07479
GOTERM_BP_DIRECT	GO:0010873~positive regulation of cholesterol esterification	4	1.37931	2.00E-04	APOA1, APOA4, APOE, AGT	266	8	16929	31.8214	0.26096	0.07559	0.07479
GOTERM_BP_DIRECT	GO:0000281~mitotic cytokinesis	6	2.06897	2.98E-04	USP8, ROCK1, RACGAP1, KIF4A, KIF20A, MYH10	266	38	16929	10.0489	0.36195	0.08684	0.08592
GOTERM_BP_DIRECT	GO:0043065~positive regulation of apoptotic process	12	4.13793	3.50E-04	ITGB1, MTCH2, B4GALT1, SCIN, LGALS1, AIFM2, DAPK1, HTRA1, HIP1R, SCRIB, HRG, CLU	266	202	16929	3.78076	0.41005	0.08684	0.08592
GOTERM_BP_DIRECT	GO:0019882~antigen processing and presentation	6	2.06897	4.80E-04	CD74, BOLA-NC1, BOLA-DQB, GBA1, BOLA-DRA, BOLA	266	42	16929	9.09184	0.51508	0.08684	0.08592
GOTERM_BP_DIRECT	GO:0050821~protein stabilization	10	3.44828	5.72E-04	USP7, PDCD10, GNAQ, HIP1R, AAK1, APOA1, CSN3, PPIB, CLU, RTN4	266	149	16929	4.27133	0.578	0.08684	0.08592
GOTERM_BP_DIRECT	GO:0042744~hydrogen peroxide catabolic process	5	1.72414	5.73E-04	GPX3, HBE2, HBE1, HBE4, APOA4	266	25	16929	12.7286	0.57891	0.08684	0.08592
GOTERM_BP_DIRECT	GO:0010951~negative regulation of endopeptidase activity	7	2.41379	5.76E-04	SERPINB12, SERPIND1, AHSG, SERPINC1, TIMP3, SERPING1, HRG	266	66	16929	6.75	0.5805	0.08684	0.08592
GOTERM_BP_DIRECT	GO:0000727~double-strand break repair via break-induced replication	4	1.37931	7.51E-04	MCM7, MCM3, MCM5, MCM2	266	12	16929	21.2143	0.67838	0.09419	0.09319
GOTERM_BP_DIRECT	GO:0030007~cellular potassium ion homeostasis	4	1.37931	7.51E-04	ATP1A4, ATP1A3, ATP1A2, ATP1A1	266	12	16929	21.2143	0.67838	0.09419	0.09319
GOTERM_BP_DIRECT	GO:0008217~regulation of blood pressure	6	2.06897	8.11E-04	GNA11, STK39, ATP1A2, ATP1A1, ATP2B1, CSN2	266	47	16929	8.12462	0.70623	0.09419	0.09319
GOTERM_BP_DIRECT	GO:1902430~negative regulation of beta-amyloid formation	4	1.37931	9.66E-04	RTN2, ROCK1, APOE, RTN4	266	13	16929	19.5824	0.76726	0.0966	0.09558
GOTERM_BP_DIRECT	GO:0036376~sodium ion export from cell	4	1.37931	9.66E-04	ATP1A4, ATP1A3, ATP1A2, ATP1A1	266	13	16929	19.5824	0.76726	0.0966	0.09558
GOTERM_BP_DIRECT	GO:0007017~microtubule-based process	5	1.72414	0.00102	TUBA3E, TUBB2B, TUBB2A, TUBB4B, TUBA4A	266	29	16929	10.9729	0.787	0.0966	0.09558
GOTERM_BP_DIRECT	GO:0042730~fibrinolysis	4	1.37931	0.00121	SERPING1, PLG, HRG, F2	266	14	16929	18.1837	0.84027	0.10783	0.10669
GOTERM_BP_DIRECT	GO:0016477~cell migration	11	3.7931	0.00133	ITGB1, JUP, SDC4, GPC1, FSCN1, CTNNA3, SCRIB, LAMC1, GPC4, CTNNA2, THBS1	266	202	16929	3.4657	0.86654	0.11181	0.11063
GOTERM_BP_DIRECT	GO:0051480~regulation of cytosolic calcium ion concentration	5	1.72414	0.00149	TRPC5, TRPC4, ATP2B4, ATP2B3, ATP2B1	266	32	16929	9.9442	0.89538	0.11872	0.11746
GOTERM_BP_DIRECT	GO:0043537~negative regulation of blood vessel endothelial cell migration	4	1.37931	0.00183	JUP, ATP2B4, APOE, HRG	266	16	16929	15.9107	0.9366	0.13122	0.12983
GOTERM_BP_DIRECT	GO:0060339~negative regulation of type I interferon-mediated signaling pathway	4	1.37931	0.00183	YTHDF2, YTHDF3, ISG15, SAMHD1	266	16	16929	15.9107	0.9366	0.13122	0.12983
GOTERM_BP_DIRECT	GO:0051918~negative regulation of fibrinolysis	3	1.03448	0.00237	PLG, HRG, THBS1	266	5	16929	38.1857	0.97198	0.1623	0.16058
GOTERM_BP_DIRECT	GO:0006883~cellular sodium ion homeostasis	4	1.37931	0.0026	ATP1A4, ATP1A3, ATP1A2, ATP1A1	266	18	16929	14.1429	0.98033	0.1706	0.16879
GOTERM_BP_DIRECT	GO:0006270~DNA replication initiation	4	1.37931	0.00409	MCM7, MCM3, MCM5, MCM2	266	21	16929	12.1224	0.99795	0.22952	0.22709
GOTERM_BP_DIRECT	GO:0018149~peptide cross-linking	4	1.37931	0.00409	KRT2, KRT1, KRT10, THBS1	266	21	16929	12.1224	0.99795	0.22952	0.22709
GOTERM_BP_DIRECT	GO:0006979~response to oxidative stress	6	2.06897	0.00423	APP, PRDX4, GPX3, PSIP1, APOE, HMOX2	266	68	16929	5.61555	0.99833	0.22952	0.22709
GOTERM_BP_DIRECT	GO:0006874~cellular calcium ion homeostasis	6	2.06897	0.0045	STOML2, ATP2B4, ATP2B3, ATP2A1, ATP2B1, APOE	266	69	16929	5.53416	0.99889	0.22952	0.22709
GOTERM_BP_DIRECT	GO:0034063~stress granule assembly	4	1.37931	0.00469	YTHDF2, YTHDF3, CIRBP, LSM14A	266	22	16929	11.5714	0.99916	0.22952	0.22709
GOTERM_BP_DIRECT	GO:0046034~ATP metabolic process	4	1.37931	0.00469	ENPP1, AK4, ATP1A2, ENPP3	266	22	16929	11.5714	0.99916	0.22952	0.22709
GOTERM_BP_DIRECT	GO:0034372~very-low-density lipoprotein particle remodeling	3	1.03448	0.00487	APOA1, APOA4, APOE	266	7	16929	27.2755	0.99937	0.22952	0.22709
GOTERM_BP_DIRECT	GO:0034380~high-density lipoprotein particle assembly	3	1.03448	0.00487	APOA1, APOA4, APOE	266	7	16929	27.2755	0.99937	0.22952	0.22709
GOTERM_BP_DIRECT	GO:0030193~regulation of blood coagulation	3	1.03448	0.00487	SERPINC1, SERPING1, HRG	266	7	16929	27.2755	0.99937	0.22952	0.22709
GOTERM_BP_DIRECT	GO:0016579~protein deubiquitination	6	2.06897	0.00572	USP8, USP15, USP7, USP4, USP39, UCHL5	266	73	16929	5.23092	0.99983	0.25388	0.25119
GOTERM_BP_DIRECT	GO:1902600~hydrogen ion transmembrane transport	6	2.06897	0.00572	ATP6AP2, ATP1A4, ATP1A3, ATP1A2, ATP1A1, ATP6V0A1	266	73	16929	5.23092	0.99983	0.25388	0.25119
GOTERM_BP_DIRECT	GO:0046007~negative regulation of activated T cell proliferation	3	1.03448	0.00642	ARG1, SCRIB, IDO1	266	8	16929	23.8661	0.99994	0.26195	0.25917
GOTERM_BP_DIRECT	GO:1902669~positive regulation of axon guidance	3	1.03448	0.00642	TUBB2B, TUBB2A, TUBB4B	266	8	16929	23.8661	0.99994	0.26195	0.25917
GOTERM_BP_DIRECT	GO:0034340~response to type I interferon	3	1.03448	0.00642	SHMT2, MX1, ISG15	266	8	16929	23.8661	0.99994	0.26195	0.25917
GOTERM_BP_DIRECT	GO:0006749~glutathione metabolic process	5	1.72414	0.00769	CLIC5, GSTA5, GSTA2, GSTA1, CLIC2	266	50	16929	6.36429	0.99999	0.30529	0.30206
GOTERM_BP_DIRECT	GO:0007596~blood coagulation	5	1.72414	0.00824	SERPIND1, F10, SERPINC1, SERPING1, PLG	266	51	16929	6.2395	1	0.31702	0.31366
GOTERM_BP_DIRECT	GO:0006826~iron ion transport	4	1.37931	0.0084	TF, TFRC, FTH1, CP	266	27	16929	9.42857	1	0.31702	0.31366
GOTERM_BP_DIRECT	GO:0006955~immune response	11	3.7931	0.00867	CD74, VTN, IL1RN, BOLA-NC1, FTH1, ENPP1, BOLA-DQB, BOLA-DRA, BOLA, B2M, THBS1	266	263	16929	2.66187	1	0.3191	0.31572
GOTERM_BP_DIRECT	GO:0043691~reverse cholesterol transport	3	1.03448	0.01011	APOA1, APOA4, APOE	266	10	16929	19.0929	1	0.3317	0.32818
GOTERM_BP_DIRECT	GO:0007213~G-protein coupled acetylcholine receptor signaling pathway	3	1.03448	0.01011	GNAQ, GNA11, AGRN	266	10	16929	19.0929	1	0.3317	0.32818
GOTERM_BP_DIRECT	GO:0006144~purine nucleobase metabolic process	3	1.03448	0.01011	TTR, GMPR2, GMPR	266	10	16929	19.0929	1	0.3317	0.32818
GOTERM_BP_DIRECT	GO:0051290~protein heterotetramerization	3	1.03448	0.01011	RRM1, KRT1, KRT10	266	10	16929	19.0929	1	0.3317	0.32818
GOTERM_BP_DIRECT	GO:0061157~mRNA destabilization	3	1.03448	0.01011	YTHDF2, YTHDF3, ROCK1	266	10	16929	19.0929	1	0.3317	0.32818
GOTERM_BP_DIRECT	GO:0006099~tricarboxylic acid cycle	4	1.37931	0.01346	PDHA1, IDH2, SDHA, IDH3A	266	32	16929	7.95536	1	0.41657	0.41215
GOTERM_BP_DIRECT	GO:0019886~antigen processing and presentation of exogenous peptide antigen via MHC class II	4	1.37931	0.01346	CD74, BOLA-DQB, BOLA-DRA, B2M	266	32	16929	7.95536	1	0.41657	0.41215
GOTERM_BP_DIRECT	GO:0000278~mitotic cell cycle	6	2.06897	0.01353	TUBA1C, TUBA3E, TUBB2B, TUBB2A, TUBB4B, TUBA4A	266	90	16929	4.24286	1	0.41657	0.41215
GOTERM_BP_DIRECT	GO:0072659~protein localization to plasma membrane	7	2.41379	0.01492	JUP, ROCK1, CDH1, CD81, RDX, LRP6, S100A10	266	127	16929	3.50787	1	0.45014	0.44536
GOTERM_BP_DIRECT	GO:0046889~positive regulation of lipid biosynthetic process	3	1.03448	0.01699	APOA1, APOA4, APOE	266	13	16929	14.6868	1	0.49314	0.48791
GOTERM_BP_DIRECT	GO:0033700~phospholipid efflux	3	1.03448	0.01699	APOA1, APOA4, APOE	266	13	16929	14.6868	1	0.49314	0.48791
GOTERM_BP_DIRECT	GO:0045445~myoblast differentiation	3	1.03448	0.01962	ITGB1, LGALS1, PLG	266	14	16929	13.6378	1	0.54417	0.5384
GOTERM_BP_DIRECT	GO:1900016~negative regulation of cytokine production involved in inflammatory response	3	1.03448	0.01962	CHID1, PDCD4, F2	266	14	16929	13.6378	1	0.54417	0.5384
GOTERM_BP_DIRECT	GO:0016525~negative regulation of angiogenesis	5	1.72414	0.01983	ROCK1, ATP2B4, HRG, THBS1, GTF2I	266	66	16929	4.82143	1	0.54417	0.5384
GOTERM_BP_DIRECT	GO:2000379~positive regulation of reactive oxygen species metabolic process	3	1.03448	0.02535	F2, XDH, THBS1	266	16	16929	11.933	1	0.67116	0.66405
GOTERM_BP_DIRECT	GO:0002476~antigen processing and presentation of endogenous peptide antigen via MHC class Ib	3	1.03448	0.02535	BOLA-NC1, LOC616942, BOLA	266	16	16929	11.933	1	0.67116	0.66405
GOTERM_BP_DIRECT	GO:1990573~potassium ion import across plasma membrane	4	1.37931	0.02611	ATP1A4, ATP1A3, ATP1A2, ATP1A1	266	41	16929	6.20906	1	0.67925	0.67205
GOTERM_BP_DIRECT	GO:0006468~protein phosphorylation	8	2.75862	0.02727	APP, ROCK1, PIK3R4, AAK1, ADAM10, FAM20C, CSNK1D	266	186	16929	2.73733	1	0.69743	0.69004
GOTERM_BP_DIRECT	GO:0032355~response to estradiol	3	1.03448	0.02844	CSN3, CSN2, GGT1	266	17	16929	11.2311	1	0.70354	0.69608
GOTERM_BP_DIRECT	GO:0032012~regulation of ARF protein signal transduction	3	1.03448	0.02844	CYTH3, CYTH2, CYTH1	266	17	16929	11.2311	1	0.70354	0.69608
GOTERM_BP_DIRECT	GO:0006898~receptor-mediated endocytosis	4	1.37931	0.02955	HIP1R, APOE, GUSB, AP2A2	266	43	16929	5.92027	1	0.71915	0.71153
GOTERM_BP_DIRECT	GO:1903416~response to glycoside	2	0.68966	0.03106	ATP1A2, ATP1A1	266	2	16929	63.6429	1	0.73523	0.72744
GOTERM_BP_DIRECT	GO:0050728~negative regulation of inflammatory response	5	1.72414	0.03132	KRT1, APOA1, ENPP3, APOE, CSN2	266	76	16929	4.18703	1	0.73523	0.72744
GOTERM_BP_DIRECT	GO:0006953~acute-phase response	3	1.03448	0.03167	AHSG, SAA3, F2	266	18	16929	10.6071	1	0.73523	0.72744
GOTERM_BP_DIRECT	GO:0070588~calcium ion transmembrane transport	4	1.37931	0.03322	TRPC5, TRPC4, ATP2B4, ATP2A1	266	45	16929	5.65714	1	0.75942	0.75137
GOTERM_BP_DIRECT	GO:0043113~receptor clustering	3	1.03448	0.03504	PIGR, SCRIB, AGRN	266	19	16929	10.0489	1	0.78912	0.78075
GOTERM_BP_DIRECT	GO:0030500~regulation of bone mineralization	3	1.03448	0.03854	ECM1, MGP, ENPP1	266	20	16929	9.54643	1	0.85515	0.84608
GOTERM_BP_DIRECT	GO:0070328~triglyceride homeostasis	3	1.03448	0.04216	APOA1, APOA4, APOE	266	21	16929	9.09184	1	0.89442	0.88493
GOTERM_BP_DIRECT	GO:0033344~cholesterol efflux	3	1.03448	0.04216	APOA1, APOA4, APOE	266	21	16929	9.09184	1	0.89442	0.88493
GOTERM_BP_DIRECT	GO:0031175~neuron projection development	5	1.72414	0.04282	APP, MAP2, MAPT, APOE, ATXN10	266	84	16929	3.78827	1	0.89442	0.88493
GOTERM_BP_DIRECT	GO:0051894~positive regulation of focal adhesion assembly	3	1.03448	0.04591	ROCK1, HRG, S100A10	266	22	16929	8.67857	1	0.89442	0.88493
GOTERM_BP_DIRECT	GO:0042157~lipoprotein metabolic process	3	1.03448	0.04591	APOA1, APOA4, APOE	266	22	16929	8.67857	1	0.89442	0.88493
GOTERM_BP_DIRECT	GO:0006986~response to unfolded protein	3	1.03448	0.04591	DNAJC3, STT3B, THBS1	266	22	16929	8.67857	1	0.89442	0.88493
GOTERM_BP_DIRECT	GO:0042832~defense response to protozoan	3	1.03448	0.04591	ARG1, LOC513659, GBP4	266	22	16929	8.67857	1	0.89442	0.88493
GOTERM_BP_DIRECT	GO:1902995~positive regulation of phospholipid efflux	2	0.68966	0.04623	APOA1, APOE	266	3	16929	42.4286	1	0.89442	0.88493
GOTERM_BP_DIRECT	GO:0045822~negative regulation of heart contraction	2	0.68966	0.04623	ATP1A2, ATP1A1	266	3	16929	42.4286	1	0.89442	0.88493
GOTERM_BP_DIRECT	GO:2001027~negative regulation of endothelial cell chemotaxis	2	0.68966	0.04623	HRG, THBS1	266	3	16929	42.4286	1	0.89442	0.88493
GOTERM_BP_DIRECT	GO:0070536~protein K63-linked deubiquitination	3	1.03448	0.04977	USP8, USP7, SHMT2	266	23	16929	8.30124	1	0.94523	0.93521
GOTERM_BP_DIRECT	GO:0032956~regulation of actin cytoskeleton organization	4	1.37931	0.05011	ROCK1, FSCN1, HRG, BAIAP2	266	53	16929	4.80323	1	0.94523	0.93521
GOTERM_BP_DIRECT	GO:0042167~heme catabolic process	2	0.68966	0.06116	BLVRA, HMOX2	266	4	16929	31.8214	1	1	0.99005
GOTERM_BP_DIRECT	GO:0035089~establishment of apical/basal cell polarity	2	0.68966	0.06116	FSCN1, SCRIB	266	4	16929	31.8214	1	1	0.99005
GOTERM_BP_DIRECT	GO:0045684~positive regulation of epidermis development	2	0.68966	0.06116	KRT2, KRT10	266	4	16929	31.8214	1	1	0.99005
GOTERM_BP_DIRECT	GO:1900223~positive regulation of beta-amyloid clearance	2	0.68966	0.06116	ROCK1, APOE	266	4	16929	31.8214	1	1	0.99005
GOTERM_BP_DIRECT	GO:0010248~establishment or maintenance of transmembrane electrochemical gradient	2	0.68966	0.06116	ATP1A2, ATP1A1	266	4	16929	31.8214	1	1	0.99005
GOTERM_BP_DIRECT	GO:0002830~positive regulation of type 2 immune response	2	0.68966	0.06116	CD74, IDO1	266	4	16929	31.8214	1	1	0.99005
GOTERM_BP_DIRECT	GO:0051838~cytolysis by host of symbiont cells	2	0.68966	0.06116	HRG, F2	266	4	16929	31.8214	1	1	0.99005
GOTERM_BP_DIRECT	GO:0010751~negative regulation of nitric oxide mediated signal transduction	2	0.68966	0.06116	ATP2B4, THBS1	266	4	16929	31.8214	1	1	0.99005
GOTERM_BP_DIRECT	GO:0002503~peptide antigen assembly with MHC class II protein complex	3	1.03448	0.06203	BOLA-DQB, BOLA-DRA, B2M	266	26	16929	7.34341	1	1	0.99005
GOTERM_BP_DIRECT	GO:0046718~viral entry into host cell	3	1.03448	0.06203	ITGB1, CD81, CTSB	266	26	16929	7.34341	1	1	0.99005
GOTERM_BP_DIRECT	GO:0009408~response to heat	3	1.03448	0.06203	PSIP1, PLG, CSN2	266	26	16929	7.34341	1	1	0.99005
GOTERM_BP_DIRECT	GO:0031647~regulation of protein stability	4	1.37931	0.06241	USP8, USP7, CD81, USP4	266	58	16929	4.38916	1	1	0.99005
GOTERM_BP_DIRECT	GO:0045931~positive regulation of mitotic cell cycle	3	1.03448	0.0707	APP, DUSP3, PKN2	266	28	16929	6.81888	1	1	0.99005
GOTERM_BP_DIRECT	GO:0019915~lipid storage	3	1.03448	0.0707	APOA1, PLIN2, GBA1	266	28	16929	6.81888	1	1	0.99005
GOTERM_BP_DIRECT	GO:0032436~positive regulation of proteasomal ubiquitin-dependent protein catabolic process	4	1.37931	0.07316	CSNK1D, GBA1, CLU	266	62	16929	4.10599	1	1	0.99005
GOTERM_BP_DIRECT	GO:0016925~protein sumoylation	3	1.03448	0.07518	TRIM28, RANGAP1, SAE1	266	29	16929	6.58374	1	1	0.99005
GOTERM_BP_DIRECT	GO:1903494~response to dehydroepiandrosterone	2	0.68966	0.07586	CSN3, CSN2	266	5	16929	25.4571	1	1	0.99005
GOTERM_BP_DIRECT	GO:0030300~regulation of intestinal cholesterol absorption	2	0.68966	0.07586	APOA1, APOA4	266	5	16929	25.4571	1	1	0.99005
GOTERM_BP_DIRECT	GO:1990034~calcium ion export from cell	2	0.68966	0.07586	ATP2B3, ATP2B1	266	5	16929	25.4571	1	1	0.99005
GOTERM_BP_DIRECT	GO:0006271~DNA strand elongation involved in DNA replication	2	0.68966	0.07586	MCM7, MCM3	266	5	16929	25.4571	1	1	0.99005
GOTERM_BP_DIRECT	GO:1902975~mitotic DNA replication initiation	2	0.68966	0.07586	MCM3, MCM2	266	5	16929	25.4571	1	1	0.99005
GOTERM_BP_DIRECT	GO:1903496~response to 11-deoxycorticosterone	2	0.68966	0.07586	CSN3, CSN2	266	5	16929	25.4571	1	1	0.99005
GOTERM_BP_DIRECT	GO:0042158~lipoprotein biosynthetic process	2	0.68966	0.07586	APOA1, APOE	266	5	16929	25.4571	1	1	0.99005
GOTERM_BP_DIRECT	GO:0015833~peptide transport	2	0.68966	0.07586	TAP2, TAP1	266	5	16929	25.4571	1	1	0.99005
GOTERM_BP_DIRECT	GO:0060341~regulation of cellular localization	2	0.68966	0.07586	PARD6B, PARD6A	266	5	16929	25.4571	1	1	0.99005
GOTERM_BP_DIRECT	GO:0009649~entrainment of circadian clock	2	0.68966	0.07586	GNAQ, GNA11	266	5	16929	25.4571	1	1	0.99005
GOTERM_BP_DIRECT	GO:0051099~positive regulation of binding	2	0.68966	0.07586	CLIC2, S100A10	266	5	16929	25.4571	1	1	0.99005
GOTERM_BP_DIRECT	GO:0010466~negative regulation of peptidase activity	2	0.68966	0.07586	ECM1, CTSB	266	5	16929	25.4571	1	1	0.99005
GOTERM_BP_DIRECT	GO:0006102~isocitrate metabolic process	2	0.68966	0.07586	IDH2, IDH3A	266	5	16929	25.4571	1	1	0.99005
GOTERM_BP_DIRECT	GO:0071681~cellular response to indole-3-methanol	2	0.68966	0.07586	JUP, CDH1	266	5	16929	25.4571	1	1	0.99005
GOTERM_BP_DIRECT	GO:0001578~microtubule bundle formation	3	1.03448	0.08439	MAP2, KIF20A, MARK4	266	31	16929	6.15899	1	1	0.99005
GOTERM_BP_DIRECT	GO:0008542~visual learning	3	1.03448	0.08439	ITGB1, APP, ATP1A2	266	31	16929	6.15899	1	1	0.99005
GOTERM_BP_DIRECT	GO:0042307~positive regulation of protein import into nucleus	3	1.03448	0.08912	JUP, TRIM28, CDH1	266	32	16929	5.96652	1	1	0.99005
GOTERM_BP_DIRECT	GO:0086073~bundle of His cell-Purkinje myocyte adhesion involved in cell communication	2	0.68966	0.09033	JUP, CTNNA3	266	6	16929	21.2143	1	1	0.99005
GOTERM_BP_DIRECT	GO:0032489~regulation of Cdc42 protein signal transduction	2	0.68966	0.09033	APOA1, APOE	266	6	16929	21.2143	1	1	0.99005
GOTERM_BP_DIRECT	GO:2000369~regulation of clathrin-dependent endocytosis	2	0.68966	0.09033	HIP1R, AAK1	266	6	16929	21.2143	1	1	0.99005
GOTERM_BP_DIRECT	GO:1902806~regulation of cell cycle G1/S phase transition	2	0.68966	0.09033	ATP2B4, CIRBP	266	6	16929	21.2143	1	1	0.99005
GOTERM_BP_DIRECT	GO:0009143~nucleoside triphosphate catabolic process	2	0.68966	0.09033	ENPP1, ENPP3	266	6	16929	21.2143	1	1	0.99005
GOTERM_BP_DIRECT	GO:0034115~negative regulation of heterotypic cell-cell adhesion	2	0.68966	0.09033	IL1RN, APOA1	266	6	16929	21.2143	1	1	0.99005
GOTERM_BP_DIRECT	GO:0035520~monoubiquitinated protein deubiquitination	2	0.68966	0.09033	USP15, USP7	266	6	16929	21.2143	1	1	0.99005
GOTERM_BP_DIRECT	GO:0051256~mitotic spindle midzone assembly	2	0.68966	0.09033	RACGAP1, KIF4A	266	6	16929	21.2143	1	1	0.99005
GOTERM_BP_DIRECT	GO:0070585~protein localization to mitochondrion	2	0.68966	0.09033	MTCH2, ISG15	266	6	16929	21.2143	1	1	0.99005
GOTERM_BP_DIRECT	GO:0010629~negative regulation of gene expression	7	2.41379	0.09321	APP, PDCD10, ATP2B4, ADAM10, APOE, PARP9, XDH	266	199	16929	2.23869	1	1	0.99005
GOTERM_BP_DIRECT	GO:0018105~peptidyl-serine phosphorylation	6	2.06897	0.0947	PRKCH, ROCK1, STK39, CSNK1D, PKN2	266	154	16929	2.47959	1	1	0.99005
GOTERM_BP_DIRECT	GO:0098609~cell-cell adhesion	5	1.72414	0.0967	TJP1, LGALS1, CTNNA3, SCRIB, CTNNA2	266	111	16929	2.8668	1	1	0.99005

GOTERM_BP_DIRECT	GO:0031623~receptor internalization	3	1.03448	0.09881	ITGB1, TFRC, CD81	266	34	16929	5.61555	1	1	0.99005

Category	Term	Count	%	PValue	Genes	List Total	Pop Hits	Pop Total	Fold Enricl	Bonferron	Benjamini	FDR
GOTERM_CC_DIRECT	GO:0005615~extracellular space	54	18.6207	4.20E-12	APP, IL1RN, ECM1, B4GALT1, TINAGL1, MSTN, PLOD3, CSN3, GBA1, CSN2, CLU, LGALS1, FCGRT, DAG1, ENPP1, TIMP3, FAM3C, GUSB, BOLA, CTSB, CHID1, BOLA-NC1, AHSG, ARG1, GPX3, F2, RBP4, FAM20C, SERPING1, SERPINB12, SERPINC1, SEMA3G, PLG, THBS1, VTN, TTR, S100A16, C9, GPC1, S100A14, APOE, GC, LO	279	1399	20801	2.87777	1.41E-09	1.41E-09	1.25E-09
GOTERM_CC_DIRECT	GO:0005737~cytoplasm	90	31.0345	1.18E-07	APP, CLIC5, THUMPD3, CLU, TUBA1C, LGALS1, HINT2, CDH1, AIFM2, FTH1, DAG1, CLIC2, NUDT14, LMCD1, GTF2I, DUSP3, PRKCH, MME, USP4, DAPK1, ARG1, ADAM10, ARHGEF18, CSNK1D, PARP9, TUBA4A, UCHL5, EML4, ENAH, TUBB2B, TUBB2A, FSCN1, MAPT, PPIB, BLVRA, ARF5, IDO1, MCM2, USP15, GALT, ROCK1,	279	3943	20801	1.70175	3.97E-05	1.98E-05	1.76E-05
GOTERM_CC_DIRECT	GO:0048471~perinuclear region of cytoplasm	22	7.58621	6.87E-07	ITGB1, APP, GSDMA, TFRC, MX1, HIP1R, CSNK1D, RANGAP1, CLU, COPS3, TF, CDH1, MCM3, S100A4, PKN2, S100A14, ENPP3, ATXN10, PPIB, ATP6V0A1, CTSB	279	446	20801	3.67763	2.31E-04	7.71E-05	6.84E-05
GOTERM_CC_DIRECT	GO:0005829~cytosol	68	23.4483	7.84E-06	MCM7, THUMPD3, CHD5, GIMAP4, LIPA, CLU, LGALS1, AIFM2, CLIC2, USP8, USP7, DUSP3, PRKCH, USP4, ARG1, CMBL, RANGAP1, UCHL5, DNAJC3, ENAH, FSCN1, PLIN2, MCM5, S100A4, MAPT, TBC1D23, BLVRA, USP15, SERPINB12, TOMM40, NXN, SHMT2, PIK3R4, HTRA1, STK39, ZC3HAV1, CYTH3, PARD6B, PRDX4, PD	279	2967	20801	1.70872	0.00264	6.60E-04	5.86E-04
GOTERM_CC_DIRECT	GO:0005576~extracellular region	28	9.65517	2.12E-05	APP, MSTN, PON2, HTRA1, CSN3, PLG, CSN2, LAMC1, THBS1, C9, DAG1, TIMP3, ENPP3, B2M, CHID1, F10, AHSG, ISG15, CP, TCN2, MGP, NUCB1, PAEP, VWA1, S100A4, MAPT, HRG, MATN4	279	833	20801	2.50607	0.00711	0.00143	0.00127
GOTERM_CC_DIRECT	GO:0030496~midbody	11	3.7931	3.12E-05	EML4, USP8, RACGAP1, SEPTIN2, MICAL1, KIF4A, CDCA8, PKN2, KIF20A, MARK4, MYH10	279	148	20801	5.54129	0.01045	0.00175	0.00155
GOTERM_CC_DIRECT	GO:0019897~extrinsic component of plasma membrane	5	1.72414	6.48E-05	USP8, STOML2, AAK1, PLG, S100A10	279	17	20801	21.9281	0.0216	0.00255	0.00227
GOTERM_CC_DIRECT	GO:0009986~cell surface	17	5.86207	6.48E-05	ITGB1, CD74, APP, SDC4, TRPC4, ADAM10, APOA4, KRT10, THBS1, LRP6, GPC1, ENPP1, BOLA-DRA, GPC4, GUSB, HRG, S100A10	279	384	20801	3.30064	0.02161	0.00255	0.00227
GOTERM_CC_DIRECT	GO:0045121~membrane raft	10	3.44828	6.82E-05	ITGB1, APP, STOML2, MME, GPC1, ATP1A4, ATP2B4, DAG1, ATP1A1, S100A10	279	131	20801	5.69126	0.02273	0.00255	0.00227
GOTERM_CC_DIRECT	GO:0005912~adherens junction	9	3.10345	2.01E-04	CYTH3, NIBAN2, JUP, CDH1, RDX, ADAM10, CTNNA3, SCRIB, CTNNA2	279	119	20801	5.63866	0.06539	0.00573	0.00508
GOTERM_CC_DIRECT	GO:0005874~microtubule	12	4.13793	2.01E-04	EML4, TUBA1C, TUBA3E, TUBB2B, TUBB2A, MAP2, MX2, MX1, MAPT, KIF20A, TUBB4B, TUBA4A	279	221	20801	4.04827	0.06542	0.00573	0.00508
GOTERM_CC_DIRECT	GO:0005794~Golgi apparatus	24	8.27586	2.14E-04	CD74, CLIC5, APP, B4GALT1, GALT, AHSG, ADAM10, CSN3, CSNK1D, GBA1, CSN2, HS3ST1, LRP6, CDH1, PDCD10, NUCB1, FAM20C, AP3S2, FAM3C, TBC1D23, KIF20A, APOE, ATP6V0A1	279	754	20801	2.37312	0.06965	0.00573	0.00508
GOTERM_CC_DIRECT	GO:0009897~external side of plasma membrane	16	5.51724	2.21E-04	ITGB1, CD74, BOLA-NC1, B4GALT1, TFRC, ATP6AP2, F2, THBS1, TCN2, FCGRT, DAG1, ENPP3, GPC4, LOC616942, BOLA, CTSB	279	384	20801	3.10648	0.07175	0.00573	0.00508
GOTERM_CC_DIRECT	GO:0042555~MCM complex	4	1.37931	2.64E-04	MCM7, MCM3, MCM5, MCM2	279	10	20801	29.8222	0.08524	0.00636	0.00565
GOTERM_CC_DIRECT	GO:0032154~cleavage furrow	6	2.06897	2.92E-04	RACGAP1, SEPTIN2, RDX, PKN2, KIF20A, MYH10	279	44	20801	10.1667	0.09362	0.00655	0.00581
GOTERM_CC_DIRECT	GO:0071162~CMG complex	4	1.37931	3.60E-04	MCM7, MCM3, MCM5, MCM2	279	11	20801	27.1111	0.11424	0.00714	0.00633
GOTERM_CC_DIRECT	GO:0005905~clathrin-coated pit	6	2.06897	3.60E-04	APP, TF, TFRC, HIP1R, AAK1, AP2M1	279	46	20801	9.72464	0.11432	0.00714	0.00633
GOTERM_CC_DIRECT	GO:0030175~filopodium	6	2.06897	9.76E-04	ITGB1, B4GALT1, RDX, FSCN1, DAG1, BAIAP2	279	57	20801	7.84795	0.28051	0.01828	0.01622
GOTERM_CC_DIRECT	GO:0001772~immunological synapse	5	1.72414	0.00131	STOML2, CD81, SCRIB, BOLA-DRA, ATP2B1	279	36	20801	10.3549	0.35784	0.0233	0.02067
GOTERM_CC_DIRECT	GO:0035748~myelin sheath abaxonal region	3	1.03448	0.00173	ITGB1, EXOC4, SCRIB	279	5	20801	44.7333	0.44264	0.0292	0.02591
GOTERM_CC_DIRECT	GO:0034364~high-density lipoprotein particle	4	1.37931	0.00195	SAA3, APOA1, APOA4, APOE	279	19	20801	15.6959	0.48249	0.02991	0.02654
GOTERM_CC_DIRECT	GO:0001533~cornified envelope	4	1.37931	0.00195	JUP, KRT2, KRT1, KRT10	279	19	20801	15.6959	0.48249	0.02991	0.02654
GOTERM_CC_DIRECT	GO:0010494~cytoplasmic stress granule	6	2.06897	0.0023	YTHDF2, YTHDF3, ROCK1, CIRBP, PABPC1L, LSM14A	279	69	20801	6.48309	0.54005	0.03373	0.02993
GOTERM_CC_DIRECT	GO:0030315~T-tubule	4	1.37931	0.00263	RTN2, ATP2B4, ATP1A2, ATP1A1	279	21	20801	14.2011	0.58802	0.0369	0.03274
GOTERM_CC_DIRECT	GO:0031090~organelle membrane	7	2.41379	0.00331	MCM7, MCM3, RPA1, MCM5, ATP1A2, ATP1A1, MCM2	279	108	20801	4.8323	0.67323	0.04467	0.03963
GOTERM_CC_DIRECT	GO:0042470~melanosome	6	2.06897	0.00371	ITGB1, TFRC, ATP1A1, PPIB, ATP6V0A1, CTSB	279	77	20801	5.80952	0.7145	0.04812	0.0427
GOTERM_CC_DIRECT	GO:0097197~tetraspanin-enriched microdomain	3	1.03448	0.00473	CD81, ADAM10, SAMHD1	279	8	20801	27.9583	0.79735	0.05898	0.05233
GOTERM_CC_DIRECT	GO:0005890~sodium:potassium-exchanging ATPase complex	3	1.03448	0.00602	ATP1A4, ATP1A2, ATP1A1	279	9	20801	24.8519	0.8694	0.07248	0.06431
GOTERM_CC_DIRECT	GO:0016324~apical plasma membrane	10	3.44828	0.00634	CLIC5, TF, PARD6B, PARD6A, RDX, STK39, HIP1R, ATP1A1, ENPP3, CTSB	279	248	20801	3.00627	0.88293	0.07373	0.06542
GOTERM_CC_DIRECT	GO:0016323~basolateral plasma membrane	8	2.75862	0.00686	B4GALT1, TFRC, TRPC4, CD81, STK39, SCRIB, ATP1A1, ATP2B1	279	165	20801	3.61481	0.90163	0.07703	0.06835
GOTERM_CC_DIRECT	GO:0042627~chylomicron	3	1.03448	0.00746	APOA1, APOA4, APOE	279	10	20801	22.3667	0.91986	0.08112	0.07197
GOTERM_CC_DIRECT	GO:0015629~actin cytoskeleton	8	2.75862	0.00853	STOML2, ZNF185, SCIN, DAPK1, SEPTIN2, FSCN1, CTNNA2, BAIAP2	279	172	20801	3.4677	0.94422	0.08981	0.07969
GOTERM_CC_DIRECT	GO:0015630~microtubule cytoskeleton	6	2.06897	0.00898	TUBB2B, TUBB2A, SHMT2, SEPTIN2, PIK3R4, TUBB4B	279	95	20801	4.70877	0.95209	0.09166	0.08133
GOTERM_CC_DIRECT	GO:0030027~lamellipodium	7	2.41379	0.00934	ITGB1, ROCK1, CDH1, FSCN1, DAG1, PKN2, MYH10	279	134	20801	3.89469	0.95772	0.09227	0.08186
GOTERM_CC_DIRECT	GO:0042613~MHC class II protein complex	4	1.37931	0.00958	CD74, BOLA-DQB, BOLA-DRA, B2M	279	33	20801	9.03704	0.96103	0.09227	0.08186
GOTERM_CC_DIRECT	GO:0098978~glutamatergic synapse	9	3.10345	0.01216	ITGB1, NAPB, PRKAR2B, ADAM10, ATP2B3, PLG, ATP2B1, APOE, GPC4	279	229	20801	2.93013	0.98383	0.11117	0.09864
GOTERM_CC_DIRECT	GO:0005856~cytoskeleton	11	3.7931	0.01221	ENAH, STOML2, JUP, ROCK1, MICAL1, RDX, HIP1R, DAG1, CTNNA3, CTNNA2, BAIAP2	279	324	20801	2.53121	0.98406	0.11117	0.09864
GOTERM_CC_DIRECT	GO:0045171~intercellular bridge	5	1.72414	0.01369	SEPTIN2, MICAL1, CDCA8, KIF20A, TUBB4B	279	69	20801	5.40258	0.99039	0.12139	0.1077
GOTERM_CC_DIRECT	GO:0031838~haptoglobin-hemoglobin complex	3	1.03448	0.01457	HBE2, HBE1, HBE4	279	14	20801	15.9762	0.99288	0.12588	0.11168
GOTERM_CC_DIRECT	GO:0005833~hemoglobin complex	3	1.03448	0.01666	HBE2, HBE1, HBE4	279	15	20801	14.9111	0.99653	0.1376	0.12209
GOTERM_CC_DIRECT	GO:0005938~cell cortex	6	2.06897	0.01674	CLIC5, PARD6B, SCIN, PARD6A, SEPTIN2, MYH10	279	111	20801	4.03003	0.99662	0.1376	0.12209
GOTERM_CC_DIRECT	GO:0005604~basement membrane	5	1.72414	0.0173	DAG1, VWA1, TIMP3, LAMC1, AGRN	279	74	20801	5.03754	0.99721	0.13885	0.12319
GOTERM_CC_DIRECT	GO:0034361~very-low-density lipoprotein particle	3	1.03448	0.01888	APOA1, APOA4, APOE	279	16	20801	13.9792	0.99837	0.14794	0.13126
GOTERM_CC_DIRECT	GO:0043198~dendritic shaft	3	1.03448	0.02121	APP, CYTH2, PRKAR2B	279	17	20801	13.1569	0.99927	0.15729	0.13955
GOTERM_CC_DIRECT	GO:0005802~trans-Golgi network	7	2.41379	0.02135	CHID1, MME, CDH1, PLOD3, GBA1, ATP11A, TBC1D23	279	161	20801	3.24155	0.99931	0.15729	0.13955
GOTERM_CC_DIRECT	GO:0016020~membrane	24	8.27586	0.02147	ITGB1, NAPB, MTCH2, TRPC5, PRKCH, CD81, MX2, GLYCAM1, MX1, PIK3R4, ATP2A1, SCRIB, ACSL4, LAMC1, BAIAP2, ABHD12, PARD6B, PARD6A, GNAQ, GNA11, DAG1, NUCB1, PLIN2, CLIC2	279	1093	20801	1.63708	0.99933	0.15729	0.13955
GOTERM_CC_DIRECT	GO:0030136~clathrin-coated vesicle	4	1.37931	0.02485	APP, HIP1R, AAK1, ADAM10	279	47	20801	6.34515	0.99979	0.17821	0.15811
GOTERM_CC_DIRECT	GO:1902560~GMP reductase complex	2	0.68966	0.02655	GMPR2, GMPR	279	2	20801	74.5556	0.99988	0.18641	0.16539
GOTERM_CC_DIRECT	GO:0043197~dendritic spine	5	1.72414	0.02825	ITGB1, APP, CYTH2, USP8, PRKAR2B	279	86	20801	4.33463	0.99994	0.19426	0.17235
GOTERM_CC_DIRECT	GO:0005911~cell-cell junction	6	2.06897	0.03145	APP, PRKCH, TRPC4, CDH1, FSCN1, DAG1	279	131	20801	3.41476	0.99998	0.212	0.1881
GOTERM_CC_DIRECT	GO:0055037~recycling endosome	5	1.72414	0.03616	ITGB1, APP, TF, TFRC, ATP11A	279	93	20801	4.00836	1	0.23895	0.21201
GOTERM_CC_DIRECT	GO:0043231~intracellular membrane-bounded organelle	9	3.10345	0.03799	CLIC5, PDHA1, ATP2B4, ATP2B3, AP3S2, ATP2B1, PPIB, LIPA, CLU	279	285	20801	2.35439	1	0.24367	0.2162
GOTERM_CC_DIRECT	GO:0005739~mitochondrion	21	7.24138	0.03913	CLIC5, MTCH2, USP15, RRM1, TXNRD2, SHMT2, IDH2, HIP1R, ATP2A1, ACSL4, SDHA, HSPE1, CLU, GATD3, ALDH4A1, HINT2, AIFM2, OXCT1, PGAM5, DCPS, IDH3A	279	977	20801	1.60252	1	0.24367	0.2162
GOTERM_CC_DIRECT	GO:0042825~TAP complex	2	0.68966	0.03956	TAP2, TAP1	279	3	20801	49.7037	1	0.24367	0.2162
GOTERM_CC_DIRECT	GO:0005923~bicellular tight junction	5	1.72414	0.03991	CYTH3, TJP1, CYTH2, PARD6B, PARD6A	279	96	20801	3.8831	1	0.24367	0.2162
GOTERM_CC_DIRECT	GO:0051233~spindle midzone	3	1.03448	0.04049	APP, RACGAP1, CDCA8	279	24	20801	9.31944	1	0.24367	0.2162
GOTERM_CC_DIRECT	GO:0005788~endoplasmic reticulum lumen	4	1.37931	0.04821	DNAJC3, OS9, SELENOF, PPIB	279	61	20801	4.88889	1	0.28502	0.25288
GOTERM_CC_DIRECT	GO:0001726~ruffle	4	1.37931	0.05631	CYTH3, ROCK1, FSCN1, BAIAP2	279	65	20801	4.58803	1	0.3272	0.29031
GOTERM_CC_DIRECT	GO:0070062~extracellular exosome	4	1.37931	0.05843	NAGLU, TFRC, CD81, APOE	279	66	20801	4.51852	1	0.33377	0.29613
GOTERM_CC_DIRECT	GO:0030424~axon	7	2.41379	0.06324	APP, MME, ATP6AP2, ADAM10, ATP1A1, MAPT, CTNNA2	279	210	20801	2.48519	1	0.35367	0.31379
GOTERM_CC_DIRECT	GO:1990712~HFE-transferrin receptor complex	2	0.68966	0.06507	TF, TFRC	279	5	20801	29.8222	1	0.35367	0.31379
GOTERM_CC_DIRECT	GO:0042824~MHC class I peptide loading complex	2	0.68966	0.06507	TAP2, TAP1	279	5	20801	29.8222	1	0.35367	0.31379
GOTERM_CC_DIRECT	GO:0016342~catenin complex	3	1.03448	0.06791	JUP, CDH1, CTNNA2	279	32	20801	6.98958	1	0.36327	0.32231
GOTERM_CC_DIRECT	GO:0005764~lysosome	7	2.41379	0.0706	CHID1, CD74, TINAGL1, NAGLU, GBA1, GUSB, CTSB	279	216	20801	2.41615	1	0.37175	0.32983
GOTERM_CC_DIRECT	GO:0031410~cytoplasmic vesicle	7	2.41379	0.07689	CLIC5, ATP6AP2, LOC513659, FAM3C, TBC1D23, AP2M1, GBP4	279	221	20801	2.36149	1	0.39605	0.3514
GOTERM_CC_DIRECT	GO:0031232~extrinsic component of external side of plasma membrane	2	0.68966	0.07757	TF, PLG	279	6	20801	24.8519	1	0.39605	0.3514
GOTERM_CC_DIRECT	GO:0000932~P-body	4	1.37931	0.08414	YTHDF2, YTHDF3, DCPS, LSM14A	279	77	20801	3.87302	1	0.42322	0.3755
GOTERM_CC_DIRECT	GO:0014069~postsynaptic density	5	1.72414	0.08694	USP8, ADAM10, SCRIB, ATP1A1, RTN4	279	125	20801	2.98222	1	0.43086	0.38228
GOTERM_CC_DIRECT	GO:0034366~spherical high-density lipoprotein particle	2	0.68966	0.0899	APOA1, CLU	279	7	20801	21.3016	1	0.43906	0.38955
GOTERM_CC_DIRECT	GO:0045202~synapse	9	3.10345	0.09446	SDCBP, MME, GPC1, GNAQ, GNA11, EXOC4, GPC4, AGRN, LRP6	279	346	20801	1.93931	1	0.45478	0.4035

Category	Term	Count	%	PValue	Genes	List Total	Pop Hits	Pop Total	Fold Enricl	Bonferron	Benjamini	FDR
GOTERM_MF_DIRECT	GO:0005515~protein binding	20	6.89655	2.51E-10	B4GALT1, JUP, AHSG, ADAM10, CSN3, PLG, ATP1A2, ATP1A1, CSN2, F2, AP2A2, TUBA4A, DNAJC3, CYTH2, OS9, PRKAR2B, DAG1, PAEP, MAPT, PPIB	261	216	18413	6.53221	1.15E-07	1.15E-07	1.08E-07
GOTERM_MF_DIRECT	GO:0043236~laminin binding	8	2.75862	1.78E-09	ITGB1, ECM1, LGALS1, TINAGL1, GPC1, DAG1, AGRN, THBS1	261	17	18413	33.199	8.12E-07	4.06E-07	3.84E-07
GOTERM_MF_DIRECT	GO:0005525~GTP binding	18	6.2069	4.23E-05	DAPK1, SEPTIN2, MX2, MX1, LOC513659, AK4, GIMAP4, SAMHD1, TUBB4B, GNL1, TUBA4A, TUBA1C, TUBA3E, TUBB2B, TUBB2A, GNA11, GBP4, ARF5	261	390	18413	3.25606	0.01911	0.00615	0.00581
GOTERM_MF_DIRECT	GO:0005391~sodium:potassium-exchanging ATPase activity	4	1.37931	5.39E-05	ATP1A4, ATP1A3, ATP1A2, ATP1A1	261	6	18413	47.0319	0.0243	0.00615	0.00581
GOTERM_MF_DIRECT	GO:0046872~metal ion binding	42	14.4828	2.01E-04	ITGB1, ROCK1, PON2, GMPR2, ATP1A4, GMPR, CHD5, ATP1A3, CSN2, ATP1A1, ZC3HAV1, TUBA1C, TUBA3E, MAN2A2, MICAL1, GNA11, ENPP1, TIMP3, ENPP3, NUDT14, HMOX2, ZC3H15, ZNF185, USP4, HBE2, HBE1, RPA1, HBE4, ATP2B4, ADAM10, ATP2B3, ATP2B1, TUBB4B, HSPE1, TUBA4A, TUBB2B, TCN2, ASPH, ST	261	1630	18413	1.8178	0.08774	0.01512	0.01429
GOTERM_MF_DIRECT	GO:0005388~calcium-transporting ATPase activity	4	1.37931	2.20E-04	ATP2B4, ATP2B3, ATP2A1, ATP2B1	261	9	18413	31.3546	0.09527	0.01512	0.01429
GOTERM_MF_DIRECT	GO:0003924~GTPase activity	15	5.17241	2.32E-04	SEPTIN2, MX2, MX1, LOC513659, TUBB4B, GNL1, TUBA4A, TUBA1C, TUBA3E, TUBB2B, TUBB2A, GNAQ, GNA11, GBP4, ARF5	261	326	18413	3.24607	0.10046	0.01512	0.01429
GOTERM_MF_DIRECT	GO:0042803~protein homodimerization activity	21	7.24138	2.77E-04	PRMT8, JUP, MSTN, MME, TFRC, TXNRD2, SHMT2, HIP1R, ADAM10, APOA1, ATP2A1, APOA4, CSN2, LRP6, JCHAIN, S100A16, PDCD10, ENPP1, APOE, XDH, S100A10	261	588	18413	2.51957	0.1188	0.01521	0.01437
GOTERM_MF_DIRECT	GO:0016787~hydrolase activity	10	3.44828	3.00E-04	TUBA1C, TUBA3E, HINT2, NAGLU, ATP1A3, CMBL, ATP1A1, ENPP3, DCPS, TUBA4A	261	151	18413	4.67205	0.12794	0.01521	0.01437
GOTERM_MF_DIRECT	GO:0042802~identical protein binding	30	10.3448	4.12E-04	APP, USP15, B4GALT1, SDC4, MSTN, SERPINC1, PON2, HTRA1, CSN3, SAMHD1, PRDX4, TTR, CDH1, APOE, DCPS, NUDT14, RRM1, DAPK1, USP4, GPX3, GLYCAM1, MX1, APOA1, BAIAP2, ALDH4A1, PRPF6, PAEP, VWA1, S100A4, FAM98B	261	1047	18413	2.02143	0.17142	0.0188	0.01777
GOTERM_MF_DIRECT	GO:0005200~structural constituent of cytoskeleton	6	2.06897	5.63E-04	TUBA1C, TUBA3E, TUBB2B, TUBB2A, TUBB4B, TUBA4A	261	48	18413	8.81849	0.22661	0.02335	0.02207
GOTERM_MF_DIRECT	GO:0005524~ATP binding	35	12.069	0.00169	MCM7, ROCK1, PIK3R4, ATP1A4, STK39, CHD5, ATP1A3, AK4, ATP1A2, ATP2A1, ABCB8, ATP1A1, ENPP1, MARK4, MYH10, RRM1, PRKCH, DAPK1, ATP2B4, TAP2, NME3, TAP1, ATP2B3, CSNK1D, ATP2B1, ATP11A, HSPE1, KIF4A, AAK1, MCM3, PKN2, MCM5, KIF20A, MCM2	261	1419	18413	1.74008	0.53801	0.06322	0.05976
GOTERM_MF_DIRECT	GO:0060228~phosphatidylcholine-sterol O-acyltransferase activator activity	3	1.03448	0.00193	APOA1, APOA4, APOE	261	5	18413	42.3287	0.58583	0.06322	0.05976
GOTERM_MF_DIRECT	GO:0016209~antioxidant activity	4	1.37931	0.00194	PRDX4, APOA4, APOE, CSN2	261	18	18413	15.6773	0.58769	0.06322	0.05976
GOTERM_MF_DIRECT	GO:0008201~heparin binding	8	2.75862	0.00284	APP, MSTN, GPNMB, SERPINC1, APOE, HRG, F2, THBS1	261	133	18413	4.24348	0.72685	0.08639	0.08166
GOTERM_MF_DIRECT	GO:0030280~structural constituent of epidermis	3	1.03448	0.00398	KRT2, KRT1, KRT10	261	7	18413	30.2348	0.83776	0.096	0.09073
GOTERM_MF_DIRECT	GO:0031721~hemoglobin alpha binding	3	1.03448	0.00398	HBE2, HBE1, HBE4	261	7	18413	30.2348	0.83776	0.096	0.09073
GOTERM_MF_DIRECT	GO:0003678~DNA helicase activity	5	1.72414	0.00398	MCM7, MCM3, CHD5, MCM5, MCM2	261	46	18413	7.66825	0.83777	0.096	0.09073
GOTERM_MF_DIRECT	GO:0005509~calcium ion binding	19	6.55172	0.0041	F10, ATP2A1, F2, THBS1, SCIN, CRACR2B, S100A16, CDH1, MGP, DAG1, NUCB1, S100A4, S100A14, ENPP3, AGRN, MATN4, CAPS, S100A10, MATN2	261	635	18413	2.11088	0.84633	0.096	0.09073
GOTERM_MF_DIRECT	GO:0016887~ATPase activity	12	4.13793	0.00421	MCM7, MCM3, ATP2B4, CHD5, ATP2A1, MCM5, ATP1A2, ATP1A1, ATP2B1, KIF20A, HSPE1, MCM2	261	305	18413	2.77565	0.85398	0.096	0.09073
GOTERM_MF_DIRECT	GO:0051087~chaperone binding	6	2.06897	0.00549	DNAJC3, PLG, ATP1A2, ATP1A1, HSPE1, CP	261	80	18413	5.29109	0.91885	0.11926	0.11273
GOTERM_MF_DIRECT	GO:0016491~oxidoreductase activity	8	2.75862	0.00655	PRDX4, AIFM2, TXNRD2, MCM3, SELENOF, SDHA, XDH, CP	261	155	18413	3.64118	0.95004	0.13279	0.12551
GOTERM_MF_DIRECT	GO:0030247~polysaccharide binding	3	1.03448	0.0067	VTN, ENPP1, BOLA-DRA	261	9	18413	23.516	0.95332	0.13279	0.12551
GOTERM_MF_DIRECT	GO:0004869~cysteine-type endopeptidase inhibitor activity	4	1.37931	0.00702	SERPINB12, AHSG, CSN2, HRG	261	28	18413	10.0783	0.95982	0.13346	0.12614
GOTERM_MF_DIRECT	GO:0019829~cation-transporting ATPase activity	3	1.03448	0.00829	ATP2B4, ATP2B3, ATP2B1	261	10	18413	21.1644	0.97758	0.14982	0.1416
GOTERM_MF_DIRECT	GO:0045296~cadherin binding	5	1.72414	0.00854	JUP, TRPC4, CDH1, CTNNA3, CTNNA2	261	57	18413	6.18841	0.98	0.14982	0.1416
GOTERM_MF_DIRECT	GO:0003697~single-stranded DNA binding	6	2.06897	0.00897	MCM7, MCM3, MCM5, SAMHD1, JCHAIN, MCM2	261	90	18413	4.70319	0.98355	0.15145	0.14314
GOTERM_MF_DIRECT	GO:0004843~thiol-dependent ubiquitin-specific protease activity	6	2.06897	0.01025	USP8, USP15, USP7, USP4, USP39, UCHL5	261	93	18413	4.55148	0.99089	0.16694	0.15779
GOTERM_MF_DIRECT	GO:0004867~serine-type endopeptidase inhibitor activity	7	2.41379	0.01239	APP, SERPINB12, SERPIND1, SERPINC1, SERPING1, HRG, AGT	261	135	18413	3.65804	0.99661	0.1932	0.18261
GOTERM_MF_DIRECT	GO:0048306~calcium-dependent protein binding	5	1.72414	0.01273	S100A16, ATP2B4, S100A4, S100A14, S100A10	261	64	18413	5.51155	0.9971	0.1932	0.18261
GOTERM_MF_DIRECT	GO:0004674~protein serine/threonine kinase activity	11	3.7931	0.0135	PRKCH, ROCK1, DAPK1, STK39, PIK3R4, AAK1, FAM20C, CSNK1D, PKN2, MARK4	261	312	18413	2.48727	0.99797	0.1932	0.18261
GOTERM_MF_DIRECT	GO:0004712~protein serine/threonine/tyrosine kinase activity	10	3.44828	0.01389	PRKCH, ROCK1, DAPK1, STK39, PIK3R4, AAK1, CSNK1D, PKN2, MARK4	261	267	18413	2.64224	0.9983	0.1932	0.18261
GOTERM_MF_DIRECT	GO:0070679~inositol 1,4,5 trisphosphate binding	3	1.03448	0.01398	CYTH2, TRPC5, TRPC4	261	13	18413	16.2803	0.99837	0.1932	0.18261
GOTERM_MF_DIRECT	GO:0043177~organic acid binding	3	1.03448	0.01616	HBE2, HBE1, HBE4	261	14	18413	15.1174	0.99941	0.21675	0.20487
GOTERM_MF_DIRECT	GO:0008017~microtubule binding	9	3.10345	0.0184	EML4, RACGAP1, MAP2, MX2, KIF4A, MX1, MAPT, KIF20A, MARK4	261	234	18413	2.71338	0.99979	0.2397	0.22656
GOTERM_MF_DIRECT	GO:0002020~protease binding	5	1.72414	0.02161	ITGB1, ECM1, SERPINB12, SERPINC1, TIMP3	261	75	18413	4.70319	0.99995	0.27378	0.25877
GOTERM_MF_DIRECT	GO:0050321~tau-protein kinase activity	3	1.03448	0.02349	CSNK1D, MARK4	261	17	18413	12.4496	0.99998	0.28953	0.27366
GOTERM_MF_DIRECT	GO:0004866~endopeptidase inhibitor activity	3	1.03448	0.02619	AHSG, HRG, UCHL5	261	18	18413	11.758	0.99999	0.29738	0.28108
GOTERM_MF_DIRECT	GO:0005344~oxygen transporter activity	3	1.03448	0.02619	HBE2, HBE1, HBE4	261	18	18413	11.758	0.99999	0.29738	0.28108
GOTERM_MF_DIRECT	GO:0051015~actin filament binding	8	2.75862	0.0267	ZNF185, SCIN, NEBL, HIP1R, FSCN1, CTNNA3, CTNNA2, MYH10	261	205	18413	2.75309	1	0.29738	0.28108
GOTERM_MF_DIRECT	GO:0008289~lipid binding	6	2.06897	0.02777	CYTH2, APOA1, APOA4, APOE, AP2A2, AP2M1	261	120	18413	3.52739	1	0.29738	0.28108
GOTERM_MF_DIRECT	GO:0003920~GMP reductase activity	2	0.68966	0.02804	GMPR2, GMPR	261	2	18413	70.5479	1	0.29738	0.28108
GOTERM_MF_DIRECT	GO:0008556~potassium-transporting ATPase activity	2	0.68966	0.02804	ATP1A3, ATP1A1	261	2	18413	70.5479	1	0.29738	0.28108
GOTERM_MF_DIRECT	GO:0035615~clathrin adaptor activity	3	1.03448	0.029	HIP1R, AP2A2, AP2M1	261	19	18413	11.1391	1	0.30054	0.28406
GOTERM_MF_DIRECT	GO:0019825~oxygen binding	3	1.03448	0.03496	HBE2, HBE1, HBE4	261	21	18413	10.0783	1	0.3466	0.3276
GOTERM_MF_DIRECT	GO:0051787~misfolded protein binding	3	1.03448	0.03496	DNAJC3, DNAJB9, CLU	261	21	18413	10.0783	1	0.3466	0.3276
GOTERM_MF_DIRECT	GO:0005507~copper ion binding	4	1.37931	0.03705	CUTA, GPC1, APOA4, CP	261	52	18413	5.42676	1	0.35942	0.33971
GOTERM_MF_DIRECT	GO:0004601~peroxidase activity	3	1.03448	0.03811	PRDX4, GPX3, GSTA1	261	22	18413	9.62017	1	0.36204	0.34219
GOTERM_MF_DIRECT	GO:0001618~virus receptor activity	3	1.03448	0.04136	ITGB1, CD81, DAG1	261	23	18413	9.2019	1	0.37346	0.35298
GOTERM_MF_DIRECT	GO:0070053~thrombospondin receptor activity	2	0.68966	0.04177	SDC4, F2	261	3	18413	47.0319	1	0.37346	0.35298
GOTERM_MF_DIRECT	GO:1905056~calcium-transporting ATPase activity involved in regulation of presynaptic cytosolic calcium ion concentration	2	0.68966	0.04177	ATP2B3, ATP2B1	261	3	18413	47.0319	1	0.37346	0.35298
GOTERM_MF_DIRECT	GO:0001786~phosphatidylserine binding	4	1.37931	0.04662	SCIN, GSDMA, MME, THBS1	261	57	18413	4.95073	1	0.40884	0.38642

GOTERM_MF_DIRECT	GO:0030246~carbohydrate binding	6	2.06897	0.04865	LGALS1, MAN2A2, OS9, KRT1, MLEC, GUSB	261	140	18413	3.02348	1	0.41855	0.39561
GOTERM_MF_DIRECT	GO:0030165~PDZ domain binding	4	1.37931	0.0529	EXOC4, ATP2B4, ATP2B3, ATP2B1	261	60	18413	4.70319	1	0.42042	0.39737
GOTERM_MF_DIRECT	GO:1990239~steroid hormone binding	2	0.68966	0.0553	ATP1A2, ATP1A1	261	4	18413	35.2739	1	0.42042	0.39737
GOTERM_MF_DIRECT	GO:0015440~peptide-transporting ATPase activity	2	0.68966	0.0553	TAP2, TAP1	261	4	18413	35.2739	1	0.42042	0.39737
GOTERM_MF_DIRECT	GO:0015433~peptide antigen-transporting ATPase activity	2	0.68966	0.0553	TAP2, TAP1	261	4	18413	35.2739	1	0.42042	0.39737
GOTERM_MF_DIRECT	GO:0070051~fibrinogen binding	2	0.68966	0.0553	F2, THBS1	261	4	18413	35.2739	1	0.42042	0.39737
GOTERM_MF_DIRECT	GO:0004528~phosphodiesterase I activity	2	0.68966	0.0553	ENPP1, ENPP3	261	4	18413	35.2739	1	0.42042	0.39737
GOTERM_MF_DIRECT	GO:0023026~MHC class II protein complex binding	3	1.03448	0.05532	BOLA-DQB, BOLA-DRA, B2M	261	27	18413	7.83865	1	0.42042	0.39737
GOTERM_MF_DIRECT	GO:0050660~flavin adenine dinucleotide binding	4	1.37931	0.05729	AIFM2, TXNRD2, SDHA, XDH	261	62	18413	4.55148	1	0.42829	0.40481
GOTERM_MF_DIRECT	GO:0008092~cytoskeletal protein binding	3	1.03448	0.06671	B4GALT1, NEBL, KRT2	261	30	18413	7.05479	1	0.46321	0.43781
GOTERM_MF_DIRECT	GO:0031210~phosphatidylcholine binding	3	1.03448	0.06671	APOA1, APOA4, JCHAIN	261	30	18413	7.05479	1	0.46321	0.43781
GOTERM_MF_DIRECT	GO:0046978~TAP1 binding	2	0.68966	0.06864	TAP2, TAP1	261	5	18413	28.2192	1	0.46321	0.43781
GOTERM_MF_DIRECT	GO:0042287~MHC protein binding	2	0.68966	0.06864	TAP2, TAP1	261	5	18413	28.2192	1	0.46321	0.43781
GOTERM_MF_DIRECT	GO:0004791~thioredoxin-disulfide reductase activity	2	0.68966	0.06864	TXNRD2, NXN	261	5	18413	28.2192	1	0.46321	0.43781
GOTERM_MF_DIRECT	GO:0045545~syndecan binding	2	0.68966	0.06864	SDCBP, GPNMB	261	5	18413	28.2192	1	0.46321	0.43781
GOTERM_MF_DIRECT	GO:0020037~heme binding	6	2.06897	0.06908	HBE2, HBE1, HBE4, HRG, HMOX2, IDO1	261	155	18413	2.73089	1	0.46321	0.43781
GOTERM_MF_DIRECT	GO:0005178~integrin binding	5	1.72414	0.07057	ITGB1, GPNMB, CD81, ISG15, THBS1	261	110	18413	3.20672	1	0.46635	0.44078
GOTERM_MF_DIRECT	GO:0004364~glutathione transferase activity	3	1.03448	0.0747	GSTA5, GSTA2, GSTA1	261	32	18413	6.61386	1	0.48506	0.45847
GOTERM_MF_DIRECT	GO:0019901~protein kinase binding	10	3.44828	0.07872	DNAJC3, RACGAP1, TFRC, MAP2, PDCD10, MICAL1, ADAM10, KIF20A, AP2A2, TUBA4A	261	368	18413	1.91706	1	0.48506	0.45847
GOTERM_MF_DIRECT	GO:0043014~alpha-tubulin binding	3	1.03448	0.0788	EML4, B4GALT1, RACGAP1	261	33	18413	6.41344	1	0.48506	0.45847
GOTERM_MF_DIRECT	GO:0031267~small GTPase binding	7	2.41379	0.08063	PARD6A, PRKCH, ROCK1, MICAL1, EXOC4, PKN2, RANGAP1	261	212	18413	2.32941	1	0.48506	0.45847
GOTERM_MF_DIRECT	GO:0042608~T cell receptor binding	2	0.68966	0.0818	STOML2, BOLA-DRA	261	6	18413	23.516	1	0.48506	0.45847
GOTERM_MF_DIRECT	GO:1990247~N6-methyladenosine-containing RNA binding	2	0.68966	0.0818	YTHDF2, YTHDF3	261	6	18413	23.516	1	0.48506	0.45847
GOTERM_MF_DIRECT	GO:0042289~MHC class II protein binding	2	0.68966	0.0818	CD74, CD81	261	6	18413	23.516	1	0.48506	0.45847
GOTERM_MF_DIRECT	GO:0004175~endopeptidase activity	3	1.03448	0.08297	ADAM10, PLG, CTSB	261	34	18413	6.22481	1	0.48506	0.45847
GOTERM_MF_DIRECT	GO:0005547~phosphatidylinositol-3,4,5-trisphosphate binding	3	1.03448	0.08297	CYTH3, RACGAP1, HIP1R	261	34	18413	6.22481	1	0.48506	0.45847
GOTERM_MF_DIRECT	GO:0017124~SH3 domain binding	4	1.37931	0.08437	ENAH, MICAL1, HIP1R, ADAM10	261	73	18413	3.86564	1	0.48699	0.46029
GOTERM_MF_DIRECT	GO:0008013~beta-catenin binding	4	1.37931	0.08979	TRPC4, CDH1, CTNNA3, CTNNA2	261	75	18413	3.76255	1	0.51179	0.48373
GOTERM_MF_DIRECT	GO:0019001~guanyl nucleotide binding	2	0.68966	0.09477	GNAQ, GNA11	261	7	18413	20.1565	1	0.53313	0.50391
GOTERM_MF_DIRECT	GO:0048487~beta-tubulin binding	3	1.03448	0.09587	EML4, B4GALT1, RACGAP1	261	37	18413	5.7201	1	0.53313	0.50391

Category	Term	Count	%	PValue	Genes	List Total	Pop Hits	Pop Total	Fold Enricl	Bonferron	Benjamini	FDR
KEGG_PATHWAY	bta04145:Phagosome	18	6.2069	3.11E-08	ITGB1, BOLA-NC1, TFRC, TAP2, TAP1, TUBB4B, THBS1, TUBA4A, TUBA1C, TUBA3E, TUBB2B, FCGR2A, TUBB2A, BOLA-DQB, BOLA-DRA, LOC616942, BOLA, ATP6V0A1	189	164	9288	5.39373	7.83E-06	7.83E-06	7.02E-06
KEGG_PATHWAY	bta04961:Endocrine and other factor-regulated calcium reabsorption	10	3.44828	6.88E-07	GNAQ, ATP1A4, ATP2B4, ATP1A3, ATP2B3, ATP1A2, ATP1A1, ATP2B1, AP2A2, AP2M1	189	51	9288	9.63585	1.73E-04	8.67E-05	7.78E-05
KEGG_PATHWAY	bta04978:Mineral absorption	10	3.44828	2.86E-06	TF, FTH1, ATP1A4, ATP2B4, ATP1A3, ATP2B3, ATP1A2, ATP1A1, ATP2B1, HMOX2	189	60	9288	8.19048	7.20E-04	2.40E-04	2.15E-04
KEGG_PATHWAY	bta04612:Antigen processing and presentation	10	3.44828	4.69E-05	CD74, BOLA-NC1, TAP2, TAP1, BOLA-DQB, BOLA-DRA, LOC616942, BOLA, B2M, CTSB	189	84	9288	5.85034	0.01174	0.00295	0.00265
KEGG_PATHWAY	bta04925:Aldosterone synthesis and secretion	10	3.44828	1.34E-04	GNAQ, GNA11, ATP1A4, ATP2B4, ATP1A3, ATP2B3, ATP1A2, ATP1A1, ATP2B1, AGT	189	96	9288	5.11905	0.03321	0.00563	0.00505
KEGG_PATHWAY	bta04610:Complement and coagulation cascades	9	3.10345	5.69E-04	VTN, SERPIND1, F10, SERPINC1, C9, SERPING1, PLG, F2, CLU	189	93	9288	4.75576	0.13371	0.0205	0.01838
KEGG_PATHWAY	bta04022:cGMP-PKG signaling pathway	12	4.13793	6.90E-04	ROCK1, GNAQ, GNA11, ATP1A4, ATP2B4, ATP1A3, ATP2B3, ATP2A1, ATP1A2, ATP1A1, ATP2B1, GTF2I	189	171	9288	3.44862	0.15965	0.02173	0.01949
KEGG_PATHWAY	bta04972:Pancreatic secretion	9	3.10345	0.00105	GNAQ, ATP1A4, ATP2B4, ATP1A3, ATP2B3, ATP2A1, ATP1A2, ATP1A1, ATP2B1	189	102	9288	4.33613	0.23276	0.02648	0.02375
KEGG_PATHWAY	bta04261:Adrenergic signaling in cardiomyocytes	10	3.44828	0.00402	GNAQ, ATP1A4, ATP2B4, ATP1A3, ATP2B3, ATP2A1, ATP1A2, ATP1A1, ATP2B1, AGT	189	154	9288	3.19109	0.63722	0.06047	0.05423
KEGG_PATHWAY	bta04918:Thyroid hormone synthesis	7	2.41379	0.00402	TTR, GPX3, GNAQ, ATP1A4, ATP1A3, ATP1A2, ATP1A1	189	75	9288	4.58667	0.63767	0.06047	0.05423
KEGG_PATHWAY	bta04144:Endocytosis	13	4.48276	0.0041	USP8, BOLA-NC1, TFRC, AP2A2, CYTH3, CYTH2, PARD6B, PARD6A, LOC616942, BOLA, AP2M1, ARF5, CYTH1	189	245	9288	2.60758	0.64455	0.06047	0.05423
KEGG_PATHWAY	bta00983:Drug metabolism - other enzymes	7	2.41379	0.00429	RRM1, GSTA5, GSTA2, GSTA1, NME3, GUSB, XDH	189	76	9288	4.52632	0.66184	0.06047	0.05423
KEGG_PATHWAY	bta04970:Salivary secretion	8	2.75862	0.00432	GNAQ, ATP1A4, ATP2B4, ATP1A3, ATP2B3, ATP1A2, ATP1A1, ATP2B1	189	101	9288	3.8925	0.66404	0.06047	0.05423
KEGG_PATHWAY	bta03030:DNA replication	5	1.72414	0.00577	MCM7, MCM3, RPA1, MCM5, MCM2	189	36	9288	6.8254	0.76755	0.07657	0.06867
KEGG_PATHWAY	bta04512:ECM-receptor interaction	7	2.41379	0.0092	ITGB1, VTN, SDC4, DAG1, LAMC1, AGRN, THBS1	189	89	9288	3.86517	0.90271	0.11361	0.10189
KEGG_PATHWAY	bta04964:Proximal tubule bicarbonate reclamation	4	1.37931	0.00947	ATP1A4, ATP1A3, ATP1A2, ATP1A1	189	22	9288	8.93506	0.90903	0.11361	0.10189
KEGG_PATHWAY	bta04520:Adherens junction	7	2.41379	0.01075	TJP1, ROCK1, CDH1, PDCD10, CTNNA3, CTNNA2, BAIAP2	189	92	9288	3.73913	0.93435	0.12312	0.11042
KEGG_PATHWAY	bta04216:Ferroptosis	5	1.72414	0.01472	TF, TFRC, FTH1, ACSL4, CP	189	47	9288	5.22796	0.9762	0.16132	0.14468
KEGG_PATHWAY	bta00230:Purine metabolism	8	2.75862	0.0176	RRM1, GMPR2, GMPR, ENPP1, NME3, AK4, ENPP3, XDH	189	132	9288	2.97835	0.98861	0.18405	0.16506
KEGG_PATHWAY	bta04514:Cell adhesion molecules	9	3.10345	0.01826	ITGB1, BOLA-NC1, SDC4, CDH1, GLYCAM1, BOLA-DQB, BOLA-DRA, LOC616942, BOLA	189	164	9288	2.69686	0.99038	0.18405	0.16506
KEGG_PATHWAY	bta05330:Allograft rejection	5	1.72414	0.02068	BOLA-NC1, BOLA-DQB, BOLA-DRA, LOC616942, BOLA	189	52	9288	4.72527	0.99484	0.19279	0.1729
KEGG_PATHWAY	bta05416:Viral myocarditis	6	2.06897	0.02175	BOLA-NC1, DAG1, BOLA-DQB, BOLA-DRA, LOC616942, BOLA	189	79	9288	3.73237	0.99607	0.19279	0.1729
KEGG_PATHWAY	bta04142:Lysosome	8	2.75862	0.0219	NAGLU, AP3S2, GBA1, GUSB, CD68, LIPA, ATP6V0A1, CTSB	189	138	9288	2.84886	0.99622	0.19279	0.1729
KEGG_PATHWAY	bta00020:Citrate cycle (TCA cycle)	4	1.37931	0.02219	PDHA1, IDH2, SDHA, IDH3A	189	30	9288	6.55238	0.9965	0.19279	0.1729
KEGG_PATHWAY	bta04940:Type I diabetes mellitus	5	1.72414	0.02342	BOLA-NC1, BOLA-DQB, BOLA-DRA, LOC616942, BOLA	189	54	9288	4.55026	0.99745	0.19402	0.174
KEGG_PATHWAY	bta05165:Human papillomavirus infection	14	4.82759	0.02387	ITGB1, BOLA-NC1, MX2, MX1, ISG15, SCRIB, LAMC1, THBS1, VTN, PARD6B, PARD6A, LOC616942, BOLA, ATP6V0A1	189	347	9288	1.98271	0.99773	0.19402	0.174
KEGG_PATHWAY	bta05412:Arrhythmogenic right ventricular cardiomyopathy	6	2.06897	0.02628	ITGB1, JUP, DAG1, CTNNA3, ATP2A1, CTNNA2	189	83	9288	3.5525	0.99878	0.20479	0.18366
KEGG_PATHWAY	bta05016:Huntington disease	13	4.48276	0.02682	NDUFA8, GPX3, PIK3R4, TUBB4B, SDHA, AP2A2, TUBA4A, TUBA1C, TUBA3E, TUBB2B, TUBB2A, GNAQ, AP2M1	189	316	9288	2.0217	0.99894	0.20479	0.18366
KEGG_PATHWAY	bta04911:Insulin secretion	6	2.06897	0.02876	GNAQ, GNA11, ATP1A4, ATP1A3, ATP1A2, ATP1A1	189	85	9288	3.46891	0.99936	0.21313	0.19114
KEGG_PATHWAY	bta05332:Graft-versus-host disease	5	1.72414	0.0312	BOLA-NC1, BOLA-DQB, BOLA-DRA, LOC616942, BOLA	189	59	9288	4.16465	0.99966	0.22466	0.20148
KEGG_PATHWAY	bta05132:Salmonella infection	11	3.7931	0.03757	CYTH3, TUBA1C, CYTH2, TUBA3E, TUBB2B, TUBB2A, EXOC4, TUBB4B, TUBA4A, CYTH1, S100A10	189	259	9288	2.08715	0.99994	0.26107	0.23413
KEGG_PATHWAY	bta04960:Aldosterone-regulated sodium reabsorption	4	1.37931	0.03833	ATP1A4, ATP1A3, ATP1A2, ATP1A1	189	37	9288	5.31274	0.99995	0.26107	0.23413
KEGG_PATHWAY	bta04260:Cardiac muscle contraction	6	2.06897	0.04011	ASPH, ATP1A4, ATP1A3, ATP2A1, ATP1A2, ATP1A1	189	93	9288	3.17051	0.99997	0.26602	0.23857
KEGG_PATHWAY	bta05169:Epstein-Barr virus infection	10	3.44828	0.043	BOLA-NC1, USP7, TAP2, TAP1, BOLA-DQB, ISG15, BOLA-DRA, LOC616942, BOLA, B2M	189	229	9288	2.14598	0.99998	0.27787	0.2492
KEGG_PATHWAY	bta05320:Autoimmune thyroid disease	5	1.72414	0.04647	BOLA-NC1, BOLA-DQB, BOLA-DRA, LOC616942, BOLA	189	67	9288	3.66738	0.99999	0.29277	0.26257
KEGG_PATHWAY	bta05200:Pathways in cancer	18	6.2069	0.0477	ITGB1, JUP, ROCK1, DAPK1, TXNRD2, LAMC1, F2, AGT, LRP6, EML4, GSTA5, CDH1, GNAQ, GSTA2, GNA11, GSTA1, CTNNA3, CTNNA2	189	542	9288	1.63205	1	0.29318	0.26293
KEGG_PATHWAY	bta00860:Porphyrin metabolism	4	1.37931	0.04962	GUSB, CP, BLVRA, HMOX2	189	41	9288	4.79443	1	0.29773	0.26701
KEGG_PATHWAY	bta04814:Motor proteins	9	3.10345	0.05145	TUBA1C, TUBA3E, TUBB2B, TUBB2A, KIF4A, KIF20A, TUBB4B, MYH10, TUBA4A	189	201	9288	2.20043	1	0.30154	0.27043
KEGG_PATHWAY	bta05205:Proteoglycans in cancer	9	3.10345	0.05526	ITGB1, VTN, SDC4, ROCK1, GPC1, RDX, PDCD4, TIMP3, THBS1	189	204	9288	2.16807	1	0.31647	0.28382
KEGG_PATHWAY	bta04973:Carbohydrate digestion and absorption	4	1.37931	0.05903	ATP1A4, ATP1A3, ATP1A2, ATP1A1	189	44	9288	4.46753	1	0.33057	0.29647
KEGG_PATHWAY	bta00513:Various types of N-glycan biosynthesis	4	1.37931	0.06574	B4GALT1, MAN2A2, STT3B, B4GALNT3	189	46	9288	4.27329	1	0.34978	0.31369
KEGG_PATHWAY	bta05100:Bacterial invasion of epithelial cells	5	1.72414	0.06781	ITGB1, CDH1, SEPTIN2, CTNNA3, CTNNA2	189	76	9288	3.23308	1	0.34978	0.31369
KEGG_PATHWAY	bta04971:Gastric acid secretion	5	1.72414	0.06781	GNAQ, ATP1A4, ATP1A3, ATP1A2, ATP1A1	189	76	9288	3.23308	1	0.34978	0.31369
KEGG_PATHWAY	bta05022:Pathways of neurodegeneration - multiple diseases	16	5.51724	0.06877	APP, NDUFA8, TOMM40, GPX3, PIK3R4, ATP2A1, TUBB4B, SDHA, TUBA4A, LRP6, TUBA1C, TUBA3E, TUBB2B, TUBB2A, GNAQ, MAPT	189	487	9288	1.61455	1	0.34978	0.31369
KEGG_PATHWAY	bta04360:Axon guidance	8	2.75862	0.0694	ENAH, ITGB1, TRPC5, PARD6B, PARD6A, ROCK1, TRPC4, SEMA3G	189	178	9288	2.20867	1	0.34978	0.31369
KEGG_PATHWAY	bta04979:Cholesterol metabolism	4	1.37931	0.07277	APOA1, APOA4, APOE, LIPA	189	48	9288	4.09524	1	0.35959	0.32249
KEGG_PATHWAY	bta04810:Regulation of actin cytoskeleton	9	3.10345	0.08989	ENAH, ITGB1, SCIN, ROCK1, C9, RDX, F2, BAIAP2, MYH10	189	227	9288	1.9484	1	0.42915	0.38488
KEGG_PATHWAY	bta04977:Vitamin digestion and absorption	3	1.03448	0.09026	TCN2, APOA1, APOA4	189	25	9288	5.89714	1	0.42915	0.38488
KEGG_PATHWAY	bta04614:Renin-angiotensin system	3	1.03448	0.09652	MME, ATP6AP2, AGT	189	26	9288	5.67033	1	0.44346	0.3977
KEGG_PATHWAY	bta04390:Hippo signaling pathway	7	2.41379	0.09679	PARD6B, PARD6A, CDH1, CSNK1D, CTNNA3, SCRIB, CTNNA2	189	156	9288	2.20513	1	0.44346	0.3977

D1vsD15 Up-regulated

Category	Term	Count	%	PValue	Genes	List Total	Pop Hits	Pop Total	Fold Enric	Bonferron	Benjamini	FDR
GOTERM_BP_DIRECT	GO:0006002~fructose 6-phosphate metabolic process	5	1.65017	4.34E-06	PFKL, GFPT2, PFKM, FBP1, FBP2	273	8	16929	38.7569	0.00466	0.00467	0.00464
GOTERM_BP_DIRECT	GO:0010498~proteasomal protein catabolic process	7	2.31023	1.04E-05	PSMB6, PSMB7, PSMB4, PSMA4, PSMB5, PSMB3, PSMA2	273	32	16929	13.5649	0.01112	0.00559	0.00556
GOTERM_BP_DIRECT	GO:0016310~phosphorylation	11	3.63036	2.39E-05	PRPS1, PFKL, MVK, PANK3, GK, LOC538702, NME2, S100B, PFKM, MAPK13, GK2	273	120	16929	5.68434	0.02539	0.00857	0.00852
GOTERM_BP_DIRECT	GO:0030388~fructose 1,6-bisphosphate metabolic process	4	1.32013	3.21E-04	PFKL, PFKM, FBP1, FBP2	273	9	16929	27.5604	0.29214	0.08636	0.0858
GOTERM_BP_DIRECT	GO:0043161~proteasome-mediated ubiquitin-dependent protein catabolic process	10	3.30033	8.72E-04	ITCH, PSMB5, PSMC6, PSMD7, PSMC3, PSMD14, PSMB3, UBE2G1, RAD23B, SOD1	273	154	16929	4.02669	0.60918	0.18782	0.1866
GOTERM_BP_DIRECT	GO:0002181~cytoplasmic translation	7	2.31023	0.00149	SARS1, RPL31, TMA7, RPL26, RPL29, RPL10A, RPS21	273	77	16929	5.63736	0.79862	0.24884	0.24722
GOTERM_BP_DIRECT	GO:0006096~glycolytic process	5	1.65017	0.00164	PFKL, ENO1, ENO2, ENO3, GCK	273	32	16929	9.68922	0.83017	0.24884	0.24722
GOTERM_BP_DIRECT	GO:0016311~dephosphorylation	5	1.65017	0.00185	PPP3CA, ALPL, HDHD2, FBP1, PSPH	273	33	16929	9.3956	0.86365	0.24884	0.24722
GOTERM_BP_DIRECT	GO:0046167~glycerol-3-phosphate biosynthetic process	3	0.9901	0.00249	GK, LOC538702, GK2	273	5	16929	37.2066	0.93186	0.29561	0.29369
GOTERM_BP_DIRECT	GO:0006511~ubiquitin-dependent protein catabolic process	11	3.63036	0.00274	USP24, ITCH, NPLOC4, PSMA4, CUL5, PSMA2, ADRM1, PSMD3, UBE2G1, GET4, RBX1	273	217	16929	3.14341	0.94819	0.29561	0.29369
GOTERM_BP_DIRECT	GO:0005975~carbohydrate metabolic process	8	2.64026	0.00341	LDHB, MDH1, HEXB, GPD1, PGM3, FBP1, FBP2, PGM1	273	121	16929	4.0999	0.97479	0.30629	0.3043
GOTERM_BP_DIRECT	GO:1990592~protein K69-linked ufmylation	3	0.9901	0.0037	UFM1, UBA5, UFC1	273	6	16929	31.0055	0.98148	0.30629	0.3043
GOTERM_BP_DIRECT	GO:0071569~protein ufmylation	3	0.9901	0.0037	UFM1, UBA5, UFC1	273	6	16929	31.0055	0.98148	0.30629	0.3043
GOTERM_BP_DIRECT	GO:0061621~canonical glycolysis	3	0.9901	0.00512	PFKL, ENO1, PFKM	273	7	16929	26.5761	0.99603	0.32444	0.32233
GOTERM_BP_DIRECT	GO:0006739~NADP metabolic process	3	0.9901	0.00512	MDH1, IDH1, GCK	273	7	16929	26.5761	0.99603	0.32444	0.32233
GOTERM_BP_DIRECT	GO:0006072~glycerol-3-phosphate metabolic process	3	0.9901	0.00512	GK, LOC538702, GPD1	273	7	16929	26.5761	0.99603	0.32444	0.32233
GOTERM_BP_DIRECT	GO:0019563~glycerol catabolic process	3	0.9901	0.00512	GK, LOC538702, GK2	273	7	16929	26.5761	0.99603	0.32444	0.32233
GOTERM_BP_DIRECT	GO:0006750~glutathione biosynthetic process	3	0.9901	0.00676	GSS, MGST2, GCLM	273	8	16929	23.2541	0.99933	0.40424	0.40161
GOTERM_BP_DIRECT	GO:0006695~cholesterol biosynthetic process	4	1.32013	0.00726	FDPS, IDI1, MVK, DHCR7	273	25	16929	9.92176	0.99961	0.41176	0.40908
GOTERM_BP_DIRECT	GO:0034976~response to endoplasmic reticulum stress	5	1.65017	0.01245	UFM1, UBA5, ALOX15, UFC1, CERT1	273	56	16929	5.5367	1	0.65932	0.65503
GOTERM_BP_DIRECT	GO:0006071~glycerol metabolic process	3	0.9901	0.01286	GK, LOC538702, GK2	273	11	16929	16.9121	1	0.65932	0.65503
GOTERM_BP_DIRECT	GO:0010033~response to organic substance	3	0.9901	0.01526	FKBP1A, ANXA5, SOD1	273	12	16929	15.5027	1	0.74728	0.74242
GOTERM_BP_DIRECT	GO:0061077~chaperone-mediated protein folding	4	1.32013	0.017	FKBP1A, FKBP11, FKBP2, LOC526524	273	34	16929	7.29541	1	0.76902	0.76402
GOTERM_BP_DIRECT	GO:0050872~white fat cell differentiation	3	0.9901	0.01785	FABP4, CTBP2, CTBP1	273	13	16929	14.3102	1	0.76902	0.76402
GOTERM_BP_DIRECT	GO:0061709~reticulophagy	3	0.9901	0.01785	UFM1, UBA5, UFC1	273	13	16929	14.3102	1	0.76902	0.76402
GOTERM_BP_DIRECT	GO:0009116~nucleoside metabolic process	3	0.9901	0.02061	PRPS2, PRPS1, PNP	273	14	16929	13.2881	1	0.85034	0.84482
GOTERM_BP_DIRECT	GO:0007052~mitotic spindle organization	4	1.32013	0.02132	DCTN6, PTPA, NUDC, DCTN2	273	37	16929	6.70389	1	0.85034	0.84482
GOTERM_BP_DIRECT	GO:0006633~fatty acid biosynthetic process	4	1.32013	0.02288	SCD, FASN, ACACB, ACACA	273	38	16929	6.52747	1	0.85447	0.84891
GOTERM_BP_DIRECT	GO:0042953~lipoprotein transport	3	0.9901	0.02353	LOC518526, MSR1, CD36	273	15	16929	12.4022	1	0.85447	0.84891
GOTERM_BP_DIRECT	GO:0006979~response to oxidative stress	5	1.65017	0.0238	PSMB5, NAPRT, IDH1, LPO, GCLM	273	68	16929	4.55963	1	0.85447	0.84891
GOTERM_BP_DIRECT	GO:0006541~glutamine metabolic process	3	0.9901	0.02984	GFPT2, CAD, CTPS1	273	17	16929	10.9431	1	1	0.99442
GOTERM_BP_DIRECT	GO:0038127~ERBB signaling pathway	2	0.66007	0.03188	MVP, MAPK1	273	2	16929	62.011	1	1	0.99442
GOTERM_BP_DIRECT	GO:0005986~sucrose biosynthetic process	2	0.66007	0.03188	FBP1, FBP2	273	2	16929	62.011	1	1	0.99442
GOTERM_BP_DIRECT	GO:1990830~cellular response to leukemia inhibitory factor	5	1.65017	0.0399	CTBP2, GFPT2, CACYBP, GCLM, SLC25A5	273	80	16929	3.87569	1	1	0.99442
GOTERM_BP_DIRECT	GO:0006564~L-serine biosynthetic process	2	0.66007	0.04743	PSAT1, PSPH	273	3	16929	41.3407	1	1	0.99442
GOTERM_BP_DIRECT	GO:0097720~calcineurin-mediated signaling	2	0.66007	0.04743	PPP3CA, PPP3CC	273	3	16929	41.3407	1	1	0.99442
GOTERM_BP_DIRECT	GO:2001295~malonyl-CoA biosynthetic process	2	0.66007	0.04743	ACACB, ACACA	273	3	16929	41.3407	1	1	0.99442
GOTERM_BP_DIRECT	GO:1901223~negative regulation of NIK/NF-kappaB signaling	3	0.9901	0.05214	PPM1A, DDX3X, UACA	273	23	16929	8.08839	1	1	0.99442
GOTERM_BP_DIRECT	GO:0006641~triglyceride metabolic process	3	0.9901	0.0563	GK, LOC538702, GK2	273	24	16929	7.75137	1	1	0.99442
GOTERM_BP_DIRECT	GO:0030307~positive regulation of cell growth	4	1.32013	0.05844	PSMD10, DDX3X, KRT17, SFN	273	55	16929	4.50989	1	1	0.99442
GOTERM_BP_DIRECT	GO:0016192~vesicle-mediated transport	7	2.31023	0.06087	AP3M1, GDI1, CLTB, CCZ1, TRAPPC11, VPS45, AP3S1	273	173	16929	2.50912	1	1	0.99442
GOTERM_BP_DIRECT	GO:0009156~ribonucleoside monophosphate biosynthetic process	2	0.66007	0.06274	PRPS2, PRPS1	273	4	16929	31.0055	1	1	0.99442
GOTERM_BP_DIRECT	GO:0006047~UDP-N-acetylglucosamine metabolic process	2	0.66007	0.06274	GFPT2, GNE	273	4	16929	31.0055	1	1	0.99442
GOTERM_BP_DIRECT	GO:0046686~response to cadmium ion	2	0.66007	0.06274	SLC34A1, GSS	273	4	16929	31.0055	1	1	0.99442
GOTERM_BP_DIRECT	GO:0033499~galactose catabolic process via UDP-galactose	2	0.66007	0.06274	GALE, GALM	273	4	16929	31.0055	1	1	0.99442
GOTERM_BP_DIRECT	GO:0032024~positive regulation of insulin secretion	3	0.9901	0.0694	PFKM, AACS, GCK	273	27	16929	6.89011	1	1	0.99442
GOTERM_BP_DIRECT	GO:0019915~lipid storage	3	0.9901	0.07397	LOC518526, PLIN3, CD36	273	28	16929	6.64403	1	1	0.99442
GOTERM_BP_DIRECT	GO:0044341~sodium-dependent phosphate transport	2	0.66007	0.0778	SLC34A2, SLC34A1	273	5	16929	24.8044	1	1	0.99442
GOTERM_BP_DIRECT	GO:0009298~GDP-mannose biosynthetic process	2	0.66007	0.0778	GMPPB, PMM2	273	5	16929	24.8044	1	1	0.99442
GOTERM_BP_DIRECT	GO:0009165~nucleotide biosynthetic process	2	0.66007	0.0778	PRPS2, PRPS1	273	5	16929	24.8044	1	1	0.99442
GOTERM_BP_DIRECT	GO:0007017~microtubule-based process	3	0.9901	0.07864	DCTN2, DCTN3, MAP3K11	273	29	16929	6.41493	1	1	0.99442
GOTERM_BP_DIRECT	GO:0006198~cAMP catabolic process	2	0.66007	0.09263	PDE4B, PDE4A	273	6	16929	20.6703	1	1	0.99442
GOTERM_BP_DIRECT	GO:1902523~positive regulation of protein K63-linked ubiquitination	2	0.66007	0.09263	DDX3X, UBE2V1	273	6	16929	20.6703	1	1	0.99442
GOTERM_BP_DIRECT	GO:0006084~acetyl-CoA metabolic process	2	0.66007	0.09263	ACACB, ACACA	273	6	16929	20.6703	1	1	0.99442
GOTERM_BP_DIRECT	GO:0006015~5-phosphoribose 1-diphosphate biosynthetic process	2	0.66007	0.09263	PRPS2, PRPS1	273	6	16929	20.6703	1	1	0.99442
GOTERM_BP_DIRECT	GO:1990000~amyloid fibril formation	2	0.66007	0.09263	FKBP1A, CD36	273	6	16929	20.6703	1	1	0.99442
GOTERM_BP_DIRECT	GO:0006099~tricarboxylic acid cycle	3	0.9901	0.09315	MDH1, IDH1, ACO1	273	32	16929	5.81353	1	1	0.99442
GOTERM_BP_DIRECT	GO:0043280~positive regulation of cysteine-type endopeptidase activity involved in apoptotic process	3	0.9901	0.09315	DDX3X, PDCD5, UACA	273	32	16929	5.81353	1	1	0.99442

Category	Term	Count	%	PValue	Genes	List Total	Pop Hits	Pop Total	Fold Enric	Bonferron	Benjamini	FDR
GOTERM_CC_DIRECT	GO:0005829~cytosol	106	34.9835	1.89E-22	AHCYL2, PANK3, PANK1, CTNND1, PDCD5, YARS1, DPYSL2, PSPH, KPNA1, CAST, SH3GLB2, SLC34A1, TOMM34, RIMOC1, GPD1, UBE2V1, LAP3, STAMBP, PSMD10, FAM114A1, UBA5, ALOX15, GIGYF2, ACACA, DPP3, SFN, CERT1, FDPS, IDH1, PMM2, STARD10, DENND4C, ASPSCR1, MTHFR, SNF8, HARS2, NFKB2, EIF5, CAP	281	2967	20801	2.64464	5.52E-20	5.52E-20	5.33E-20
GOTERM_CC_DIRECT	GO:0005737~cytoplasm	115	37.9538	1.33E-17	TPMT, CTNND1, SDR9C7, ENO1, YARS1, LGALS3, PNP, FNTA, DPYSL2, CHP1, EIF2D, PSPH, KPNA1, CAST, SH3GLB2, LOC526524, NCCRP1, LAP3, TARS1, IDI1, AARS1, UBA5, LPO, STK3, DPP3, RIC8A, LDHB, PRRC1, SFN, EIF2B3, IDH1, PMM2, DENND4C, DNAJC12, QDPR, CAPRIN1, FASN, BLMH, ACO1, GDI1, GPHN, GMPPB, I	281	3943	20801	2.15898	3.89E-15	1.94E-15	1.88E-15
GOTERM_CC_DIRECT	GO:0005839~proteasome core complex	7	2.31023	2.58E-07	PSMB6, PSMB7, PSMB4, PSMA4, PSMB5, PSMB3, PSMA2	281	21	20801	24.675	7.54E-05	2.51E-05	2.43E-05
GOTERM_CC_DIRECT	GO:0019774~proteasome core complex, beta-subunit complex	5	1.65017	9.84E-06	PSMB6, PSMB7, PSMB4, PSMB5, PSMB3	281	11	20801	33.6477	0.00287	7.19E-04	6.94E-04
GOTERM_CC_DIRECT	GO:0005741~mitochondrial outer membrane	10	3.30033	5.32E-05	HADHB, GK, MSTO1, OPA1, MGST3, LOC538702, TOMM34, KMO, GCK, GK2	281	126	20801	5.87499	0.01542	0.00311	0.003
GOTERM_CC_DIRECT	GO:0005739~mitochondrion	29	9.57096	1.44E-04	AARS1, ACACB, ACAT2, GK2, PSMB6, PPP3CA, PSMB4, TMEM102, LOC538702, DBT, MAPK1, CRYM, CERT1, ACAD10, RANBP2, GK, IDH1, TOMM34, HARS2, GCK, ASS1, SOD1, HADHB, CYP27A1, GARS1, SFXN1, ACO1, LAP3, HAGH	281	977	20801	2.19726	0.04117	0.00701	0.00677
GOTERM_CC_DIRECT	GO:0000502~proteasome complex	5	1.65017	2.76E-04	PSMD10, PSMD7, PSMC3, ADRM1, RAD23B	281	24	20801	15.4219	0.07747	0.01152	0.01112
GOTERM_CC_DIRECT	GO:0016020~membrane	29	9.57096	8.62E-04	FKBP11, DCTN2, HEXB, ALOX15, LAMC1, JAKMIP1, GIGYF2, ABR, NT5E, OPA1, DPYSL2, CD36, SEC61B, CAST, SH3GLB2, SLC34A2, TMEM120B, LAMB2, STARD10, ANXA5, FKBP1A, CYP27A1, PPM1A, PFKL, SCD, PIK3C3, STAMBP, SLC25A5, PFKM	281	1093	20801	1.96406	0.2226	0.03146	0.03038
GOTERM_CC_DIRECT	GO:0000015~phosphopyruvate hydratase complex	3	0.9901	0.00106	ENO1, ENO2, ENO3	281	4	20801	55.5187	0.26721	0.03453	0.03334
GOTERM_CC_DIRECT	GO:0005777~peroxisome	6	1.9802	0.00342	IDI1, MVK, EHHADH, IDH1, PIK3C3, SOD1	281	75	20801	5.92199	0.63188	0.09976	0.09635
GOTERM_CC_DIRECT	GO:0008541~proteasome regulatory particle, lid subcomplex	3	0.9901	0.00479	PSMD14, ADRM1, PSMD3	281	8	20801	27.7593	0.75405	0.1272	0.12285
GOTERM_CC_DIRECT	GO:0022625~cytosolic large ribosomal subunit	7	2.31023	0.00574	RPL30, RPL31, LOC101906221, RPL23A, RPL26, RPL29, RPL10A	281	120	20801	4.31812	0.81355	0.13957	0.13479
GOTERM_CC_DIRECT	GO:0005794~Golgi apparatus	20	6.60066	0.00683	GDI1, FAM114A1, UBA5, DENND4C, DBI, FNDC3A, GIGYF2, AP3M1, LOC518526, PRRC1, FASN, TRAPPC11, MAPK1, ACO1, AP3S1, SCLY, CD36, CERT1, ITM2B, RAB3GAP1	281	754	20801	1.96353	0.86483	0.15342	0.14816
GOTERM_CC_DIRECT	GO:0022627~cytosolic small ribosomal subunit	6	1.9802	0.00774	DDX3X, RPS19, EIF2D, FAU, RPS21, RPS10	281	91	20801	4.88076	0.89667	0.1615	0.15597
GOTERM_CC_DIRECT	GO:0008540~proteasome regulatory particle, base subcomplex	3	0.9901	0.01277	PSMD10, PSMC6, PSMC3	281	13	20801	17.0827	0.97655	0.24352	0.23518
GOTERM_CC_DIRECT	GO:0031982~vesicle	5	1.65017	0.01334	SLC34A2, DYNC1I2, DCTN2, SLC34A1, GIGYF2	281	68	20801	5.44301	0.98021	0.24352	0.23518
GOTERM_CC_DIRECT	GO:0030496~midbody	7	2.31023	0.01519	CENPF, NUDC, GDI1, DCTN3, CTNND1, PIK3C3, LAP3	281	148	20801	3.50118	0.98856	0.25079	0.2422
GOTERM_CC_DIRECT	GO:0031090~organelle membrane	6	1.9802	0.01546	PSMC6, YBX2, RPL26, YBX1, YARS1, ITM2B	281	108	20801	4.1125	0.98943	0.25079	0.2422
GOTERM_CC_DIRECT	GO:0005654~nucleoplasm	42	13.8614	0.01839	WASHC4, DCTN5, DDX3X, ACSS2, FAM114A1, CTBP1, WIPF2, UACA, LIMD2, PTPA, PSMB6, PPP3CA, NT5E, PSMB4, PSMD7, PSMB5, OPA1, PDE4A, CERT1, GET4, KPNA1, RANBP2, FDPS, SH3GLB2, CAD, ADRM1, ASPSCR1, CACYBP, SNF8, S100B, RAD23B, RABGGTA, RIMOC1, PM20D2, ITCH, CENPF, PSMA4, HDGF, STAMB	281	2188	20801	1.42095	0.99558	0.28266	0.27298
GOTERM_CC_DIRECT	GO:0042645~mitochondrial nucleoid	4	1.32013	0.02129	HADHB, LRRC59, DBT, SLC25A5	281	44	20801	6.72954	0.99814	0.29885	0.28862
GOTERM_CC_DIRECT	GO:0005869~dynactin complex	3	0.9901	0.02149	DCTN6, DCTN2, DCTN3	281	17	20801	13.0632	0.99824	0.29885	0.28862
GOTERM_CC_DIRECT	GO:0030018~Z disc	5	1.65017	0.03455	FKBP1A, PPP3CA, GLRX3, PDE4B, FBP2	281	91	20801	4.0673	0.99997	0.45852	0.44281
GOTERM_CC_DIRECT	GO:0005635~nuclear envelope	5	1.65017	0.03822	RANBP2, LRRC59, CENPF, MGST3, MGST2	281	94	20801	3.9375	0.99999	0.47224	0.45607
GOTERM_CC_DIRECT	GO:0005945~6-phosphofructokinase complex	2	0.66007	0.03984	PFKL, PFKM	281	3	20801	49.3499	0.99999	0.47224	0.45607
GOTERM_CC_DIRECT	GO:0005813~centrosome	12	3.9604	0.04043	DCTN6, DYNC1I2, DCTN5, ACTR1A, PSMB5, DDX3X, MDH1, DCTN2, DCTN3, PDE4B, SORBS1, MAP3K11	281	446	20801	1.9917	0.99999	0.47224	0.45607
GOTERM_CC_DIRECT	GO:0005783~endoplasmic reticulum	16	5.28053	0.04603	CAST, AHCYL2, MGST3, MGST2, DBI, CNPY3, YBX1, GIGYF2, HADHB, UFM1, TBL2, AGR2, YIPF6, CHP1, ACO1, CERT1	281	687	20801	1.72402	1	0.5169	0.49919
GOTERM_CC_DIRECT	GO:0070062~extracellular exosome	4	1.32013	0.05945	FKBP1A, GARS1, FN1, YBX1	281	66	20801	4.48636	1	0.64292	0.6209
GOTERM_CC_DIRECT	GO:0005955~calcineurin complex	2	0.66007	0.06552	PPP3CA, PPP3CC	281	5	20801	29.61	1	0.64371	0.62167
GOTERM_CC_DIRECT	GO:0098791~Golgi subcompartment	2	0.66007	0.06552	ARFGEF2, GBF1	281	5	20801	29.61	1	0.64371	0.62167
GOTERM_CC_DIRECT	GO:0010494~cytoplasmic stress granule	4	1.32013	0.06613	DDX3X, CAPRIN1, YBX1, GIGYF2	281	69	20801	4.2913	1	0.64371	0.62167
GOTERM_CC_DIRECT	GO:0005634~nucleus	67	22.1122	0.06999	DDX3X, PANK3, LOC107133263, PANK1, PDCD5, CTNND1, YBX2, YBX1, YARS1, LGALS3, PDE4B, CHP1, PDE4A, KPNA1, NUDC, GLRX3, NME2, CACYBP, RBX1, PPM1A, SARS1, SFPQ, PSMA4, PSMA2, PREB, UBE2V1, FBP1, FBP2, PSMD10, CTBP2, WBP2, CTBP1, UBA5, MVP, ACACB, UACA, STK3, PTPA, PSMB7, PSMB4, PSMB	281	4131	20801	1.2006	1	0.6593	0.63673
GOTERM_CC_DIRECT	GO:0032991~macromolecular complex	9	2.9703	0.07504	ITCH, TMEM102, SUGT1, GIGYF2, RAB3GAP1, WARS1, SOD1, HSPD1, STK3	281	326	20801	2.04363	1	0.67267	0.64964
GOTERM_CC_DIRECT	GO:0002189~ribose phosphate diphosphokinase complex	2	0.66007	0.0781	PRPS2, PRPS1	281	6	20801	24.675	1	0.67267	0.64964
GOTERM_CC_DIRECT	GO:0005874~microtubule	7	2.31023	0.07832	DYNC1I2, NUDC, OPA1, DCTN2, DPYSL2, JAKMIP1, MAP3K11	281	221	20801	2.34468	1	0.67267	0.64964
GOTERM_CC_DIRECT	GO:0098831~presynaptic active zone cytoplasmic component	2	0.66007	0.09052	CTBP2, CTBP1	281	7	20801	21.15	1	0.74266	0.71723
GOTERM_CC_DIRECT	GO:0043231~intracellular membrane-bounded organelle	8	2.64026	0.09156	FKBP1A, RPIA, LRG1, MGST3, CCZ1, LRRC40, MGST2, AP3S1	281	285	20801	2.07789	1	0.74266	0.71723

Category	Term	Count	%	PValue	Genes	List Total	Pop Hits	Pop Total	Fold Enric	Bonferron	Benjamini	FDR
GOTERM_MF_DIRECT	GO:0042802~identical protein binding	40	13.2013	1.12E-08	PRPS2, PRPS1, MVK, DCTN2, CTBP1, MGST3, MVP, MGST2, DBI, ACACB, GPHN, ACACA, STK3, NT5E, UGP2, AGR2, MAPK1, SFN, MSR1, SH3GLB2, GLRX3, CAD, FN1, CTPS1, HARS2, ASS1, RPIA, GARS1, PM20D2, PFKL, PSMC6, PSMC3, PSAT1, EIF3J, YIPF6, ANGPTL4, FBP1, PFKM, TARS1, FBP2	253	1047	18413	2.78046	5.26E-06	5.26E-06	5.17E-06
GOTERM_MF_DIRECT	GO:0005524~ATP binding	45	14.8515	2.44E-07	PRPS2, DDX3Y, PRPS1, AARS1, MVK, DDX3X, PANK3, PANK1, UBA5, YARS1, ACACB, GPHN, ACACA, AACS, CAMKK2, GK2, HSPD1, STK3, PTPA, LOC538702, MAPK1, MAP2K4, MAP2K2, GK, GSS, CAD, NME2, CTPS1, UBE2G1, HARS2, GCK, ASS1, WARS1, MAPK13, GARS1, SARS1, PFKL, PSMC6, PSMC3, CAPRIN1, PIK3C3, PFK	253	1419	18413	2.30799	1.14E-04	5.72E-05	5.62E-05
GOTERM_MF_DIRECT	GO:0004298~threonine-type endopeptidase activity	6	1.9802	8.35E-07	PSMB6, PSMB7, PSMB4, PSMA4, PSMB5, PSMB3	253	14	18413	31.1909	3.91E-04	1.30E-04	1.28E-04
GOTERM_MF_DIRECT	GO:0042803~protein homodimerization activity	24	7.92079	6.60E-06	PRPS1, PANK3, PANK1, GSS, UBA5, IDH1, CACYBP, ENO1, SNF8, S100B, WARS1, SOD1, FKBP1A, PTPA, SARS1, CENPF, GALE, SFPQ, PSMD7, GPD1, AOX1, CRYM, MAP3K11, PSPH	253	588	18413	2.97056	0.00308	7.72E-04	7.59E-04
GOTERM_MF_DIRECT	GO:0000287~magnesium ion binding	13	4.29043	2.67E-05	PRPS2, PRPS1, MVK, GSS, IDH1, ENO1, ENO2, ENO3, STK3, PPM1A, PGM3, PGM1, PSPH	253	205	18413	4.61523	0.01244	0.0025	0.00246
GOTERM_MF_DIRECT	GO:0016740~transferase activity	7	2.31023	2.00E-04	RANBP2, FDPS, GARS1, FNTA, NAPRT, UBE2G1, SCLY	253	62	18413	8.21695	0.08956	0.01564	0.01537
GOTERM_MF_DIRECT	GO:0003755~peptidyl-prolyl cis-trans isomerase activity	6	1.9802	4.43E-04	FKBP1A, RANBP2, PTPA, FKBP11, FKBP2, LOC526524	253	47	18413	9.29089	0.1872	0.0296	0.0291
GOTERM_MF_DIRECT	GO:0004634~phosphopyruvate hydratase activity	3	0.9901	0.0011	ENO1, ENO2, ENO3	253	4	18413	54.584	0.40235	0.06431	0.06321
GOTERM_MF_DIRECT	GO:0051287~NAD binding	5	1.65017	0.00159	CTBP2, CTBP1, IDH1, GPD1, AOX1	253	37	18413	9.83495	0.52436	0.0825	0.08109
GOTERM_MF_DIRECT	GO:0004370~glycerol kinase activity	3	0.9901	0.00182	GK, LOC538702, GK2	253	5	18413	43.6672	0.57278	0.08497	0.08352
GOTERM_MF_DIRECT	GO:0005085~guanyl-nucleotide exchange factor activity	10	3.30033	0.00219	RIC8A, ABR, ARFGEF2, EIF2B3, DENND4C, RAB3IP, GBF1, CCZ1, PREB, RAB3GAP1	253	206	18413	3.53294	0.64196	0.09327	0.09168
GOTERM_MF_DIRECT	GO:0048029~monosaccharide binding	3	0.9901	0.00495	PFKL, PFKM, FBP1	253	8	18413	27.292	0.90184	0.19295	0.18965
GOTERM_MF_DIRECT	GO:0004708~MAP kinase kinase activity	3	0.9901	0.0063	MAP2K4, MAP2K2, MAPK1	253	9	18413	24.2596	0.94815	0.22693	0.22306
GOTERM_MF_DIRECT	GO:0016597~amino acid binding	3	0.9901	0.00781	AARS1, CAD, ASS1	253	10	18413	21.8336	0.9745	0.26104	0.25658
GOTERM_MF_DIRECT	GO:0016773~phosphotransferase activity, alcohol group as acceptor	3	0.9901	0.01125	GK, LOC538702, GK2	253	12	18413	18.1947	0.99498	0.30967	0.30437
GOTERM_MF_DIRECT	GO:0016208~AMP binding	3	0.9901	0.01125	PFKL, PFKM, FBP1	253	12	18413	18.1947	0.99498	0.30967	0.30437
GOTERM_MF_DIRECT	GO:0070122~isopeptidase activity	3	0.9901	0.01125	PSMD7, PSMD14, STAMBP	253	12	18413	18.1947	0.99498	0.30967	0.30437
GOTERM_MF_DIRECT	GO:0003735~structural constituent of ribosome	11	3.63036	0.01306	RPL30, RPS19, RPL31, LOC101906221, RPL23A, RPL26, FAU, RPL29, RPL10A, RPS21, RPS10	253	320	18413	2.50177	0.99788	0.33968	0.33387
GOTERM_MF_DIRECT	GO:0001222~transcription corepressor binding	4	1.32013	0.01724	CTBP2, CTBP1, HDGF, ENO1	253	40	18413	7.27787	0.99971	0.4247	0.41744

GOTERM_MF_DIRECT	GO:0070628~proteasome binding	3	0.9901	0.01973	PSMD14, ADRM1, RAD23B	253	16	18413	13.646	0.99991	0.4617	0.4538
GOTERM_MF_DIRECT	GO:0051087~chaperone binding	5	1.65017	0.02411	FN1, SUGT1, SOD1, GET4, HSPD1	253	80	18413	4.54867	0.99999	0.53732	0.52813
GOTERM_MF_DIRECT	GO:0042132~fructose 1,6-bisphosphate 1-phosphatase activity	2	0.66007	0.02719	FBP1, FBP2	253	2	18413	72.7787	1	0.57831	0.56842
GOTERM_MF_DIRECT	GO:0005515~protein binding	8	2.64026	0.0295	PPP3CA, AHCYL2, DPYSL2, CLTB, NME2, FN1, S100B, SOD1	253	216	18413	2.69551	1	0.58779	0.57774
GOTERM_MF_DIRECT	GO:0030169~low-density lipoprotein particle binding	3	0.9901	0.03014	LOC518526, MSR1, CD36	253	20	18413	10.9168	1	0.58779	0.57774
GOTERM_MF_DIRECT	GO:0046872~metal ion binding	32	10.5611	0.03831	IDI1, ACACB, GPHN, ACACA, LIMD2, DPP3, STK3, PPP3CA, NT5E, UGP2, PPP3CC, NAPRT, PDE4B, PDE4A, DTD1, RANBP2, FDPS, ARMT1, GLRX3, PMM2, NME2, HDHD2, ATOX1, SOD1, PFKL, NPLOC4, SCD, ACO1, HAGH, FBP1, PFKM, FBP2	253	1630	18413	1.42878	1	0.6313	0.62051
GOTERM_MF_DIRECT	GO:1905502~acetyl-CoA binding	2	0.66007	0.0405	PANK3, PANK1	253	3	18413	48.5191	1	0.6313	0.62051
GOTERM_MF_DIRECT	GO:0003989~acetyl-CoA carboxylase activity	2	0.66007	0.0405	ACACB, ACACA	253	3	18413	48.5191	1	0.6313	0.62051
GOTERM_MF_DIRECT	GO:0004614~phosphoglucomutase activity	2	0.66007	0.0405	PGM3, PGM1	253	3	18413	48.5191	1	0.6313	0.62051
GOTERM_MF_DIRECT	GO:0070095~fructose-6-phosphate binding	2	0.66007	0.0405	PFKL, PFKM	253	3	18413	48.5191	1	0.6313	0.62051
GOTERM_MF_DIRECT	GO:0003872~6-phosphofructokinase activity	2	0.66007	0.0405	PFKL, PFKM	253	3	18413	48.5191	1	0.6313	0.62051
GOTERM_MF_DIRECT	GO:0016779~nucleotidyltransferase activity	3	0.9901	0.04226	GMPPB, EIF2B3, GMPPA	253	24	18413	9.09733	1	0.6313	0.62051
GOTERM_MF_DIRECT	GO:0003743~translation initiation factor activity	4	1.32013	0.04317	EIF2B3, EIF5, EIF3J, EIF2D	253	57	18413	5.10727	1	0.6313	0.62051
GOTERM_MF_DIRECT	GO:0019900~kinase binding	4	1.32013	0.04703	PFKL, CHP1, JAKMIP1, PFKM	253	59	18413	4.93415	1	0.64362	0.63261
GOTERM_MF_DIRECT	GO:0051020~GTPase binding	3	0.9901	0.05233	PREB, ENO1, YBX1	253	27	18413	8.08652	1	0.64362	0.63261
GOTERM_MF_DIRECT	GO:0050660~flavin adenine dinucleotide binding	4	1.32013	0.05313	AOX1, MTHFR, KMO, ACAD10	253	62	18413	4.6954	1	0.64362	0.63261
GOTERM_MF_DIRECT	GO:0004594~pantothenate kinase activity	2	0.66007	0.05363	PANK3, PANK1	253	4	18413	36.3893	1	0.64362	0.63261
GOTERM_MF_DIRECT	GO:0004464~leukotriene-C4 synthase activity	2	0.66007	0.05363	MGST3, MGST2	253	4	18413	36.3893	1	0.64362	0.63261
GOTERM_MF_DIRECT	GO:0033192~calmodulin-dependent protein phosphatase activity	2	0.66007	0.05363	PPP3CA, PPP3CC	253	4	18413	36.3893	1	0.64362	0.63261
GOTERM_MF_DIRECT	GO:0004663~Rab geranylgeranyltransferase activity	2	0.66007	0.05363	FNTA, RABGGTA	253	4	18413	36.3893	1	0.64362	0.63261
GOTERM_MF_DIRECT	GO:0016791~phosphatase activity	3	0.9901	0.06316	ARMT1, HDHD2, FBP1	253	30	18413	7.27787	1	0.73896	0.72633
GOTERM_MF_DIRECT	GO:0019210~kinase inhibitor activity	2	0.66007	0.06659	S100B, WARS1	253	5	18413	29.1115	1	0.74571	0.73297
GOTERM_MF_DIRECT	GO:0016616~oxidoreductase activity, acting on the CH-OH group of donors, NAD or NADP as acceptor	3	0.9901	0.06692	QDPR, CTBP2, CTBP1	253	31	18413	7.0431	1	0.74571	0.73297
GOTERM_MF_DIRECT	GO:0000049~tRNA binding	4	1.32013	0.06874	SARS1, AARS1, YARS1, DTD1	253	69	18413	4.21905	1	0.74813	0.73534
GOTERM_MF_DIRECT	GO:0043546~molybdopterin cofactor binding	2	0.66007	0.07937	AOX1, GPHN	253	6	18413	24.2596	1	0.81212	0.79824
GOTERM_MF_DIRECT	GO:0004749~ribose phosphate diphosphokinase activity	2	0.66007	0.07937	PRPS2, PRPS1	253	6	18413	24.2596	1	0.81212	0.79824
GOTERM_MF_DIRECT	GO:0019899~enzyme binding	6	1.9802	0.08137	MSR1, SARS1, ARMT1, EHHADH, CAD, HDHD2	253	168	18413	2.59924	1	0.81212	0.79824
GOTERM_MF_DIRECT	GO:0008237~metallopeptidase activity	3	0.9901	0.09094	PSMD7, PSMD14, STAMBP	253	37	18413	5.90097	1	0.81212	0.79824
GOTERM_MF_DIRECT	GO:0036402~proteasome-activating ATPase activity	2	0.66007	0.09197	PSMC6, PSMC3	253	7	18413	20.7939	1	0.81212	0.79824
GOTERM_MF_DIRECT	GO:0005436~sodium:phosphate symporter activity	2	0.66007	0.09197	SLC34A2, SLC34A1	253	7	18413	20.7939	1	0.81212	0.79824
GOTERM_MF_DIRECT	GO:0016627~oxidoreductase activity, acting on the CH-CH group of donors	2	0.66007	0.09197	DHCR7, ACAD10	253	7	18413	20.7939	1	0.81212	0.79824
GOTERM_MF_DIRECT	GO:0036041~long-chain fatty acid binding	2	0.66007	0.09197	FABP3, FABP4	253	7	18413	20.7939	1	0.81212	0.79824
GOTERM_MF_DIRECT	GO:0070061~fructose binding	2	0.66007	0.09197	PFKL, PFKM	253	7	18413	20.7939	1	0.81212	0.79824
GOTERM_MF_DIRECT	GO:0043295~glutathione binding	2	0.66007	0.09197	GSS, MGST2	253	7	18413	20.7939	1	0.81212	0.79824

Category	Term	Count	%	PValue	Genes	List Total	Pop Hits	Pop Total	Fold Enric	Bonferron	Benjamini	FDR
KEGG_PATHWAY	bta01100:Metabolic pathways	77	25.4125	8.63E-14	AHCYL2, PANK3, PANK1, HEXB, ENO1, ENO2, ENO3, GPHN, AACS, SGSH, GMPPB, PNP, NAPRT, LOC538702, DBT, GMPPA, PDE4B, PGM3, AOX1, PDE4A, PGM1, PSPH, CAD, NME2, CTPS1, KMO, HADHB, CYP27A1, BDH1, EHHADH, PIK3C3, LAP3, HAGH, FBP1, FBP2, GNE, PRPS2, PRPS1, IDI1, MVK, ACSS2, MGST3, ALOX15	193	1594	9288	2.3247	2.29E-11	2.29E-11	2.03E-11
KEGG_PATHWAY	bta01200:Carbon metabolism	18	5.94059	9.42E-11	PRPS2, PRPS1, ACSS2, MDH1, IDH1, ENO1, ENO2, ENO3, GCK, ACAT2, RPIA, PFKL, PSAT1, ACO1, FBP1, PFKM, FBP2, PSPH	193	111	9288	7.80395	2.50E-08	1.06E-08	9.39E-09
KEGG_PATHWAY	bta03050:Proteasome	13	4.29043	1.20E-10	PSMD14, ADRM1, PSMB6, PSMB7, PSMB4, PSMC6, PSMD7, PSMA4, PSMB5, PSMC3, PSMA2, PSMB3, PSMD3	193	47	9288	13.311	3.18E-08	1.06E-08	9.39E-09
KEGG_PATHWAY	bta01230:Biosynthesis of amino acids	14	4.62046	2.44E-09	PRPS2, PRPS1, IDH1, ENO1, ENO2, ENO3, ASS1, RPIA, PFKL, PSAT1, PAH, ACO1, PFKM, PSPH	193	73	9288	9.22933	6.47E-07	1.62E-07	1.43E-07
KEGG_PATHWAY	bta00010:Glycolysis / Gluconeogenesis	12	3.9604	6.33E-08	LDHB, PFKL, ACSS2, GALM, ENO1, ENO2, ENO3, PFKM, FBP1, FBP2, GCK, PGM1	193	64	9288	9.02332	1.68E-05	2.91E-06	2.58E-06
KEGG_PATHWAY	bta01250:Biosynthesis of nucleotide sugars	10	3.30033	7.09E-08	GMPPB, GALE, UGP2, GFPT2, PMM2, GMPPA, PGM3, GCK, PGM1, GNE	193	39	9288	12.3396	1.88E-05	2.91E-06	2.58E-06
KEGG_PATHWAY	bta00520:Amino sugar and nucleotide sugar metabolism	11	3.63036	8.39E-08	GMPPB, GALE, UGP2, HEXB, GFPT2, PMM2, GMPPA, PGM3, GCK, PGM1, GNE	193	52	9288	10.1802	2.22E-05	2.91E-06	2.58E-06
KEGG_PATHWAY	bta03320:PPAR signaling pathway	13	4.29043	8.78E-08	GK, DBI, SORBS1, GK2, CYP27A1, LOC518526, FABP3, FABP4, SCD, EHHADH, LOC538702, ANGPTL4, CD36	193	81	9288	7.72366	2.33E-05	2.91E-06	2.58E-06
KEGG_PATHWAY	bta05017:Spinocerebellar ataxia	16	5.28053	3.08E-07	PSMD14, ADRM1, PSMB6, PSMB7, PSMB4, PSMC6, PSMD7, PSMA4, PSMB5, PSMC3, OPA1, PSMA2, PSMB3, PSMD3, PIK3C3, SLC25A5	193	146	9288	5.2739	8.16E-05	9.07E-06	8.04E-06
KEGG_PATHWAY	bta01240:Biosynthesis of cofactors	16	5.28053	4.39E-07	PANK3, PANK1, GSS, CAD, PMM2, NME2, CTPS1, KMO, GPHN, GMPPB, UGP2, PSAT1, NAPRT, GMPPA, ALPL, GCLM	193	150	9288	5.13326	1.16E-04	1.16E-05	1.03E-05
KEGG_PATHWAY	bta05016:Huntington disease	23	7.59076	5.51E-07	DCTN6, DCTN5, DCTN2, PSMD14, NDUFA4, DCTN3, ADRM1, CLTB, SOD1, PSMB6, PSMB7, ACTR1A, PSMB4, PSMC6, PSMD7, PSMA4, PSMB5, PSMC3, PSMA2, PSMB3, PSMD3, PIK3C3, SLC25A5	193	316	9288	3.50272	1.46E-04	1.33E-05	1.18E-05
KEGG_PATHWAY	bta05014:Amyotrophic lateral sclerosis	25	8.25083	9.13E-07	DCTN6, DCTN5, PSMD14, DCTN2, DCTN3, PSMB6, PPP3CA, PSMB7, ACTR1A, PSMB4, PSMD7, PPP3CC, PSMB5, PSMB3, PSMD3, RANBP2, NDUFA4, ADRM1, SOD1, MAPK13, PSMA4, PSMC6, PSMC3, PSMA2, PIK3C3	193	379	9288	3.17443	2.42E-04	2.02E-05	1.79E-05
KEGG_PATHWAY	bta05020:Prion disease	21	6.93069	1.20E-06	PSMD14, NDUFA4, ADRM1, LAMC1, SOD1, MAPK13, PSMB6, PPP3CA, PSMB7, PSMB4, PSMC6, PSMD7, PSMA4, PSMB5, PPP3CC, PSMC3, PSMA2, PSMB3, PSMD3, MAPK1, SLC25A5	193	279	9288	3.62226	3.17E-04	2.44E-05	2.17E-05
KEGG_PATHWAY	bta00030:Pentose phosphate pathway	8	2.64026	1.54E-06	PRPS2, RPIA, PRPS1, PFKL, PFKM, FBP1, FBP2, PGM1	193	29	9288	13.2757	4.07E-04	2.91E-05	2.58E-05
KEGG_PATHWAY	bta05022:Pathways of neurodegeneration - multiple diseases	28	9.24092	2.41E-06	DCTN6, DCTN5, PSMD14, DCTN2, DCTN3, PSMB6, PPP3CA, PSMB7, ACTR1A, PSMB4, PSMD7, PPP3CC, PSMB5, PSMB3, PSMD3, MAPK1, MAP2K2, NDUFA4, ADRM1, UBE2G1, SOD1, MAPK13, PSMA4, PSMC6, PSMC3, PSMA2, PIK3C3, SLC25A5	193	487	9288	2.7669	6.38E-04	4.25E-05	3.77E-05
KEGG_PATHWAY	bta00052:Galactose metabolism	7	2.31023	3.45E-05	GALE, PFKL, UGP2, GALM, PFKM, GCK, PGM1	193	31	9288	10.8668	0.00911	5.72E-04	5.07E-04
KEGG_PATHWAY	bta00051:Fructose and mannose metabolism	7	2.31023	5.99E-05	GMPPB, PFKL, PMM2, GMPPA, PFKM, FBP1, FBP2	193	34	9288	9.90795	0.01575	9.34E-04	8.28E-04
KEGG_PATHWAY	bta05012:Parkinson disease	17	5.61056	2.21E-04	PSMD14, NDUFA4, ADRM1, UBE2G1, SOD1, PSMB6, PSMB7, PSMB4, PSMC6, PSMD7, PSMA4, PSMB5, PSMC3, PSMA2, PSMB3, PSMD3, SLC25A5	193	280	9288	2.92184	0.05685	0.00312	0.00276
KEGG_PATHWAY	bta04152:AMPK signaling pathway	11	3.63036	2.23E-04	LOC518526, PFKL, SCD, FASN, CD36, PFKM, FBP1, ACACB, ACACA, FBP2, CAMKK2	193	123	9288	4.3038	0.05748	0.00312	0.00276
KEGG_PATHWAY	bta00620:Pyruvate metabolism	7	2.31023	2.65E-04	LDHB, ACSS2, MDH1, HAGH, ACACB, ACACA, ACAT2	193	44	9288	7.65615	0.0678	0.00339	0.00301
KEGG_PATHWAY	bta04922:Glucagon signaling pathway	10	3.30033	2.69E-04	PPP3CA, LDHB, PFKL, PPP3CC, PFKM, FBP1, ACACB, ACACA, FBP2, GCK	193	103	9288	4.67227	0.06877	0.00339	0.00301
KEGG_PATHWAY	bta00640:Propanoate metabolism	6	1.9802	3.93E-04	LDHB, ACSS2, EHHADH, DBT, ACACB, ACACA	193	31	9288	9.31439	0.09897	0.00474	0.0042
KEGG_PATHWAY	bta05010:Alzheimer disease	20	6.60066	6.03E-04	MAP2K2, PSMD14, NDUFA4, ADRM1, PSMB6, PPP3CA, PSMB7, PSMB4, PSMC6, PSMD7, PSMA4, PSMB5, PPP3CC, PSMC3, PSMA2, PSMB3, PSMD3, MAPK1, PIK3C3, SLC25A5	193	400	9288	2.40622	0.14778	0.00695	0.00616
KEGG_PATHWAY	bta00900:Terpenoid backbone biosynthesis	5	1.65017	8.04E-04	FDPS, IDI1, MVK, FNTA, ACAT2	193	21	9288	11.4582	0.19195	0.00888	0.00787
KEGG_PATHWAY	bta00970:Aminoacyl-tRNA biosynthesis	7	2.31023	0.00184	GARS1, SARS1, AARS1, YARS1, HARS2, TARS1, WARS1	193	63	9288	5.34715	0.3867	0.01954	0.01733
KEGG_PATHWAY	bta04066:HIF-1 signaling pathway	9	2.9703	0.00195	LDHB, PFKL, MAP2K2, MAPK1, ENO1, ENO2, ENO3, PFKM, RBX1	193	110	9288	3.93745	0.40463	0.01993	0.01767
KEGG_PATHWAY	bta03010:Ribosome	11	3.63036	0.0022	RPL30, RPS19, RPL31, LOC101906221, RPL23A, RPL26, FAU, RPL29, RPL10A, RPS21, RPS10	193	165	9288	3.20829	0.44257	0.02094	0.01857
KEGG_PATHWAY	bta04910:Insulin signaling pathway	10	3.30033	0.00221	MAP2K2, FASN, PPP1R3D, MAPK1, SORBS1, FBP1, ACACB, ACACA, FBP2, GCK	193	138	9288	3.48727	0.44391	0.02094	0.01857
KEGG_PATHWAY	bta05230:Central carbon metabolism in cancer	7	2.31023	0.00273	LDHB, PFKL, MAP2K2, IDH1, MAPK1, PFKM, GCK	193	68	9288	4.95398	0.51505	0.02492	0.0221
KEGG_PATHWAY	bta00280:Valine, leucine and isoleucine degradation	6	1.9802	0.00357	HADHB, EHHADH, DBT, AOX1, AACS, ACAT2	193	50	9288	5.77492	0.61258	0.03155	0.02798
KEGG_PATHWAY	bta00270:Cysteine and methionine metabolism	6	1.9802	0.00424	LDHB, AHCYL2, MDH1, PSAT1, GSS, GCLM	193	52	9288	5.55281	0.67554	0.03623	0.03213
KEGG_PATHWAY	bta01212:Fatty acid metabolism	6	1.9802	0.00677	HADHB, SCD, EHHADH, FASN, ACACA, ACAT2	193	58	9288	4.97838	0.83455	0.05603	0.04969
KEGG_PATHWAY	bta00480:Glutathione metabolism	6	1.9802	0.00836	MGST3, IDH1, GSS, MGST2, LAP3, GCLM	193	61	9288	4.73354	0.8919	0.06713	0.05953
KEGG_PATHWAY	bta05171:Coronavirus disease - COVID-19	13	4.29043	0.01265	RPL30, RPL31, LOC101906221, RPL23A, RPL10A, MAPK13, RPS19, MAPK1, RPL26, FAU, RPL29, RPS21, RPS10	193	278	9288	2.25042	0.96574	0.0986	0.08744
KEGG_PATHWAY	bta00650:Butanoate metabolism	4	1.32013	0.01766	BDH1, EHHADH, AACS, ACAT2	193	27	9288	7.12953	0.99111	0.13373	0.11859
KEGG_PATHWAY	bta05132:Salmonella infection	12	3.9604	0.01851	DCTN6, DYNC1I2, MAP2K4, DCTN5, ACTR1A, MAP2K2, DCTN2, DCTN3, MAPK1, PIK3C3, KPNA1, MAPK13	193	259	9288	2.2297	0.99293	0.13626	0.12083
KEGG_PATHWAY	bta00230:Purine metabolism	8	2.64026	0.01952	PRPS2, PRPS1, NT5E, PNP, NME2, PDE4B, PDE4A, PGM1	193	132	9288	2.91663	0.99462	0.13982	0.124
KEGG_PATHWAY	bta03018:RNA degradation	6	1.9802	0.02244	PFKL, ENO1, ENO2, ENO3, PFKM, HSPD1	193	78	9288	3.70187	0.99756	0.15647	0.13876
KEGG_PATHWAY	bta00630:Glyoxylate and dicarboxylate metabolism	4	1.32013	0.02344	ACSS2, MDH1, ACO1, ACAT2	193	30	9288	6.41658	0.99814	0.15925	0.14123
KEGG_PATHWAY	bta04370:VEGF signaling pathway	5	1.65017	0.03158	PPP3CA, MAP2K2, PPP3CC, MAPK1, MAPK13	193	58	9288	4.14865	0.9998	0.20919	0.18551
KEGG_PATHWAY	bta00760:Nicotinate and nicotinamide metabolism	4	1.32013	0.04322	NT5E, PNP, NAPRT, AOX1	193	38	9288	5.06572	0.99999	0.27272	0.24185
KEGG_PATHWAY	bta00790:Folate biosynthesis	4	1.32013	0.04322	QDPR, PAH, ALPL, GPHN	193	38	9288	5.06572	0.99999	0.27272	0.24185
KEGG_PATHWAY	bta00061:Fatty acid biosynthesis	3	0.9901	0.05236	FASN, ACACB, ACACA	193	18	9288	8.02073	1	0.32268	0.28615
KEGG_PATHWAY	bta05208:Chemical carcinogenesis - reactive oxygen species	10	3.30033	0.05982	MAP2K4, MAP2K2, NDUFA4, MGST3, MGST2, MAPK1, LPO, SLC25A5, SOD1, MAPK13	193	239	9288	2.01357	1	0.36031	0.31952
KEGG_PATHWAY	bta04141:Protein processing in endoplasmic reticulum	8	2.64026	0.06419	ERLEC1, TRAM1, NPLOC4, PREB, UBE2G1, SEC61B, RAD23B, RBX1	193	171	9288	2.25143	1	0.37799	0.3352
KEGG_PATHWAY	bta04144:Endocytosis	10	3.30033	0.06787	WASHC4, ARFGEF2, SH3GLB2, ITCH, WIPF2, CLTB, VPS45, STAMBP, SNF8, FOLR1	193	245	9288	1.96426	1	0.38965	0.34554
KEGG_PATHWAY	bta00380:Tryptophan metabolism	4	1.32013	0.06911	EHHADH, AOX1, KMO, ACAT2	193	46	9288	4.18473	1	0.38965	0.34554
KEGG_PATHWAY	bta04931:Insulin resistance	6	1.9802	0.07515	PTPA, LOC518526, GFPT2, PPP1R3D, CD36, ACACB	193	109	9288	2.64905	1	0.41352	0.36671
KEGG_PATHWAY	bta04979:Cholesterol metabolism	4	1.32013	0.07646	CYP27A1, LOC518526, CD36, ANGPTL4	193	48	9288	4.01036	1	0.41352	0.36671
KEGG_PATHWAY	bta04962:Vasopressin-regulated water reabsorption	4	1.32013	0.08026	DCTN6, DYNC1I2, DCTN5, DCTN2	193	49	9288	3.92852	1	0.42539	0.37724
KEGG_PATHWAY	bta04936:Alcoholic liver disease	7	2.31023	0.09735	MAP2K4, SCD, FASN, ACACB, ACACA, MAPK13, CAMKK2	193	153	9288	2.20177	1	0.50583	0.44857

D1vsD15 Down-regulated

Category	Term	Count	%	PValue	Genes	List Total	Pop Hits	Pop Total	Fold Enricl	Bonferron	Benjamini	FDR
GOTERM_BP_DIRECT	GO:0000398~mRNA splicing, via spliceosome	25	5.980861	1.18E-13	SF3B2, SF3B6, EIF4A3, SNU13, SNRPD2, U2AF2, TRA2B, DHX16, SNRPB2, SRSF10, SF3A1, SNRPN, MAGOHB, PPP1CA, HNRNPM, LSM8, LSM7, DDX39A, PRPF6, DDX39B, HNRNPA2B1, SRSF2, SRSF4, SRSF7, SNRPB	385	156	16929	7.046703	2.08E-10	2.08E-10	2.06E-10
GOTERM_BP_DIRECT	GO:0006099~tricarboxylic acid cycle	9	2.15311	4.26E-07	SUCLA2, IDH2, OGDH, DLST, SDHC, OGDHL, PDHB, SDHB, IDH3A	385	32	16929	12.36696	7.49E-04	3.75E-04	3.72E-04
GOTERM_BP_DIRECT	GO:0090131~mesenchyme migration	5	1.196172	8.64E-06	ACTA2, ACTA1, ACTC1, ACTB, ACTG2	385	7	16929	31.40816	0.015104	0.005073	0.00503
GOTERM_BP_DIRECT	GO:0072659~protein localization to plasma membrane	13	3.110048	3.22E-05	SEC23A, NHERF1, CD81, RDX, ATP1B3, EHD1, RAP1A, CDH1, FLOT1, FLNA, FLOT2, RAB8A, S100A10	385	127	16929	4.501012	0.055127	0.014176	0.014055
GOTERM_BP_DIRECT	GO:0061640~cytoskeleton-dependent cytokinesis	5	1.196172	2.92E-04	SEPTIN2, SEPTIN6, SEPTIN9, SEPTIN7, SEPTIN11	385	15	16929	14.65714	0.401808	0.102754	0.101879
GOTERM_BP_DIRECT	GO:0006397~mRNA processing	11	2.631579	4.07E-04	HNRNPL, RBM39, HNRNPK, U2AF2, HNRNPF, HNRNPA2B1, MAGOH, TARDBP, MAGOHB, RBMX, DCPS	385	120	16929	4.030714	0.511334	0.104443	0.103554
GOTERM_BP_DIRECT	GO:0048025~negative regulation of mRNA splicing, via spliceosome	5	1.196172	4.91E-04	HNRNPK, U2AF2, SRSF4, RBMX, SRSF7	385	17	16929	12.93277	0.578691	0.104443	0.103554
GOTERM_BP_DIRECT	GO:0010873~positive regulation of cholesterol esterification	4	0.956938	5.96E-04	APOA1, APOA4, APOE, AGT	385	8	16929	21.98571	0.649871	0.104443	0.103554
GOTERM_BP_DIRECT	GO:0030240~skeletal muscle thin filament assembly	4	0.956938	5.96E-04	ACTA2, ACTA1, ACTC1, ACTB	385	8	16929	21.98571	0.649871	0.104443	0.103554
GOTERM_BP_DIRECT	GO:0007015~actin filament organization	11	2.631579	6.37E-04	TF, MARCKS, MYO1C, ACTC1, POF1B, TPM1, TMOD2, CTNNA1, CTNNA3, CORO1B, MYO1F	385	127	16929	3.808549	0.674419	0.104443	0.103554
GOTERM_BP_DIRECT	GO:0000381~regulation of alternative mRNA splicing, via spliceosome	8	1.913876	6.57E-04	DDX17, DDX5, TRA2B, EIF4A3, MAGOH, SRSF2, MAGOHB, RBMX	385	65	16929	5.411868	0.685613	0.104443	0.103554
GOTERM_BP_DIRECT	GO:0034314~Arp2/3 complex-mediated actin nucleation	5	1.196172	7.71E-04	ACTR3, ARPC2, ARPC1B, ARPC4, IQGAP2	385	19	16929	11.57143	0.742899	0.104443	0.103554
GOTERM_BP_DIRECT	GO:0006268~DNA unwinding involved in DNA replication	5	1.196172	7.71E-04	MCM7, MCM3, RPA1, MCM5, MCM6	385	19	16929	11.57143	0.742899	0.104443	0.103554
GOTERM_BP_DIRECT	GO:0098609~cell-cell adhesion	10	2.392344	9.56E-04	LGALS1, CTNNA1, MYH9, CTNNA3, CTNNA2, DSC1, LPP, TJP3, PPP1CA, TJP2	385	111	16929	3.96139	0.814289	0.120197	0.119173
GOTERM_BP_DIRECT	GO:0051028~mRNA transport	6	1.435407	0.001403	MYO1C, EIF4A3, MAGOH, NUP98, MAGOHB, SRSF7	385	37	16929	7.130502	0.915637	0.162077	0.160696
GOTERM_BP_DIRECT	GO:0000226~microtubule cytoskeleton organization	10	2.392344	0.001473	TUBA1C, TUBA3E, TUBB2B, TUBB2A, PRC1, TBCD, MAP4, TUBB4B, TUBA4A, MARK2	385	118	16929	3.726392	0.925365	0.162077	0.160696
GOTERM_BP_DIRECT	GO:0007163~establishment or maintenance of cell polarity	6	1.435407	0.001586	ACTR3, PARD6B, PARD6A, STK25, CTNNA1, ACTB	385	38	16929	6.942857	0.938903	0.164298	0.162899
GOTERM_BP_DIRECT	GO:0034613~cellular protein localization	7	1.674641	0.002142	SEPTIN2, CTNNA1, SEPTIN6, LAMTOR2, SEPTIN9, SEPTIN7, SEPTIN11	385	59	16929	5.216949	0.977089	0.192609	0.190968
GOTERM_BP_DIRECT	GO:0000727~double-strand break repair via break-induced replication	4	0.956938	0.002187	MCM7, MCM3, MCM5, MCM6	385	12	16929	14.65714	0.978856	0.192609	0.190968
GOTERM_BP_DIRECT	GO:0030007~cellular potassium ion homeostasis	4	0.956938	0.002187	ATP1A4, ATP1B3, ATP1A2, ATP1A1	385	12	16929	14.65714	0.978856	0.192609	0.190968
GOTERM_BP_DIRECT	GO:0048260~positive regulation of receptor-mediated endocytosis	4	0.956938	0.002796	C3, TF, HNRNPK, CLU	385	13	16929	13.52967	0.992781	0.194843	0.193183
GOTERM_BP_DIRECT	GO:0036376~sodium ion export from cell	4	0.956938	0.002796	ATP1A4, ATP1B3, ATP1A2, ATP1A1	385	13	16929	13.52967	0.992781	0.194843	0.193183
GOTERM_BP_DIRECT	GO:0010761~fibroblast migration	4	0.956938	0.002796	SGPL1, NHERF1, LAMTOR2, IQGAP1	385	13	16929	13.52967	0.992781	0.194843	0.193183
GOTERM_BP_DIRECT	GO:2000622~regulation of nuclear-transcribed mRNA catabolic process, nonsense-mediated decay	3	0.717703	0.002987	EIF4A3, MAGOH, MAGOHB	385	4	16929	32.97857	0.994849	0.194843	0.193183
GOTERM_BP_DIRECT	GO:0090168~Golgi reassembly	3	0.717703	0.002987	VCPIP1, STK25, PDCD10	385	4	16929	32.97857	0.994849	0.194843	0.193183
GOTERM_BP_DIRECT	GO:0010248~establishment or maintenance of transmembrane electrochemical gradient	3	0.717703	0.002987	BAX, ATP1A2, ATP1A1	385	4	16929	32.97857	0.994849	0.194843	0.193183
GOTERM_BP_DIRECT	GO:0006104~succinyl-CoA metabolic process	3	0.717703	0.002987	SUCLA2, OGDH, DLST	385	4	16929	32.97857	0.994849	0.194843	0.193183
GOTERM_BP_DIRECT	GO:0007017~microtubule-based process	5	1.196172	0.003952	TUBA3E, TUBB2B, TUBB2A, TUBB4B, TUBA4A	385	29	16929	7.581281	0.999063	0.248542	0.246425
GOTERM_BP_DIRECT	GO:0008217~regulation of blood pressure	6	1.435407	0.004105	ACTA2, GNA11, STK39, ATP1A2, ATP1A1, ACTB	385	47	16929	5.613374	0.999285	0.249273	0.24715
GOTERM_BP_DIRECT	GO:0008380~RNA splicing	8	1.913876	0.004356	DDX5, HNRNPK, U2AF2, HNRNPF, MAGOH, TARDBP, MAGOHB, RBMX	385	90	16929	3.908571	0.999541	0.254015	0.251852
GOTERM_BP_DIRECT	GO:0032456~endocytic recycling	6	1.435407	0.004499	SNX3, EHD1, SNX27, CMTM6, VPS35L, ARF6	385	48	16929	5.496429	0.999644	0.254015	0.251852
GOTERM_BP_DIRECT	GO:0097242~beta-amyloid clearance	3	0.717703	0.004904	C3, MME, C5AR1	385	5	16929	26.38286	0.999826	0.254015	0.251852
GOTERM_BP_DIRECT	GO:0051918~negative regulation of fibrinolysis	3	0.717703	0.004904	PLG, HRG, THBS1	385	5	16929	26.38286	0.999826	0.254015	0.251852
GOTERM_BP_DIRECT	GO:0014829~vascular smooth muscle contraction	3	0.717703	0.004904	ACTA2, ACTB, ACTG2	385	5	16929	26.38286	0.999826	0.254015	0.251852
GOTERM_BP_DIRECT	GO:1902600~hydrogen ion transmembrane transport	7	1.674641	0.006215	ATP6AP2, ATP1A4, ATP1A2, ATP1A1, ATP6V0C, COX5A, ATP6V0A1	385	73	16929	4.216438	0.999983	0.310211	0.307569
GOTERM_BP_DIRECT	GO:1900026~positive regulation of substrate adhesion-dependent cell spreading	5	1.196172	0.006342	ARPC2, FLNA, APOA1, UNC13D, S100A10	385	33	16929	6.662338	0.999986	0.310211	0.307569
GOTERM_BP_DIRECT	GO:0050821~protein stabilization	10	2.392344	0.006985	PDCD10, GNAQ, LAMP2, FLOT1, FLOT2, FLNA, APOA1, ATP1B3, CLU, RTN4	385	149	16929	2.951103	0.999996	0.322941	0.320191
GOTERM_BP_DIRECT	GO:0051683~establishment of Golgi localization	3	0.717703	0.007246	NHERF1, STK25, PDCD10	385	6	16929	21.98571	0.999997	0.322941	0.320191
GOTERM_BP_DIRECT	GO:1905605~positive regulation of maintenance of permeability of blood-brain barrier	3	0.717703	0.007246	OCLN, TJP3, TJP2	385	6	16929	21.98571	0.999997	0.322941	0.320191
GOTERM_BP_DIRECT	GO:0006883~cellular sodium ion homeostasis	4	0.956938	0.007335	ATP1A4, ATP1B3, ATP1A2, ATP1A1	385	18	16929	9.771429	0.999998	0.322941	0.320191
GOTERM_BP_DIRECT	GO:0007188~adenylate cyclase-modulating G-protein coupled receptor signaling pathway	5	1.196172	0.009536	GNA14, GNAL, GNAQ, GNA11, GNAI3	385	37	16929	5.942085	1	0.409269	0.405783
GOTERM_BP_DIRECT	GO:0034380~high-density lipoprotein particle assembly	3	0.717703	0.009994	APOA1, APOA4, APOE	385	7	16929	18.8449	1	0.409269	0.405783
GOTERM_BP_DIRECT	GO:0034372~very-low-density lipoprotein particle remodeling	3	0.717703	0.009994	APOA1, APOA4, APOE	385	7	16929	18.8449	1	0.409269	0.405783
GOTERM_BP_DIRECT	GO:0006270~DNA replication initiation	4	0.956938	0.011371	MCM7, MCM3, MCM5, MCM6	385	21	16929	8.37551	1	0.444994	0.441204
GOTERM_BP_DIRECT	GO:0010592~positive regulation of lamellipodium assembly	4	0.956938	0.011371	ACTR3, MSTN, ARPC2, TWF2	385	21	16929	8.37551	1	0.444994	0.441204
GOTERM_BP_DIRECT	GO:0006509~membrane protein ectodomain proteolysis	4	0.956938	0.012949	DAG1, ADAM10, MYH9, RBMX	385	22	16929	7.994805	1	0.481564	0.477462
GOTERM_BP_DIRECT	GO:1902669~positive regulation of axon guidance	3	0.717703	0.013126	TUBB2B, TUBB2A, TUBB4B	385	8	16929	16.48929	1	0.481564	0.477462
GOTERM_BP_DIRECT	GO:0045324~late endosome to vacuole transport	3	0.717703	0.013126	CHMP2B, PIK3R4, CHMP3	385	8	16929	16.48929	1	0.481564	0.477462
GOTERM_BP_DIRECT	GO:1990573~potassium ion import across plasma membrane	5	1.196172	0.013625	SLC12A2, ATP1A4, ATP1B3, ATP1A2, ATP1A1	385	41	16929	5.362369	1	0.489683	0.485512
GOTERM_BP_DIRECT	GO:0042752~regulation of circadian rhythm	5	1.196172	0.01603	PSPC1, CDK1, HNRNPD, TARDBP, PPP1CA	385	43	16929	5.112957	1	0.552402	0.547696
GOTERM_BP_DIRECT	GO:0000278~mitotic cell cycle	7	1.674641	0.016535	TUBA1C, TUBA3E, TUBB2B, TUBB2A, TBCD, TUBB4B, TUBA4A	385	90	16929	3.42	1	0.552402	0.547696
GOTERM_BP_DIRECT	GO:0060158~phospholipase C-activating dopamine receptor signaling pathway	3	0.717703	0.016625	GNA14, NHERF1, GNA11	385	9	16929	14.65714	1	0.552402	0.547696
GOTERM_BP_DIRECT	GO:0033539~fatty acid beta-oxidation using acyl-CoA dehydrogenase	3	0.717703	0.016625	ACADVL, ACADM, ACADS	385	9	16929	14.65714	1	0.552402	0.547696
GOTERM_BP_DIRECT	GO:0006260~DNA replication	6	1.435407	0.017924	RRM1, SUPT16H, RBBP4, NFIB, RPA1, RBBP7	385	67	16929	3.93774	1	0.584538	0.579559
GOTERM_BP_DIRECT	GO:0043691~reverse cholesterol transport	3	0.717703	0.020473	APOA1, APOA4, APOE	385	10	16929	13.19143	1	0.621608	0.616313
GOTERM_BP_DIRECT	GO:0006103~2-oxoglutarate metabolic process	3	0.717703	0.020473	IDH2, OGDH, DLST	385	10	16929	13.19143	1	0.621608	0.616313
GOTERM_BP_DIRECT	GO:0007191~adenylate cyclase-activating dopamine receptor signaling pathway	3	0.717703	0.020473	NHERF1, GNAL, GNB1	385	10	16929	13.19143	1	0.621608	0.616313
GOTERM_BP_DIRECT	GO:0044319~wound healing, spreading of cells	3	0.717703	0.020473	PDCD10, FLNA, CD44	385	10	16929	13.19143	1	0.621608	0.616313
GOTERM_BP_DIRECT	GO:0043484~regulation of RNA splicing	5	1.196172	0.021598	HNRNPL, HNRNPH1, HNRNPF, TRA2B, HNRNPH3	385	47	16929	4.677812	1	0.644642	0.639151
GOTERM_BP_DIRECT	GO:0042981~regulation of apoptotic process	8	1.913876	0.022089	APEX1, ANP32E, BAX, ANP32B, APOE, TNFAIP8L2, TARDBP, CLU	385	123	16929	2.85993	1	0.646014	0.640511
GOTERM_BP_DIRECT	GO:0015031~protein transport	13	3.110048	0.022378	STXBP2, CMTM6, ACTN4, LIN7C, SNX3, MYO1C, CHMP2B, MYH9, CHMP3, KIF20A, NUP98, SIL1, VPS35L	385	273	16929	2.093878	1	0.646014	0.640511
GOTERM_BP_DIRECT	GO:0030048~actin filament-based movement	3	0.717703	0.024652	MYH14, MYH9, MYH10	385	11	16929	11.99221	1	0.686076	0.680232
GOTERM_BP_DIRECT	GO:0051764~actin crosslink formation	3	0.717703	0.024652	MARCKS, FLNA, BAIAP2	385	11	16929	11.99221	1	0.686076	0.680232
GOTERM_BP_DIRECT	GO:0051017~actin filament bundle assembly	4	0.956938	0.024934	MARCKS, CALD1, BAIAP2, CORO1B	385	28	16929	6.281633	1	0.686076	0.680232
GOTERM_BP_DIRECT	GO:0009615~response to virus	5	1.196172	0.026461	ACTA2, HNRNPUL1, HSPB1, DDX21, ACTB	385	50	16929	4.397143	1	0.708416	0.702382
GOTERM_BP_DIRECT	GO:2001244~positive regulation of intrinsic apoptotic signaling pathway	4	0.956938	0.027355	BCLAF1, NHERF1, BAX, CLU	385	29	16929	6.065025	1	0.708416	0.702382
GOTERM_BP_DIRECT	GO:0031032~actomyosin structure organization	4	0.956938	0.027355	MYH14, MYH9, MYH10, MYL9	385	29	16929	6.065025	1	0.708416	0.702382
GOTERM_BP_DIRECT	GO:0016925~protein sumoylation	4	0.956938	0.027355	UBE2I, RANGAP1, SAE1, LOC112446383	385	29	16929	6.065025	1	0.708416	0.702382
GOTERM_BP_DIRECT	GO:0007159~leukocyte cell-cell adhesion	3	0.717703	0.029145	APOA4, ITGAL, FERMT3	385	12	16929	10.99286	1	0.722876	0.716718
GOTERM_BP_DIRECT	GO:0051693~actin filament capping	3	0.717703	0.029145	SCIN, RDX, SPTAN1	385	12	16929	10.99286	1	0.722876	0.716718
GOTERM_BP_DIRECT	GO:0001508~action potential	3	0.717703	0.029145	GNA14, GNAQ, GNA11	385	12	16929	10.99286	1	0.722876	0.716718
GOTERM_BP_DIRECT	GO:0001578~microtubule bundle formation	4	0.956938	0.032559	CLIP1, PRC1, KIF20A, MAPRE1	385	31	16929	5.673733	1	0.786323	0.779625
GOTERM_BP_DIRECT	GO:0046889~positive regulation of lipid biosynthetic process	3	0.717703	0.033936	APOA1, APOA4, APOE	385	13	16929	10.14725	1	0.786323	0.779625
GOTERM_BP_DIRECT	GO:0002026~regulation of the force of heart contraction	3	0.717703	0.033936	MYL3, ATP1A2, ATP1A1	385	13	16929	10.14725	1	0.786323	0.779625
GOTERM_BP_DIRECT	GO:0030520~intracellular estrogen receptor signaling pathway	3	0.717703	0.033936	DDX17, DDX5, SAFB	385	13	16929	10.14725	1	0.786323	0.779625
GOTERM_BP_DIRECT	GO:0033700~phospholipid efflux	3	0.717703	0.033936	APOA1, APOA4, APOE	385	13	16929	10.14725	1	0.786323	0.779625
GOTERM_BP_DIRECT	GO:0010976~positive regulation of neuron projection development	5	1.196172	0.035915	SNX3, RAP1A, TWF2, APOE, MARK2	385	55	16929	3.997403	1	0.821389	0.814393
GOTERM_BP_DIRECT	GO:0042730~fibrinolysis	3	0.717703	0.039008	PLG, HRG, F2	385	14	16929	9.422449	1	0.869539	0.862132
GOTERM_BP_DIRECT	GO:1900016~negative regulation of cytokine production involved in inflammatory response	3	0.717703	0.039008	CHID1, PDCD4, F2	385	14	16929	9.422449	1	0.869539	0.862132
GOTERM_BP_DIRECT	GO:0031175~neuron projection development	6	1.435407	0.042141	EHD1, STMN1, APOE, MAP4, ATXN10, MANF	385	84	16929	3.140816	1	0.918438	0.910615
GOTERM_BP_DIRECT	GO:0010759~positive regulation of macrophage chemotaxis	3	0.717703	0.044348	MSTN, C5AR1, MAPK3	385	15	16929	8.794286	1	0.918438	0.910615
GOTERM_BP_DIRECT	GO:0022617~extracellular matrix disassembly	3	0.717703	0.044348	FLOT1, PLG, LAMC1	385	15	16929	8.794286	1	0.918438	0.910615
GOTERM_BP_DIRECT	GO:0036481~intrinsic apoptotic signaling pathway in response to hydrogen peroxide	2	0.478469	0.044853	STK25, PDCD10	385	2	16929	43.97143	1	0.918438	0.910615
GOTERM_BP_DIRECT	GO:1903416~response to glycoside	2	0.478469	0.044853	ATP1A2, ATP1A1	385	2	16929	43.97143	1	0.918438	0.910615
GOTERM_BP_DIRECT	GO:0034635~glutathione transport	2	0.478469	0.044853	NHERF1, MGST1	385	2	16929	43.97143	1	0.918438	0.910615
GOTERM_BP_DIRECT	GO:0032231~regulation of actin filament bundle assembly	2	0.478469	0.044853	RDX, FLNA	385	2	16929	43.97143	1	0.918438	0.910615
GOTERM_BP_DIRECT	GO:0060271~cilium assembly	9	2.15311	0.049042	EHD1, ACTR3, SEPTIN2, SEPTIN6, FLNA, ASAP1, ATXN10, SEPTIN7, RAB8A	385	177	16929	2.235835	1	0.97715	0.968826
GOTERM_BP_DIRECT	GO:1904355~positive regulation of telomere capping	3	0.717703	0.04994	NEK7, HNRNPD, MAPK3	385	16	16929	8.244643	1	0.97715	0.968826
GOTERM_BP_DIRECT	GO:0045851~pH reduction	3	0.717703	0.04994	ATP6AP2, ATP6V0C, ATP6V0A1	385	16	16929	8.244643	1	0.97715	0.968826
GOTERM_BP_DIRECT	GO:0002476~antigen processing and presentation of endogenous peptide antigen via MHC class Ib	3	0.717703	0.04994	BOLA-NC1, LOC616942, BOLA	385	16	16929	8.244643	1	0.97715	0.968826
GOTERM_BP_DIRECT	GO:0007409~axonogenesis	5	1.196172	0.052032	CNP, STK25, FLOT1, ACTB, RAB8A	385	62	16929	3.546083	1	1	0.992045
GOTERM_BP_DIRECT	GO:0048026~positive regulation of mRNA splicing, via spliceosome	3	0.717703	0.055769	TRA2B, NUP98, RBMX	385	17	16929	7.759664	1	1	0.992045
GOTERM_BP_DIRECT	GO:0061028~establishment of endothelial barrier	3	0.717703	0.055769	RAP1B, RAP1A, RDX	385	17	16929	7.759664	1	1	0.992045
GOTERM_BP_DIRECT	GO:1902476~chloride transmembrane transport	5	1.196172	0.059964	SLC12A2, CLIC5, CLIC4, CLIC2, CLIC1	385	65	16929	3.382418	1	1	0.992045
GOTERM_BP_DIRECT	GO:0030050~vesicle transport along actin filament	3	0.717703	0.061824	MYO1C, ACTN4, MYO1F	385	18	16929	7.328571	1	1	0.992045
GOTERM_BP_DIRECT	GO:0051016~barbed-end actin filament capping	3	0.717703	0.061824	SCIN, TWF2, ADD1	385	18	16929	7.328571	1	1	0.992045
GOTERM_BP_DIRECT	GO:0000380~alternative mRNA splicing, via spliceosome	3	0.717703	0.061824	DDX17, HNRNPM, DDX5	385	18	16929	7.328571	1	1	0.992045
GOTERM_BP_DIRECT	GO:0048709~oligodendrocyte differentiation	3	0.717703	0.061824	VTN, CNP, TRPC4	385	18	16929	7.328571	1	1	0.992045
GOTERM_BP_DIRECT	GO:0030838~positive regulation of actin filament polymerization	4	0.956938	0.065552	ARPC2, NCKAP1L, BAIAP2, ARF6	385	41	16929	4.289895	1	1	0.992045
GOTERM_BP_DIRECT	GO:0046037~GMP metabolic process	2	0.478469	0.066521	GMPR2, NT5C2	385	3	16929	29.31429	1	1	0.992045
GOTERM_BP_DIRECT	GO:1902995~positive regulation of phospholipid efflux	2	0.478469	0.066521	APOA1, APOE	385	3	16929	29.31429	1	1	0.992045
GOTERM_BP_DIRECT	GO:2001027~negative regulation of endothelial cell chemotaxis	2	0.478469	0.066521	HRG, THBS1	385	3	16929	29.31429	1	1	0.992045
GOTERM_BP_DIRECT	GO:0045822~negative regulation of heart contraction	2	0.478469	0.066521	ATP1A2, ATP1A1	385	3	16929	29.31429	1	1	0.992045
GOTERM_BP_DIRECT	GO:0001765~membrane raft assembly	2	0.478469	0.066521	FLOT2, S100A10	385	3	16929	29.31429	1	1	0.992045
GOTERM_BP_DIRECT	GO:0008360~regulation of cell shape	7	1.674641	0.06732	NHERF1, RDX, MYH9, F2, BAIAP2, MYH10, MYL12B	385	126	16929	2.442857	1	1	0.992045
GOTERM_BP_DIRECT	GO:0043113~receptor clustering	3	0.717703	0.068089	PIGR, FLNA, ITGAL	385	19	16929	6.942857	1	1	0.992045
GOTERM_BP_DIRECT	GO:0007049~cell cycle	9	2.15311	0.068499	STK10, PARD6B, PARD6A, MCM7, RBBP4, ERH, MCM5, MCM6, PPP1CA	385	190	16929	2.082857	1	1	0.992045
GOTERM_BP_DIRECT	GO:0051402~neuron apoptotic process	4	0.956938	0.069456	TRPC5, DNAJC5, GBA1, NAE1	385	42	16929	4.187755	1	1	0.992045
GOTERM_BP_DIRECT	GO:0070830~bicellular tight junction assembly	4	0.956938	0.073463	OCLN, CLDN3, POF1B, TBCD	385	43	16929	4.090365	1	1	0.992045
GOTERM_BP_DIRECT	GO:0046716~muscle cell cellular homeostasis	3	0.717703	0.074553	MSTN, LAMP2, PLG	385	20	16929	6.595714	1	1	0.992045
GOTERM_BP_DIRECT	GO:0006936~muscle contraction	4	0.956938	0.077571	CALD1, MYL1, TPM1, TMOD2	385	44	16929	3.997403	1	1	0.992045
GOTERM_BP_DIRECT	GO:0006884~cell volume homeostasis	3	0.717703	0.081204	SLC12A2, ABCB8, ADD1	385	21	16929	6.281633	1	1	0.992045
GOTERM_BP_DIRECT	GO:0070328~triglyceride homeostasis	3	0.717703	0.081204	APOA1, APOA4, APOE	385	21	16929	6.281633	1	1	0.992045
GOTERM_BP_DIRECT	GO:0033344~cholesterol efflux	3	0.717703	0.081204	APOA1, APOA4, APOE	385	21	16929	6.281633	1	1	0.992045
GOTERM_BP_DIRECT	GO:0022904~respiratory electron transport chain	3	0.717703	0.081204	AIFM2, GBA1, SDHB	385	21	16929	6.281633	1	1	0.992045
GOTERM_BP_DIRECT	GO:0009113~purine nucleobase biosynthetic process	2	0.478469	0.087699	SHMT2, PPAT	385	4	16929	21.98571	1	1	0.992045
GOTERM_BP_DIRECT	GO:0046040~IMP metabolic process	2	0.478469	0.087699	ADSS2, NT5C2	385	4	16929	21.98571	1	1	0.992045
GOTERM_BP_DIRECT	GO:1990809~endoplasmic reticulum tubular network membrane organization	2	0.478469	0.087699	ATL2, RTN4	385	4	16929	21.98571	1	1	0.992045
GOTERM_BP_DIRECT	GO:0051838~cytolysis by host of symbiont cells	2	0.478469	0.087699	HRG, F2	385	4	16929	21.98571	1	1	0.992045
GOTERM_BP_DIRECT	GO:0042167~heme catabolic process	2	0.478469	0.087699	BLVRA, HMOX2	385	4	16929	21.98571	1	1	0.992045
GOTERM_BP_DIRECT	GO:0006986~response to unfolded protein	3	0.717703	0.08803	STT3B, THBS1, MANF	385	22	16929	5.996104	1	1	0.992045

GOTERM_BP_DIRECT	GO:0006904~vesicle docking involved in exocytosis	3	0.717703	0.08803	PLEK, STXBP2, RAB8A	385	22	16929	5.996104	1	1	0.992045
GOTERM_BP_DIRECT	GO:0045216~cell-cell junction organization	3	0.717703	0.08803	FLNA, TJP3, TJP2	385	22	16929	5.996104	1	1	0.992045
GOTERM_BP_DIRECT	GO:0042157~lipoprotein metabolic process	3	0.717703	0.08803	APOA1, APOA4, APOE	385	22	16929	5.996104	1	1	0.992045
GOTERM_BP_DIRECT	GO:0016477~cell migration	9	2.15311	0.089738	SDC4, CTNNA1, CTNNA3, NCKAP1L, LAMC1, CTNNA2, MAPRE1, THBS1, CORO1B	385	202	16929	1.959123	1	1	0.992045
GOTERM_BP_DIRECT	GO:0043065~positive regulation of apoptotic process	9	2.15311	0.089738	MTCH2, SCIN, LGALS1, AIFM2, HTRA1, BAX, IRF5, HRG, CLU	385	202	16929	1.959123	1	1	0.992045
GOTERM_BP_DIRECT	GO:0045893~positive regulation of transcription, DNA-templated	12	2.870813	0.097909	ILF3, TF, MSTN, NFIB, CDH1, APEX1, SPP1, APOE, ACTN4, ILF2, AGT, UCHL5	385	311	16929	1.696647	1	1	0.992045

Category	Term	Count	%	PValue	Genes	List Total	Pop Hits	Pop Total	Fold Enric	Bonferron	Benjamini	FDR
GOTERM_CC_DIRECT	GO:0071013~catalytic step 2 spliceosome	19	4.545455	1.45E-14	HNRNPA3, DDX5, SF3B2, SF3A1, SNRPN, SF3B6, EIF4A3, MAGOHB, PRPF8, HNRNPM, LSM7, HNRNPK, PRPF6, SNRPD2, HNRNPH1, MAGOH, SNRNP200, RBMX, SNRPB	403	81	20801	12.10731	6.40E-12	6.39E-12	5.26E-12
GOTERM_CC_DIRECT	GO:0071005~U2-type precatalytic spliceosome	13	3.110048	1.12E-10	SF3B2, SF3A1, SNU13, MAGOHB, PRPF8, LSM8, LSM7, PRPF6, SNRPD2, DHX16, SNRPB2, SNRNP200, SNRPB	403	49	20801	13.69388	4.91E-08	2.46E-08	2.02E-08
GOTERM_CC_DIRECT	GO:1990904~ribonucleoprotein complex	20	4.784689	6.34E-10	DDX17, DDX5, SSB, IQGAP1, ACTN4, HNRNPAB, ELAVL1, ILF2, ACTB, HNRNPM, LSM7, ILF3, HNRNPK, HNRNPH1, HNRNPF, HNRNPA2B1, HNRNPD, HNRNPH3, NUP98, PABPC1L	403	167	20801	6.181483	2.79E-07	8.19E-08	6.74E-08
GOTERM_CC_DIRECT	GO:0001725~stress fiber	13	3.110048	7.44E-10	NEBL, SEPTIN9, LPP, SEPTIN11, CORO1B, ACTB, MYL12B, ACTA2, ACTA1, MYH14, MYH9, MYL9, MYH10	403	57	20801	11.77193	3.28E-07	8.19E-08	6.74E-08
GOTERM_CC_DIRECT	GO:0005737~cytoplasm	124	29.66507	1.03E-08	GLTP, PATJ, EIF4A3, CPOX, CLU, ACTB, IPO4, ACTG2, LGALS1, CDH1, MAGOH, STMN1, DAG1, CAST, SNRPN, FNBP1, ADAM10, ACTN4, TUBA4A, ASRGL1, ILF3, CLIP1, DDX39A, DDX39B, PSPC1, SRSF2, SRSF7, IDO1, SNRPB, CNP, SHMT2, PIK3R4, TWF2, AK4, IQGAP1, LIN7C, IQGAP2, CORO1B, SDCBP, VCPIP1, SCIN, RAP1A,	403	3943	20801	1.623208	4.54E-06	9.08E-07	7.47E-07
GOTERM_CC_DIRECT	GO:0030027~lamellipodium	16	3.827751	5.10E-08	ACTR3, TWF2, IQGAP2, CORO1B, ACTB, ACTG2, ACTA2, ACTA1, ACTC1, ARPC2, CDH1, CTNNA1, FLOT1, DAG1, FLOT2, MYH10	403	134	20801	6.163031	2.25E-05	3.74E-06	3.08E-06
GOTERM_CC_DIRECT	GO:0030175~filopodium	11	2.631579	1.25E-07	ACTA2, ACTA1, ACTC1, NHERF1, RDX, DAG1, TWF2, IQGAP2, BAIAP2, ACTB, ACTG2	403	57	20801	9.960864	5.50E-05	7.85E-06	6.46E-06
GOTERM_CC_DIRECT	GO:0005856~cytoskeleton	23	5.502392	3.92E-07	FNBP1, TPM1, RDX, TMOD2, TWF2, ACTN4, BAIAP2, SEPTIN9, SEPTIN11, CORO1B, ACTB, ADD1, ACTG2, PPP1CA, PRC1, CALD1, DAG1, CTNNA3, TAGLN2, CTNNA2, TAGLN3, SPTAN1, MAPK3	403	324	20801	3.664055	1.72E-04	2.16E-05	1.77E-05
GOTERM_CC_DIRECT	GO:0005912~adherens junction	14	3.349282	5.30E-07	RDX, TBCD, ADAM10, ACTB, ADD1, PPP1CA, POF1B, CDH1, CTNNA1, FLOT1, MYH9, FLOT2, CTNNA3, CTNNA2	403	119	20801	6.072398	2.33E-04	2.59E-05	2.13E-05
GOTERM_CC_DIRECT	GO:0005903~brush border	9	2.15311	8.12E-07	ACTR3, MYL6, MYO1C, MME, MYH14, FLNA, MYH9, ACTB, MYL12B	403	40	20801	11.61346	3.57E-04	3.39E-05	2.79E-05
GOTERM_CC_DIRECT	GO:0016607~nuclear speck	23	5.502392	8.47E-07	DDX17, RBM39, SF3B2, SF3A1, EIF4A3, MAGOHB, PPP1CA, ATXN2L, BCLAF1, DDX39A, PRPF6, DDX39B, PSPC1, U2AF2, APEX1, MAGOH, SRSF2, SNRPB2, SRSF4, SRSF7, SRSF10, LUC7L2, ATP6V0A1	403	339	20801	3.501929	3.73E-04	3.39E-05	2.79E-05
GOTERM_CC_DIRECT	GO:0030496~midbody	15	3.588517	1.14E-06	CLIC4, RALB, SEPTIN2, GNAI3, SEPTIN6, CDCA8, IQGAP1, SEPTIN7, PPP1CA, PRC1, CHMP2B, CDK1, KIF20A, MYH10, RAB8A	403	148	20801	5.231289	5.01E-04	4.08E-05	3.36E-05
GOTERM_CC_DIRECT	GO:0046540~U4/U6 x U5 tri-snRNP complex	9	2.15311	1.21E-06	LSM8, LSM7, SNRPN, PRPF6, SNRPD2, SNU13, SNRNP200, PRPF8, SNRPB	403	42	20801	11.06044	5.30E-04	4.08E-05	3.36E-05
GOTERM_CC_DIRECT	GO:0005938~cell cortex	13	3.110048	1.62E-06	CLIC5, SEPTIN2, MYL12B, MARCKS, PARD6B, SCIN, PARD6A, MYO1C, GIPC1, SPTAN1, MYL9, MYH10, ARF6	403	111	20801	6.045045	7.14E-04	5.10E-05	4.20E-05
GOTERM_CC_DIRECT	GO:0032154~cleavage furrow	9	2.15311	1.75E-06	SEPTIN2, RDX, SEPTIN6, MYH9, KIF20A, MYH10, SEPTIN7, PPP1CA, ARF6	403	44	20801	10.55769	7.70E-04	5.13E-05	4.22E-05
GOTERM_CC_DIRECT	GO:0016460~myosin II complex	5	1.196172	2.00E-06	MYH14, MYH9, MYH10, MYL9, MYL12B	403	6	20801	43.01282	8.79E-04	5.50E-05	4.52E-05
GOTERM_CC_DIRECT	GO:0016328~lateral plasma membrane	9	2.15311	7.52E-06	SLC12A2, MYO1C, CLDN3, CDH1, TBCD, ATP1A1, IQGAP1, LIN7C, MARK2	403	53	20801	8.764877	0.003303	1.95E-04	1.60E-04
GOTERM_CC_DIRECT	GO:0015630~microtubule cytoskeleton	11	2.631579	1.52E-05	TUBB2B, TUBB2A, SHMT2, SEPTIN2, PIK3R4, SEPTIN6, TUBB4B, SEPTIN9, SPTAN1, SEPTIN7, SEPTIN11	403	95	20801	5.976518	0.006679	3.72E-04	3.06E-04
GOTERM_CC_DIRECT	GO:0005923~bicellular tight junction	11	2.631579	1.67E-05	CYTH2, OCLN, PATJ, PARD6B, PARD6A, CLDN3, POF1B, TBCD, LIN7C, TJP3, TJP2	403	96	20801	5.914263	0.007327	3.87E-04	3.18E-04
GOTERM_CC_DIRECT	GO:0005874~microtubule	16	3.827751	2.80E-05	KIF23, IQGAP1, TUBB4B, SEPTIN9, TUBA4A, TUBA1C, TUBA3E, CLIP1, TUBB2B, TUBB2A, STMN1, KIF1C, KIF20A, MAP4, DYNLRB1, MAPRE1	403	221	20801	3.73686	0.012252	5.96E-04	4.90E-04
GOTERM_CC_DIRECT	GO:0016324~apical plasma membrane	17	4.066986	2.84E-05	SLC12A2, CLIC5, NHERF1, RDX, STK39, ATP1B3, ATP1A1, SEPTIN7, TF, PARD6B, MUC1, OCLN, PARD6A, MAL2, CD44, ABCG2, CTSB	403	248	20801	3.538151	0.012438	5.96E-04	4.90E-04
GOTERM_CC_DIRECT	GO:0071004~U2-type prespliceosome	6	1.435407	5.28E-05	LSM7, SF3A1, SNRPN, U2AF2, LUC7L2, SNRPB	403	22	20801	14.07692	0.022963	0.001056	8.69E-04
GOTERM_CC_DIRECT	GO:0005829~cytosol	87	20.8134	6.06E-05	GLTP, CLIC4, MCM7, ARPC1B, AKR1B1, CLU, ELAVL1, ACTB, HERC4, LGALS1, SNRPD2, AIFM2, CLIC2, CAST, ACTR3, ACTN4, RANGAP1, UNC13D, UCHL5, OAS1X, ATXN2L, MTAP, MYL6, TMEM214, HNRNPH1, DNAJC5, SRSF2, MYH9, CHMP3, PLIN2, MCM5, S100A4, BLVRA, CD44, SNRPB, SEC23A, TOMM40, HDAC1, SHMT2	403	2967	20801	1.513495	0.026319	0.001145	9.42E-04
GOTERM_CC_DIRECT	GO:0048471~perinuclear region of cytoplasm	23	5.502392	6.37E-05	SEC23A, CLIC4, NHERF1, STX7, TWF2, ACTN4, RANGAP1, SEPTIN9, CLU, CORO1B, EHD1, TF, RAP1A, CDH1, APEX1, MCM3, FLOT2, S100A4, ANP32B, ATXN10, CLIC1, ATP6V0A1, CTSB	403	446	20801	2.66178	0.027655	0.001145	9.42E-04
GOTERM_CC_DIRECT	GO:0005884~actin filament	9	2.15311	6.67E-05	ACTA2, ACTA1, ACTC1, POF1B, TPM1, TWF2, MARK2, CORO1B, ACTB	403	71	20801	6.542795	0.028921	0.001145	9.42E-04
GOTERM_CC_DIRECT	GO:0097513~myosin II filament	4	0.956938	6.96E-05	MYH14, MYH9, MYH10, MYL9	403	5	20801	41.29231	0.030164	0.001145	9.42E-04
GOTERM_CC_DIRECT	GO:0005686~U2 snRNP	7	1.674641	7.03E-05	SF3B2, SF3A1, SNRPN, SNRPD2, SF3B6, SNRPB2, SNRPB	403	37	20801	9.765073	0.030446	0.001145	9.42E-04
GOTERM_CC_DIRECT	GO:0032153~cell division site	5	1.196172	8.56E-05	SEPTIN2, SEPTIN6, SEPTIN9, SEPTIN7, SEPTIN11	403	13	20801	19.85207	0.036954	0.001215	9.99E-04
GOTERM_CC_DIRECT	GO:0005940~septin ring	5	1.196172	8.56E-05	SEPTIN2, SEPTIN6, SEPTIN9, SEPTIN7, SEPTIN11	403	13	20801	19.85207	0.036954	0.001215	9.99E-04
GOTERM_CC_DIRECT	GO:0031105~septin complex	5	1.196172	8.56E-05	SEPTIN2, SEPTIN6, SEPTIN9, SEPTIN7, SEPTIN11	403	13	20801	19.85207	0.036954	0.001215	9.99E-04
GOTERM_CC_DIRECT	GO:0016581~NuRD complex	5	1.196172	8.56E-05	HDAC2, RBBP4, HDAC1, RBBP7, MTA2	403	13	20801	19.85207	0.036954	0.001215	9.99E-04
GOTERM_CC_DIRECT	GO:0030139~endocytic vesicle	7	1.674641	1.46E-04	EHD1, OCLN, TF, STX7, FLOT2, APOA1, ARF6	403	42	20801	8.602564	0.062291	0.00201	0.001653
GOTERM_CC_DIRECT	GO:0005681~spliceosomal complex	9	2.15311	2.02E-04	DDX5, HNRNPK, DDX39B, HNRNPF, TRA2B, HNRNPA2B1, SRSF2, SNRNP200, RBMX	403	83	20801	5.596849	0.085141	0.002696	0.002218
GOTERM_CC_DIRECT	GO:0005682~U5 snRNP	6	1.435407	2.13E-04	SNRPN, PRPF6, SNRPD2, SNRNP200, PRPF8, SNRPB	403	29	20801	10.67905	0.089478	0.002757	0.002268
GOTERM_CC_DIRECT	GO:0005911~cell-cell junction	11	2.631579	2.35E-04	RAP1B, ACTR3, CLIC4, TRPC4, CDH1, MYADM, DAG1, FLNA, CSK, LIN7C, DSC1	403	131	20801	4.334116	0.098303	0.002956	0.002432
GOTERM_CC_DIRECT	GO:0005869~dynactin complex	5	1.196172	2.68E-04	ACTA2, ACTA1, ACTC1, ACTB, ACTG2	403	17	20801	15.181	0.111211	0.003274	0.002694
GOTERM_CC_DIRECT	GO:0070062~extracellular exosome	8	1.913876	2.79E-04	NAGLU, CD81, LAMP2, HNRNPA2B1, SRI, APOE, RBMX, CLIC1	403	66	20801	6.25641	0.11554	0.003318	0.00273
GOTERM_CC_DIRECT	GO:0005739~mitochondrion	36	8.61244	3.53E-04	CLIC5, MTCH2, ACADVL, CLIC4, ECHS1, SHMT2, GSTP1, MGST1, DLST, OGDHL, COX5A, CLU, AIFM2, OXCT1, ACADM, DCPS, ACADS, CLIC1, IDH3A, HSPA9, RRM1, IDH2, HSPE1, PPP1CA, ILF3, SUCLA2, P4HA1, APEX1, GPD2, OGDH, UQCRC1, VDAC2, BAX, PGAM5, UQCRC2, ADSS2	403	977	20801	1.901897	0.144022	0.004092	0.003366
GOTERM_CC_DIRECT	GO:0005902~microvillus	7	1.674641	5.35E-04	CLIC4, MYO1C, NHERF1, RDX, IQGAP2, CD44, MYO1F	403	53	20801	6.817126	0.20981	0.005782	0.004757
GOTERM_CC_DIRECT	GO:0015629~actin cytoskeleton	12	2.870813	5.37E-04	ZNF185, SCIN, MYO1C, CALD1, SEPTIN2, TPM1, CTNNA1, ACTN4, CTNNA2, BAIAP2, ACTB, MYO1F	403	172	20801	3.601073	0.210575	0.005782	0.004757
GOTERM_CC_DIRECT	GO:0016600~flotillin complex	4	0.956938	5.52E-04	CDH1, FLOT1, CTNNA1, FLOT2	403	9	20801	22.94017	0.215645	0.005782	0.004757
GOTERM_CC_DIRECT	GO:0005890~sodium:potassium-exchanging ATPase complex	4	0.956938	5.52E-04	ATP1A4, ATP1B3, ATP1A2, ATP1A1	403	9	20801	22.94017	0.215645	0.005782	0.004757
GOTERM_CC_DIRECT	GO:0005834~heterotrimeric G-protein complex	6	1.435407	6.89E-04	GNA14, GNAL, GNAQ, GNA11, GNB1, GNAI3	403	37	20801	8.370062	0.261542	0.007049	0.005799
GOTERM_CC_DIRECT	GO:0042555~MCM complex	4	0.956938	7.77E-04	MCM7, MCM3, MCM5, MCM6	403	10	20801	20.64615	0.289698	0.007771	0.006394
GOTERM_CC_DIRECT	GO:0005819~spindle	8	1.913876	0.001046	PRC1, SEPTIN2, HSPB1, SEPTIN6, MYH9, KIF20A, MAPRE1, SEPTIN7	403	82	20801	5.035647	0.36913	0.00986	0.008112
GOTERM_CC_DIRECT	GO:0005865~striated muscle thin filament	4	0.956938	0.001053	ACTA2, ACTA1, TMOD2, ACTB	403	11	20801	18.76923	0.371036	0.00986	0.008112
GOTERM_CC_DIRECT	GO:0071162~CMG complex	4	0.956938	0.001053	MCM7, MCM3, MCM5, MCM6	403	11	20801	18.76923	0.371036	0.00986	0.008112
GOTERM_CC_DIRECT	GO:0005788~endoplasmic reticulum lumen	7	1.674641	0.001137	TOR4A, OS9, P4HA1, SELENOF, UGGT1, SIL1, PDIA5	403	61	20801	5.923077	0.393803	0.010422	0.008575
GOTERM_CC_DIRECT	GO:0044297~cell body	6	1.435407	0.001384	ACTA2, CYTH2, ACTA1, ACTC1, ACTB, ACTG2	403	43	20801	7.202147	0.45624	0.012182	0.010022
GOTERM_CC_DIRECT	GO:0071006~U2-type catalytic step 1 spliceosome	4	0.956938	0.001384	EIF4A3, SNRNP200, MAGOHB, PRPF8	403	12	20801	17.20513	0.456385	0.012182	0.010022
GOTERM_CC_DIRECT	GO:0016323~basolateral plasma membrane	11	2.631579	0.001429	SLC12A2, TRPC4, CD81, C5AR1, STK39, FLOT1, FLOT2, ATP1B3, ATP1A1, LIN7C, CD44	403	165	20801	3.441026	0.466947	0.012327	0.010142
GOTERM_CC_DIRECT	GO:0016459~myosin complex	6	1.435407	0.001702	MYL6, MYO1C, MYL1, MYL3, MYH10, MYO1F	403	45	20801	6.882051	0.527354	0.014399	0.011847
GOTERM_CC_DIRECT	GO:0005885~Arp2/3 protein complex	4	0.956938	0.001774	ACTR3, ARPC2, ARPC1B, ARPC4	403	13	20801	15.88166	0.542152	0.014727	0.012116
GOTERM_CC_DIRECT	GO:0030016~myofibril	5	1.196172	0.001949	CALD1, MYL1, TPM1, TMOD2, TWF2	403	28	20801	9.217033	0.576124	0.015879	0.013064
GOTERM_CC_DIRECT	GO:0005654~nucleoplasm	62	14.83254	0.002185	ACADVL, PSIP1, ELAVL1, DAG1, DHX16, CLIC2, ACADS, SNRPN, ILF2, SDHB, UCHL5, OAS1X, ILF3, HNRNPUL1, HNRNPH1, EWSR1, S100A4, HNRNPH3, KIF20A, SNRNP200, ABCG2, DDX5, STK39, DLST, DDX21, CDCA8, ABCB8, SRI, PDHB, SAMHD1, SNX27, HNRNPDL, TRA2B, MTA2, DCPS, MARK2, TRAP1, HNRNPA3, SEPTIN2	403	2188	20801	1.462593	0.617981	0.017477	0.014379
GOTERM_CC_DIRECT	GO:0005759~mitochondrial matrix	12	2.870813	0.002396	TRAP1, ECHS1, SHMT2, CDK1, OGDH, DLST, AK4, OGDHL, ACADM, HSPE1, ACADS, IDH3A	403	207	20801	2.992196	0.652056	0.018829	0.015491
GOTERM_CC_DIRECT	GO:0016020~membrane	36	8.61244	0.002444	MTCH2, CLIC4, CNP, CD81, MGST1, PIK3R4, CPOX, LAMC1, ACTB, RAP1B, PARD6B, MUC1, PARD6A, GNA11, FLOT1, DAG1, FLOT2, CLIC2, CLIC1, CAST, CYB5A, TRPC5, NHERF1, GLYCAM1, BAIAP2, OAS1X, ATXN2L, GNAQ, DNAJC5, GNB1, NUCB1, HHIPL1, VDAC2, BAX, PLIN2, DSC1	403	1093	20801	1.700049	0.659242	0.018864	0.01552
GOTERM_CC_DIRECT	GO:0071011~precatalytic spliceosome	5	1.196172	0.002862	LSM8, SF3B2, SNRPD2, SF3B6, SNU13	403	31	20801	8.325062	0.716591	0.02134	0.017557
GOTERM_CC_DIRECT	GO:0005689~U12-type spliceosomal complex	5	1.196172	0.002862	SF3B2, LSM7, SNRPD2, SF3B6, PPP1CA	403	31	20801	8.325062	0.716591	0.02134	0.017557
GOTERM_CC_DIRECT	GO:0042995~cell projection	9	2.15311	0.003303	ACTR3, SCIN, HNRNPK, ARPC4, ATP1A2, SPTAN1, RTN4, CD44, FERMT3	403	127	20801	3.657783	0.766796	0.024224	0.01993
GOTERM_CC_DIRECT	GO:0030485~smooth muscle contractile fiber	3	0.717703	0.003585	ACTA2, ACTB, IDO1	403	5	20801	30.96923	0.794034	0.025439	0.020929
GOTERM_CC_DIRECT	GO:0045252~oxoglutarate dehydrogenase complex	3	0.717703	0.003585	OGDH, DLST, OGDHL	403	5	20801	30.96923	0.794034	0.025439	0.020929
GOTERM_CC_DIRECT	GO:0042470~melanosome	7	1.674641	0.003752	CNP, DNAJC5, FLOT1, ATP1B3, ATP1A1, ATP6V0A1, CTSB	403	77	20801	4.692308	0.808724	0.026205	0.02156
GOTERM_CC_DIRECT	GO:0031410~cytoplasmic vesicle	12	2.870813	0.003946	CLIC5, MYO1C, ATP6AP2, C5AR1, ANP32E, FLOT1, FLOT2, FAM3C, ELAVL1, AP2M1, SERPINA3-7, AGFG1	403	221	20801	2.802645	0.824428	0.026868	0.022105
GOTERM_CC_DIRECT	GO:0045121~membrane raft	9	2.15311	0.00399	MYO1C, MME, ATP1A4, FLOT1, DAG1, MAL2, ATP1A1, ABCG2, S100A10	403	131	20801	3.546095	0.82781	0.026868	0.022105
GOTERM_CC_DIRECT	GO:0005685~U1 snRNP	5	1.196172	0.00403	SNRPN, SNRPD2, SNRPB2, LUC7L2, SNRPB	403	34	20801	7.590498	0.830829	0.026868	0.022105
GOTERM_CC_DIRECT	GO:0031090~organelle membrane	8	1.913876	0.005015	MCM7, MCM3, RPA1, MCM5, ATP1A2, MCM6, ATP1A1, SUPT5H	403	108	20801	3.823362	0.890511	0.032931	0.027093
GOTERM_CC_DIRECT	GO:0098978~glutamatergic synapse	12	2.870813	0.005149	EGLN1, RAP1B, RAP1A, ACTC1, ARPC2, GIPC1, ADAM10, FLOT2, PLG, APOE, ELAVL1, ACTB	403	229	20801	2.704736	0.896845	0.033319	0.027412
GOTERM_CC_DIRECT	GO:0000776~kinetochore	8	1.913876	0.005272	SEPTIN2, CHMP2B, SEPTIN6, NUP98, RANGAP1, CKAP5, SEPTIN7, PPP1CA	403	109	20801	3.788285	0.902297	0.033619	0.027659
GOTERM_CC_DIRECT	GO:0034364~high-density lipoprotein particle	4	0.956938	0.005515	SAA3, APOA1, APOA4, APOE	403	19	20801	10.8664	0.912272	0.034668	0.028523
GOTERM_CC_DIRECT	GO:0001931~uropod	3	0.717703	0.007337	FLOT1, FLOT2, MYH9	403	7	20801	22.12088	0.960841	0.045468	0.037408
GOTERM_CC_DIRECT	GO:0070161~anchoring junction	4	0.956938	0.008399	SCIN, RAP1A, HNRNPK, ACTN4	403	22	20801	9.384615	0.975546	0.050691	0.041705
GOTERM_CC_DIRECT	GO:0001726~ruffle	6	1.435407	0.00841	MYO1C, NHERF1, MYH9, IQGAP1, BAIAP2, ARF6	403	65	20801	4.764497	0.975671	0.050691	0.041705
GOTERM_CC_DIRECT	GO:0005687~U4 snRNP	4	0.956938	0.009522	SNRPN, SNRPD2, DDX39B, SNRPB	403	23	20801	8.976589	0.98515	0.056617	0.04658
GOTERM_CC_DIRECT	GO:0097197~tetraspanin-enriched microdomain	3	0.717703	0.009658	CD81, ADAM10, SAMHD1	403	8	20801	19.35577	0.986021	0.056661	0.046616
GOTERM_CC_DIRECT	GO:0008021~synaptic vesicle	7	1.674641	0.009817	MME, SEPTIN2, GIPC1, DNAJC5, STX7, SEPTIN6, RAB8A	403	94	20801	3.843699	0.986976	0.056707	0.046654
GOTERM_CC_DIRECT	GO:0005925~focal adhesion	9	2.15311	0.009924	ARPC2, RDX, DAG1, MYH9, IQGAP1, MAPRE1, LPP, ACTB, MAPK3	403	153	20801	3.036199	0.987577	0.056707	0.046654
GOTERM_CC_DIRECT	GO:0030864~cortical actin cytoskeleton	5	1.196172	0.010146	CDH1, FLOT1, FLOT2, IQGAP1, SPTAN1	403	44	20801	5.865385	0.988744	0.057231	0.047086
GOTERM_CC_DIRECT	GO:0002102~podosome	4	0.956938	0.010729	SCIN, HNRNPK, ASAP1, FERMT3	403	24	20801	8.602564	0.991315	0.059136	0.048653
GOTERM_CC_DIRECT	GO:0045171~intercellular bridge	6	1.435407	0.010752	PRC1, SEPTIN2, CDCA8, KIF23, KIF20A, TUBB4B	403	69	20801	4.488294	0.991405	0.059136	0.048653
GOTERM_CC_DIRECT	GO:0005576~extracellular region	27	6.45933	0.01142	MSTN, PON2, LAMA4, HTRA1, PLG, PRELP, LAMC1, THBS1, KNG1, C3, DAG1, SPP1, A2M, B2M, CHID1, NHERF1, F10, CP, TCN2, MGP, HNRNPA2B1, NUCB1, VWA1, S100A4, HRG, CFB, CD44	403	833	20801	1.673008	0.993615	0.062036	0.051039
GOTERM_CC_DIRECT	GO:0030122~AP-2 adaptor complex	3	0.717703	0.01226	AP2S1, AP2A2, AP2M1	403	9	20801	17.20513	0.995607	0.065785	0.054123
GOTERM_CC_DIRECT	GO:0005770~late endosome	7	1.674641	0.01367	CHID1, TF, RAP1A, STX7, PIK3R4, LAMTOR2, MAPK3	403	101	20801	3.577304	0.997657	0.072467	0.059621
GOTERM_CC_DIRECT	GO:0042627~chylomicron	3	0.717703	0.015131	APOA1, APOA4, APOE	403	10	20801	15.48462	0.998779	0.077412	0.063689
GOTERM_CC_DIRECT	GO:0005688~U6 snRNP	3	0.717703	0.015131	LSM8, LSM7, DDX39B	403	10	20801	15.48462	0.998779	0.077412	0.063689
GOTERM_CC_DIRECT	GO:0000220~vacuolar proton-transporting V-type ATPase, V0 domain	3	0.717703	0.015131	ATP6AP2, ATP6V0C, ATP6V0A1	403	10	20801	15.48462	0.998779	0.077412	0.063689
GOTERM_CC_DIRECT	GO:0005634~nucleus	98	23.44498	0.019405	EIF4A3, CLU, ACTB, IPO4, LOC112446383, SNRPD2, AGFG1, DDX17, SNRPN, ADAM10, ACTN4, ILF3, MTAP, CLIP1, PRPF6, HNRNPUL1, EWSR1, PSPC1, MATR3, ERH, S100A4, KIF20A, TAGLN3, SRSF7, SNRPB, DDX5, SHMT2, SNU13, IQGAP1, VCPIP1, HNRNPDL, UBE2I, ARPC4, MAGOHB, SUPT5H, HNRNPL, HNRNPM, LSM8,	403	4131	20801	1.224475	0.99982	0.098141	0.080744
GOTERM_CC_DIRECT	GO:0071007~U2-type catalytic step 2 spliceosome	4	0.956938	0.023479	SNRPD2, SNRPB2, PRPF8, SNRPB	403	32	20801	6.451923	0.999971	0.117393	0.096583
GOTERM_CC_DIRECT	GO:0005743~mitochondrial inner membrane	14	3.349282	0.024333	TRAP1, MTCH2, ACADVL, TOMM40, SHMT2, IDH2, SDHC, ABCB8, COX5A, CLU, SDHB, UQCRC1, UQCRC2, SLC25A24	403	362	20801	1.996175	0.99998	0.120299	0.098974
GOTERM_CC_DIRECT	GO:0046930~pore complex	3	0.717703	0.025245	ADAM10, VDAC2, BAX	403	13	20801	11.91124	0.999987	0.122062	0.100424
GOTERM_CC_DIRECT	GO:0005684~U2-type spliceosomal complex	3	0.717703	0.025245	SF3B2, SF3A1, SF3B6	403	13	20801	11.91124	0.999987	0.122062	0.100424
GOTERM_CC_DIRECT	GO:0042584~chromaffin granule membrane	3	0.717703	0.029081	DNAJC5, SRI, ARF6	403	14	20801	11.06044	0.999998	0.139084	0.114428
GOTERM_CC_DIRECT	GO:0097225~sperm midpiece	4	0.956938	0.029702	RAP1A, NHERF1, ATP1A4, VDAC2	403	35	20801	5.898901	0.999998	0.140524	0.115613
GOTERM_CC_DIRECT	GO:0045177~apical part of cell	5	1.196172	0.030257	CLIC4, PATJ, RDX, C5AR1, MYL12B	403	61	20801	4.230769	0.999999	0.14163	0.116523
GOTERM_CC_DIRECT	GO:0000781~chromosome, telomeric region	6	1.435407	0.031668	SUN2, HDAC2, RBBP4, RBBP7, MTA2, PPP1CA	403	91	20801	3.403212	0.999999	0.14345	0.11802
GOTERM_CC_DIRECT	GO:0030018~Z disc	6	1.435407	0.031668	NEBL, HSPB1, FLNA, SRI, MYL9, MYL12B	403	91	20801	3.403212	0.999999	0.14345	0.11802
GOTERM_CC_DIRECT	GO:0001772~immunological synapse	4	0.956938	0.03195	SNX27, CD81, STX7, MYH9	403	36	20801	5.735043	0.999999	0.14345	0.11802
GOTERM_CC_DIRECT	GO:0030670~phagocytic vesicle membrane	4	0.956938	0.03195	BOLA-NC1, LAMP2, BOLA, RAB8A	403	36	20801	5.735043	0.999999	0.14345	0.11802
GOTERM_CC_DIRECT	GO:0035145~exon-exon junction complex	3	0.717703	0.033133	EIF4A3, MAGOH, MAGOHB	403	15	20801	10.32308	1	0.147259	0.121154
GOTERM_CC_DIRECT	GO:0005741~mitochondrial outer membrane	7	1.674641	0.03579	MTCH2, CYB5R3, AIFM2, MGST1, VDAC2, BAX, PPP1CA	403	126	20801	2.867521	1	0.157476	0.129559
GOTERM_CC_DIRECT	GO:0035098~ESC/E(Z) complex	3	0.717703	0.037391	HDAC2, RBBP4, RBBP7	403	16	20801	9.677885	1	0.161296	0.132702
GOTERM_CC_DIRECT	GO:0034361~very-low-density lipoprotein particle	3	0.717703	0.037391	APOA1, APOA4, APOE	403	16	20801	9.677885	1	0.161296	0.132702
GOTERM_CC_DIRECT	GO:1902560~GMP reductase complex	2	0.478469	0.038279	GMPR2, GMPR	403	2	20801	51.61538	1	0.161951	0.133242
GOTERM_CC_DIRECT	GO:1990501~exon-exon junction subcomplex mago-y14	2	0.478469	0.038279	MAGOH, MAGOHB	403	2	20801	51.61538	1	0.161951	0.133242
GOTERM_CC_DIRECT	GO:0005783~endoplasmic reticulum	21	5.023923	0.04514	CAST, CYB5A, GIMAP1, MGST1, GBA1, TAPBPL, ELAVL1, THBS1, MANF, SGPL1, OS9, ASPH, P4HA1, TMEM214, APEX1, DNAJB9, BAX, APOE, ANP32B, UGGT1, CLIC1	403	687	20801	1.577763	1	0.18916	0.155627
GOTERM_CC_DIRECT	GO:0035371~microtubule plus-end	3	0.717703	0.046487	CLIP1, MAPRE1, CKAP5	403	18	20801	8.602564	1	0.192967	0.158759
GOTERM_CC_DIRECT	GO:0043231~intracellular membrane-bounded organelle	11	2.631579	0.051158	EHD1, CYB5A, CLIC5, FNBP1, DNAJC5, AP2S1, CTNNA1, SPTAN1, EPS15, CLU, MAPK3	403	285	20801	1.992173	1	0.210369	0.173076
GOTERM_CC_DIRECT	GO:0030424~axon	9	2.15311	0.051927	MME, ATP6AP2, ADAM10, KIF1C, ATP1A1, ACADM, CTNNA2, ACTB, SEPTIN11	403	210	20801	2.212088	1	0.211553	0.174051
GOTERM_CC_DIRECT	GO:0042645~mitochondrial nucleoid	4	0.956938	0.053001	HSPA9, ACADVL, SHMT2, VDAC2	403	44	20801	4.692308	1	0.213948	0.176021
GOTERM_CC_DIRECT	GO:0034707~chloride channel complex	4	0.956938	0.059084	CLIC5, CLIC4, CLIC2, CLIC1	403	46	20801	4.488294	1	0.236338	0.194442
GOTERM_CC_DIRECT	GO:0009925~basal plasma membrane	3	0.717703	0.066755	SLC12A2, TF, MYO1C	403	22	20801	7.038462	1	0.264613	0.217704
GOTERM_CC_DIRECT	GO:0005749~mitochondrial respiratory chain complex II, succinate dehydrogenase complex (ubiquinone)	2	0.478469	0.075097	SDHC, SDHB	403	4	20801	25.80769	1	0.292413	0.240576
GOTERM_CC_DIRECT	GO:0070820~tertiary granule	2	0.478469	0.075097	STX7, STXBP2	403	4	20801	25.80769	1	0.292413	0.240576
GOTERM_CC_DIRECT	GO:0055038~recycling endosome membrane	4	0.956938	0.079172	EHD1, CMTM6, RAB8A, ARF6	403	52	20801	3.970414	1	0.305578	0.251407
GOTERM_CC_DIRECT	GO:0043197~dendritic spine	5	1.196172	0.085283	CYTH2, GIPC1, ASAP1, SEPTIN11, RAB8A	403	86	20801	3.000894	1	0.3263	0.268456

GOTERM_CC_DIRECT	GO:0005769~early endosome	8	1.913876	0.088479	SNX3, EHD1, TF, RAP1A, SNX27, MME, FLOT1, MAPK3	403	197	20801	2.096056	1	0.33561	0.276116
GOTERM_CC_DIRECT	GO:0030665~clathrin-coated vesicle membrane	3	0.717703	0.089357	ATP6AP2, ATP6V0C, ATP6V0A1	403	26	20801	5.955621	1	0.336042	0.276471
GOTERM_CC_DIRECT	GO:0030054~cell junction	6	1.435407	0.090458	SCIN, RAP1A, HNRNPK, CDH1, ACTN4, SPTAN1	403	123	20801	2.517824	1	0.337301	0.277507
GOTERM_CC_DIRECT	GO:0097227~sperm annulus	2	0.478469	0.092975	SEPTIN2, SEPTIN6	403	5	20801	20.64615	1	0.338091	0.278157
GOTERM_CC_DIRECT	GO:0098826~endoplasmic reticulum tubular network membrane	2	0.478469	0.092975	ATL2, RTN4	403	5	20801	20.64615	1	0.338091	0.278157
GOTERM_CC_DIRECT	GO:0005787~signal peptidase complex	2	0.478469	0.092975	SPCS3, SEC11A	403	5	20801	20.64615	1	0.338091	0.278157
GOTERM_CC_DIRECT	GO:0005768~endosome	8	1.913876	0.093954	CYTH2, ARPC2, CDH1, FLOT1, FLOT2, EPN1, RAB8A, VPS35L	403	200	20801	2.064615	1	0.33885	0.278781
GOTERM_CC_DIRECT	GO:0005615~extracellular space	35	8.373206	0.099513	IL1RN, TINAGL1, MSTN, CNP, SERPINC1, PLG, PRELP, GBA1, CLU, THBS1, KNG1, C3, VTN, LGALS1, DAG1, SPP1, FAM3C, APOE, A2M, LOC616942, BOLA, CTSB, S100A10, CHID1, FGFBP1, BOLA-NC1, F10, IL16, F2, CP, AGT, MANF, TF, TCN2, SERPINA3-7	403	1399	20801	1.291307	1	0.35598	0.292875

Category	Term	Count	%	PValue	Genes	List Total	Pop Hits	Pop Total	Fold Enricl	Bonferron	Benjamini	FDR
GOTERM_MF_DIRECT	GO:0051015~actin filament binding	27	6.45933	1.63E-13	ARPC1B, TWF2, IQGAP1, IQGAP2, CORO1B, ADD1, SCIN, POF1B, CTNNA1, FLNA, MYH14, FLNB, CTNNA3, CTNNA2, SPTAN1, MYH10, ACTR3, ZNF185, NEBL, TPM1, ARPC4, ACTN4, MARCKS, MYO1C, ARPC2, MYH9, MYO1F	384	205	18413	6.315434	8.36E-11	8.36E-11	8.13E-11
GOTERM_MF_DIRECT	GO:0003723~RNA binding	48	11.48325	2.91E-12	DDX5, CNP, SF3B6, EIF4A3, DDX21, SNU13, SAMHD1, SNRPD2, HNRNPDL, U2AF2, MAGOH, DHX16, SNRPB2, SRSF10, DDX17, RBM39, HNRNPA3, SF3A1, SNRPN, SSB, SAFB, MAGOHB, RANGAP1, HNRNPAB, ATXN2L, HNRNPM, LSM8, DDX39A, HNRNPK, PRPF6, HNRNPUL1, DDX39B, PSPC1, HNRNPH1, EWSR1, APEX1, [illegible]	384	716	18413	3.21456	1.49E-09	7.47E-10	7.27E-10
GOTERM_MF_DIRECT	GO:0042802~identical protein binding	50	11.96172	8.83E-08	GLTP, ACADVL, MSTN, HSPB1, ACTB, CDH1, ACADM, SIL1, FNBP1, TPM1, APOA1, CLDN3, DDX39A, PRPF6, DDX39B, HNRNPH1, EWSR1, VWA1, IRF5, S100A4, MCM6, MAPRE1, SNRNP200, SDC4, SERPINC1, PON2, ATL2, HTRA1, DDX21, SAMHD1, NT5C2, CORO1B, TRA2B, CTNNA1, FLNB, RPRD1B, CSK, APOE, DCPS, [illegible]	384	1047	18413	2.289901	4.54E-05	1.51E-05	1.47E-05
GOTERM_MF_DIRECT	GO:0005525~GTP binding	27	6.45933	1.99E-07	RALB, ATL2, GIMAP1, GNAI3, AK4, SAMHD1, SEPTIN11, RAP1B, TUBA1C, GNA14, TUBA3E, NRAS, RAP1A, GNA11, RAB8A, SEPTIN2, SEPTIN6, TUBB4B, SEPTIN9, SEPTIN7, TUBA4A, EHD1, TUBB2B, TUBB2A, GNAL, ADSS2, ARF6	384	390	18413	3.319651	1.02E-04	2.55E-05	2.48E-05
GOTERM_MF_DIRECT	GO:0016787~hydrolase activity	16	3.827751	6.21E-07	HDAC2, DDX21, ATP1A1, NT5C2, TUBA4A, ACTB, ACTG2, ACTA2, TUBA1C, TUBA3E, ACTA1, ACTC1, NAGLU, SNRNP200, DCPS, ATP6V0C	384	151	18413	5.08085	3.19E-04	6.39E-05	6.21E-05
GOTERM_MF_DIRECT	GO:0003924~GTPase activity	23	5.502392	1.43E-06	RALB, SEPTIN2, ATL2, GNAI3, SEPTIN6, TUBB4B, SEPTIN9, TUBA4A, SEPTIN7, SEPTIN11, RAP1B, TUBA1C, GNA14, TUBA3E, NRAS, TUBB2B, TUBB2A, GNAL, GNAQ, GNA11, GNB1, RAB8A, ARF6	384	326	18413	3.383012	7.36E-04	1.23E-04	1.19E-04
GOTERM_MF_DIRECT	GO:0005524~ATP binding	57	13.63636	2.51E-06	MCM7, EIF4A3, ACTB, ACTG2, LOC112446383, STK10, DHX16, KIF1C, DDX17, ACTR3, TAP2, NME3, KIF23, ACTA2, ACTA1, DDX39A, DDX39B, MCM3, MYH9, MCM5, MCM6, KIF20A, SNRNP200, ABCG2, TOR4A, DDX5, PIK3R4, TWF2, ATP1A4, STK39, DDX21, ABCB8, AK4, ATP1A2, ATP1A1, NT5C2, MYH14, CSK, MYH10, [illegible]	384	1419	18413	1.926131	0.00129	1.84E-04	1.79E-04
GOTERM_MF_DIRECT	GO:0005200~structural constituent of cytoskeleton	9	2.15311	5.94E-06	ACTR3, TUBA1C, TUBA3E, TUBB2B, TUBB2A, ARPC2, TUBB4B, TUBA4A, ADD1	384	48	18413	8.990723	0.003048	3.82E-04	3.71E-04
GOTERM_MF_DIRECT	GO:0005515~protein binding	17	4.066986	1.24E-05	ACTR3, TBCD, ADAM10, PLG, ATP1A2, ATP1A1, F2, ITGAL, AP2A2, TUBA4A, ACTB, EHD1, CYTH2, OS9, DNAJC5, GNB1, DAG1	384	216	18413	3.773884	0.006332	7.06E-04	6.87E-04
GOTERM_MF_DIRECT	GO:0000146~microfilament motor activity	7	1.674641	1.59E-05	MYL6, MYO1C, ACTC1, MYH14, MYH9, MYH10, MYO1F	384	27	18413	12.43162	0.008151	8.18E-04	7.96E-04
GOTERM_MF_DIRECT	GO:0005080~protein kinase C binding	7	1.674641	7.59E-05	MARCKS, PARD6B, PARD6A, PLEK, TWF2, HSPB1, FLNA	384	35	18413	9.590104	0.038248	0.003256	0.003168
GOTERM_MF_DIRECT	GO:0016887~ATPase activity	19	4.545455	7.60E-05	DDX17, HSPA9, TRAP1, DDX5, MCM7, EIF4A3, KIF23, ATP1A2, ATP1A1, HSPE1, DDX39A, DDX39B, MCM3, DHX16, KIF1C, MCM5, KIF20A, MCM6, ABCG2	384	305	18413	2.987082	0.038325	0.003256	0.003168
GOTERM_MF_DIRECT	GO:0003724~RNA helicase activity	8	1.913876	3.24E-04	DDX17, DDX5, DDX39A, DDX39B, EIF4A3, DHX16, DDX21, SNRNP200	384	63	18413	6.088955	0.153418	0.012809	0.01246
GOTERM_MF_DIRECT	GO:0004866~endopeptidase inhibitor activity	5	1.196172	4.48E-04	C3, HRG, A2M, KNG1, UCHL5	384	18	18413	13.31959	0.205522	0.01643	0.015982
GOTERM_MF_DIRECT	GO:0031683~G-protein beta/gamma-subunit complex binding	5	1.196172	5.58E-04	GNA14, GNAL, GNAQ, GNA11, GNAI3	384	19	18413	12.61856	0.24925	0.019107	0.018586
GOTERM_MF_DIRECT	GO:0005509~calcium ion binding	27	6.45933	8.43E-04	SRI, IQGAP1, THBS1, MYL12B, SCIN, CDH1, DAG1, SPTAN1, EPS15, SLC25A24, S100A10, GCA, F10, EPS15L1, ACTN4, F2, EHD1, MYL6, MYL1, GPD2, MGP, MYL3, NUCB1, S100A4, DSC1, MYL9, MATN2	384	635	18413	2.038841	0.351797	0.027085	0.026348
GOTERM_MF_DIRECT	GO:0003779~actin binding	13	3.110048	0.00305	ACTR3, ARPC1B, TPM1, RDX, TWF2, ARPC4, ACTN4, SCIN, MYO1C, CALD1, GIPC1, DAG1, SPTAN1	384	228	18413	2.734021	0.791929	0.092205	0.089693
GOTERM_MF_DIRECT	GO:0008289~lipid binding	9	2.15311	0.003624	GLTP, CYTH2, APOA1, BAX, APOA4, APOE, AP2A2, EPN1, AP2M1	384	120	18413	3.596289	0.845297	0.103492	0.100674
GOTERM_MF_DIRECT	GO:0060228~phosphatidylcholine-sterol O-acyltransferase activator activity	3	0.717703	0.00414	APOA1, APOA4, APOE	384	5	18413	28.77031	0.881426	0.11199	0.108939
GOTERM_MF_DIRECT	GO:0043236~laminin binding	4	0.956938	0.004888	LGALS1, TINAGL1, DAG1, THBS1	384	17	18413	11.28248	0.919444	0.122325	0.118994
GOTERM_MF_DIRECT	GO:0019904~protein domain specific binding	10	2.392344	0.004998	HNRNPM, OCLN, HNRNPK, TRA2B, LAMP2, CHMP2B, MYH9, PLG, IQGAP1, SRSF7	384	154	18413	3.11367	0.923867	0.122325	0.118994
GOTERM_MF_DIRECT	GO:0001664~G-protein coupled receptor binding	6	1.435407	0.006076	GNA14, GNAL, GNAQ, GNA11, GNAI3, FLNA	384	56	18413	5.137556	0.956395	0.136865	0.133137
GOTERM_MF_DIRECT	GO:0005391~sodium:potassium-exchanging ATPase activity	3	0.717703	0.006124	ATP1A4, ATP1A2, ATP1A1	384	6	18413	23.97526	0.957471	0.136865	0.133137
GOTERM_MF_DIRECT	GO:0019001~guanyl nucleotide binding	3	0.717703	0.008457	GNA14, GNAQ, GNA11	384	7	18413	20.55022	0.987287	0.181111	0.176178
GOTERM_MF_DIRECT	GO:0019901~protein kinase binding	16	3.827751	0.010514	SLC12A2, TRAP1, NEK6, ADAM10, IQGAP1, ELAVL1, AP2A2, TUBA4A, ACTB, PPP1CA, ACTA2, PRC1, PDCD10, IRF5, KIF20A, MAPRE1	384	368	18413	2.084805	0.995629	0.211714	0.205948
GOTERM_MF_DIRECT	GO:0050839~cell adhesion molecule binding	5	1.196172	0.011066	CDH1, RDX, ITGAL, TJP3, TJP2	384	42	18413	5.708395	0.996719	0.211714	0.205948
GOTERM_MF_DIRECT	GO:0003995~acyl-CoA dehydrogenase activity	3	0.717703	0.011121	ACADVL, ACADM, ACADS	384	8	18413	17.98145	0.996812	0.211714	0.205948
GOTERM_MF_DIRECT	GO:0019003~GDP binding	6	1.435407	0.012774	RAP1B, NRAS, RALB, GNAI3, RAB8A, ARF6	384	67	18413	4.294076	0.998651	0.232401	0.226071
GOTERM_MF_DIRECT	GO:0003725~double-stranded RNA binding	6	1.435407	0.01356	OAS1X, ILF3, DDX21, TUBB4B, ILF2, ELAVL1	384	68	18413	4.230928	0.999104	0.232401	0.226071
GOTERM_MF_DIRECT	GO:0031267~small GTPase binding	11	2.631579	0.013564	EHD1, MYO1C, PARD6A, RAP1A, FLNA, IQGAP1, IQGAP2, RANGAP1, IPO4, UNC13D, RAB8A	384	212	18413	2.487999	0.999106	0.232401	0.226071
GOTERM_MF_DIRECT	GO:0003729~mRNA binding	11	2.631579	0.015717	HNRNPL, HNRNPM, SSB, HNRNPK, TRA2B, EIF4A3, SRSF4, NUP98, SUPT5H, RBMX, LUC7L2	384	217	18413	2.430672	0.999709	0.260598	0.2535
GOTERM_MF_DIRECT	GO:0008013~beta-catenin binding	6	1.435407	0.019978	NHERF1, TRPC4, CDH1, CTNNA1, CTNNA3, CTNNA2	384	75	18413	3.836042	0.999969	0.32003	0.311313
GOTERM_MF_DIRECT	GO:0005096~GTPase activator activity	10	2.392344	0.020547	SEC23A, GNAQ, TBCD, PGAM5, ASAP1, NCKAP1L, IQGAP1, IQGAP2, RANGAP1, AGFG1	384	194	18413	2.471676	0.999977	0.32003	0.311313
GOTERM_MF_DIRECT	GO:0042803~protein homodimerization activity	21	5.023923	0.022425	GCA, MSTN, MME, SHMT2, PLEK, TPM1, HSPB1, ADAM10, APOA1, APOA4, ACTN4, ELAVL1, STK10, STK25, PDCD10, FLNA, MYH9, BAX, APOE, ABCG2, S100A10	384	588	18413	1.712519	0.999991	0.33901	0.329776
GOTERM_MF_DIRECT	GO:0036002~pre-mRNA binding	3	0.717703	0.024815	DDX5, TRA2B, SRSF2	384	12	18413	11.98763	0.999998	0.364429	0.354503
GOTERM_MF_DIRECT	GO:0051087~chaperone binding	6	1.435407	0.02561	SLC12A2, PLG, ATP1A2, ATP1A1, HSPE1, CP	384	80	18413	3.596289	0.999998	0.365654	0.355694
GOTERM_MF_DIRECT	GO:0060090~binding, bridging	6	1.435407	0.026846	SEPTIN2, SEPTIN6, LAMTOR2, SEPTIN9, SEPTIN7, SEPTIN11	384	81	18413	3.55189	0.999999	0.372944	0.362786
GOTERM_MF_DIRECT	GO:0070679~inositol 1,4,5 trisphosphate binding	3	0.717703	0.02893	CYTH2, TRPC5, TRPC4	384	13	18413	11.0655	1	0.381276	0.370891
GOTERM_MF_DIRECT	GO:0004407~histone deacetylase activity	3	0.717703	0.02893	HDAC2, HDAC1, MTA2	384	13	18413	11.0655	1	0.381276	0.370891
GOTERM_MF_DIRECT	GO:0045296~cadherin binding	5	1.196172	0.030688	TRPC4, CDH1, CTNNA1, CTNNA3, CTNNA2	384	57	18413	4.206186	1	0.394336	0.383596
GOTERM_MF_DIRECT	GO:0003920~GMP reductase activity	2	0.478469	0.041169	GMPR2, GMPR	384	2	18413	47.95052	1	0.503836	0.490113
GOTERM_MF_DIRECT	GO:0061656~SUMO conjugating enzyme activity	2	0.478469	0.041169	UBE2I, LOC112446383	384	2	18413	47.95052	1	0.503836	0.490113
GOTERM_MF_DIRECT	GO:0046872~metal ion binding	45	10.76555	0.044471	HDAC2, HDAC1, PON2, GMPR2, ATP1A4, GMPR, ASAP1, ATP1A1, ITGAL, NT5C2, COX5A, LPP, TUBA1C, GNA14, TUBA3E, MAN2A2, PPAT, GNA11, CSK, HMOX2, AGFG1, CYB5A, ZNF185, RPA1, ADAM10, SDHC, TUBB4B, HSPE1, SDHB, TUBA4A, PPP1CA, TUBB2B, UBLCP1, TCN2, ASPH, STK25, PLSCR2, EWSR1, APEX1, OGDH[illegible]	384	1630	18413	1.323787	1	0.531579	0.5171
GOTERM_MF_DIRECT	GO:0008307~structural constituent of muscle	3	0.717703	0.047773	MYL6, MYL1, DAG1	384	17	18413	8.461857	1	0.54567	0.530808
GOTERM_MF_DIRECT	GO:0051010~microtubule plus-end binding	3	0.717703	0.047773	CLIP1, MAPRE1, CKAP5	384	17	18413	8.461857	1	0.54567	0.530808
GOTERM_MF_DIRECT	GO:0043495~protein anchor	3	0.717703	0.053022	SUN2, NHERF1, MYH9	384	18	18413	7.991753	1	0.583532	0.567638
GOTERM_MF_DIRECT	GO:0051117~ATPase binding	5	1.196172	0.055502	RALB, RDX, SNU13, ATP1B3, ATP6V0A1	384	69	18413	3.474675	1	0.583532	0.567638
GOTERM_MF_DIRECT	GO:0035615~clathrin adaptor activity	3	0.717703	0.058465	AP2S1, AP2A2, AP2M1	384	19	18413	7.571135	1	0.583532	0.567638
GOTERM_MF_DIRECT	GO:0008201~heparin binding	7	1.674641	0.05955	MSTN, SERPINC1, PRELP, APOE, HRG, F2, THBS1	384	133	18413	2.523712	1	0.583532	0.567638
GOTERM_MF_DIRECT	GO:0015485~cholesterol binding	4	0.956938	0.059816	CD81, APOA1, VDAC2, APOA4	384	43	18413	4.460514	1	0.583532	0.567638
GOTERM_MF_DIRECT	GO:0004591~oxoglutarate dehydrogenase (succinyl-transferring) activity	2	0.478469	0.061116	OGDH, OGDHL	384	3	18413	31.96701	1	0.583532	0.567638
GOTERM_MF_DIRECT	GO:0043008~ATP-dependent protein binding	2	0.478469	0.061116	DDX39B, SAE1	384	3	18413	31.96701	1	0.583532	0.567638
GOTERM_MF_DIRECT	GO:0070053~thrombospondin receptor activity	2	0.478469	0.061116	SDC4, F2	384	3	18413	31.96701	1	0.583532	0.567638
GOTERM_MF_DIRECT	GO:0005516~calmodulin binding	7	1.674641	0.061305	MARCKS, MYO1C, CALD1, IQGAP1, IQGAP2, MYH10, SPTAN1	384	134	18413	2.504878	1	0.583532	0.567638
GOTERM_MF_DIRECT	GO:0017022~myosin binding	3	0.717703	0.064092	ACTC1, CALD1, GIPC1	384	20	18413	7.192578	1	0.598969	0.582654
GOTERM_MF_DIRECT	GO:0051787~misfolded protein binding	3	0.717703	0.069892	HSPA9, DNAJB9, CLU	384	21	18413	6.850074	1	0.632089	0.614872
GOTERM_MF_DIRECT	GO:0003678~DNA helicase activity	4	0.956938	0.070359	MCM7, MCM3, MCM5, MCM6	384	46	18413	4.169611	1	0.632089	0.614872
GOTERM_MF_DIRECT	GO:0005102~receptor binding	9	2.15311	0.071325	MYO1C, NHERF1, GIPC1, LAMA4, PLG, HRG, F2, KNG1, FRK	384	209	18413	2.064855	1	0.632089	0.614872
GOTERM_MF_DIRECT	GO:0005254~chloride channel activity	4	0.956938	0.077819	CLIC5, CLIC4, CLIC2, CLIC1	384	48	18413	3.995877	1	0.6686	0.650389
GOTERM_MF_DIRECT	GO:1990239~steroid hormone binding	2	0.478469	0.080648	ATP1A2, ATP1A1	384	4	18413	23.97526	1	0.6686	0.650389
GOTERM_MF_DIRECT	GO:0070051~fibrinogen binding	2	0.478469	0.080648	F2, THBS1	384	4	18413	23.97526	1	0.6686	0.650389
GOTERM_MF_DIRECT	GO:0043047~single-stranded telomeric DNA binding	2	0.478469	0.080648	HNRNPA2B1, RPA1	384	4	18413	23.97526	1	0.6686	0.650389

Category	Term	Count	%	PValue	Genes	List Total	Pop Hits	Pop Total	Fold Enricl	Bonferron	Benjamini	FDR
KEGG_PATHWAY	bta04530:Tight junction	26	6.220096	1.68E-10	PATJ, ARPC1B, ACTB, MYL12B, TUBA1C, TUBA3E, PARD6B, PARD6A, RAP1A, MYH14, MYH10, RAB8A, ACTR3, NHERF1, RDX, ARPC4, ACTN4, TUBA4A, OCLN, MYL6, CLDN3, ARPC2, MYH9, MYL9, TJP3, TJP2	287	178	9288	4.727088	4.80E-08	4.80E-08	4.09E-08
KEGG_PATHWAY	bta04814:Motor proteins	26	6.220096	2.30E-09	ACTB, ACTG2, MYL12B, TUBA1C, TUBA3E, MYH14, KIF1C, MYH10, TPM1, KIF23, TUBB4B, TUBA4A, ACTA2, ACTA1, TUBB2B, MYL6, MYO1C, TUBB2A, ACTC1, MYL1, MYL3, MYH9, KIF20A, DYNLRB1, MYL9, MYO1F	287	201	9288	4.186177	6.57E-07	3.29E-07	2.80E-07
KEGG_PATHWAY	bta03040:Spliceosome	28	6.698565	5.19E-09	DDX5, SF3B2, SF3B6, EIF4A3, SNU13, PRPF8, SNRPD2, U2AF2, TRA2B, MAGOH, DHX16, SNRPB2, SRSF10, HNRNPA3, SF3A1, MAGOHB, HNRNPM, LSM8, LSM7, HNRNPK, PRPF6, DDX39B, SRSF2, SRSF4, SNRNP200, RBMX, SRSF7, SNRPB	287	241	9288	3.759943	1.48E-06	4.95E-07	4.22E-07
KEGG_PATHWAY	bta04810:Regulation of actin cytoskeleton	23	5.502392	1.78E-06	ACTR3, ARPC1B, RDX, ARPC4, IQGAP1, ACTN4, IQGAP2, F2, ITGAL, BAIAP2, KNG1, ACTB, PPP1CA, MYL12B, NRAS, SCIN, ARPC2, MYH14, MYH9, NCKAP1L, MYL9, MYH10, MAPK3	287	227	9288	3.279007	5.10E-04	1.28E-04	1.09E-04
KEGG_PATHWAY	bta00020:Citrate cycle (TCA cycle)	9	2.15311	2.38E-06	SUCLA2, IDH2, OGDH, DLST, SDHC, OGDHL, PDHB, SDHB, IDH3A	287	30	9288	9.708711	6.80E-04	1.36E-04	1.16E-04
KEGG_PATHWAY	bta04144:Endocytosis	23	5.502392	6.28E-06	ACTR3, BOLA-NC1, ARPC1B, ARPC4, ASAP1, EPS15L1, AP2A2, EPN1, SNX3, EHD1, CYTH2, PARD6B, PARD6A, ARPC2, CHMP2B, AP2S1, CHMP3, LOC616942, BOLA, EPS15, AP2M1, RAB8A, ARF6	287	245	9288	3.0381	0.001793	2.24E-04	1.91E-04
KEGG_PATHWAY	bta04145:Phagosome	18	4.30622	1.13E-05	BOLA-NC1, STX7, TAP2, TUBB4B, THBS1, TUBA4A, ACTB, C3, TUBA1C, TUBA3E, TUBB2B, FCGR2A, TUBB2A, LAMP2, LOC616942, BOLA, ATP6V0C, ATP6V0A1	287	164	9288	3.551967	0.003232	3.60E-04	3.07E-04
KEGG_PATHWAY	bta04260:Cardiac muscle contraction	12	2.870813	1.26E-04	ACTC1, ASPH, MYL3, TPM1, ATP1A4, UQCRC1, ATP1B3, ATP1A2, ATP1A1, UQCR10, UQCRC2, COX5A	287	93	9288	4.17579	0.035343	0.002768	0.002361
KEGG_PATHWAY	bta04961:Endocrine and other factor-regulated calcium reabsorption	9	2.15311	1.48E-04	PLCB3, GNAQ, ATP1A4, AP2S1, ATP1B3, ATP1A2, ATP1A1, AP2A2, AP2M1	287	51	9288	5.711006	0.041313	0.003013	0.002571
KEGG_PATHWAY	bta04670:Leukocyte transendothelial migration	13	3.110048	2.28E-04	GNAI3, ACTN4, ITGAL, ACTB, MYL12B, RAP1B, OCLN, RAP1A, CLDN3, CTNNA1, CTNNA3, CTNNA2, MYL9	287	116	9288	3.626817	0.063093	0.004344	0.003706
KEGG_PATHWAY	bta05016:Huntington disease	23	5.502392	2.95E-04	NDUFA8, HDAC2, HDAC1, PIK3R4, SDHC, UQCR10, TUBB4B, COX5A, AP2A2, SDHB, TUBA4A, TUBA1C, TUBA3E, PLCB3, TUBB2B, TUBB2A, GNAQ, AP2S1, UQCRC1, VDAC2, BAX, UQCRC2, AP2M1	287	316	9288	2.355489	0.080857	0.005269	0.004495
KEGG_PATHWAY	bta04270:Vascular smooth muscle contraction	14	3.349282	3.69E-04	AGT, ACTG2, PPP1CA, ACTA2, PLCB3, MYL6, CALD1, GNAQ, GNA11, MYH14, MYH9, MYL9, MYH10, MAPK3	287	140	9288	3.236237	0.100253	0.006213	0.005301
KEGG_PATHWAY	bta04610:Complement and coagulation cascades	11	2.631579	5.54E-04	C3, VTN, F10, SERPINC1, C5AR1, PLG, A2M, F2, CLU, KNG1, CFB	287	93	9288	3.827807	0.146492	0.008798	0.007506
KEGG_PATHWAY	bta01200:Carbon metabolism	12	2.870813	5.99E-04	ECHS1, SUCLA2, SHMT2, IDH2, OGDH, DLST, SDHC, OGDHL, PDHB, ACADS, SDHB, IDH3A	287	111	9288	3.498635	0.15737	0.009009	0.007686
KEGG_PATHWAY	bta04611:Platelet activation	12	2.870813	0.001317	RAP1B, PLCB3, FCGR2A, RAP1A, GNAQ, GNAI3, F2, ACTB, MAPK3, PPP1CA, MYL12B, FERMT3	287	122	9288	3.183184	0.314013	0.018832	0.016067
KEGG_PATHWAY	bta01210:2-Oxocarboxylic acid metabolism	6	1.435407	0.00272	IDH2, OGDH, DLST, OGDHL, PDHB, IDH3A	287	32	9288	6.067944	0.5411	0.034916	0.029789
KEGG_PATHWAY	bta04261:Adrenergic signaling in cardiomyocytes	13	3.110048	0.002815	TPM1, ATP1A4, GNAI3, ATP1B3, ATP1A2, ATP1A1, AGT, PPP1CA, PLCB3, ACTC1, GNAQ, MYL3, MAPK3	287	154	9288	2.731888	0.553444	0.034916	0.029789
KEGG_PATHWAY	bta05010:Alzheimer disease	24	5.741627	0.00292	NDUFA8, MME, PIK3R4, ADAM10, SDHC, UQCR10, TUBB4B, COX5A, SDHB, RTN4, TUBA4A, TUBA1C, TUBA3E, PLCB3, NRAS, TUBB2B, TUBB2A, GNAQ, UQCRC1, VDAC2, APOE, NAE1, UQCRC2, MAPK3	287	400	9288	1.941742	0.566659	0.034916	0.029789
KEGG_PATHWAY	bta04666:Fc gamma R-mediated phagocytosis	10	2.392344	0.00293	ACTR3, MARCKS, SCIN, FCGR2A, ARPC2, ARPC1B, ARPC4, ASAP1, MAPK3, ARF6	287	97	9288	3.336327	0.567952	0.034916	0.029789
KEGG_PATHWAY	bta04929:GnRH secretion	8	1.913876	0.003376	PLCB3, NRAS, TRPC5, TRPC4, GNAQ, GNA11, SPP1, MAPK3	287	64	9288	4.045296	0.619827	0.03862	0.032948
KEGG_PATHWAY	bta04510:Focal adhesion	15	3.588517	0.003997	LAMA4, LAMC1, ACTN4, THBS1, ACTB, PPP1CA, MYL12B, RAP1B, VTN, RAP1A, SPP1, FLNA, FLNB, MYL9, MAPK3	287	203	9288	2.391308	0.681943	0.043542	0.037148
KEGG_PATHWAY	bta04972:Pancreatic secretion	10	2.392344	0.004111	RAP1B, SLC12A2, PLCB3, RAP1A, GNAQ, ATP1A4, ATP1B3, ATP1A2, ATP1A1, RAB8A	287	102	9288	3.172781	0.692122	0.043542	0.037148
KEGG_PATHWAY	bta05012:Parkinson disease	18	4.30622	0.005854	TRAP1, NDUFA8, GNAI3, SDHC, UQCR10, TUBB4B, COX5A, SDHB, TUBA4A, TUBA1C, TUBA3E, TUBB2B, TUBB2A, GNAL, UQCRC1, VDAC2, BAX, UQCRC2	287	280	9288	2.080438	0.813477	0.059796	0.051015
KEGG_PATHWAY	bta04022:cGMP-PKG signaling pathway	13	3.110048	0.006524	ATP1A4, GNAI3, ATP1B3, ATP1A2, ATP1A1, KNG1, PPP1CA, PLCB3, GNAQ, GNA11, VDAC2, MYL9, MAPK3	287	171	9288	2.460297	0.846163	0.064336	0.054888
KEGG_PATHWAY	bta05022:Pathways of neurodegeneration - multiple diseases	26	6.220096	0.008212	TOMM40, PIK3R4, UQCR10, COX5A, TUBA1C, TUBA3E, NRAS, RAB8A, MAPK3, NDUFA8, TRAP1, SDHC, TUBB4B, SDHB, TUBA4A, ATXN2L, PLCB3, TUBB2B, TUBB2A, GNAQ, CHMP2B, UQCRC1, VDAC2, BAX, UQCRC2, TARDBP	287	487	9288	1.727765	0.905408	0.078283	0.066787
KEGG_PATHWAY	bta04971:Gastric acid secretion	8	1.913876	0.0087	PLCB3, GNAQ, ATP1A4, GNAI3, ATP1B3, ATP1A2, ATP1A1, ACTB	287	76	9288	3.406565	0.917832	0.079885	0.068154
KEGG_PATHWAY	bta03013:Nucleocytoplasmic transport	10	2.392344	0.008938	UBE2I, DDX39B, TPR, EIF4A3, MAGOH, NUP98, MAGOHB, RANGAP1, IPO4, LOC112446383	287	115	9288	2.814119	0.923298	0.079885	0.068154
KEGG_PATHWAY	bta05213:Endometrial cancer	7	1.674641	0.009276	NRAS, CDH1, CTNNA1, BAX, CTNNA3, CTNNA2, MAPK3	287	59	9288	3.839603	0.930425	0.080393	0.068587
KEGG_PATHWAY	bta05143:African trypanosomiasis	6	1.435407	0.00988	PLCB3, GNAQ, LAMA4, APOA1, KNG1, IDO1	287	43	9288	4.515679	0.941556	0.083107	0.070903
KEGG_PATHWAY	bta05205:Proteoglycans in cancer	14	3.349282	0.010343	DDX5, SDC4, RDX, IQGAP1, THBS1, ACTB, PPP1CA, VTN, NRAS, PDCD4, FLNA, FLNB, CD44, MAPK3	287	204	9288	2.220947	0.948883	0.084521	0.072109
KEGG_PATHWAY	bta04371:Apelin signaling pathway	11	2.631579	0.011673	ACTA2, PLCB3, NRAS, CDH1, GNAQ, GNB1, MYL3, PIK3R4, GNAI3, SPP1, MAPK3	287	141	9288	2.524724	0.965198	0.092735	0.079116
KEGG_PATHWAY	bta04919:Thyroid hormone signaling pathway	10	2.392344	0.012252	HDAC2, PLCB3, NRAS, HDAC1, ATP1A4, ATP1B3, ATP1A2, ATP1A1, ACTB, MAPK3	287	121	9288	2.674576	0.970571	0.093205	0.079517
KEGG_PATHWAY	bta05020:Prion disease	17	4.066986	0.012384	NDUFA8, SDHC, UQCR10, LAMC1, TUBB4B, COX5A, SDHB, TUBA4A, TUBA1C, TUBA3E, TUBB2B, TUBB2A, UQCRC1, VDAC2, BAX, UQCRC2, MAPK3	287	279	9288	1.971901	0.971671	0.093205	0.079517
KEGG_PATHWAY	bta04015:Rap1 signaling pathway	14	3.349282	0.015506	RALB, GNAI3, ITGAL, THBS1, ACTB, RAP1B, PARD6B, PLCB3, NRAS, PARD6A, RAP1A, CDH1, GNAQ, MAPK3	287	215	9288	2.107317	0.988547	0.113711	0.097013
KEGG_PATHWAY	bta04720:Long-term potentiation	7	1.674641	0.019152	RAP1B, PLCB3, NRAS, RAP1A, GNAQ, MAPK3, PPP1CA	287	69	9288	3.283139	0.996037	0.136774	0.116688
KEGG_PATHWAY	bta04512:ECM-receptor interaction	8	1.913876	0.019607	VTN, SDC4, LAMA4, DAG1, SPP1, LAMC1, THBS1, CD44	287	89	9288	2.908977	0.996529	0.136774	0.116688
KEGG_PATHWAY	bta05165:Human papillomavirus infection	19	4.545455	0.02136	PATJ, HDAC2, BOLA-NC1, NHERF1, HDAC1, LAMA4, LAMC1, THBS1, VTN, PARD6B, NRAS, PARD6A, SPP1, BAX, LOC616942, BOLA, ATP6V0C, ATP6V0A1, MAPK3	287	347	9288	1.772003	0.99792	0.145455	0.124094
KEGG_PATHWAY	bta00785:Lipoic acid metabolism	4	0.956938	0.022345	OGDH, DLST, OGDHL, PDHB	287	20	9288	6.472474	0.99844	0.148618	0.126793
KEGG_PATHWAY	bta03030:DNA replication	5	1.196172	0.023911	MCM7, MCM3, RPA1, MCM5, MCM6	287	36	9288	4.494774	0.999014	0.155421	0.132597
KEGG_PATHWAY	bta05146:Amoebiasis	9	2.15311	0.025393	GNA14, PLCB3, GNAL, GNAQ, GNA11, LAMA4, HSPB1, LAMC1, ACTN4	287	115	9288	2.532707	0.999361	0.161386	0.137686
KEGG_PATHWAY	bta04960:Aldosterone-regulated sodium reabsorption	5	1.196172	0.02618	ATP1A4, ATP1B3, ATP1A2, ATP1A1, MAPK3	287	37	9288	4.373293	0.999493	0.162769	0.138866
KEGG_PATHWAY	bta04925:Aldosterone synthesis and secretion	8	1.913876	0.028344	PLCB3, GNAQ, GNA11, ATP1A4, ATP1B3, ATP1A2, ATP1A1, AGT	287	96	9288	2.696864	0.999732	0.17194	0.14669
KEGG_PATHWAY	bta04964:Proximal tubule bicarbonate reclamation	4	0.956938	0.028857	ATP1A4, ATP1B3, ATP1A2, ATP1A1	287	22	9288	5.884067	0.999769	0.17194	0.14669
KEGG_PATHWAY	bta05142:Chagas disease	9	2.15311	0.030349	C3, GNA14, PLCB3, GNAL, GNAQ, GNA11, GNAI3, KNG1, MAPK3	287	119	9288	2.447574	0.999851	0.174855	0.149177
KEGG_PATHWAY	bta05170:Human immunodeficiency virus 1 infection	14	3.349282	0.030569	BOLA-NC1, GNAI3, TAP2, SAMHD1, NRAS, GNAQ, GNA11, GNB1, CDK1, BAX, LOC616942, BOLA, B2M, MAPK3	287	236	9288	1.919802	0.999861	0.174855	0.149177
KEGG_PATHWAY	bta05216:Thyroid cancer	5	1.196172	0.033739	NRAS, CDH1, TPR, BAX, MAPK3	287	40	9288	4.045296	0.999945	0.189202	0.161417
KEGG_PATHWAY	bta04071:Sphingolipid signaling pathway	9	2.15311	0.034477	PLCB3, SGPL1, NRAS, SPTLC2, GNAQ, GNAI3, BAX, KNG1, MAPK3	287	122	9288	2.387388	0.999956	0.189625	0.161778
KEGG_PATHWAY	bta04978:Mineral absorption	6	1.435407	0.036861	TF, ATP1A4, ATP1B3, ATP1A2, ATP1A1, HMOX2	287	60	9288	3.236237	0.999978	0.198909	0.169699
KEGG_PATHWAY	bta04730:Long-term depression	6	1.435407	0.039182	PLCB3, NRAS, GNAQ, GNA11, GNAI3, MAPK3	287	61	9288	3.183184	0.999989	0.207519	0.177044

KEGG_PATHWAY	bta04973:Carbohydrate digestion and absorption	5	1.196172	0.045599	PLCB3, ATP1A4, ATP1B3, ATP1A2, ATP1A1	287	44	9288	3.677542	0.999998	0.234556	0.200111
KEGG_PATHWAY	bta01232:Nucleotide metabolism	7	1.674641	0.046747	RRM1, GMPR2, GMPR, NME3, AK4, ADSS2, NT5C2	287	85	9288	2.665136	0.999999	0.234556	0.200111
KEGG_PATHWAY	bta04911:Insulin secretion	7	1.674641	0.046747	PLCB3, GNAQ, GNA11, ATP1A4, ATP1B3, ATP1A2, ATP1A1	287	85	9288	2.665136	0.999999	0.234556	0.200111
KEGG_PATHWAY	bta05135:Yersinia infection	10	2.392344	0.048276	ACTR3, FCGR2A, ARPC2, GNAQ, ARPC1B, ARPC4, BAIAP2, ACTB, MAPK3, ARF6	287	154	9288	2.101453	0.999999	0.238051	0.203092
KEGG_PATHWAY	bta05203:Viral carcinogenesis	13	3.110048	0.052916	HDAC2, BOLA-NC1, HDAC1, ACTN4, C3, NRAS, SCIN, HNRNPK, CDK1, BAX, LOC616942, BOLA, MAPK3	287	231	9288	1.821259	1	0.256509	0.21884
KEGG_PATHWAY	bta05415:Diabetic cardiomyopathy	12	2.870813	0.06477	NDUFA8, PLCB3, UQCRC1, VDAC2, SDHC, PDHB, UQCR10, UQCRC2, COX5A, SDHB, AGT, PPP1CA	287	213	9288	1.823232	1	0.308735	0.263396
KEGG_PATHWAY	bta05206:MicroRNAs in cancer	15	3.588517	0.06829	HDAC2, UBE2I, HDAC1, TPM1, RDX, KIF23, THBS1, LOC112446383, MARCKS, NRAS, HNRNPK, STMN1, PDCD4, CD44, MAPK3	287	293	9288	1.656777	1	0.320178	0.273159
KEGG_PATHWAY	bta00190:Oxidative phosphorylation	9	2.15311	0.069492	NDUFA8, UQCRC1, SDHC, UQCR10, UQCRC2, ATP6V0C, COX5A, SDHB, ATP6V0A1	287	141	9288	2.065683	1	0.32056	0.273485
KEGG_PATHWAY	bta05200:Pathways in cancer	24	5.741627	0.072451	EGLN1, HDAC2, RALB, HDAC1, LAMA4, GSTP1, GNAI3, MGST1, LAMC1, F2, AGT, KNG1, PLCB3, NRAS, CDH1, GNAQ, GNA11, GNB1, TPR, CTNNA1, BAX, CTNNA3, CTNNA2, MAPK3	287	542	9288	1.43302	1	0.328906	0.280605
KEGG_PATHWAY	bta04210:Apoptosis	9	2.15311	0.074113	TUBA1C, TUBA3E, NRAS, BAX, SPTAN1, TUBA4A, ACTB, CTSB, MAPK3	287	143	9288	2.036792	1	0.331191	0.282554
KEGG_PATHWAY	bta05163:Human cytomegalovirus infection	13	3.110048	0.07762	BOLA-NC1, GNAI3, TAP2, PLCB3, NRAS, GNAQ, GNA11, GNB1, BAX, LOC616942, BOLA, B2M, MAPK3	287	246	9288	1.710207	1	0.34153	0.291375
KEGG_PATHWAY	bta04918:Thyroid hormone synthesis	6	1.435407	0.080919	PLCB3, GNAQ, ATP1A4, ATP1B3, ATP1A2, ATP1A1	287	75	9288	2.58899	1	0.350651	0.299157
KEGG_PATHWAY	bta04970:Salivary secretion	7	1.674641	0.091082	SLC12A2, PLCB3, GNAQ, ATP1A4, ATP1B3, ATP1A2, ATP1A1	287	101	9288	2.242936	1	0.383078	0.326822
KEGG_PATHWAY	bta03015:mRNA surveillance pathway	7	1.674641	0.091082	DDX39B, EIF4A3, MAGOH, PABPC1L, TARDBP, MAGOHB, PPP1CA	287	101	9288	2.242936	1	0.383078	0.326822
KEGG_PATHWAY	bta05416:Viral myocarditis	6	1.435407	0.095973	BOLA-NC1, DAG1, ITGAL, LOC616942, BOLA, ACTB	287	79	9288	2.457901	1	0.3978	0.339382
KEGG_PATHWAY	bta04921:Oxytocin signaling pathway	9	2.15311	0.099941	PLCB3, NRAS, MYL6, GNAQ, GNAI3, MYL9, ACTB, MAPK3, PPP1CA	287	153	9288	1.903669	1	0.40833	0.348365

Proteomic analysis of buffalo milk somatic cells reveals metabolomic and immunological transitions during early lactation

Priyanka M. Kittur[1], Lija Satheesan[1], Narasimha Tanuj Gunturu[2], Yallappa M. Somagond[1,3], A. P. Madhusoodan[1], Ravi Kumar Gandham[2], Rani Alex[4], and Ajay Kumar Dang[1]*

Supplementary Table S1: Details of various primers used for the validation in the study.

Target genes	Sequence (5′→3′)	Size (bp)	Annealing Temp (°C)	Acc. No.
IDH1	F: CAACCGGGATTGCCAAAGTG	528	61	>XM_006044596.3
	R: GGGTTTTACCCATCCGCTCA			
ACACB	F: GGGGACAGAAGCCAGGAGAT	424	61	>XM_006044834.4
	R: TGGGCATCCAGGCTGAATTT			
CD36	F: TTGCTCAGTGTTGGTGTGGT	225	60	>XM_006049691.4
	R: AGATCCATGGCTACTGGAAAATCA			
FABP3	F: TGAAGTCACTCGGTGTCGGT	227	60	>NM_001290882.1
	R: TTGCACGTGGACAAGTTTGC			
APOA1	F: TCTGGGACAACCTGGAAAAGG	564	60	>XM_025266849.2
	R: ATTCTAAGAAACCGAAGTGATGGC			
APOE	F: CTGGGATCCGTGAGTCCCTAC	111	60	>XM_006067539.4
	R: GGCAACCCACAGAACCTTCA			
VTN	F: TTCCATGACTACAGCGAGGC	286	60	>XM_006042202.4
	R: GGAAGGCAAAGAGGGAACCA			
SERPINC1	F: TTCAAGGGCCTGTGGAAGTC	126	60	>XM_006063663.4
	R: TCTCCGATAGCGGAACTTGC			
PLG	F: AGAAACCCGAGGCACTTCTG	263	60	>XM_025294313.3
	R: ACACACCAGGCTTATTGGGG			
TTR	F: GGTGGTGTTCACAGCCAATG	141	60	>XM_006053996.4
	R: CTCATCCTTCAAGTCCGCCG			
TF	F: GGTTCAGTGACGGGGCAATA	217	60	>XM_006051414.4
	R: ACAGCCACAGCAAGATACCC			
RPS23	F: CCCAATGATGGTTGCTTGAA	101	60	>XM_006059350.4
	R: CGGACTCCAGGAATGTCACC			
RPS9	F: CCTCGACCAAGAGCTGAAG	64	60	>XM_006053433.4
	R: CCTCCAGACCTCACGTTTGTTC			

F, Forward; R, Reverse; IDH1, isocitrate dehydrogenase 1; ACACB, acetyl-coenzyme A carboxylase beta; CD36, cluster of differentiation 36; FABP3, fatty acid binding protein 3; APOA1, Apolipoprotein A1; APOE, apolipoprotein E; VTN, vitronectin; SERPINC1, Serpin Family C Member 1; PLG, plasminogen; TTR, transthyretin; TF, transferrin; RPS23, ribosomal protein S23; RPS9, ribosomal protein S9.
